# Ultrastructure of human platelet concentrates after treatment with pathogen reduction technologies for prolonged storage

**DOI:** 10.3389/fmed.2025.1682909

**Published:** 2025-10-20

**Authors:** Mohammed Barham, Tobias Odenthal, Susanne M. Picker, Andrea Grandoch, Birgit S. Gathof, Wolfram F. Neiss

**Affiliations:** ^1^Department II of Anatomy, Faculty of Medicine, University Hospital Cologne, University of Cologne, Cologne, Germany; ^2^Department I of Anatomy, Faculty of Medicine, University Hospital Cologne, University of Cologne, Cologne, Germany; ^3^Transfusion Medicine, Faculty of Medicine, University Hospital Cologne, University of Cologne, Cologne, Germany; ^4^Department for Oral and Craniomaxillofacial and Plastic Surgery, Faculty of Medicine, University Hospital of Cologne, University of Cologne, Cologne, Germany

**Keywords:** blood system, INTERCEPT, MIRASOL-PRT, mitochondria, canalicular system, pathogen-reduction technology

## Abstract

**Background and objectives:**

Pathogen reduction technologies (PRTs) increase blood supply safety but may also increase platelet storage lesion, probably due to mitochondrial DNA damage. The purpose of this study was to investigate whether these changes are morphologically detectable.

**Materials and methods:**

Blood platelets were obtained by triple-dose apheresis collection (*n* = 8). Immediately after splitting, single units were left untreated (CONTROL) or treated with either psoralen-UVA (INTERCEPT) or riboflavin-UVB (MIRASOL). All platelet units were resuspended in platelet additive solution (INTERSOL or SSP+) and stored for up to seven days. Seven samples from each donation were examined by electron microscopy fresh, i.e., immediately after collection, and after1 day and 7 days of storage either untreated or treated with INTERCEPT or MIRASOL PRT. The volumes of mitochondria and of the canalicular system (CS) were measured.

**Results:**

Freshly isolated platelets (0 days storage) contained 2.4% mitochondria (volume density) and 4.5% CS (volume density). After 1 day of storage mitochondrial volume density was reduced to 1.5% in untreated, 1.3% in INTERCEPT-treated and 1.6% in MIRASOL-treated platelet concentrates, i.e., a loss of up to 37% of mitochondrial volume regardless of treatment. After 7 days storage mitochondrial volume density was 1.3, 1.3 and 1.5% respectively; neither at 1 nor at 7 days storage were any noteworthy differences between untreated, INTERCEPT or MIRASOL-treated platelets. In stark contrast to mitochondria the CS ballooned up to 88% in all groups. After 1 day of storage CS volume density was increased to 8.6% in untreated, 8.4% in INTERCEPT-treated and 7.0% in MIRASOL-treated platelet concentrates. After 7 days storage CS volume density was 8.0, 8.3 and 6.3% respectively; neither at 1 nor at 7 days storage were significant differences between untreated, INTERCEPT or MIRASOL-treated platelets. Only at 7 days a slight tendency of a smaller CS in MIRASOL versus INTERCEPT and untreated CONTROL groups was observed.

**Conclusion:**

Platelet mitochondrial volume shrinks and canalicular system swells within the first 24 h after collection and then both remain rather constant for up to seven days with or without PRT treatment. Pathogen reduction technology – both INTERCEPT and MIRASOL – does not increase morphological platelet storage lesion.

## 1 Introduction

Despite all efforts to increase the safety of blood platelet concentrates transfusion, the risk of contamination with a variety of pathogens is a real and major risk to patients (1–8). In addition, the useful timespan of platelet storage is severely limited due to deterioration of platelet quality and function over time (9, 10). Treatment of platelet concentrates with pathogen reduction technologies (PRTs) such as the INTERCEPT (I) Blood System (Cerus) (11–16) or the MIRASOL (M)-Pathogen Reduction Technology (Terumo BCT) (13–17) are used to inactivate most pathogen contamination, and thus enhance the safety of platelet concentrates (PCs) during the storage at 22 °C.

INTERCEPT is based on the psoralen compound amotosalen that selectively binds to nucleic acids and lipids, but not to proteins, and intercalates into the helical regions of DNA or RNA, forming tight cross-links when irradiated with UV light (wavelength of 320 to 400 nm). These cross-links prevent the separation of nucleic acid strands, which in turn prevents nucleic acid replication (18–23). MIRASOL is based on riboflavin that intercalates with DNA and RNA and, when exposed to 265 to 370 nm UV light, riboflavin-photolysis induces guanine oxidation, resulting in single-strand breaks. This treatment can provide up to 98% protection against transfusion of bacterially contaminated units at the most clinically relevant contamination levels of less than 20 CFU per product, as well as gives potential protection against protozoan infections such as *Babesia microti* in apheresis PLT products (23–26) and various emerging viruses and leucocytes (23). However, both INTERCEPT and MIRASOL have been shown to retard real-time PCR amplification of mitochondrial DNA, indicating mtDNA damage in platelets (19, 21, 27).

The major challenge for all pathogen reduction technologies (PRTs) is to maintain the clinical function of platelet concentrates, i.e., appropriate and sufficient retention of adhesive, aggregative and procoagulant protection to restore haemostasis in the recipient, not just platelet count (28). Furthermore, PRTs can have side effects on the platelet function and even be implicated in platelet refractoriness and alloimmunization (16). Several findings indicate that PRT can impair the responsiveness of platelets to agonists and trigger platelet activation (7). PLTs are known to be very sensitive to their microenvironment, and there is a reasonable issue that PRT treatment could enhance PLT storage lesion in apheresis PLTs (28). To our knowledge this is the first study to demonstrate the ultrastructure of PRT-treated platelet concentrates using pathogen-reduction techniques. Do PRTs cause morphological damages to mitochondria or other organelles of platelets?

The German Guideline Hemotherapy (29) specifies a maximal storage time of five days (5 × 24 h) for platelet products treated with amotosalen/UVA (29: Chapter 3.2.24, p. 54). According to the EU Guide to the preparation, use and quality assurance of blood components (2023) “the maximum storage time for platelets may be extended to 7 days depending on the pathogen inactivation technology and on the type of additive solution” [30: Chapter 5C, p. 253; compare also Andreu (31), Raval et al. (32), and Novelo-Garza and Benítez-Arvizu (33)]. Therefore, pathogen inactivation needs to be considered in the broad context of the history and future of transfusion safety for patients.

## 2 Materials and methods

Immediately after platelet collection from the donor, the fresh platelets were subjected to either PRT treatment (INTERCEPT or MIRASOL) or served as control.

Freshly collected and platelet concentrates stored for 1 and 7 days were prepared for and analysed by transmission electron microscopy in a batch at the same time. The volume densities of mitochondria and that of the total (open plus closed) canalicular system (CS) were measured by stereology. Mitochondrial volume is linearly related to `V_O2max_, i.e., respiratory function (34, 35) and mitochondria play an important role in platelet activation (36). The CS is an invaginated membrane system found within platelets that provides a conduit for the release of stored substances during activation (37). Its extent is directly proportional to the efficiency of granule release and platelet spreading (38, 39). A larger CS volume results in a faster hemostatic response.

### 2.1 Preparation of platelet concentrates

Following written informed consent donor eligibility was based on European Committee (40) and German guidelines and requirements for PLT donation (29). A total of nine triple-dose PLT collections were obtained from nine healthy volunteers using an apheresis collection device (Trima Accel, Version 5.1, Terumo BCT, formerly Gambro BCT, Lakewood, CO) according to the manufacturer’s instructions. The collection targets per donor were 10.0 × 10^11^ PLTs in 330 mL of autologous plasma. The whole collection units yielded a mean volume of 336 mL/donor (range 326–344 mL). Mean PLT dose was 9.5 × 10^11^ PLTs (range 8.8–10.7 × 10^11^) corresponding to a mean PLT concentration of 2,810 × 10^9^/l [range 2,620–3,138 × 10^9^/l; for further details see Picker et al. (28)]. At this point 1,000 μL of platelet concentrate was removed for immediate glutaraldehyde fixation and EM preparation (sample: Untreated after apheresis; 0 days storage). The remaining 325–343 mL PLT concentrate per donor were divided into three units (bags) prior to resuspension in PAS, as previously described (28, 41). All units were leucoreduced using the process-controlled leukoreduction system.

### 2.2 Treatment with pathogen-reduction technology

For each of the 9 donors one unit (C unit) remained untreated and was resuspended in 150 mL of INTERSOL plus 30 mL of saline to compensate for the addition of volume of the photosensitizers to the PRT-treated units. The plasma: PAS ratio was 35–40: 60–65 in all units.

One unit/donor received PRT treatment with the INTERCEPT Blood System (I unit; Cerus Corp., Concord, CA; https://interceptbloodsystem.com) and one unit/donor with the MIRASOL-PRT System (M unit; Terumo BCT, Zaventem, B; formerly Caridian BCT Biotechnologies, Lakewood, Co; https://www.terumobct.com). PRT treatment was started within two hours of the collection according to the manufacturer’s instructions, as described previously (28, 41–43) and performed as such.

#### 2.2.1 INTERCEPT PRT

110 mL PLT concentrate + 180 mL lNTERSOL = PAS-III = PAS-C (Fenwal, Inc., a Fresenius-Kabi company, Lake Zurich, IL; containing: 452 mg/100 mL NaCl, 305 mg/100 mL Na_2_HPO_4_, 105 mg/100 mL NaH_2_PO_4_, 318 mg/100 mL Na_3_-citrate, 442 mg/100 mL Na-acetate), add Amotosalen HCl to 150 μM/L final concentration. Illumination was performed automatically using the Intercept device at 3,0J/cm^2^ in the 320–400 nm range. The CAD (compound adsorption device) time ranged from 4 h 20 min (3 bags) to 13 h 10 min (3 bags) and to 15 h 50 min (3 bags) (28, p. 27).

#### 2.2.2 MIRASOL PRT

110 mL PLT concentrate, add 11 mL 500 μM/L riboflavin to 50 μM/L final concentration. 8–10 min 6.2 J/mL illumination at 265–370 nm with agitation at 25 °C. After illumination add 150 mL SSP + = PAS-E (MacoPharma, Langen, Germany; composition 69.3 mmoL/L NaCl, 28.2 mmoL/L NaH_2_PO_4_/Na_2_HPO_4_, 10.8 mmoL/L Na_3_-citrate, 32.5 mmoL/L Na-acetate, 5.0 mmoL/L KCl, 1.5 mmoL/L MgCl_2_/MgSO_4_) (41, p. 2312).

All 27 single units (3 units/donor x 9 donors) were then stored in PL2410 plastic containers for seven days at 22 ± 2 °C on a flat-bed agitator (Helmer Laboratories, Noblesville, IN) at 50–60 agitations/min (rpm). Two samples of 1,000 μL each were aseptically collected for TEM preparation from each unit on storage day 1 (i.e., the day after PRT treatment) and day 7.

### 2.3 Electron microscopy

All samples for this TEM study were collected from the identical bags of blood of the same donors and apheresis as those of the biochemical investigations of Picker et al. (28) at the respective same time. However, only the samples of eight donors were investigated by TEM, as the material of one donor was lost in transit.

#### 2.3.1 Control and PRT treatment

Immediately after apheresis and resuspension (pretreatment platelets, day zero, CONTROL) and PRT treatment (INTERCEPT or MIRASOL), throughout seven days of storage period, 7 samples from each donation were prepared for transmission electron microscopy by this specifically devised procedure:

1. Add 20 μL of 50% glutardialdehyde (Fluka) to 1,000 μL of plasma/platelet concentrate (without RBCs) in Eppendorf tube, mix 30 s on Vortex and fix 90 min at room temperature.
2. Spin 2 min at 8,000 g, discard supernatant, resuspend in modified Tyrode solution (143 mmoL/L NaCI + 5.6 mmoL/L KCl + 1.0 mmoL/L MgC1_2_ + 11.9 mmoL/L NaHC0_3_ + 3.2 mmoL/L NaH_2_P0_4_, pH 7.2–7.4, 300 mosmol) at room temperature.
3. Repeat step 2.
4. Spin 2 min at 8,000 g, discard supernatant, resuspend in 1% OsO_4_ in Tyrode solution (3 mL 4% OsO_4_ + 3 mL distilled H_2_O + 6 mL 2x conc. Tyrode) and postfix overnight (about 18 h) in the dark with slow agitation at room temperature.
5. Repeat step 2 three times, but spin the postfixed platelets at 10,000 g.
6. Spin at 10,000 g, discard supernatant, embed pellet in small volume of 3% Agar dissolved in Tyrode, add 80% ethanol and place Eppendorf tube standing (no agitation) at 4 °C to facilitate hardening of the Agar-enclosed pellet.
7. Remove pellet from Eppendorf tube and trim it into small cubes (about 2 mm x 2 mm x 2 mm), place probes in glass vials containing 70% acetone; store overnight standing at 4 °C.
8. Dehydrate with acetone and infiltrate with araldite CY212 (Durcopan ACM, Fluka, Switzerland) with slow agitation on a rotary mixer: 2× 5 min 70% acetone, 15 min 90% acetone, 4× 15 min 100% acetone, 45 min 3 parts acetone/1part Durcupan ACM M1; 45 min 2 parts acetone/2parts M1; 45 min 1 part acetone/3 parts M1, all changes at room temperature.
9. Overnight on slow rotary mixer at 50 °C pure M1, 60 min at 50 °C fresh M1 on rotary mixer; 3 h on rotary mixer at 40 °C pure Durcupan ACM M2.
10. Embed probes in pure M2 in polyethylene capsules (TAAB) and polymerize 48 h at 70 °C.

Of these araldite blocks ultrathin sections (30–40 nm, grey interference colour) were cut with a 35° diamond knife (Diatome, Switzerland) on a Leica Ultracut E, mounted on formvar/carbon-coated 200-mesh copper grids and contrasted with uranyl acetate and lead citrate solution. TEM was performed with a Zeiss EM109 (80 kV, 200 μm condenser and 30 μm objective apertures, TRS-1 K-Camera). Magnification of micrographs was calibrated by means of a cross-grating replica (2,160 lines/mm; Polaron, England).

The digital electron micrographs were stored and analyzed on the computer screen. To overcome inter- and intra-observer bias, electron micrography and image measurements were performed completely blinded.

### 2.4 Image analysis of electron micrographs

EM investigations were performed to quantify platelet components. Using the MATLAB & SIMULINK version 7.6.0324 program (R2008a, Licence number 115735, The MathWorks™), a total of 1,053 electron micrographs were digitally analyzed at a primary magnification of 12.000 × and the volumes of mitochondria and of the canalicular system within the platelets, i.e., the ratio of mitochondria and /CS volumes to total platelet volume (V/V), were measured (44, 45).

### 2.5 Statistical evaluation

Our results are expressed as mean ± standard deviation and were analyzed using computer software (SPSS 15.0 for windows, SPSS software GmbH, Munich, Germany). The significant difference was at *p* < 0.05, *post hoc* paired comparisons were performed using the Mann–Whitney U test. The difference between study groups was determined by two-way ANOVA using GraphPad Prism software.

## 3 Results

### 3.1 General ultrastructure

All platelets that we have observed, freshly prepared after apheresis or stored for 1 or 7 days, showed the same ultrastructure as described elsewhere for normal platelets (46). The only remarkable qualitative findings (i.e., findings directly evident to the eye) in our material were a depletion of glycogen particles after 7 days of storage and a lack of mitochondria and an abundance of large, swollen profiles of the canalicular system in all groups of stored platelets (1 or 7 days of storage; untreated, INTERCEPT OR MIRASOL PRT) compared to “normal” platelets immediately fixed after apheresis (day 0). Qualitatively judged, however, there was no discernible difference between the 6 groups of stored platelets, which is why we performed systematic sampling, applying strict rules of stereology (44, 45) to quantitatively measure the volume density (Vv) of mitochondria and the canalicular system.

### 3.2 Stereology of mitochondria and the canalicular system

**Figure 1:**
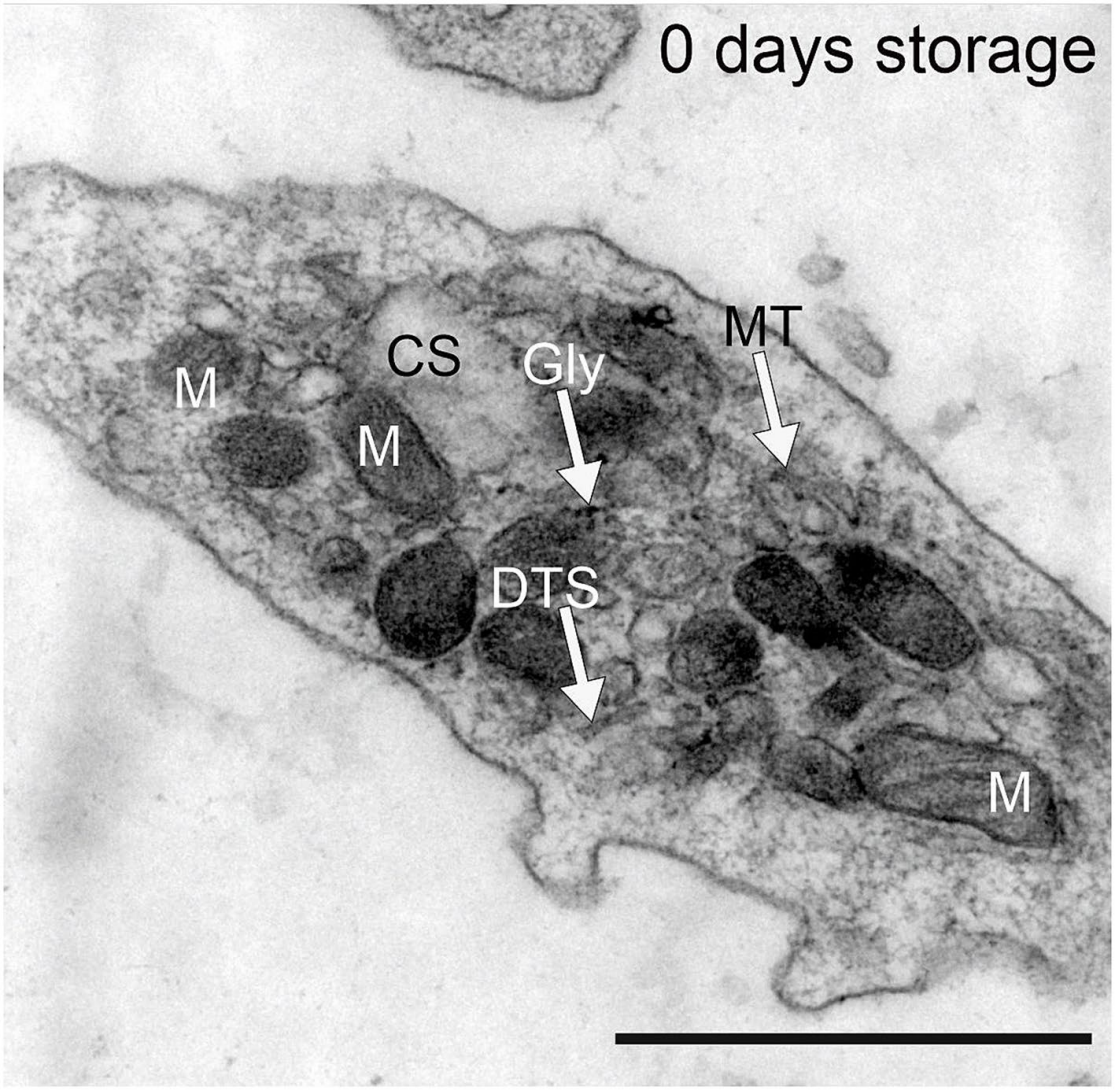
Platelets obtained by apheresis prior to PRT treatment from healthy Volunteers. DTS = dense tubular system; Gly = glycogen particle, M = mitochondria, MT = microtubules, CS = canalicular system. Scale bar 1 μm, primary magnification 20.000 × .

**Figure 2:**
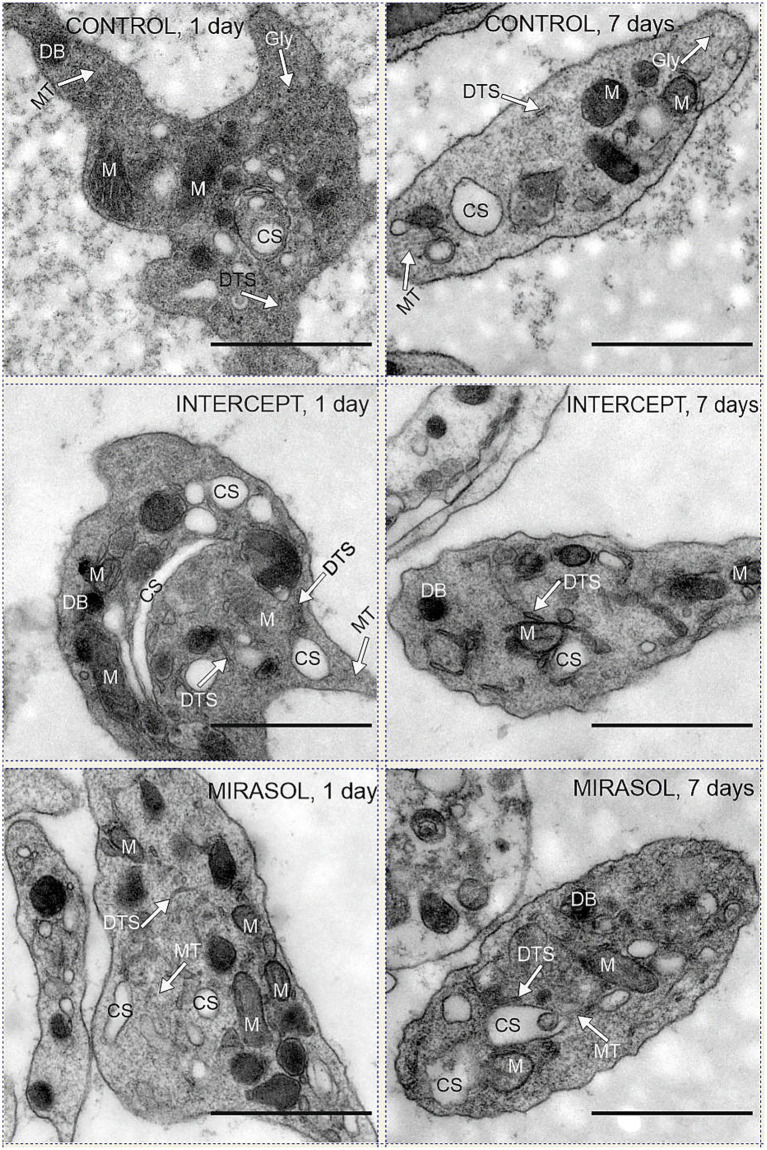
Mitochondria and CS in control, INTERCEPT- and MIRASOL treated platelets. Upper row: untreated controls PLTs suspended in PAS III (SSP+) and stored for one or seven days. Middle row: psoralen-UVA treated platelets (INTERCEPT) suspended in PAS III (SSP+) and stored for one or seven days. Lower row: Riboflavin-UVB treated platelets (MIRASOL) suspended in PAS III (SSP+) and stored for one or seven days. DB = dense body, DTS = dense tubular system; M = mitochondria, CS = canalicular system, MT = microtubules. Scale bars 1 μm, primary magnification of all electron micrographs 20.000 × .

Fresh human platelets (0 days storage) obtained directly by apheresis from the donor without treatment with pathogen reduction technology contained 2.4% volume of respiratory mitochondria and 4.5% volume of canalicular system (Figure 1; Tables 1, 2). After one day of storage, the volume density of mitochondria in the untreated control and treated platelet concentrates with INTERCEPT and MIRASOL was smaller than on day 0, 1.5% in Control, 1.3% in INTERCEPT or 1.6% in MIRASOL (Figure 2; Table 1). In contrast, the canalicular system had increased to 8.6% of cytoplasmic volume (Control), 8.4% (INTERCEPT) or 7.0% (MIRASOL) (Figure 2; Table 2). Significant differences were not observed at *p* < 0.05 between the untreated control and treated groups. Our results show that storage of platelets for 24 h resulted in a loss of up to 37% of mitochondrial volume density (1.3–1.6%) and an increase of up to 88% (7.0–8.6%) of CS in all experimental groups.

**Table 1:** Volume density of mitochondria in human platelet concentrates of eight donors.

Time	Untreated after apheresis	CONTROL untreated platelet concentrate	INTERCEPT treated platelet concentrate	MIRASOL treated platelet concentrate
0 days storage	2.4 ± 0.3%	_	_	_
1 day storage	_	1.5 ± 0.3%	1.3 ± 0.3%	1.6 ± 0.4%
7 days storage	_	1.3 ± 0.6%	1.3 ± 0.4%	1.5 ± 0.3%

Day 0 values were measured prior to splitting and PRT treatment. Each value is the mean ± SD.

**Table 2:** Volume density of canalicular system (CS) in human platelet concentrates of eight donors.

Time	Untreated after apheresis	CONTROL untreated platelet concentrate	INTERCEPT treated platelet concentrate	MIRASOL treated platelet concentrate
0 days storage	4.5 ± 1.8%	_	_	_
1 day storage	_	8.6 ± 2.3%	8.4 ± 1.9%	7.0 ± 1.3%
7 days storage	_	8.0 ± 1.4%	8.3 ± 1.4%	6.3 ± 2.2%

Day 0 values were measured prior to splitting and PRT treatment. Each value is the mean ± SD.

Storage of platelets for an additional six days (7 days storage) did not change the structure and content of mitochondria and CS in all groups. Mitochondria remained on average 1.3–1.5% (1.3% each for Control and INTERCEPT or 1.5% for MIRASOL) (Figure 2; Table 1), and the CS contents were 8.0, 8.3% or 6.3%, respectively on average (Figure 2; Table 2). Statistically, the values were not significantly different at *p* < 0.05 compared to 1-day of storage, although after 7 days of storage there was a slight tendency towards a lower CS volume in the MIRASOL-treated group compared to untreated or INTERSOL-treated platelets.

## 4 Discussion

Resting platelets have been shown to have high mitochondrial activity compared to other blood cell types (47). Mitochondria are responsible for the production of ATP through oxidative phosphorylation, which is essential for cellular energy needs. In platelets, adequate ATP production is essential to maintain their function and they also play a role in buffering intracellular calcium, which is critical for platelet activation and aggregation, i.e., haemostasis and thrombosis. Mitochondrial volume is an important factor influencing their function and energy metabolism and is a key aspect of cell biology that reflects the space occupied by mitochondria and is an indicator of mitochondrial content and activity within the platelet. It is typically assessed using advanced imaging techniques such as electron microscopy. Larger mitochondrial volumes typically indicate greater energy production capacity and potentially greater functional capacity (48, 49), and may allow for better calcium handling, thereby influencing the platelet response during the coagulation process. Mitochondrial volume in relation to platelet function affects a range of physiological and pathological processes, from routine haemostasis to complex cerebrovascular, neurological and metabolic diseases (50).

Before discussing the effects of pathogen reduction treatment and storage on platelet concentrates the normal values must be considered, i.e., volume density of mitochondria and canalicular system in circulating, not yet activated platelets.

Obviously, it is impossible to fix the ultrastructure of platelets inside the human blood stream, they have to be collected in some way prior to fixation for electron microscopy. Whether any – and if so which – changes in ultrastructure may occur during collection, cannot be ascertained. Table 3 shows a comparison of normal data as found in the literature. The quickest and most straightforward method of preparation was cannulation of a human vein with a syringe already containing the highly toxic fixative solution and aspiration of blood into the syringe (Table 3: “immediately fixed blood”; 51–53). Today this approach appears incompatible with medical ethics. Other methods are the collection and subsequent fixation of citrate-treated blood (51–54) and – standard today – apheresis followed by fixation (55, this study).

**Table 3:** Normal data of freshly collected platelets.

References	Method of preparation	Mitochondria	CS
Morgenstern and Kho (51)	Citrate-treated blood	2.1%	n.a.
Morgenstern and Kho (51)	Immediately fixed blood	2.8%	n.a.
Morgenstern (52)	Immediately fixed or citrate-treated blood	ca. 3.0%	ca. 16.0%
Stahl et al. (53)	Immediately fixed or citrate-treated blood	1.1%	6.2%
de Duyvené Wit et al. (54) (mean of all density groups)	Citrate-treated blood	2.3%	10.1%
Klinger and Klüter (55)	Apheresis: day 1	n.a.	11.3%
**This study**	**Apheresis: freshly prepared**	**2.4%**	**4.5%**

All numbers represent mean values of the volume density of mitochondria and CS compared to the literature. n.a. = not applicable.

Pertaining to mitochondria all studies (51, 52, 54, 55) except for Stahl et al. (53: 1.1%) reported a normal volume density of 2.1 to 2.8% (Table 3), with our data of 2.4% in the middle of this range, suggesting that absolute platelet cell volume and platelet mitochondrial volume are little affected by the method of platelet collection.

Regarding the CS, the reported normal volume density ranges from our measurements of 4.5% up to 16% (Table 3). There is no explanation for these divergences apart from a hypothetical higher susceptibility of the CS to variations in cell preparation for TEM. Both in our study and likewise in that of Klinger and Klüter (55) the same preparation method was used for specimens after different storage times. Without PRT we observed an increase in CS volume density from 4.5% (day 0) to 8.0% (day 7; see Table 2) and Klinger and Klüter (55) reported an increase from 11.3% (day 1) to 16.2% [day 8; see Table 1 in Klinger and Klüter (55)]. Hence, any increase of the CS probably indicates some progress in storage lesion, whereas mitochondria dwindle during storage (see below).

In previous work on different pathogen reduction technologies, differences in mitochondria-based oxidative phosphorylation have been reported: upregulation after riboflavin-UVB treatment (i.e., increased anoxidative glycolytic flux and oxidative phosphorylation) and impairment of mitochondria-based respiration after psoralen-UVA treatment (28, 41–43). Obviously, these biochemical differences cannot be detected morphologically in the platelet concentrates. However, our study appears to be the first to document ultrastructural characteristics of the platelet storage lesion as it develops after pathogen reduction treatment. As pointed out in the review of Escolar et al. (22) “The term ‘storage lesion’ was coined to describe a series of structural and functional alterations during the storage of red blood cells and has also been applied to platelet concentrates. This lesion starts early during the process of collection, increases progressively during storage, and compromises the *in vivo* function of transfused platelets” [see (56, 57)]. Our morphological data differ in one point from previous descriptions of the storage lesion: In our material mitochondrial volume and CS volume per platelet volume did not change progressively during storage, but rather abruptly during preparation of the platelet concentrate, i.e., from platelets freshly fixed immediately after apheresis to platelets stored for 1 day after preparation – regardless of the method of treatment.

Storage of platelets for 24 h resulted in a decrease in the volume of mitochondrial content by up to 37% (1.3–1.6%, Table 1) and an increase in CS by up to 88% (7.0–8.6%, Table 2) in all experimental groups. In contrast, storage of platelets for an additional six days did not any more change the outcome. Mitochondrial content remained at 1.3–1.5% of platelet volume and CS at 6.3–8.3%.

In summary, the data of untreated, INTERCEPT-treated and MIRASOL-treated platelets stored for 1 or 7 days are almost identical, when the standard deviations of the data are considered (see Tables 1, 2). This strongly suggests that *PRT either with INTERCEPT or MIRASOL does not afflict platelets more than “plain” storage in PAS*. Only at 7 days a very slight tendency of a smaller CS in MIRASOL versus INTERCEPT and untreated CONTROL groups was observed. This is consistent with previous results, in which treatment with MIRASOL was slightly superior to INTERCEPT due to resistance to hyperosmotic shock (HSR) and aggregation with TRAP-6 (Thrombin Receptor Activator Peptide 6) (28, 41).

## 5 Conclusion

This study demonstrates that the content of mitochondria and the canalicular system is remarkably altered within the first 24 h after apheresis: Mitochondrial volume shrinks up to 37%, CS volume swells up to 88%, when platelets fixed immediately after apheresis and resuspension (day 0) are compared to platelets fixed after 1 day of storage. These changes are identical in platelets stored in PAS but left otherwise untreated and in platelets that have received either INTERCEPT or MIRASOL PRT treatment. The initial changes remain fairly constant for up to 7 days of storage in all groups. Our data suggest that Pathogen Reduction Technology – both INTERCEPT and MIRASOL – does not increase morphological platelet storage lesion as compared to simple storage in PAS.

## Data Availability

The datasets presented in this article will be made available by the corresponding author, without undue reservation.

## References

[1] Spiess BD. Platelet transfusions: the science behind safety, risks and appropriate applications. Best Pract Res Clin Anaesthesiol. (2010) 24:65–83. doi: 10.1016/j.bpa.2009.11.001, PMID: 20402171

[2] Busch MP Kleinman SH Nemo GJ. Current and emerging infectious risks of blood transfusions. JAMA. (2003) 289:959–62. doi: 10.1001/jama.289.8.959, PMID: 12597733

[3] Jacobs MR Good CE Lazarus HM Yomtovian RA. Relationship between bacterial load, species virulence, and transfusion reaction with transfusion of bacterially contaminated platelets. Clin Infect Dis. (2008) 46:1214-1–1214-1220. doi: 10.1086/52914d 18444858

[4] Müller B Walther-Wenke G Kalus M Alt T Bux J Zeiler T. Routine bacterial screening of platelet concentrates by flowcytometry and its impact on product safety and supply. Vox Sang. (2014) 108:209–18. doi: 10.1111/vox.12214, PMID: 25469957

[5] Hong H Xiao W Lazarus HM Good CE Maitta RW Jacobs MR. Detection of septic transfusion reactions to platelet transfusions by activeand passive surveillance. Blood. (2016) 127:496–502. doi: 10.1182/blood-2015-07-655944 26598718

[6] Fenwick AJ Gehrie EA Marshall CE Tobian AR Shrestha R Kacker S. Secondary bacterial culture of platelets to mitigate transfusion-associated sepsis: a 3-year analysis at a large academic institution. Transfusion. (2020) 60:2021–8. doi: 10.1111/trf.15978, PMID: 32750171 PMC10007897

[7] Osman A Hitzler WE Provost P. The platelets' perspective to pathogen reduction technologies. Platelets. (2018) 29:140–7. doi: 10.1080/09537104.2017.1293806, PMID: 28355122

[8] Salunkhe V van der Meer PF de Korte D Seghatchian J Gutiérrez L. Development of blood transfusion product pathogen reduction treatments: a review of methods, current applications and demands. Transfus Apher Sci. (2015) 52:19–34. doi: 10.1016/j.transci.2014.12.016, PMID: 25620756

[9] Aubron C Flint AWJ Yves Ozier Y McQuilten Z. Platelet storage duration and its clinical and transfusion outcomes: a systematic review. Crit Care. (2018) 22:185. doi: 10.1186/s13054-018-2114-x 30077181 PMC6091146

[10] Trochanowska-Pauk N Walski T Bohara R Mikolas J Kubica K. Platelet storage—problems, improvements, and new perspectives. Int J Mol Sci. (2024) 25:7779. doi: 10.3390/ijms25147779, PMID: 39063021 PMC11277025

[11] Leitner GC Hagn G Niederstaetter L Bileck B Plessl-Walder K Horvath M. Intercept pathogen reduction in platelet concentrates, in contrast to gamma irradiation, induces the formation of trans-arachidonic acids and affects eicosanoid release during storage. Biomolecules. (2022) 12:1258. doi: 10.3390/biom12091258 36139096 PMC9496540

[12] Slichter SJ Raife TJ Davis K Rheinschmidt M Buchholz DH Corash L. Platelets photochemically treated with amotosalen HCl and ultraviolet a light correct prolonged bleeding times in patients with thrombocytopenia. Transfusion. (2006) 46:731–40. doi: 10.1111/j.1537-2995.2006.00791.x, PMID: 16686840

[13] Rebulla P Vaglio S Beccaria F Bonfichi M Carella A Chiurazzi F. Clinical effectiveness of platelets in additive solution treated with two commercial pathogen-reduction technologies. Transfusion. (2017) 57:1171–83. doi: 10.1111/trf.14042 28236335

[14] Norris PJ Kaidarova Z Maiorana E Milani S Lebedeva M Busch MP. Ultraviolet light-based pathogen inactivation and alloimmunization after platelet transfusion: results from a randomized trial. Transfusion. (2018) 58:1210–7. doi: 10.1111/trf.14534, PMID: 29473173

[15] Lu W Fung M. Platelets treated with pathogen reduction technology: current status and future direction. F1000Research. (2020) 9:40. doi: 10.12688/f1000research.20816.1 PMC6979468 32047608

[16] Pati I Masiello F Pupella S Cruciani M Vincenzo De Angelis V. Efficacy and safety of pathogen-reduced platelets compared with standard apheresis platelets: a systematic review of RCTs. Pathogens. (2022) 11:639. doi: 10.3390/pathogens11060639 35745493 PMC9231062

[17] Fast LD DiLeone G Marschner S. Inactivation of human white blood cells in platelet products after pathogen reduction technology treatment in comparison to gamma irradiation. Transfusion. (2011) 51:1397–404. doi: 10.1111/j.1537-2995.2010.02984.x, PMID: 21155832

[18] Lin L Conlan MG Tessman J Cimino G Porter S. Amotosalen interactions with platelet and plasma components: absence of neoantigen formation after photochemical treatment. Transfusion. (2005) 45:1610–20. doi: 10.1111/j.1537-2995.2005.00554.x, PMID: 16181212

[19] Bruchmüller I Janetzko K Bugert P Mayaudon V Corash K Lin L. Polymerase chain reaction inhibition assay documenting the amotosalen-based photochemical pathogen inactivation process of platelet concentrates. Transfusion. (2005) 45:1464–72. doi: 10.1111/j.1537-2995.2005.00553.x 16131379

[20] Pineda A McCullough J Benjamin RJ Cable R Strauss RG Burgstaler E. Pathogen inactivation of platelets with a photochemical treatment with amotosalen HCl and ultraviolet light: process used in the SPRINT trial. Transfusion. (2006) 46:562–71. doi: 10.1111/j.1537-2995.2006.00761.x, PMID: 16584432

[21] Bakkour S Chafets DM Wen L Dupuis k Castro G Green JG. Assessment of nucleic acid modification induced by amotosalen and ultraviolet a light treatment of platelets and plasma using real-time polymerase chain reaction amplification of variable length fragments of mitochondrial DNA. Transfusion. (2016) 56:410–20. doi: 10.1111/trf.13360 26446053

[22] Escolar G Diaz-Ricart M McCullough J. Impact of different pathogen reduction technologies on the biochemistry, function, and clinical effectiveness of platelet concentrates: an updated view during a pandemic. Transfusion. (2022) 62:227–46. doi: 10.1111/trf.16747, PMID: 34870335 PMC9300014

[23] Liu H Wang X. Pathogen reduction technology for blood component: a promising solution for prevention of emerging infectious disease and bacterial contamination in blood transfusion services. J Photochem Photobiol. (2021) 8:1–6. doi: 10.1016/j.jpap.2021.100079

[24] Lachert E Woźniak J Antoniewicz-Papis J Krzywdzińska A Kubis J Mikołowska A. Study of CD69 antigen expression and integrity of leukocyte cellular membrane in stored platelet concentrates following irradiation and treatment with Mirasol^®^ PRT system. Adv Clin Exp Med. (2017) 26:7–13. doi: 10.17219/acem/68290, PMID: 28397426

[25] Lachert E Kubis J Antoniewicz-Papis J Rosiek A Woźniak J Piotrowski D. Quality control of riboflavin-treated platelet concentrates using Mirasol^®^ PRT system: polish experience. Adv Clin Exp Med. (2018) 27:765–72. doi: 10.17219/acem/68901, PMID: 29877637

[26] Caudrillier A Mallavia B Rouse L Marschner S Looney MR. Transfusion of human platelets treated with Mirasol pathogen reduction technology does not induce acute lung injury in mice. PLoS One. (2015) 10:e0133022. doi: 10.1371/journal.pone.0133022, PMID: 26176623 PMC4503436

[27] Bakkour S Chafets DM Wen L van der Meer PF Mundt JM Marschner S. Development of a mitochondrial DNA real-time polymerase chain reaction assay for quality control of pathogen reduction with riboflavin and ultraviolet light. Vox Sang. (2014) 107:351–9. doi: 10.1111/vox.12173, PMID: 24976130

[28] Picker SM Oustianskaia L Schneider V Gathof BS. Functional characteristics of apheresis-derived platelets treated with ultraviolet light combined with either amotosalen-HCl (S-59) or riboflavin (vitamin B2) for pathogen-reduction. Vox Sang. (2009) 97:26–33. doi: 10.1111/j.1423-0410.2009.01176.x, PMID: 19302416

[29] Bundesärztekammer (2023) Richtlinie zur Gewinnung von Blut und Blutbestandteilen und zur Anwendung von Blutprodukten (Deutsche Richtlinie Hämotherapie, 2023). 124 S. Available online at: https://www.bundesaerztekammer.de/fileadmin/user_upload/BAEK/Themen/Medizin_und_Ethik/Richtlinie-Haemotherapie-2023_neu2.pdf

[30] Council of Europe (2023) Guide to the preparation, use and quality Assurance of Blood Components, European Directorate for the Quality of medicines & HealthCare (edqm.Eu) Strasbourg, France, Council of Europe Publishing, 21st edition, 462. Available online at: https://www.edqm.eu/en/blood-guide

[31] Andreu G. Blood components and good practices in transfusion. Presse Med. (2015) 44:165–77. doi: 10.1016/j.lpm.2014.06.032, PMID: 25542709

[32] Raval JS Griggs JR Fleg A. Blood product transfusion in adults: indications, adverse reactions, and modifications. Am Fam Physician. (2020) 102:30–8. 32603068

[33] Novelo-Garza B Benítez-Arvizu G. Obtaining blood components in blood banks. Rev Med Inst Mex Seguro Soc. (2023) 61:52–8. PMC10395912 36378143

[34] Schwerzmann K Hoppeler H Kayar SR Weibel ER. Oxidative capacity of muscle and mitochondria: correlation of physiological, biochemical, and morphometric characteristics. Proc Natl Acad Sci USA. (1989) 86:1583–7. doi: 10.1073/pnas.86.5.1583, PMID: 2922400 PMC286742

[35] Weibel ER Hoppeler H. Exercise-induced maximal metabolic rate scales with muscle aerobic capacity. J Exp Biol. (2005) 208:1635–44. doi: 10.1242/jeb.01548, PMID: 15855395

[36] Perales Villarroel JP Figueredo R Guan Y Tomaiuolo M Karamercan MA Welsh J. Increased platelet storage time is associated with mitochondrial dysfunction and impaired platelet function. J Surg Res. (2013) 184:422–9. doi: 10.1016/j.jss.2013.05.097, PMID: 23830370 PMC3879371

[37] Selvadurai MV Hamilton JR. Structure and function of the open canalicular system – the platelet’s specialized internal membrane network. Platelets. (2018) 29:319–25. doi: 10.1080/09537104.2018.1431388, PMID: 29442528

[38] Heijnen H Van Der Sluijs P. Platelet secretory behaviour: as diverse as the granules… or not? J Thromb Haemost. (2015) 13:2141–51. doi: 10.1111/jth.13147 26391322

[39] Pokrovskaya ID Tobin M Desai D Joshi S Kamykowski JA Zhang G. Canalicular system reorganization during mouse platelet activation as revealed by 3D ultrastructural analysis. Platelets. (2021) 32:97–104. doi: 10.1080/09537104.2020.1719993 32000578 PMC7392809

[40] Paul-Ehrlich-Institute, G. M. A. A. (2005). Guidelines for the collection of blood and blood components and the usage of blood products (hemotherapy). Rev. ed. Cologne, Deutscher Ärztevelag.

[41] Picker SM Schneider V Oustianskaia L Gathof BS. Cell viability during platelet storage in correlation to cellular metabolism after different pathogen reduction technologies. Transfusion. (2009) 49:2311–8. doi: 10.1111/j.1537-2995.2009.02316.x, PMID: 19624608

[42] Kaiser-Guignard J Canellini G Lion N Abonnenc M Osselaer J-C Tissot J-D. The clinical and biological impact of new pathogen inactivation technologies on platelet concentrates. Blood Rev. (2014) 28:235–41. doi: 10.1016/j.blre.2014.07.005, PMID: 25192602

[43] Lanteri MC Santa-Maria F Laughhunn AG Yvette A Picard-Maureau M Payrat J-M. Inactivation of a broad spectrum of viruses and parasites by photochemical treatment of plasma and platelets using amotosalen and ultraviolet a light. Transfusion. (2020) 60:1319–31. doi: 10.1111/trf.15807 32333396 PMC7317863

[44] Weibel ER. Stereological methods. Volume 1. London: Academic Press (1979). 415 p.

[45] Cruz-Orive LM Weibel ER. Recent stereological methods for cell biology: a brief survey. Am J Phys Lung Cell Mol Phys. (1990) 258:L148–56. doi: 10.1152/ajplung.1990.258.4.L148, PMID: 2185653

[46] White JG Krumwiede M. Some contributions of electron microscopy to knowledge of human platelets. Thromb Haemost. (2007) 98:69–72. doi: 10.1160/TH07-04-0303, PMID: 17597993

[47] Kramer PA Ravi S Chacko B Johnson MS Darley-Usmar VM. A review of the mitochondrial and glycolytic metabolism in human platelets and leukocytes: implications for their use as bioenergetic biomarkers. Redox Biol. (2014) 2:206–10. doi: 10.1016/j.redox.2013.12.026, PMID: 24494194 PMC3909784

[48] Garcia-Souza LF Oliveira MF. Mitochondria: biological roles in platelet physiology and pathology. Int J Biochem Cell Biol. (2014) 50:156–60. doi: 10.1016/j.biocel.2014.02.015, PMID: 24569121

[49] Melchinger H Jain K Tyagi T Hwa J. Role of platelet mitochondria: life in a nucleus-free zone. Front Cardiovasc Med. (2019) 6:153. doi: 10.3389/fcvm.2019.00153, PMID: 31737646 PMC6828734

[50] Ma Y Jiang Q Yang B Hu X Shen G Shen W. Platelet mitochondria, a potent immune mediator in neurological diseases. Front Physiol. (2023) 14:1210509. doi: 10.3389/fphys.2023.1210509, PMID: 37719457 PMC10502307

[51] Morgenstern E Kho A. Morphometrische Untersuchungen an Blutplättchen: Veränderungen der Plättchenstruktur bei Poseudopodienbildung und Aggregation. Cytobiologie (Stuttg). (1977) 15:233–49.

[52] Morgenstern E. Ultracytochemistry of human blood platelets. Prog Histochem Cytochem. (1980) 12:1–86. doi: 10.1016/s0079-6336(80)80006-4 6987714

[53] Stahl K Themann H Dame WR. Ultrastructural morphometric investigations on normal human platelets. Haemostasis. (1978) 7:242–51. doi: 10.1159/000214265, PMID: 658788

[54] de Duyvené Wit LJ Badenhorst PN Heyns AD. Ultrastructural morphometric observations on serial sectioned human blood platelet subpopulations. Eur J Cell Biol. (1987) 43:408–11. 3622527

[55] Klinger MHF Klüter H. Morphological changes in thrombocytes during blood bank storage. An ultrastructural morphometric study. Ann Anat. (1993) 175:163–70. doi: 10.1016/s0940-9602(11)80176-7, PMID: 8489036

[56] Rinder HM Murphy M Mitchell JG Stocks J Ault KA Hillman RS. Progressive platelet activation with storage: evidence for shortened survival of activated platelets after transfusion. Transfusion. (1991) 31:409–14. doi: 10.1046/j.1537-2995.1991.31591263195.x, PMID: 1710840

[57] Devine DV Serrano K. The platelet storage lesion. Clin Lab Med. (2010) 30:475–87. doi: 10.1016/j.cll.2010.02.002, PMID: 20513565

