# Supplementary material for: Ultrastructure of human platelet concentrates after treatment with pathogen reduction technologies for prolonged storage
Source: Front Med (Lausanne). 2025 Oct 20;12:1682909. doi: 10.3389/fmed.2025.1682909 (PMC12580302; doi:10.3389/fmed.2025.1682909)
Supplement: Supplementary file 1 [file Data_Sheet_1.pdf]

Guide to the preparation, use and quality assurance of BLOOD COMPONENTS

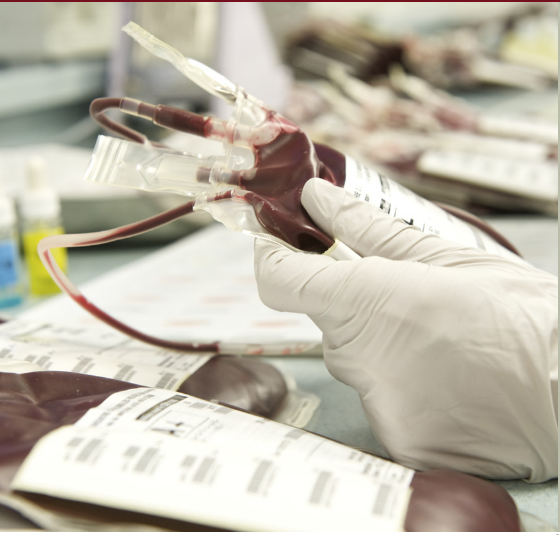

European Committee (Partial Agreement) on Blood Transfusion (CD-P-TS)

EDQM
19th Edition
2017

European Directorate for the Quality of Medicines & HealthCare | Direction européenne de la qualité du médicament & soins de santé

COUNCIL OF EUROPE
CONSEIL DE L'EUROPE

Guide to the preparation, use and quality assurance of blood components

Recommendation No. R (95) 15

19th Edition

European Directorate for the Quality of Medicines & HealthCare

The *Guide to the preparation, use and quality assurance of blood components* is published by the European Directorate for the Quality of Medicines & HealthCare of the Council of Europe (EDQM).

All rights conferred by virtue of the International Copyright Convention are specifically reserved to the Council of Europe and any reproduction or translation requires the written consent of the publisher.

Director of the Publication: Dr S. Keitel

Page layout and cover: EDQM

European Directorate for the Quality
of Medicines & HealthCare (EDQM)
Council of Europe
7, allée Kastner
CS 30026
F-67081 STRASBOURG
FRANCE

Website: www.edqm.eu
To order: www.edqm.eu/store
FAQs & EDQM HelpDesk: www.edqm.eu/hd

ISBN 978-92-871-8415-3
© Council of Europe, 2017
Printed at the Council of Europe

Supported by the European Union.

Contents

FOREWORD 23

EUROPEAN COMMITTEE (PARTIAL AGREEMENT) ON BLOOD TRANSFUSION (CD-P-TS) 29
- Chair 29
- Members 29
- Observers 35

MEMBERS OF THE AD HOC GROUP (GTS) 39
- Chair 39
- Members 39

Recommendation No. R (95) 15 45

Guide to the preparation, use and quality assurance of blood components 49

GOOD PRACTICE GUIDELINES 50
- Introductory note 50

Good Practice Guidelines for blood establishments and hospital blood banks 52

1. General principles 52

2. Personnel and organisation 61

3. Premises 65
4. Equipment and materials 69
5. Documentation 91
6. Blood collection, testing and processing 101
7. Storage and distribution 114
8. Outsourced activities management 116
9. Non-conformance and recall 119
10. Self-inspection, audits and improvements 125
11. Quality monitoring and control 126

PRINCIPLES 129

Chapter 1 Introduction 131

Chapter 2 Principles of donor selection 133
1. General remarks 133
2. Overview 134
3. Medical assessment of the donor 135
Age of the donor 135
Hazardous occupations 135
Donor deferral 136
Conditions requiring permanent deferral 136
Conditions requiring temporary deferral (suspension) 136
Vaccination 136
Conditions requiring individual assessment 136
Post-donation information 137
Infectious diseases 138
History of malignancy 140
4. Specific considerations for donors of different components . . . 141
Quantity of whole blood donation 141
Frequency of whole blood donation 142
Laboratory examination before donation 143
Apheresis donors 144
Designated donations 148
Directed donations 149

Chapter 3 Principles of blood collection 151

1. Overview 151
2. Premises for donor sessions 151
3. Equipment used at blood donation sessions 152
4. Pre-donation checks and labelling 152
5. Venepuncture 152
Preparation of the venepuncture site 152
Successful venepuncture and proper mixing 153
Handling of filled containers and samples 154
6. Apheresis 155
Pre-medication and apheresis 155
Automated apheresis 155
Manual apheresis 155
7. Repository of archive samples 155
8. Management of adverse reactions in donors 155
Prevention of adverse reactions in donors 157
Treatment of adverse reactions in donors 157
Documentation of adverse reactions in donors 158
Information for a donor with adverse reactions 158
9. Donor clinic documentation 158

Chapter 4 Principles of blood component processing 161
1. Overview 161
2. Processing procedures 162
3. Choice of anticoagulant and bag system 162
4. Centrifugation of whole blood derived blood components 164
5. Leucocyte depletion 167
6. Freezing and thawing of plasma 167
Rationale 167
Methods of freezing 168
Methods of thawing 169
Cryoprecipitation 169
7. Open and closed systems and sterile connection devices 170
8. Irradiation of cellular blood components 170
9. Prevention of CMV transmission 171
10. Pathogen reduction technologies 172
11. Purity of components 173

12. Bacterial safety of blood components 174
Overview 174
Quality control for aseptic collection and processing of blood components 175
Release as 'culture-negative to date' after bacteriological testing of all platelets 175
13. Storage of blood components 176
Equipment 176
Storage at + 2 to + 6 °C 176
Storage of frozen plasma components 177
Storage at + 20 to + 24 °C 177
Aspects of red cell preservation 177
Additive solutions 178
Micro-aggregates in blood components 179
Red cell preparations 179
Platelet preparations 179
Granulocyte preparations 180
Plasma components could be stored at different temperatures . 180
14. Transport of blood components 181
Transport of standard red cell components 181
Transport of platelet components 181
Transport of frozen plasma components 182
15. Component information and principles of labelling 182

Chapter 5 Principles of blood component monographs 183
1. Definition and properties 184
2. Preparation 184
3. Requirements and quality control 184
4. Storage and transport 185
5. Labelling 185
6. Warnings 185

Chapter 6 Principles of blood components for intrauterine, neonatal and infant use 187
1. Overview 187
2. Components for intrauterine transfusions 188

3. Components for neonatal exchange transfusion 188
4. Red cells for neonatal and infant small volume transfusion . . . 189
5. Fresh frozen plasma for neonatal and infant use 190
6. Platelets for neonatal and infant use 190

Chapter 7 Principles of autologous transfusion 193
1. Overview 193
2. Pre-deposit autologous transfusion 195
Patient selection 195
Blood collection 196
Preparation, storage and distribution of pre-deposit autologous components 197
Labelling 197
Storage 197
Records 197
Audit 198
3. Red cell salvage 198
Collection system 198
Processing system 199
Indications for the use of cell salvage 199
Parameters for quality control 199
Precautions 200

Chapter 8 Principles of immunohaematology 201
1. Overview 201
2. Immunohaematological testing 202
Blood group testing 202
3. Antibody screening and identification 203
Pre-transfusion testing 204
Quality control 206
Internal quality control 206
Quality control of reagents and techniques 206
Quality control of equipment 207
External quality assurance (proficiency testing) 207

Chapter 9 Principles of screening for markers of infection 209
1. Overview 209

2. Algorithm for infectious marker screening and confirmatory testing 210
3. Confirmatory testing 212
Anti-HIV-1/2, anti-HCV and HBsAg 212
Anti-HTLV-I-II 213
4. Nucleic acid screening 213
5. Additional screening 213
Syphilis screening 213
Anti-HBc screening 214
CMV screening 214
Malaria screening 214
Trypanosoma cruzi screening 215
NAT screening 215

Chapter 10 Principles of haemovigilance 217
1. Overview 217
2. Prerequisites for implementation of a haemovigilance network 218
Traceability of blood components 218
Co-operation between blood establishments, hospital blood banks and clinical departments 220
3. Types of adverse reactions and adverse events collected in a haemovigilance network 221
Adverse reactions in patients 221
Adverse reactions in donors 222
Adverse events 223
Device defects 224
4. Tracing and recall of potentially infectious donations for HIV, HCV or HBV (look-back) 225
Post-transfusion infection in a recipient reported to the blood establishment 225
Post-donation information 226
Recall of blood components 226
Tracing of recipients of potentially infectious blood donations (look-back/review) 226

5. Contracts between blood establishments and hospitals for haemovigilance 227
Minimum information to be captured in the initial incident report at hospital level 227
6. Reporting haemovigilance data 228
Standardisation of reporting 228
Data analysis 228
Information sent to the haemovigilance database 228
Component information 228
Information about severity 229
Information about imputability 229
Information about the type of adverse events and reactions 230

Chapter 11 Principles of clinical use of blood 231
1. Overview 231
2. Decision to transfuse 233
Patient blood management 234
3. Completion of the transfusion request form, identification of patient and blood sampling 235
4. Correct identification of the patient and obtaining a pre-transfusion sample 235
5. Testing within the laboratory 236
6. Selection and issue of appropriate blood components 236
Handling and storage of blood components in hospital clinical areas 237
7. Administration of blood components 237
Special precautions 238
Transfusion monitoring 238
Management and reporting of transfusion reactions 239
Traceability and haemovigilance 241
8. Hospital transfusion committees 241

STANDARDS 243

Chapter 1 Introduction 245

Chapter 2 Standards for selection of donors 247

1. Overview 247
2. Information to be provided to the donor 247
3. Medical assessment of the donor 249
Donor eligibility 249
Questionnaire and interview 250
Donor details 250
Age of the donor 250
Donor appearance and inspection 251
4. Donor deferral 251
Infectious diseases 254
5. Specific standards for donors of different types of components 258
Whole blood donors 258
Quantity of donation 258
Apheresis donors 259
6. Post-donation information 262

Chapter 3 Standards for collection of blood and blood components 263
1. Premises for donor sessions 263
2. Procedures and equipment used at blood donation sessions 264
3. Pre-donation checks 264
4. Labelling 264
5. Venepuncture, bleeding and mixing 265
Preparation of the venepuncture site 265
Successful venepuncture and proper mixing 266
6. Handling of filled containers and samples 267
7. Special requirements for apheresis 267
Return of red blood cells of donors undergoing manual apheresis 268
8. Repository of archive samples 268

Chapter 4 Standards for the processing, storage and distribution of blood components 269
1. Processing 269
2. Component labelling and information 270

3. Release of blood components 271
4. Storage and distribution 273
5. Irradiation of blood components 274
6. Leucocyte depletion 275
7. Bacterial safety 275

Chapter 5 Component monographs 277

Component monographs
Part A. Whole Blood components 279
1. Whole Blood 280
Definition and properties 280
Preparation 280
Requirements and quality control 280
Storage and transport 281
Labelling 281
Warnings 282
2. Whole Blood, Leucocyte-Depleted 283
Definition and properties 283
Preparation 284
Requirements and quality control 284
Storage and transport 285
Labelling 285
Warnings 286

Component monographs
Part B. Red cell components 289
1. Red Cells 290
Definition and properties 290
Preparation 290
Requirements and quality control 290
Storage and transport 291
Labelling 291
Warnings 292
2. Red Cells, Buffy Coat Removed 293
Definition and properties 293
Preparation 293

Requirements and quality control 294
Storage and transport 294
Labelling 295
Warnings 295
3. Red Cells, in Additive Solution 297
Definition and properties 297
Preparation 297
Requirements and quality control 297
Storage and transport 298
Labelling 298
Warnings 299
4. Red Cells, Buffy Coat Removed, in Additive Solution 300
Definition and properties 300
Preparation 301
Requirements and quality control 301
Storage and transport 302
Labelling 302
Warnings 303
5. Red Cells, Leucocyte-Depleted 304
Definition and properties 304
Preparation 304
Requirements and quality control 304
Storage and transport 305
Labelling 305
Warnings 306
6. Red Cells, Leucocyte-Depleted in Additive Solution 307
Definition and properties 307
Preparation 308
Requirements and quality control 308
Storage and transport 309
Labelling 309
Warnings 310
7. Red Cells, Apheresis 311
Definition and properties 311
Preparation 311

Requirements and quality control 312
Storage and transport 312
Labelling 313
Warnings 314
8. Red Cells, Washed 315
Definition and properties 315
Preparation 315
Requirements and quality control 315
Storage and transport 316
Labelling 317
Warnings 317
9. Red Cells, Cryopreserved 318
Definition and properties 318
Preparation 319
Requirements and quality control 319
Storage and transport 320
Labelling 321
Warnings 322

Component monographs
Part C. Platelet components 325
1. Platelets, Recovered, Single Unit 326
Definition and properties 326
Preparation 326
Requirements and quality control 327
Storage and transport 328
Labelling 328
Warnings 329
2. Platelets, Recovered, Pooled 330
Definition and properties 330
Preparation 330
Requirements and quality control 331
Storage and transport 332
Labelling 332
Warnings 333
3. Platelets, Recovered, Pooled, Leucocyte-Depleted 334

Definition and properties 334
Preparation 334
Requirements and quality control 335
Storage and transport 336
Labelling 337
Warnings 337
4. Platelets, Recovered, Pooled, in Additive Solution 339
Definition and properties 339
Preparation 339
Requirements and quality control 339
Storage and transport 340
Labelling 341
Warnings 342
5. Platelets, Recovered, Pooled, Leucocyte-Depleted, in Additive Solution 343
Definition and properties 343
Preparation 343
Requirements and quality control 344
Storage and transport 345
Labelling 345
Warnings 346
6. Platelets, Pooled, Pathogen-reduced 347
Definition and properties 347
Preparation 348
Requirements and quality control 348
Storage and transport 349
Labelling 349
Warnings 350
7. Platelets, Apheresis 351
Definition and properties 351
Preparation 351
Requirements and quality control 352
Storage and transport 353
Labelling 353
Warnings 354

8. Platelets, Apheresis, Leucocyte-Depleted 355
Definition and properties 355
Preparation 355
Requirements and quality control 356
Storage and transport 357
Labelling 357
Warnings 358
9. Platelets, Apheresis, in Additive Solution 359
Definition and properties 359
Preparation 359
Requirements and quality control 360
Storage and transport 361
Labelling 361
Warnings 362
10. Platelets, Apheresis, Leucocyte-Depleted, in Additive Solution 363
Definition and properties 363
Preparation 363
Requirements and quality control 364
Storage and transport 365
Labelling 365
Warnings 366
11. Platelets, Apheresis, Pathogen-reduced 367
Definition and properties 367
Preparation 368
Requirements and quality control 368
Storage and transport 369
Labelling 370
Warnings 370
12. Platelets, Cryopreserved 371
Definition and properties 371
Preparation 372
Requirements and quality control 372
Storage and transport 372
Labelling 373

Warnings ... 374

Component monographs
Part D. Plasma components ... 377
1. Plasma, Fresh Frozen ... 378
Definition and properties ... 378
Preparation ... 378
Requirements and quality control ... 379
Storage and transport ... 380
Labelling ... 381
Warnings ... 381
2. Plasma, Fresh Frozen, Pathogen Reduced ... 382
Definition and properties ... 382
Preparation ... 383
Requirements and quality control ... 383
Storage and transport ... 384
Labelling ... 385
Warnings ... 385
3. Cryoprecipitate ... 387
Definition and properties ... 387
Preparation ... 387
Requirements and quality control ... 387
Storage and transport ... 389
Labelling ... 390
Warnings ... 390
4. Cryoprecipitate, Pathogen Reduced ... 391
Definition and properties ... 391
Preparation ... 392
Requirements and quality control ... 392
Storage and transport ... 393
Labelling ... 394
Warnings ... 395
5. Plasma, Fresh Frozen, Cryoprecipitate-Depleted ... 396
Definition and properties ... 396
Preparation ... 396
Requirements and quality control ... 396

Storage and transport 396
Labelling 397
Warnings 398

Component monographs
Part E. White cell components 399
1. Granulocytes, Apheresis 400
Definition and properties 400
Preparation 401
Requirements and quality control 401
Storage and transport 402
Labelling 402
Warnings 403
2. Granulocytes, Pooled 404
Definition and properties 404
Preparation 404
Requirements and quality control 405
Storage and transport 406
Labelling 406
Warnings 406

Chapter 6 Standards for blood components for intrauterine, neonatal and infant use 409

Standards for blood components for intrauterine, neonatal and infant use
Part A. Components for intrauterine transfusions 411
1. Red Cells, Leucocyte-Depleted for Intrauterine Transfusion 412
Definition and properties 412
Preparation 412
Requirements and quality control 412
Storage and transport 413
Labelling 413
Warnings 413
2. Platelets, Leucocyte-Depleted for Intrauterine Transfusion 414
Definition and properties 414
Preparation 414

Requirements and quality control 415
Storage and transport 415
Labelling 415
Warnings 416

Standards for blood components for intrauterine, neonatal and infant use
Part B. Components for neonatal exchange transfusion 417
1. Whole Blood, Leucocyte-Depleted for Exchange Transfusion 418
Definition and properties 418
Preparation 418
Requirements and quality control 418
Storage and transport 418
Labelling 418
Warnings 419
2. Whole Blood, Leucocyte-Depleted, Plasma Reduced for Exchange Transfusion 419
Definition and properties 419
Preparation 420
Requirements and quality control 420
Storage and transport 420
Labelling 421
Warnings 421
3. Red Cells, Leucocyte-Depleted, suspended in Fresh Frozen Plasma, for Exchange Transfusion 421
Definition and properties 421
Preparation 422
Requirements and quality control 422
Storage and transport 423
Labelling 423
Warnings 423

Standards of blood components for intrauterine, neonatal and infant use
Part C. Components (small volume) for neonatal and infant transfusion 425

1. Red Cells for Neonatal and Infant Small Volume Transfusion 426
Definition and properties ... 426
Preparation ... 426
Quality control ... 426
Storage and transport ... 426
Labelling ... 427
Warnings ... 427

Chapter 7 Standards for autologous pre-deposit transfusion ... 429
1. Overview ... 429
2. Selection of patients for PAT and blood collection ... 430
Role of the physician in charge of collection ... 430
Information for donors ... 430
Contraindications or deferral criteria ... 430
3. Preparation, storage and distribution of pre-deposit autologous blood components ... 431
Blood typing and microbiological screening ... 431
Preparation ... 431
Labelling ... 431
Storage and handling ... 432
Warnings ... 432

Chapter 8 Standards for immunohaematology ... 433
1. Overview ... 433
2. Selection and validation of reagents and methods ... 433
3. Quality control ... 434
4. Blood group testing of blood donors and donations ... 434
ABO and RhD ... 434
Additional typing ... 435
Irregular antibody testing ... 435
5. Testing of patient samples ... 435
Blood grouping and antibody detection ... 436
Compatibility testing ... 436

Chapter 9 Standards for screening for infectious markers ... 439
1. Selection and validation of infectious marker tests ... 439
2. Mandatory serological screening tests ... 440

3. Additional serological screening tests 441
4. Management of reactive results in serological screening tests 441
5. Nucleic acid screening 442
6. Selective screening of donations 443

Chapter 10 Standards for haemovigilance .445
1. Overview 445
2. Prerequisites for implementation of a haemovigilance network 445
Traceability of blood components 445
Confidentiality of haemovigilance data 446
3. Device defects 446
4. Post-transfusion infection reported to the blood establishment 446

APPENDIX 1.
KEY CRITERIA FOR DONOR ELIGIBILITY 449

APPENDIX 2.
TABLES FOR CALCULATION OF BLOOD VOLUMES 467

APPENDIX 3.
DATA PROCESSING SYSTEMS 483
1. Planning of a system 484
2. Defining the system 484
3. Implementation and validation 485
Functional testing of components 486
Data migration 486
Environmental testing 486
Change control 487
Maintenance of the system 487
Quality assurance 488
4. Electronic signature 489
General requirements 490
Signature manifestations 490
Signature/record linking 490

Controls for identification codes/passwords/biometrics 490

APPENDIX 4.
STATISTICAL PROCESS CONTROL 493
1. **Introduction** 494
2. **Implementation of SPC** 494
3. **Strategy for statistical sampling** 494
   Tolerance of failure 495
   Confidence level 495
4. **Frequency of control sampling** 495
   Example 1. Use of control charts 497
   Example 2. Method of scan statistics 501
   Example 3. Statistical process control for dichotomous outcomes: an approach based upon hypergeometric/binomial distributions 504

APPENDIX 5.
HEALTH ECONOMICS IN BLOOD TRANSFUSION 515
1. **Overview** 516
2. **Investing in quality** 516
3. **Costing analysis** 517
4. **Modelling cost-effectiveness analysis in transfusion** 517
5. **Economic aspects of the clinical use of blood** 518

DEFINITIONS 519

ABBREVIATIONS 531

REFERENCES 535

FOREWORD

Founded in 1949, the Council of Europe is the oldest and largest of all European institutions and currently numbers 47 member states.[1] One of its founding principles is that of increasing co-operation between member states to improve the quality of life for all Europeans.

Within this context of intergovernmental co-operation in the field of health, the Council of Europe has consistently selected ethical problems for study. The most important such ethical issue relates to the non-commercialisation of human substances, i.e. blood, organs and tissues.

With regard to blood transfusion, co-operation among member states started back in the 1950s. From the outset, the activities were inspired by the following guiding principles: promotion of voluntary, non-

1 Albania, Andorra, Armenia, Austria, Azerbaijan, Belgium, Bosnia and Herzegovina, Bulgaria, Croatia, Cyprus, Czech Republic, Denmark, Estonia, Finland, France, Georgia, Germany, Greece, Hungary, Iceland, Ireland, Italy, Latvia, Liechtenstein, Lithuania, Luxembourg, Malta, Republic of Moldova, Monaco, Montenegro, Netherlands, Norway, Poland, Portugal, Romania, Russian Federation, San Marino, Serbia, Slovak Republic, Slovenia, Spain, Sweden, Switzerland, 'the former Yugoslav Republic of Macedonia', Turkey, Ukraine, United Kingdom.

remunerated blood donation, mutual assistance, optimal use of blood and blood products and protection of the donor and the recipient.

The first result of this co-operation was the adoption of the European Agreement on the Exchange of Therapeutic Substances of Human Origin (European Treaty Series, No. 26) in 1958. It was followed by the European Agreement on the exchange of blood grouping reagents (European Treaty Series, No. 39) and of tissue-typing reagents (European Treaty Series, No. 84) in 1962 and 1976 respectively.

Around these three Agreements, the Council of Europe has established a blood transfusion programme, the aim of which is to ensure good quality and safety of blood and blood components.

Since then, the Council of Europe has adopted a number of recommendations covering ethical, social, scientific and training aspects of blood transfusion. Whereas Agreements are binding on the States that ratify them, Recommendations are policy statements to governments proposing a common course of action to be followed.

Major recommendations include Recommendation No. R (88) 4 on the responsibilities of Health Authorities in the field of blood transfusion and Recommendation No. R (95) 15, which contains a technical appendix entitled *Guide to the preparation, use and quality assurance of blood components*.

Work on Recommendation No. R (95) 15 started in 1986, when the Select Committee of Experts on Quality Assurance in Blood Transfusion Services published proposals on quality assurance in blood transfusion services.

Based on these proposals, the Select Committee produced a more comprehensive document entitled *Guide to the preparation, use and quality assurance of blood components* referred to hereafter as the Guide. The immediate success and acceptability of this document was such that the Committee of Ministers adopted it as a technical appendix to what then became Recommendation No. R (95) 15.

The purpose of this Recommendation and its technical appendix is to provide blood transfusion establishments with a set of standards and

principles relating to the preparation, use and quality assurance of blood components. The *Guide* covers all blood components that will be prepared at a blood transfusion establishment and is intended to form the basis for standard operating procedures (SOPs).

The Recommendation does not cover plasma products obtained by fractionation. In respect of plasma-derived products, technical matters are addressed by the European Pharmacopoeia whilst the European Union has a substantial body of legislation regarding pharmaceutical products including plasma-derived medicinal products.

On 27 January 2003 the European Union adopted Directive 2002/98/EC on setting standards of quality and safety for the collection, testing, processing, storage and distribution of human blood and blood components. As regards technical requirements to be set under Article 29 of the said Directive, the European Commission and the Council of Europe work closely together to ensure that these requirements are compatible with this *Guide*.

As of the 15th Edition of the *Guide*, the content has been separated into two sections. The first, entitled Principles, encompasses background information that has to be considered in forming policy decisions as well as educational aspects thus providing the 'why and how'. It also refers to developments that are not yet incorporated into standards, thus providing advance information about technical changes in the field. It was anticipated that in the subsequent editions of the *Guide*, apart from changes to its technical content, the Principles Section would be further expanded. The second section, entitled Standards, contains the matters that are considered to be 'minimum standards' aligning closely to the European Pharmacopoeia and European Commission Directives. It is intended to assist other jurisdictions to transpose these into legislation. The Standards section states 'what must be done'. Whereas blood establishments in EEA member states are required to comply with legislation derived from the European Commission Directives, this *Guide* is intended to facilitate ongoing improvements on the preparation, use and quality

assurance of blood components through education and the provision of non-binding recommendations. The *Guide* therefore provides additional information and guidance on best practices consistent with current scientific knowledge and expert opinion. At any given time, implementation of these recommendations may vary among member states and individual blood transfusion establishments, and alternative procedures, practices and standards may be in place.

Recommendation No. R (95) 15 also states that its technical appendix, the Guide, will be regularly updated to keep it in line with scientific progress. This task was assigned to the European Committee (Partial Agreement) on Blood Transfusion (CD-P-TS), a Steering Committee of the Council of Europe pursuing activities in the field of blood transfusion. The European Directorate for the Quality of Medicines & HealthCare (EDQM)[2] is in charge of the scientific secretariat for these activities.

This is the 19th Edition of the *Guide*. This publication was elaborated by a dedicated *ad hoc* working group of experts nominated by the CD-P-TS and entrusted with the responsibility of updating its text as instructed by the Committee of Ministers in Recommendation No. R (95) 15.

---

2 EDQM is a Directorate of the Council of Europe, created in 1964 on the legal basis of the Convention on the Elaboration of a European Pharmacopoeia, to which 37 member states and the European Union are signatory.
Members: Austria, Belgium, Bosnia and Herzegovina, Bulgaria, Croatia, Cyprus, Czech Republic, Denmark, Estonia, Finland, France, Germany, Greece, Hungary, Iceland, Ireland, Italy, Latvia, Lithuania, Luxembourg, Malta, Montenegro, Netherlands, Norway, Poland, Portugal, Romania, Serbia, Slovak Republic, Slovenia, Spain, Sweden, Switzerland, 'the former Yugoslav Republic of Macedonia', Turkey, Ukraine, United Kingdom, and the European Union.
Observers: Albania, Algeria, Argentina, Armenia, Australia, Azerbaijan, Belarus, Brazil, Canada, China, Georgia, Guinea, India, Israel, Japan, Kazakhstan, Republic of Korea, Madagascar, Malaysia, Republic of Moldova, Morocco, Russian Federation, Senegal, Singapore, South Africa, Syria, Tunisia, the United States of America, the Taiwan Food and Drug Administration and the World Health Organization.

The elaboration of the 19th Edition of the *Guide* included a large public consultation of the draft version by stakeholders. All comments received were considered by the *ad hoc* expert working group in order to produce a final version by amending the text where necessary. The CD-P-TS approved the text of the *Guide* in November 2016 and released it for publication as the 19th Edition.

The 19th Edition of the *Guide* contains an updated version of the Good Practice Guidelines to fully reflect the most recent changes in good manufacturing practices relevant for blood establishments. The Good Practice Guidelines have been jointly developed by the European Directorate for the Quality of Medicines & HealthCare and the Commission of the European Union. This section of the *Guide* describes standards and specifications for the quality system to be implemented by blood establishments. In the European Union, Directive (EU) 2016/1214, published in July 2016, requests member states to ensure that blood establishments comply with the Good Practice Guidelines for their quality system by 15 February 2018.

The elaboration of the text of the 19th Edition of the *Guide* would not have been possible without the outstanding contributions of the *ad hoc* working group. Special thanks should be made to all those experts for their enlightening contributions and to the Chairperson for her dedication. A detailed list showing the composition of this working group is included. Furthermore, all participants in the public consultation and members of the CD-P-TS who have submitted many constructive comments are also warmly acknowledged.

The drafting and the publication of the 19th Edition of the *Guide* was co-ordinated within the EDQM by Dr Guy Rautmann with the assistance of Ms Ines Khraief-Zouari, Ms Nevena Kojic, Ms Mary Quinn and Mr David Crowe.

EUROPEAN COMMITTEE (PARTIAL AGREEMENT) ON BLOOD TRANSFUSION (CD-P-TS)

Composition of the Committee as of 31 October 2016

Chair

FLESLAND Oystein
The Norwegian Knowledge
Centre for the Health Services
PO Box 7004 St Olavs plass
NO – 0130 OSLO

oystein.flesland@helsedir.no

Members

Austria

SCHENNACH Harald
Central Institute for Blood
Transfusion and Immunology
(ZIB)
TILAK – University Clinics –
Regional Hospital
Anichstrasse 35
AT – 6020 INNSBRUCK

harald.schennach@tirol-kliniken.at

Belgium

Nomination pending

Bosnia and Herzegovina

Nomination pending

Bulgaria

MASHAROVA Natalia
National Centre of Transfusion Haematology
112 Bratia Miladinovi St.
BG – 1202 SOFIA

nathalie_54@abv.bg

Croatia

VUK Tomislav
Croatian Institute of Transfusion Medicine
Petrova 3
CRO – 10 000 ZAGREB

tomislav.vuk@hztm.hr

Cyprus

KIOUPI Stala
Cyprus Ministry of Health
Medical and Public Health Services
Giorgio Prodromou 1 and Hilonos 17
CY – 1449 NICOSIA

s.kioupi@cytanet.com.cy

Czech Republic

TUREK Petr
Thomayer Hospital
Videnskà, 800
RTC – 140 59 PRAHA 4

petr.turek@ftn.cz

Denmark

BAGGE HANSEN Morten
Blood Transfusion Centre
Righospitalet
Blegdamsvej, 9
DK – 2100 COPENHAGEN

morten.bagge.hansen@regionh.dk

Estonia

KULLASTE Riin
North Estonia Medical Centre's Blood Centre
2 Adala Street
ZES – 10614 TALLINN

riin.kullaste@regionaalhaigla.ee

Finland

CASTRÉN Johanna
Finnish Red Cross Blood Service
Kivihaantie, 7
FI – 00310 HELSINKI

johanna.castren@bloodservice.fi

France

DELBOSC Arlette
Direction Générale de la Santé, Ministère de la Santé
14 Avenue Duquesne, Bureau PP4
FR – 75007 PARIS

arlette.delbosc@sante.gouv.fr

Germany

STAHL Dorothea
Paul Ehrlich Institute
Paul Ehrlich Strasse, 51-59
DE – 63207 LANGEN

dorothea.stahl@pei.de

STROBEL Johanna (substitute)
Federal Ministry of Health
DE – 53107 BONN

johanna.strobel@bmg.bund.de

Greece

POLITIS Constantina
Ministry of Health, National Blood Centre
Coordinating Haemovigilance Centre, Hellenic CDC
10 Averof Str,
GR – 10433 ATHENS

cpolitis@keelpno.gr

DADIOTIS Loukas (substitute)
General Hospital of Piraeus Tzaneio
GR – 18536 PIRAEUS

aimodosia@tzaneio.gr

Hungary

BAROTI TOTH Klara
Hungarian National Blood Transfusion Service
19-21 Karolina St.
H – 1113 BUDAPEST

barotine.toth.klara@ovsz.hu

Ireland

Nomination pending

Italy

LIUMBRUNO Giancarlo
Italian National Blood Centre
Istituto Superiore di Sanità
Via Giano della Bella No 27
IT – 00162 ROME

giancarlo.liumbruno@iss.it

DE ANGELIS Vincenzo (substitute)
Udine University Hospital
P. le S. Maria della Misericordia, 15
IT – 33100 UDINE

deangelis.vincenzo@aoud.sanita.fvg.it

Latvia

DAUGAVVANAGA Anita
State Blood Donor Center, Riga
Selpils Street 9
LV – 1007 RIGA

anita.daugavvanaga@vadc.gov.lv

Lithuania

NAUJOKAITE Alvyda
Ministry of Health of the
Republic of Lithuania
Vilniaus St., 33
LT – 01506 VILNIUS

alvyda.naujokaite@sam.lt

OGINSKIENE Rasa (substitute)
Ministry of Health
28 Naikupės str.
LT - 93194 KLAIPĖDA

r.oginskiene@kraujodonoryste.lt

Luxembourg

COURRIER Paul
Centre de Transfusion
sanguine de la Croix Rouge
luxembourgeoise
42 boulevard Joseph II
L – 1840 LUXEMBOURG

paul.courrier@croix-rouge.lu

Malta

LASPINA Stefan
Mater Dei Hospital Blood Bank
Pathology Department, Block C,
Level -1
MT – MSD 2090 TAL-QROQQ

stefan.laspina@gov.mt

Montenegro

RASOVIC Gordana
Institute for Blood Transfusion
of Montenegro
Dzona Dzeksona BB
ME – 81000 PODGORICA

gordana.rasovic@ztkcg.me

Netherlands

Nomination pending

Poland

RADZIWON Piotr Marek
Regional Centre for Transfusion
Medicine
Ul.M.Sklodowskiej Curie 23
PL – 15-950 BIALYSTOK

pradziwon@rckik.bialystok.pl

Portugal

SOUSA Ana Paula
Centro de Sangue e da
Transplantação de Lisboa
Parque de Saúde de Lisboa, Av.
do Brasil
PT – 1749-005 LISBOA

ana.paula@ipst.min-saude.pt

CHIN TAD MUON Mário
Centro de Sangue e da
Transplantação de Coimbra
Rua Escola Inês Castro Póvoa
PT – 3040-226 COIMBRA

mario.chin@ipst.min-saude.pt

Romania

DOBROTA Alina Mirella
Regional Blood Transfusion Centre
St. Nicolas lorga, n 85
Constanta County
RO – 900587 CONSTANTA

alina_mirella@yahoo.com

Serbia

VASILJEVIC Nada
Ministry of Health
Direction of Biomedicine
Pasterova 1
SRB – 11000 BELGRADE

vnada12345@gmail.com

Slovak Republic

ROSOCHOVA Jana
Ministerstvo zdravotníctva SR
Limbova 2
SK – 837 52 Bratislava

rosochova@ntssr.sk

Slovenia

ROZMAN Primoz
Blood Transfusion Centre of Slovenia
Slajmerjeva ulica 6
SI – 1000 LJUBLJANA

primoz.rozman@ztm.si

Irena RAZBORSEK (substitute)
Blood Transfusion centre of Slovenia
Slajmerjeva ulica 6
SI – 1000 LJUBLJANA

irena.razborsek@ztm.si

Spain

FERNANDEZ ALVAREZ Carmen
Ministry of Health, Social Services and Equality
Servicio de Hematologia Hospital de Cabuenes
Calle Los Prados nº 395
ES – 33394 GIJON

carmen.fernandez@sespa.princast.es
contactsetscfa@gmail.com

Sweden

NORDA Rut
Klinisk immunologi och transfusionsmedicin
Uppsala University Hospital
Akademiska Sjukhuset, ing 61
SE – 751 85 UPPSALA

rut.norda@akademiska.se

STROM Helena (substitute)
Socialstyrelsen
The National Board of Health and Welfare
SE – 106 30 STOCKHOLM

helena.strom@socialstyrelsen.se

Switzerland

AMSLER Lorenz
Swissmedic
Hallerstrasse 7
CH – 3000 BERN 9

lorenz.amsler@swissmedic.ch

AMAR EL-DUSOUQUI Soraya (substitute)
Croix Rouge Suisse
Service de la Transfusion Sanguine
Laupenstrasse, 37
CP 5510
CH – 3001 BERN

soraya.amar@blutspende.ch

'The former Yugoslav Republic of Macedonia'

VELKOVA Emilija
Institute of Transfusion Medicine
Mother Theresa 17
MAC – 1000 SKOPJE

emavelkova@yahoo.com

Turkey

ERTUGRUL ORUC Nigar
Blood Transfusion Centre
Diskapi Yildirim Beyazit
Training and Research Hospital
Ministry of Health
TR – 06110 ANKARA

nigarertugrul@gmail.com

United Kingdom

MACLENNAN Sheila (Vice Chair)
NHS Blood and Transplant
Leeds Centre
Bridle Path
UK – LEEDS LS15 7TW

sheila.maclennan@nhsbt.nhs.uk

Observers

Albania

DUROVjollca
Boulevard Bajram Curri
AL – 1001 TIRANA

Armenia

DAGHBASHYAN Smbat
Center of Haematology
Ministry of Health
7 H. Nersisyan Str.
AM – 0017 YEREVAN

armhaem@gmail.com

Australia

SMITH Glenn
Therapeutic Goods
Administration Laboratories
Office of Scientific Evaluation
136, Narrabundah Lane
Symonston
PO Box 100
AU – ACT 2609 WODEN

glenn.smith@tga.gov.au

PROSSER Ian
Therapeutic Goods
Administration Laboratories
136 Narrabundah Lane
AUS –2606 SYMONSTON ACT

ian.prosser@tga.gov.au

Canada

AGBANYO Francisca
Centre for Biologics Evaluation
3rd floor, Room 3379 AL 0603C3
1000 Eglantine Driveway
K1A 0KP
CA – OTTAWA, ONTARIO

francisca_agbanyo@hc-sc.gc.ca

Georgia

AVALISHVILI Levan
The Jo Ann Medical Centre
21 Lubliana St.
GE – 0159 TBILISI

levanavali@gmail.com

Moldova

CEBOTARI Svetlana
National Blood Transfusion
Centre
Str. Academi 11
MD – 2028 CHIȘINĂU

cebotaris@mail.ru

Republic of Belarus

POTAPNEV Michael
Belarusian Research and
Production Centre for
Haematology – Tranfusiology
Dolginovski tract, 160
BY – 220053 MINSK

rspk@anitex.by

Republic of Singapore

TEO Diana
Health Sciences Authority
11 Outram Road
SGP – 169078 SINGAPORE

diana_teo@hsa.gov.sg

Russian Federation

EIKHLER Olga
Federal Medico-Biological
Agency of Russia
Volokalamskoye shosse, 30
RU – 123182 MOSCOW

blood@fmbamail.ru

USA

EPSTEIN Jay
U.S. Food and Drug
Administration
Office of Blood Research and
Review
10903 New Hampshire Avenue
USA – SILVER SPRING, MD
20993

jay.epstein@fda.hhs.gov

WILLIAMS Alan (substitute)
U.S. Food and Drug
Administration
Division of Blood Applications
10903 New Hampshire Avenue
USA – SILVER SPRING, MD
20993

alan.williams@fda.hhs.gov

DH-BIO (Bioethics Committee, Council of Europe)

LWOFF Laurence
Head of Bioethics Unit
Human Rights Directorate
Council of Europe
FR – 67075 STRASBOURG

laurence.lwoff@coe.int

European Commission
VAN DER SPIEGEL Stefaan
DG Health & Food Safety (Santé)
Froissart Straat 101, 08/66
BE – 1040 BRUXELLES

stefaan.van-der-spiegel@ec.europa.eu

World Health Organization
Nomination pending

MEMBERS OF THE AD HOC GROUP (GTS)

Composition of the ad hoc group as of 31 October 2016

Chair

NORDA Rut
Klinisk immunologi och transfusionsmedicin
Uppsala University Hospital
Akademiska Sjukhuset, ing 61
S – 751 85 UPPSALA

rut.norda@akademiska.se

Members

BAROTI TOTH Klara
Hungarian National Blood Transfusion Service
19-21 Karolina St.
H – 1113 BUDAPEST

barotine.toth.klara@ovsz.hu

BOGDANOVA Vera
Federal medico-biological Agency, 'ROSPLASMA'
Volokalamskoye shosse, 30
RU – 123182 MOSCOW

bodgdanova@nic-itep.ru

CASTREN Johanna
Finnish Red Cross Blood Service
Kivihaantie 7
FI – 003010 HELSINKI

johanna.castren@bloodservice.fi

CHIN TAD MUON Mário
Centro de Sangue e da
Transplantação de Coimbra
Rua Escola Inês Castro Póvoa
PT – 3040-226 COIMBRA

mario.chin@ipst.min-saude.pt

DE ANGELIS Vincenzo
Udine University Hospital
P. le S. Maria della Misericordia,
15
IT – 33100 UDINE

deangelis.vincenzo@aoud.sanita.fvg.it

DE KORTE Dirk
Sanquin
PO Box 9190
NL – 1066 AD AMSTERDAM

d.dekorte@sanquin.nl

DOBROTA Alina Mirella
Regional Blood Transfusion
Centre
St. Nicolas lorga, n 85
Constanta County
RO – 900587 CONSTANTA

alina_mirella@yahoo.com

ERTUGRUL ORUC Nigar
Blood Transfusion Centre
Diskapi Yildirim Beyazit
Training and Research Hospital
Ministry of Health
TR – 06110 ANKARA

nigarertugrul@gmail.com

FERNANDEZ ALVAREZ
Carmen
Ministry of Health, Social
Services and Equality
Servicio de Hematologia Hospital
de Cabuenes
Calle Los Prados nº 395
ES – 33394 GIJÓN

carmen.fernandez@sespa.princast.es
contactsetscfa@gmail.com

FLANAGAN Peter
New Zealand Blood Service
Private Bag 92071, Victoria Street
West
NZ – 1142 AUCKLAND

peter.flanagan@nzblood.co.nz

FLESLAND Oystein
The Norwegian Knowledge
Centre for the Health Services
PO Box 7000 St Olavs plass
NO – 0130 OSLO

oystein.flesland@helsedir.no

FONTANA Stefano
Blutspendedienst SRK Bern AG
Murtenstrasse 133
CH – 3001 BERN

stefano.fontana@itransfusion.ch

GARRAUD Olivier
Institut National de la
Transfusion Sanguine
6 rue Alexandre-Cabanel
FR – 75739 PARIS

ogarraud@ints.fr

GUDMUNDSSON Sveinn
Blood Bank
Snorrabraut 60
IS – 101 REYKJAVIK

sveinn@landspitali.is

HOPKINS Andrew
MHRA
151 Buckingham Palace Road
Victoria
UK – SW1W 9SZ LONDON

andrew.hopkins@mhra.gsi.gov.uk

ILLOH Orieji
Food and Drug Administration
10903 New Hampshire Avenue
USA – SILVER SPRING, MD
20993-0002

orieji.illoh@fda.hhs.gov

KAMMER Winfried
Paul Ehrlich Institute
Paul Ehrlich Strasse 51-59
DE – 63225 LANGEN

winfried.kammer@pei.de

KLUTER Harald
Institut für Transfusionsmedizin
und Immunologie
Friedrich-Ebert-Strasse 107
DE – 68167 MANNHEIM

harald.klueter@medma.uni-heidelberg.de

LASPINA Stefan
Mater Dei Hospital Blood Bank
Pathology Department, Block C,
Level -1
MT – MSD 2090 TAL-QROQQ

stefan.laspina@gov.mt

MACLENNAN Sheila
NHSBT
Leeds Blood Centre
Bridle Path
UK – LEEDS LS15 7TW

sheila.maclennan@nhsbt.nhs.uk

MEGESSIER Pascal
French National Agency for Medicines and Health Products Safety
143/147, boulevard Anatole France
FR – 93285 SAINT-DENIS CEDEX

pascal.megessier@ansm.sante.fr

NAUJOKAITE Alvyda
Ministry of Health of the Republic of Lithuania
Vilniaus St. 33
LT – 01506 VILNIUS

alvyda.naujokaite@sam.lt

O DONGHAILE Diarmaid
Irish Blood Transfusion Service
National Blood Centre
James's Street
IRL – DUBLIN 8

Diarmaid.ODonghaile@ibts.ie

PINK Joanne
Australian Red Cross Blood Service
44 Musk Avenue
AU – KELVIN GROVE QLD, 4059

jpink@redcrossblood.org.au

POLITIS Constantina
Ministry of Health, National Blood Centre
Coordinating Haemovigilance Centre, Hellenic CDC
10 Averof Str.
GR – 10433 ATHENS

cpolitis@keelpno.gr

RADZIWON Piotr Marek
Regional Centre for Transfusion Medicine
Ul.M. Sklodowskiej Currie 23
PL – 15-950 BIALYSTOK

pradziwon@rckik.bialystok.pl

RAPAILLE André
Croix-Rouge de Belgique
18 rue Bidaut
BE – 4000 LIÈGE

andre.rapaille@croix-rouge.BE

REHACEK Vit
University Hospital Hradec Kralove
Transfusion Department
Sokolksa str. 581
CZ – 500 05 HRADEC KRALOVE

rehacekv@lfhk.cuni.cz

ROSOCHOVA Jana
National Transfusion Service
Ďumbierska 3L
SK - 831 01 BRATISLAVA

rosochova@ntssr.sk

SÄFWENBERG Jan
Uppsala University Hospital
Blood Centre
SE – SE751 85 UPPSALA

jan.safwenberg@akademiska.se

SCHÄRER Christian
Swissmedic
Hallerstrasse 7
CH – 3000 BERN 9

christian.schaerer@swissmedic.ch

SCHENNACH Harald
Central Institute for Blood
Transfusion and Immunology
(ZIB)
TILAK – University Clinics –
Regional Hospital
Anichstrasse 35
AT – 6020 INNSBRUCK

harald.schennach@tirol-kliniken.at

SORENSEN Betina
Aarhus University Hospital
Brendstrupgaardsvej 100, Skejby
DK – 8200 AARHUS N

betina.sorensen@skejby.rm.dk

VASILJEVIC Nada
Ministry of Health
Direction of Biomedicine
Pasterova 1
SRB – 11000 BELGRADE

vnada12345@gmail.com

VUK Tomislav
Croatian Institute for Blood
Transfusion
Petrova 3
HR – 10000 ZAGREB

tomislav.vuk@hztm.hr

Recommendation No. R (95) 15

of the Committee of Ministers to member states

on the preparation, use and quality assurance of blood components

(Adopted by the Committee of Ministers on 12 October 1995 at the 545th meeting of the Ministers' Deputies)

The Committee of Ministers, under the terms of Article 15.*b* of the Statute of the Council of Europe;

Considering that the aim of the Council of Europe is to achieve greater unity between its members and that this aim may be pursued, *inter alia*, by the adoption of common action in the health field;

Recalling its Resolution (78) 29 on harmonisation of legislations of member states relating to removal, grafting and transplantation of human substances;

Recalling also its Recommendations No. R (80) 5 concerning blood products for the treatment of haemophiliacs, No. R (81) 14 on preventing the transmission of infectious diseases in the international transfer of blood, its components and derivatives, No. R (84) 6 on the prevention of the transmission of malaria by blood transfusion,

No. R (85) 12 on the screening of blood donors for the presence of Aids markers, No. (86) 6 on guidelines for the preparation, quality control and use of fresh frozen plasma, No. R (88) 4 on the responsibilities of health authorities in the field of blood transfusion and No. R (93) 4 concerning clinical trials involving the use of components and fractionated products derived from human blood or plasma;

Taking into account the Council Directive 89/381/EEC extending the scope of Directives 65/65/EEC and 75/319/EEC on the approximation of provisions laid down by law, regulation or administrative action relating to proprietary medical products and laying down special provisions for medicinal products derived from human blood or human plasma;

Taking into account Agreement No. 26 on the exchange of therapeutic substances of human origin;

Considering the importance of blood components in modern haemotherapy and the necessity to ensure their safety, efficacy and quality;

Considering that such components are of human origin and that hence specific ethical and technical principles have to be taken into account;

Considering the need for harmonisation of such principles in member states;

Considering that biotechnology does not provide substitutes for most blood products;

Convinced, therefore, of the need to provide health authorities, transfusion services as well as hospital blood banks and clinical users with a set of guidelines for the preparation, use and the quality assurance of blood components;

Aware that the Guide to the preparation, use and quality assurance of blood components published by the Council of Europe has already become the generally accepted European standard and that it is therefore appropriate to give a legal basis to this guide;

Considering that this guide will be regularly updated by the committee of experts of the Council of Europe;

Recommends that the governments of member states take all necessary measures and steps to ensure that the preparation, use and quality control of blood components are carried out in accordance with the guidelines set out in the appendix to this recommendation.

Guide to the preparation, use and quality assurance of blood components

Appendix to Recommendation No. R (95) 15

GOOD PRACTICE GUIDELINES

for standards and specifications for implementing the quality system in blood establishments

Introductory note

Good Practice Guidelines have been prepared through an *ad hoc* co-operation between the European Directorate for the Quality of Medicines & HealthCare of the Council of Europe (EDQM/CoE) and the Commission of the European Union (EU).

These Good Practice Guidelines are included in this 19th Edition of the Guide to the preparation, use and quality assurance of blood components, Appendix to Recommendation No. R (95) 15 of the Committee of Ministers on the preparation, use and quality assurance of blood components.

EU Member States shall ensure, according to Directive 2005/62/EC, that the quality system in place in all blood establishments complies with the standards and specifications set out in the Annex to that Directive.

In order to implement the standards and specifications set out in the Annex to Directive 2005/62/EC, its Article 2, as amended by Directive (EU) 2016/1214, is replaced by the following:

Member States shall ensure that, in order to implement the standards and specifications set out in the Annex to this Directive, there are good practice guidelines available to and used by all blood establishments, in their quality system, good practice guidelines which take fully into account, where relevant for blood establishments, the detailed principles and guidelines of good manufacturing practice, as referred to in the first subparagraph of Article 47 of Directive 2001/83/EC. In doing so, Member States shall take into account the Good Practice Guidelines jointly developed by the Commission and the European Directorate for the Quality of Medicines & HealthCare of the Council of Europe and published by the Council of Europe.

Council of Europe Member States should take the necessary measures and steps to implement the Good Practice Guidelines published in this 19th Edition of the Guide to the preparation, use and quality assurance of blood components. These Good Practice Guidelines provide guidance on how to implement the standards and specifications of quality systems that Member States shall ensure are in place in blood establishments and hospital blood banks.

Good Practice Guidelines for blood establishments and hospital blood banks

1. General principles

1.1. *General requirements*

1.1.1. Each blood establishment must develop and maintain a Quality System that is based on EU Good Manufacturing Practices (GMP) Directive 2003/94/EC and meets the requirements identified in the Directive 2005/62/EC.

1.1.2. For blood and blood components imported from third countries and intended for use or distribution in the EU, there must be a Quality System for blood establishments in the stages preceding importation equivalent to the Quality System provided for in Article 2 of Directive 2005/62/EC.

1.1.3. Quality must be recognised as being the responsibility of all persons involved in the processes of the blood establishment, with management ensuring a systematic approach towards quality and the implementation and maintenance of a Quality System (Directive 2005/62/EC/Annex 1.1.1).

1.1.4. Attainment of this quality objective is the responsibility of executive management. It requires the participation and commitment both of staff in many different departments and at all levels within the organisation and of the organisation's suppliers and distributors. To achieve this quality objective reliably there must be a comprehensively designed and correctly implemented

Quality System incorporating Good Practice and Quality Risk Management.

1.1.5. Each actor in the supply chain should establish, document, and fully implement a comprehensively designed Quality System to deliver Quality Assurance based on the principles of Quality Risk management by incorporating Good Practice and Quality Control.

1.1.6. The basic concepts of Quality Management, Good Practice and Quality Risk Management are interrelated. They are described here in order to emphasise their relationships and fundamental importance to the preparation of blood and blood components.

1.2. Quality system

1.2.1. Quality Management is a wide-ranging concept covering all matters, which individually or collectively influence the quality of blood and blood components. It is the sum total of the organised arrangements made with the objective of ensuring that blood components are of the quality required for their intended use. Quality Management therefore incorporates Good Practice.

1.2.2. The Quality System encompasses quality management, quality assurance, continuous quality improvement, personnel, premises and equipment, documentation, collection, testing and processing, storage, distribution, quality control, blood component recall, and external and internal auditing, contract management, non-conformance and self-inspection (Directive 2005/62/EC/Annex 1.1.2).

1.2.3. The Quality System must ensure that all critical processes are specified in appropriate instructions and are carried out in accordance with the standards and specifications of Good Practice and comply with

appropriate regulations as set out in the chapters on Standards in this *Guide* (which includes the Annex to Directive 2005/62/EC).

1.2.4. The Quality System must be designed to assure the quality and safety of prepared blood and blood components, as well as ensure donor and staff safety and customer service. This strategy requires the development of clear policies, objectives and responsibilities. It also requires implementation by means of quality planning, quality control, quality assurance and quality improvement to ensure the quality and safety of blood and blood components, and to provide customer satisfaction.

1.2.5. Executive management has the ultimate responsibility to ensure that an effective Quality System is in place and resourced adequately, and that roles and responsibilities, are defined, communicated and implemented throughout the organisation. Executive management's leadership and active participation in the Quality System is essential. This leadership should ensure the support and commitment of staff at all levels and sites within the organisation to the Quality System.

1.2.6. Executive management should establish a quality policy that describes the overall intentions and direction of the blood establishment and/or hospital blood bank (hereinafter referred to as 'organisation') related to quality. They should also ensure Quality System management and Good Practice governance through management review to ensure its continuing suitability and effectiveness.

1.2.7. The Quality System should be defined and documented. A Quality Manual or equivalent document should be established and contain a description

of the Quality System (including management responsibilities).

1.2.8. All blood establishments and hospital blood banks must be supported by a quality assurance function (whether internal or related) for fulfilling quality assurance. That function must be involved in all quality-related matters, and must review and approve all appropriate quality-related documents (Directive 2005/62/EC/Annex 1.2.1).

1.2.9. An independent function with responsibility for quality assurance must be established. This quality assurance function will be responsible for the oversight of all quality processes but need not necessarily be responsible for carrying out the activities.

1.2.10. All procedures, premises and equipment that have an influence on the quality and safety of blood and blood components must be validated before introduction and must be re-validated at regular intervals, as determined as a result of these activities (Directive 2005/62/EC/Annex 1.2.2).

1.2.11. A general policy regarding qualification of facilities and equipment as well as validation of processes, automated systems and laboratory tests must be in place. The formal objective of validation is to ensure compliance with the intended use and regulatory requirements.

1.2.12. A formal change control system must be in place to plan, evaluate and document all changes that may affect the quality, traceability, availability or effect of components, or the safety of components, donors or patients. The potential impact of the proposed change must be evaluated, and the degree of revalidation or additional testing, qualification and validation needed must be determined.

1.2.13. A formal system for the handling of deviations and non-conformances must be in place. An appropriate level of root-cause analysis should be applied during the investigation of deviations, suspected product defects, and other problems. This strategy can be determined using Quality Risk Management principles. If the true root cause(s) of the issue cannot be determined, consideration should be given to identifying the most likely root cause(s) and to addressing them. Where human error is suspected or identified as the cause, this should be justified having taken care to ensure that process, procedural or system-based errors or problems have not been overlooked, if present. Appropriate corrective actions and/or preventive actions (CAPAs) should be identified and taken in response to investigations. The effectiveness of such actions should be monitored and assessed in accordance with Quality Risk Management principles.

1.2.14. Management must review the system at regular intervals to verify its effectiveness and introduce corrective measures if deemed necessary (Directive 2005/62/EC/Annex 1.1.3).

1.2.15. There should be periodic management review and monitoring both of its effectiveness, with the involvement of executive management and of the operation of the Quality System to identify opportunities for continual improvement of blood and blood components processes and the system itself.

1.2.16. Product quality reviews should be conducted with the objective of verifying the consistency of the existing process and the appropriateness of current specifications in order to highlight trends and to identify improvements in both component and process.

1.2.17. A product quality review may also be considered as an instrument for surveying the overall quality status of a blood component and its manufacturing processes, including the collection. Such a review should normally be conducted annually and should be documented. It may include:

1.2.17.1. review of starting materials;

1.2.17.2. review of critical in-process controls;

1.2.17.3. review of results of quality control and quality monitoring;

1.2.17.4. review of all changes;

1.2.17.5. review of the qualification status of equipment;

1.2.17.6. review of technical agreements and contracts;

1.2.17.7. review of all significant deviations, non-conformances, and the corrective actions implemented;

1.2.17.8. review of the findings of internal and external audits and inspections, and the corrective actions implemented;

1.2.17.9. review of complaints and recalls;

1.2.17.10. review of donor acceptance criteria;

1.2.17.11. review of donor deferrals;

1.2.17.12. review of look-back cases.

1.3. Good practice

1.3.1. Good Practice is the part of Quality Management that ensures that blood and blood components are produced and controlled consistently to the quality standards appropriate to their intended use. Good Practice is concerned with collection, processing, testing release and storage (hereinafter included in the

generic term 'preparation') and quality control. The basic requirements are:

1.3.1.1. All processes are defined clearly and reviewed systematically in the light of experience and shown to be capable of consistently delivering blood and blood components of the required quality and complying with their specifications. This strategy includes ensuring that:

1.3.1.1.1. critical steps and significant changes to the process are validated;

1.3.1.1.2. all requirements are provided including:

1.3.1.1.2.1. appropriately qualified and trained personnel;

1.3.1.1.2.2. adequate premises and space;

1.3.1.1.2.3. suitable equipment and services;

1.3.1.1.2.4. correct materials, containers and labels;

1.3.1.1.2.5. approved procedures and instructions;

1.3.1.1.2.6. suitable storage and transport;

1.3.1.1.3. instructions and procedures are written in an instructional form in clear and unambiguous language, and are applicable specifically to the facilities provided;

1.3.1.1.4. operators are trained to carry out procedures correctly;

1.3.1.1.5. records are made, manually and/or by recording instruments, during preparation which demonstrate that all the steps required by the defined procedures and instructions were in fact taken and that the quantity and quality of the blood or blood component was as expected;

1.3.1.1.6. any significant deviations are fully recorded and investigated;

1.3.1.1.7. records of preparation (including distribution) that enable the complete history of the blood or blood component to be traced are retained in a comprehensible and accessible form;

1.3.1.1.8. the distribution of the blood and blood components minimises any risk to their quality;

1.3.1.1.9. a system is available to recall any blood or blood component (including those prepared using a batch of critical materials that have been distributed or issued);

1.3.1.1.10. complaints about blood and blood components are examined, the causes of quality defects investigated, and appropriate measures taken in respect of the defective blood components to prevent reoccurrence.

1.3.1.2. Quality Control is the part of Good Practice that is concerned with sampling, specifications and testing, as well as with the organisation, documentation and release procedures which ensure that materials are not released for use in preparation, and blood and blood components are not released for distribution, until their quality has been judged to be satisfactory and that the necessary and relevant tests have been carried out. The basic requirements are:

1.3.1.2.1. adequate facilities, trained personnel and approved procedures are available for sampling, inspecting/testing starting materials, packaging materials, intermediate components, and finished blood and blood components and, if appropriate, for monitoring environmental conditions;

1.3.1.2.2. samples of starting materials, packaging materials, intermediate, and finished blood components are taken by approved personnel and methods;

1.3.1.2.3. test methods are validated;

1.3.1.2.4. records are made, manually and/or by recording instruments, which demonstrate that all the required sampling, inspecting and testing procedures were actually carried out. Any deviations are recorded and investigated fully;

1.3.1.2.5. the finished blood and blood components comply with the specifications and are correctly labelled;

1.3.1.2.6. records are made of the results of inspection, and that testing of materials, intermediate and finished blood and blood components are formally assessed against specifications;

1.3.1.2.7. no blood or blood components are released for distribution that do not comply with the requirements of the relevant authorisations.

1.3.1.3. Rolling quality reviews of all blood and blood components (including export-only blood components) should be conducted with the objective of continuously verifying the: consistency of the existing process; appropriateness of current specifications for both starting materials and finished blood components to highlight any trends and to identify product and process improvements.

1.4. Quality risk management

1.4.1. Quality Risk Management is the part of the Quality System that ensures that the process performance and quality monitoring and review systems are based on risk. Appropriate statistical tools should be used (where appropriate) in the assessment of ongoing process capability.

1.4.2. The Quality System should ensure that processes are in place to ensure the control of outsourced activities and quality of purchased materials. These processes

should incorporate the principles of Quality Risk Management and systematically ensure that:

1.4.2.1. the evaluation of the risk to quality is based on scientific knowledge, experience with the process and, ultimately, is connected to protection of the donor and patient;

1.4.2.2. the level of effort, formality and documentation of the quality risk management process is commensurate with the level of risk.

2. Personnel and organisation

2.1. Personnel must be available in sufficient numbers to carry out the activities related to the collection, testing, processing, storage and distribution of blood and blood components and be trained and assessed to be competent to perform their tasks (Directive 2005/62/EC/Annex 2.1).

2.2. The organisation should have an adequate number of personnel with the necessary qualifications and experience. Management has the ultimate responsibility to determine and provide adequate and appropriate resources (human, financial, materials, facilities and equipment) to implement and maintain the Quality Management System and continually improve its suitability and effectiveness through participation in management review. The responsibilities placed on any one individual should not be so extensive as to present any risk to quality.

2.3. There should be an organisation chart in which the relationships between key personnel are clearly shown in the managerial hierarchy. Key personnel include the following functions and their substitutes:

2.3.1. a 'Responsible Person' following Article 9 of Directive 2002/98/EC;

2.3.2. a processing manager, responsible for all processing activities;

2.3.3. a quality control manager, responsible for all quality control activities;

2.3.4. a quality assurance manager, responsible for ensuring that there are appropriate quality systems and protocols in place for the safe and secure release of all materials, equipment, reagents and blood and blood components;

2.3.5. a physician with the responsibility for ensuring the safety of donors and a physician or pharmacist with responsibility for the safety of the distributed blood components.

2.4. All personnel must have up-to-date job descriptions, which clearly set out their tasks and responsibilities. Responsibility for processing management and quality assurance must be assigned to different individuals, and who function independently (Directive 2005/62/EC/Annex 2.2).

2.5. Personnel in responsible positions should have adequate authority to carry out their responsibilities. Their duties may be delegated to designated deputies of a satisfactory qualification level. There should be no gaps or unexplained overlaps in the responsibilities of those personnel concerned with the application of Good Practice.

2.6. Individual responsibilities should be clearly defined and their correct understanding by individuals should be assessed and recorded. Personnel signature lists should be available.

2.7 All personnel must receive initial and continued training appropriate to their specific tasks. Training records must be maintained. Training programmes must be in place and must include Good Practice (Directive/2005/62/EC/Annex 2.3).

2.8. Training should be provided for all personnel whose duties take them into preparation areas or into laboratories (including the technical, maintenance and cleaning personnel).

2.9. There should be written policies and procedures to describe the approach to training, including a record of training that has taken place, its contents, and its effectiveness.

2.10. The contents of training programmes must be periodically assessed and the competence of personnel evaluated regularly (Directive/2005/62/EC/Annex 2.4).

2.11. Only persons who are authorised by defined procedures and documented as such may be involved in the collection, processing, testing and distribution processes, including quality control and quality assurance.

2.12. There must be written safety and hygiene instructions in place, adapted to the activities to be carried out, and in compliance with Council Directive 89/391/EEC and Directive 2000/54/EC of the European Parliament and of the Council (Directive/2005/62/EC/Annex 2.5).

2.13. Visitors or untrained personnel should, preferably, not be taken into the processing and laboratory areas. If this is unavoidable, they should be given information in advance, particularly about personal hygiene and the prescribed protective clothing. They should be closely supervised.

2.14. It is the organisation's responsibility to provide instructions on hygiene and health conditions that can be of relevance to the quality of blood components (e.g. during collection) and to ensure that staff report relevant health problems. These procedures should be understood and followed in a strict way by all staff members whose duties take them into the processing and laboratory areas. Personnel should be instructed to use the hand-washing facilities.

2.15. Steps should be taken to ensure as far as is practicable that no person affected by an infectious disease or having open lesions on the exposed surface of the body is engaged in the preparation of blood components. Medical examinations should be carried out when necessary to assure fitness for work and personal health. There should be instructions ensuring that health conditions that can be of relevance to the quality of blood and blood components are reported by the personnel.

2.16. There should be a written policy outlining the requirements for wearing of protective garments in the different areas. The requirements should be appropriate to the activities to be carried out.

2.17. Eating, drinking, chewing or smoking, or the storage of food, drink, smoking materials or personal medication in the processing, testing and storage areas should be prohibited. In general, any unhygienic practice within the preparation areas or in any other area where the blood or blood components might be adversely affected should be forbidden.

3. Premises

3.1. General

3.1.1. Premises including mobile sites must be located, constructed, adapted and maintained to suit the activities to be carried out. They must enable work to proceed in a logical sequence so as to minimise the risk of errors, and must allow for effective cleaning and maintenance in order to minimise the risk of contamination (Directive/2005/62/EC/Annex 3.3.1).

3.1.2. Lighting, temperature, humidity and ventilation should be appropriate and such that they do not adversely affect (directly or indirectly) blood components during their processing and storage, or the accurate functioning of equipment.

3.1.3. Premises should be designed and equipped so as to afford protection against the entry of insects or other animals.

3.1.4. Steps should be taken to prevent the entry of unauthorised people. Areas for processing, laboratory, storage, and quality control should not be used as a right of way by personnel who do not work in them.

3.1.5. Facilities should permit ease of maintenance and cleaning. Open drains should be avoided.

3.1.6. Preparation areas should be ventilated effectively, with air-control facilities (including temperature and, if necessary, humidity and filtration) appropriate to the operations undertaken within them and to the external environment.

3.1.7. Preparation areas should be suitably lit, particularly where visual checks are carried out.

3.1.6. Component sampling may be carried out within the processing area provided it does not carry any risk for other components.

3.2. Blood donor area

3.2.1. There must be an area for confidential personal interviews with, and assessment of, individuals to assess their eligibility to donate. This area must be separated from all processing areas (Directive/2005/62/EC/Annex 3.3.2).

3.2.2. Premises must satisfy common-sense requirements for the health and safety of both the staff (including those of mobile teams) and the donors concerned with due regard to relevant legislation or regulations.

3.3. Blood collection area

3.3.1. Blood collection must be carried out in an area intended for the safe withdrawal of blood from donors that is appropriately equipped for the initial treatment of donors experiencing adverse reactions or injuries from events associated with blood donation. This area must be organised in such a way as to ensure the safety of both donors and personnel as well as to avoid errors in the collection procedure (Directive/2005/62/EC/Annex 3.3.3).

3.3.2. Before premises are accepted for mobile donor sessions, their suitability must be assessed against the following criteria:

3.3.2.1. sufficient size to allow proper operation and ensure donor privacy;

3.3.2.2. safety for staff and donors;

3.3.2.3. the presence of ventilation, electrical supply, lighting, toilet and hand-washing facilities;

3.3.2.4. reliable communication, blood storage and transport;

3.3.2.5. guarantee of adequate interim storage.

3.3.3. The arrangement of the collection room and procedures should ensure that blood is collected in a safe and clean environment to minimise the risk of errors and microbial contamination.

3.3.4. Consideration should be given to the arrangement of donor beds and the handling of bags, samples and labels.

3.4. Blood testing and processing areas

3.4.1. There must be a dedicated laboratory area for testing that is separate from the blood-donor and blood-component processing area, with access restricted to authorised personnel, and must be used only for the intended purpose (Directive/2005/62/EC/Annex 3.3.4).

3.4.2. Laboratories should be designed to suit the operations to be carried out in them. Sufficient space should be given to avoid mix-ups and cross-contamination. There should be adequate suitable storage space for samples and records.

3.4.3. Special provisions may be necessary to protect sensitive instruments from vibration, electrical interference, humidity, and extremes of temperature.

3.5. Storage area

3.5.1. Storage areas must provide for appropriately secure and segregated storage of different categories of blood and blood components and materials, including quarantine and released materials as well as units of blood or blood components collected under special criteria (e.g. autologous donation). Access must be restricted

to authorised persons (Directive/2005/62/EC/Annex 3.3.5.1).

3.5.2. Provisions must be in place in the event of equipment failure or power failure in the main storage facility (Directive/2005/62/EC/Annex 3.3.5.2).

3.5.3. Storage facilities should be clean and free from litter, dust and pests (e.g. insects, rodents).

3.5.4. Storage areas should be of sufficient capacity to allow orderly storage of the various categories of materials and blood components including packaging materials, intermediate and finished components, and materials in quarantine, released, rejected, returned or recalled.

3.5.5. Storage areas should be designed or adapted to ensure good storage conditions. In particular, they should be clean and dry and maintained within predefined temperature limits. Where special storage conditions are required (e.g. temperature, humidity) these should be provided, checked and monitored. An alarm system should alert users in a timely manner to any excursion outside predefined limits.

3.5.6. Receiving and dispatch bays should protect materials and products from the weather. Reception areas should be designed and equipped to allow containers of incoming materials to be cleaned where necessary before storage. The reception area should be separate from the storage area.

3.5.7. If quarantine status is ensured by storage in separate areas, these areas must be marked clearly and their access restricted to authorised personnel. Any system replacing the physical quarantine (e.g. computerised system) should provide equivalent security.

3.5.8. Segregated areas should be allocated and identified appropriately for storage of rejected, discarded,

recalled or returned materials, or blood and blood components.

3.5.9. Special attention should be paid to the safe and secure storage of printed packaging materials (including sets of donation identifier labels).

3.6. Ancillary areas

3.6.1. Staff rest and refreshment areas should be separate from other rooms.

3.6.2. Facilities for changing clothes and for washing and toilet purposes should be readily accessible and appropriate for the number of users. Toilets should not directly open to processing, laboratory or storage areas.

3.6.3. Maintenance workshops should, as far as possible, be separated from preparation areas. If parts and tools are stored in processing and laboratory areas, they should be kept in a location reserved for that use.

3.7. Waste disposal area

3.7.1. An area must be designated for the safe disposal of waste, disposable items used during collection, testing and processing and for rejected blood or blood components (Directive/2005/62/EC/Annex 3.6).

4. Equipment and materials

4.1. General requirements

4.1.1. All equipment must be qualified, calibrated and maintained to suit its intended purpose. Operating instructions must be available and appropriate records kept (Directive/2005/62/EC/Annex 4.1).

4.1.2. Equipment must be selected to minimise any hazard to donors, personnel or blood components (Directive/2005/62/EC/Annex 4.2).

4.1.3. All validated processes must use qualified equipment. Qualification results must be documented. Regular maintenance and calibration must be carried out and documented according to established procedures. The maintenance status of each item of equipment must be available.

4.1.4. All critical equipment must have regular, planned maintenance to detect or prevent avoidable errors and keep the equipment in its optimum functional state. The maintenance intervals and actions must be determined for each item of equipment.

4.1.5. New and repaired equipment must meet qualification requirements when installed and must be authorised before use.

4.1.6. All modifications, enhancements or additions to validated systems and equipment must be managed through the change control procedure of the blood establishment. The effect of each change to the system or equipment, as well as its impact on quality and safety, must be determined to identify the extent of revalidation required.

4.1.7. Instructions for use, maintenance, servicing, cleaning and sanitation must be available.

4.1.8. Procedures must be available for each type of equipment that detail the action to be taken if malfunctions or failures occur.

4.1.9. Only reagents and materials from approved suppliers that meet the documented requirements and specifications must be used. Critical materials must be released by a person qualified to perform this task. If relevant,

materials, reagents and equipment must meet the requirements of Council Directive 93/42/EEC for medical devices and Directive 98/79/EC of the European Parliament and of the Council for *in vitro* diagnostic medical devices, or comply with equivalent standards in the case of collection in third countries (Directive/2005/62/EC/Annex 4.3).

4.1.10. Manufacturers of sterile materials (e.g. blood bag systems, anticoagulant solutions) should provide a certificate of release for each batch. The blood establishment should define acceptance criteria for such certificates in writing, and should include at least the name of the material, manufacturer, compliance with relevant requirements (e.g. pharmacopoeias or regulations for medical devices) and confirmation that the materials are sterile and pyrogen-free.

4.1.11. Status of materials (quarantined, released, rejected) should be indicated clearly.

4.1.12. Materials and reagents should be stored under the conditions established by the manufacturer and in an orderly manner that permits segregation by batch and lot as well as stock rotation.

4.1.13. Storage and use of materials should follow the 'first-in first-out' principle (i.e. the material that entered storage first should be used first) taking into account the expiry date of materials.

4.1.14. Inventory records must be retained for a period acceptable to and agreed with the competent authority (Directive/2005/62/EC/Annex 4.4).

4.1.15. Equipment and material inventory records must be kept as a means to build up a history for a processed component to facilitate recalls.

4.1.16. Repair and maintenance operations should not present any hazard to the donor, staff or quality of the blood and blood components.

4.1.17. Equipment should be designed or selected so that it can be thoroughly cleaned (and where necessary decontaminated). This should be performed according to detailed and written procedures. It should be stored only in a clean and dry condition.

4.1.18. Washing/cleaning solutions and equipment should be chosen and used so that they are not sources of contamination.

4.1.19. Equipment should be installed in such a way as to prevent any risk of error or of contamination.

4.1.20. Parts of equipment and materials that come into contact with blood and blood components must not be reactive, additive or absorptive to such an extent that they affect the quality of the component and thus present any hazard.

4.1.21. Balances and measuring equipment of an appropriate range and precision should be available. Equipment for measuring, weighing, recording and control should be calibrated and checked at defined intervals using appropriate methods. Adequate records of such tests should be maintained, including the values obtained prior to any adjustment. Calibration reports should include the accuracy of any testing equipment and traceability to a national standard. The report and/or calibration certificate must be reviewed and signed to show acceptance of the document. Any failed calibrations will require mention of non-conformance to allow investigation of the potential impact.

4.1.22. Defective equipment should be labelled clearly as such and, if possible, removed from preparation areas.

4.2. Data processing systems

4.2.1. If computerised systems are used, software, hardware and back-up procedures must be checked regularly to ensure reliability, be validated before use, and be maintained in a validated state. Hardware and software must be protected against unauthorised use or unauthorised changes. The back-up procedure must prevent loss of or damage to data at expected and unexpected down-times or function failures (Directive/2005/62/EC/Annex 4.5).

4.2.2. Systems must be properly maintained at all times. Documented maintenance plans must be developed and implemented. This strategy must include audits of quality assurance systems.

4.2.3. Changes in computerised systems must be validated; applicable documentation must be revised and relevant personnel trained appropriately before any change is introduced into routine use. Computerised systems must be maintained in a validated state. This must include user-testing to demonstrate that the system is correctly performing all specified functions both at initial installation and after any system modifications.

4.2.4. There must be a hierarchy of permitted user access to enter, amend, read or print data. Methods of preventing unauthorised entry must be in place, such as personal identity codes or passwords that are changed regularly.

4.2.5. All necessary measures must be taken to ensure protection of data. These measures must ensure that safeguards against unauthorised additions, deletions or

modifications of data and transfer of information are in place to resolve data discrepancies, and to prevent unauthorised disclosure of such information.

4.2.6. Computer systems designed to control decisions related to inventories and release of blood components should prevent the release of all blood or blood components considered not acceptable for release. Preventing release of any components from a future donation from a deferred donor should be possible.

4.3. Qualification and validation

4.3.1. General principles

4.3.1.1. Facilities and equipment need to be qualified prior to implementation. Systems, processes and tests should be validated, which involves wider consideration beyond the facilities and equipment used. In this document, however, the term validation is used in a generic sense, encompassing both qualification and validation activities.

4.3.1.2 The principles of qualification and validation are applicable to the collection, preparation, testing, distribution and issuance of blood components. It is a requirement of Good Practice that blood establishments and hospital blood banks control the critical aspects of their operations through the life cycle of the blood components and the associated processes. Any planned changes to the facilities, equipment, utilities and processes should be formally documented and the impact on the quality of blood components should be validated.

4.3.1.3 A quality risk management approach, consisting of a systematic process for the assessment, control, communication and review of risks to quality across the lifecycle of the blood component, should be applied. As

part of a quality risk management system, decisions on the scope and extent of qualification and validation should be based on a justified and documented risk assessment of the facilities, equipment, utilities and processes.

4.3.1.4 Data supporting qualification and/or validation studies which were obtained from sources outside of the blood establishment/hospital blood banks own quality system may be used provided that this approach has been justified and that there is adequate assurance that controls were in place throughout the acquisition of such data.

4.3.2. Organising and planning for validation

4.3.2.1. All qualification and validation activities should be planned and take the life cycle of facilities, equipment, utilities, process and product into consideration.

4.3.2.2. Qualification and validation activities should only be performed by suitably trained personnel who follow approved procedures and report as defined in the blood establishment quality system. There should be appropriate quality oversight over the whole validation life cycle.

4.3.2.3. The key elements of the site qualification and validation programme should be clearly defined and documented in a validation master plan (VMP) or equivalent document.

4.3.2.4. The VMP or equivalent document should define the qualification/validation system and include or reference information on at least the following:

4.3.2.4.1. qualification and validation policy;

4.3.2.4.2. the organisational structure including roles and responsibilities for qualification and validation activities;

4.3.2.4.3. summary of the facilities, equipment, systems, processes on site and their qualification and validation status;

4.3.2.4.4. change control and deviation management for qualification and validation;

4.3.2.4.5. guidance on developing acceptance criteria;

4.3.2.4.6. references to existing documents;

4.3.2.4.7. the qualification and validation strategy, including requalification, where applicable.

4.3.2.5. For large and complex projects, planning takes on added importance and separate validation plans may enhance clarity. These should be linked and traceable.

4.3.2.6. A quality risk management approach should be used for qualification and validation activities. In light of increased knowledge and understanding from any changes during the qualification and validation phase, the risk assessments should be repeated, as required. The way in which risk assessments are used to support qualification and validation activities should be clearly documented.

4.3.2.7 Appropriate checks should be incorporated into qualification and validation work to ensure the integrity of all data obtained.

4.3.3. Documentation including VMP

4.3.3.1 Good documentation practices are important to support knowledge management throughout the product lifecycle. Validation protocols should be prepared which specify how qualification and validation should be performed and which define the critical systems, attributes and parameters and the associated acceptance criteria.

4.3.3.2. All documents generated during qualification and validation should be approved and authorised by appropriate personnel as defined in the quality system.

4.3.3.3. Qualification documents may be combined together, where appropriate, e.g. installation qualification (IQ) and operational qualification (OQ).

4.3.3.4. Any significant changes to the approved protocol during execution, e.g. acceptance criteria, operating parameters etc., should be documented as a deviation and be scientifically justified.

4.3.3.5. The interrelationship between documents in complex validation projects should be clearly defined.

4.3.3.6. Where validation protocols and other documentation are supplied by a third party providing validation services, appropriate personnel at the blood establishment should confirm suitability and compliance with internal procedures before approval. Vendor protocols may be supplemented by additional documentation/ test protocols before use.

4.3.3.7. Results which fail to meet the pre-defined acceptance criteria should be recorded as a deviation and be fully investigated according to local procedures. Any implications for the validation should be discussed in the report.

4.3.3.8. The review and conclusions of the validation should be reported and the results obtained summarised against the acceptance criteria. Any subsequent changes to acceptance criteria should be scientifically justified and a final recommendation made as to the outcome of the validation.

4.3.3.9. A formal release for the next stage in the qualification and validation process should be authorised by the relevant responsible personnel either as part of the

validation report approval or as a separate summary document. Conditional approval to proceed to the next qualification stage can be given where certain acceptance criteria or deviations have not been fully addressed and there is a documented assessment that there is no significant impact on the next activity.

4.3.4. Qualification stages for equipment, facilities, and systems

4.3.4.1. Qualification activities should consider all stages from initial development of the user requirements specification through to the end of use of the equipment, facility or system. The main stages and some suggested criteria (although these depend on individual project circumstances and may be different) which could be included in each stage are indicated below.

4.3.4.2. User requirements specification (URS): the specification for equipment, facilities, utilities or systems should be defined in a URS and/or a functional specification. The essential elements of quality need to be built in at this stage and any Good Practice risks mitigated to an acceptable level. The URS should be a point of reference throughout the validation life cycle.

4.3.4.3. Design Qualification (DQ). The next element of the validation of new facilities, systems or equipment is DQ. This involves demonstration and documentation of the compliance of the design with Good Practice (i.e. the design is suitable for the intended purpose). The requirements of the user requirements specification should be verified during the design qualification.

4.3.4.4. Factory Acceptance Testing (FAT) / Site Acceptance Testing (SAT): equipment, especially if incorporating novel or complex technology, may be evaluated, if applicable, at the vendor prior to delivery. Prior to installation, equipment should be confirmed to comply with

the URS / functional specification at the vendor site, if applicable. Where appropriate and justified, documentation review and some tests could be performed at the FAT or other stages without the need to repeat on site at IQ/OQ if it can be shown that the functionality is not affected by the transport and installation. FAT may be supplemented by the execution of a SAT following the receipt of equipment at the manufacturing site.

4.3.4.5. Installation Qualification (IQ). It should be performed on new or modified facilities, systems and equipment. IQ should include, but is not limited to, the following:

4.3.4.5.1. installations of components, equipment, piping, services and instrumentation, which are checked against up-to-date engineering drawings and specifications;

4.3.4.5.2. verification of the correct installation against pre-defined criteria;

4.3.4.5.3. collection and collation of supplier operating and working instructions and maintenance requirements;

4.3.4.5.4. calibration requirements;

4.3.4.5.5. verification of construction materials.

4.3.4.6. Operational Qualification (OQ). The completion of a successful OQ should allow finalisation of calibration, operating and cleaning procedures, operator training and preventive maintenance requirements. OQ normally follows IQ but depending on the complexity of the equipment, it may be performed as a combined Installation/Operation Qualification (IOQ). OQ should include, but is not limited to, the following:

4.3.4.6.1. tests that have been developed from knowledge of processes, systems and equipment to ensure the system is operating as designed;

4.3.4.6.2. tests to confirm upper and lower operating limits, and/or 'worst case' conditions.

4.3.4.7. Performance Qualification (PQ). Although PQ is described as a separate activity, in some cases it may be appropriate to perform it in conjunction with OQ or Process Validation. PQ should follow successful completion of IQ and OQ. PQ should include, but is not limited to, the following:

4.3.4.7.1. tests, using production materials, qualified substitutes or simulated blood components proven to have equivalent behaviour, under normal and worst case operating conditions. The frequency of sampling used to confirm process control should be justified;

4.3.4.7.2. tests should cover the operating range of the intended process, unless documented evidence from the development phases confirming the operational ranges is available.

4.3.5. Re-qualification

4.3.5.1 Equipment, facilities and systems should be evaluated at an appropriate frequency to confirm that they remain in a state of control.

4.3.5.2 Where requalification is necessary and performed over a specific time period, the period should be justified and the criteria for evaluation defined. Furthermore, the possibility of small changes over time should be assessed.

4.4. Process validation

4.4.1. General

4.4.1.1. The requirements and principles outlined in this section are applicable to the preparation, distribution and issuance of blood components. They cover the initial validation of new processes, subsequent

validation of modified processes or site transfers for maintaining of the validated state (ongoing process verification). It is implicit in this section that a robust product development process is in place to enable successful process validation.

4.4.1.2. Processes should be shown to be robust and ensure consistent blood component quality prior to their distribution and routine clinical use. Processes should undergo a prospective validation programme, wherever possible. Retrospective validation is no longer an acceptable approach.

4.4.1.3. Process validation of new blood components should cover all intended processes and sites of manufacture. A scientific and risk-based validation approach could be justified for new blood components based on extensive process knowledge from the development stage in conjunction with an appropriate ongoing statistical process control. The design assumes that the validation performed is representative for all process or product settings.

4.4.1.4. For validation of processes for preparation of blood components that are transferred from one site to another or within the same site, the number of blood components used for process validation could be reduced based on existing process knowledge, including the content of the previous validation that should be available. The same approach may be used for different blood bag sizes or volumes, if justified.

4.4.1.5. Process validation should establish whether all quality attributes and process parameters, which are considered important for ensuring the validated state and acceptable blood component quality, can be consistently met by the process. A critical quality attributes (CQA) is a physical, chemical, biological or

microbiological property or characteristic that should be within an approved limit, range or distribution to ensure the desired component quality. A critical process parameter (CPP) is a process parameter whose variability has an impact on a critical quality attribute and which therefore should be monitored or controlled to ensure the process produces the desired quality. The basis by which process parameters and quality attributes were identified as being critical or non-critical should be clearly documented, taking into account the results of any risk assessment activities.

4.4.1.6. The facilities, systems and equipment to be used should be qualified before use and analytical testing methods should be validated. Facilities, systems, equipment, utilities and processes should be periodically evaluated to ensure that they are still operating appropriately.

4.4.1.7. For all blood components, process knowledge from development studies or other sources should be accessible to the blood establishment, unless otherwise justified, and be the basis for validation activities.

4.4.1.8. During process validation a variety of personnel may be involved in the preparation of blood components. Blood components should only be prepared by trained personnel in accordance with good practice using approved documentation. It is expected that processing personnel are involved in the preparation of blood components during validation to facilitate understanding of the process.

4.4.1.9. The suppliers of critical materials should be qualified prior to the preparation of blood components during process validation; otherwise a justification based on the application of quality risk management principles should be documented.

4.4.1.10. Where blood components prepared during process validation are released for clinical use, this should be pre-defined. The conditions under which they are produced should fully comply with the requirements of Good Practice, with the validation acceptance criteria and with any continuous process verification criteria (if used).

4.4.2. Concurrent validation

4.4.2.1. In exceptional circumstances and justified on the basis of significant patient benefit, where there is a strong benefit-risk ratio for the patient and with systematic control of each blood component unit for their conformity to regulatory requirements, it may be acceptable to execute the validation protocol concurrently with distribution of the units produced during validations and not to complete a validation programme before routine production. However, the decision to carry out concurrent validation should be documented in the VMP for visibility and approved by authorised personnel.

4.4.2.2. Where a concurrent validation approach has been adopted, there should be sufficient data to support a conclusion that any given blood component meets the defined acceptance criteria. The results and conclusion should be formally documented and available to the Responsible Person prior to release for clinical use.

4.4.3. Prospective validation

4.4.3.1. Using this approach, a number of blood components may be prepared under the proposed new conditions. The number of process runs carried out, the number of samples taken and the number of observations made should be based on quality risk management principles and be sufficient to allow the normal range of variation and trends to be established and to

provide sufficient data for evaluation. Each blood establishment should determine and justify the number of blood component units necessary to demonstrate assurance that the process is capable of consistently delivering quality blood components.

4.4.3.2 Preparation of blood components during the validation phase should reflect the numbers intended to be produced under normal production circumstances.

4.4.3.3 A process validation protocol should be prepared which defines the critical process parameters (CPP), critical quality attributes (CQA) and the associated acceptance criteria which should be based on development data or documented process knowledge.

4.4.3.4 Process validation protocols should include, but are not limited to the following:

4.4.3.4.1. short description of the process;

4.4.3.4.2. functions and responsibilities;

4.4.3.4.3. summary of the CQAs to be investigated;

4.4.3.4.4. summary of CPPs and their associated limits;

4.4.3.4.5. summary of other (non-critical) attributes and parameters which will be investigated or monitored during the validation activity, and the reasons for their inclusion;

4.4.3.4.6. list of the equipment/facilities/personnel to be used (including measuring/monitoring/recording equipment) together with the calibration status;

4.4.3.4.7. list of analytical methods and method validation, as appropriate.

4.4.3.4.8. proposed in-process controls with acceptance criteria and the reason(s) why each in-process control is selected;

4.4.3.4.9. additional testing to be carried out with acceptance criteria;

4.4.3.4.10. sampling plan and the rationale behind it;

4.4.3.4.11. methods for recording and evaluating results;

4.4.3.4.12. process for release and certification of units (if applicable);

4.4.3.4.13. conclusion.

4.4.4. Ongoing process, verification and maintenance of the validated state

4.4.4.1. Ongoing process verification should provide documented evidence, using statistical process control, that the process remains in a state of control during routine manufacture.

4.4.4.2. All critical processes should be constantly monitored and periodically evaluated to confirm that they remain valid. Where no significant changes have been made to the validated status, a review with evidence that the process meets the prescribed requirements may be deemed acceptable in place of a full revalidation.

4.4.4.3. Blood establishments should monitor blood component quality using statistical process control to ensure that a state of control is maintained throughout the blood component lifecycle with the relevant process trends evaluated.

4.4.4.4. The extent and frequency of ongoing process verification should be reviewed periodically. At any point throughout the product life-cycle, it may be appropriate to modify the requirements taking into account the current level of process understanding and process performance.

4.4.4.5. Ongoing process verification should be conducted under an approved protocol or equivalent documents and a corresponding report should be prepared to document the results obtained. Statistical tools should be used, where appropriate, to support any conclusions with regard to the variability and capability of a given process and to ensure a state of control.

4.4.4.6. The following items are essential to maintain a validated state:

4.4.4.6.1. calibration and monitoring;

4.4.4.6.2. preventive maintenance;

4.4.4.6.3. training and competency;

4.4.4.6.4. supplier requalification;

4.4.4.6.5. periodic review;

4.4.4.6.6. performance monitoring;

4.4.4.6.7. system retirement.

4.4.4.7. Maintenance of the validated status of the blood components should be documented in the Product Quality Review. Incremental changes over time should also be considered and the need for any additional actions, e.g. enhanced sampling, should be assessed.

4.4.4.8. Operational change control, document control and quality control procedures support the maintenance of the validated state.

4.5. Validation of test methods

4.5.1. All analytical test methods used in qualification or validation exercises should be validated with an appropriate detection and quantification limit, where necessary, as defined in 11.2.

4.5.2. Where microbial testing of blood components is carried out, the method should be validated to

confirm that the product or residues, e.g. antibiotics, do not interfere with the analysis and influence the recovery of microorganisms.

4.5.3. Where microbial testing of surfaces is carried out, validation should be performed on the test method to confirm that sanitising agents do not influence the recovery of microorganisms.

4.6. Change control

4.6.1. Change control procedures should ensure that sufficient supporting data are generated to demonstrate that the revised process results in a blood component of the desired quality, consistent with the approved specifications. Supporting data, e.g. copies of documents, should be reviewed to confirm that the impact of the change has been demonstrated prior to final approval.

4.6.2. Written procedures should be in place to describe the actions to be taken if a planned change is proposed for a starting material, blood component specification, process, equipment, environment (or site), product range, method of production or testing or any other change that may affect donor safety , blood component quality or reproducibility of the process.

4.6.3. Changes should be authorised and approved by the responsible persons or relevant functional personnel in accordance with the blood establishment's quality system.

4.6.4. Quality risk management should be used to evaluate planned changes to determine the potential impact on blood component quality, the blood establishment's quality systems, documentation, validation, regulatory status, calibration, maintenance and on any other system to avoid unintended consequences and to plan

for any necessary process validation, verification or requalification efforts.

4.6.5. Following implementation, where appropriate, an evaluation of the effectiveness of change should be carried out to confirm that the change has been successful.

4.6.6. Some changes may require notification to, or licence amendment, from a national regulatory authority.

4.7. Control of equipment and materials

4.7.1. General principles

4.7.1.1. Documented systems for purchasing equipment and materials should be available. These should identify the specific requirements for establishing and reviewing contracts for the supply of both equipment and materials.

4.7.1.2. The contracting process should include:

4.7.1.2.1. checks prior to awarding the contract to help ensure suppliers meet the organisation's needs;

4.7.1.2.2. appropriate checks on received goods to confirm they meet specifications;

4.7.1.2.3. the requirement for manufacturers to provide a certificate of analysis for critical material;

4.7.1.2.4. checks to ensure that goods in use continue to meet specifications;

4.7.1.2.5. regular contact with suppliers to help understand and resolve problems;

4.7.1.2.6. performance of regular audits.

4.7.1.3. Assessment of the performance of equipment should occur in the following situations:

4.7.1.3.1. upon commissioning of new equipment, which must include design, installation, operational and performance qualifications, and full validation data from the manufacturer;

4.7.1.3.2. after any relocation, repairs or adjustments that might potentially alter equipment functioning;

4.7.1.3.3. if ever a doubt arises that the equipment is not functioning appropriately.

4.7.1.4. Consideration should be given to the quality, safety and efficacy of any blood components prepared before discovery of the fault adjustment.

4.7.2. Calibration and monitoring of equipment

4.7.2.1. It is necessary to establish a mechanism to ensure the adequacy of the calibration and monitoring programmes, and that qualified personnel are available for their implementation. A calibration and monitoring plan should be used to define the requirements for establishing and implementing a calibration programme that includes the frequency of monitoring.

4.7.2.2. Trending and analyses of calibration and monitoring results should be a continuous process. Intervals of calibration and monitoring should be determined for each item of equipment to achieve and maintain a desired level of accuracy and quality. The calibration and monitoring procedure should be based on a recognised international standard. The calibration status of all equipment that requires calibration should be readily available.

4.7.2.3. To ensure appropriate performance of a system or equipment, a monitoring plan should be developed and implemented. The plan should take into account the criticality of the system or equipment, and should outline monitoring, user-notification and

problem-resolution mechanisms. If an unusual event is observed, personnel should follow the standard response described in the monitoring plan. The standard response should involve notifying affected personnel and, possibly, initiation of a resolution response to the problem and risk assessment of the affected blood components. Depending on the severity of the problem and the criticality of the system or equipment, a back-up plan may need to be implemented to keep the process or system operating.

4.7.2.4. In addition to testing that evaluates the suitability of the implemented changes, sufficient validation should be conducted on the entire system to demonstrate that portions of the system not involved in the change are not adversely impacted.

4.7.2.5. The training programme should be reassessed for any critical change in environment, equipment or processes. Training records (including plans and protocols of training status) must ensure that training needs are identified, planned, delivered and documented appropriately for maintenance of validated systems and equipment.

4.7.2.6. The ability of a supplier to maintain its activities relating to a system or equipment must be re-qualified on a regular basis; notably to anticipate weaknesses in services or to manage changes in the system, equipment or supplier. The periodicity and detail of the re-qualification process depends on the level of risk of using the system or equipment, and should be planned for each supplier.

4.7.2.7. A periodic review process should be established to ensure that documentation for the system or equipment is complete, current and accurate. A report of the review process should be produced. When

deviations or problems are found, actions should be identified, prioritised, planned and implemented.

5. Documentation

5.1. General principles

5.1.1. Good documentation constitutes an essential part of the Quality System and is key to operating in compliance with Good Practice requirements. Various types of documents and media used should be defined fully in the Quality Management System of the organisation.

5.1.2. Documentation may exist in various forms: paper-based, electronic or photographic. The main objective of the system of documentation used must be to establish, control, monitor and record all activities that directly or indirectly impact on all aspects of the quality and safety of blood and blood components as well as any derived medicinal products. The Quality Management System should include sufficient instructional detail to facilitate common understanding of the requirements, in addition to providing for adequate recording of the various processes and evaluation of any observations, so that ongoing application of the requirements may be demonstrated.

5.1.3. There are two primary types of documentation used to manage and record Good Practice compliance: instructions (directions, requirements) and records/reports. Appropriate practices should be applied with respect to the type of document. Suitable controls should be implemented to ensure the accuracy, integrity, availability and legibility of documents. Instruction documents should be free from errors and available in writing. The term 'written' means

recorded or documented on media from which data may be rendered in a readable form for humans.

5.2. Required good practice documentation (by type)

5.2.1. Documents setting out specifications, procedures and records covering each activity undertaken by a blood establishment must be in place and kept up-to-date (Directive/2005/62/EC/Annex 5.1).

5.2.2. Instructions (directions or requirements)

5.2.2.1. Specifications describe in detail the requirements to which the blood and blood components or materials used or obtained during preparation and distribution must conform. They serve as a basis for quality evaluation (specifications set out in the Standards section of *Chapter 5 – Component monographs* contained in the *Guide to the preparation, use and quality assurance of blood components* published by the Council of Europe may be used).

5.2.2.2. Testing instructions detail all the starting materials, equipment and computerised systems (if any) to be used and specify all sampling and testing instructions. If applied, in-process controls should be specified, together with their acceptance criteria.

5.2.2.3. Procedures (otherwise known as Standard Operating Procedures or SOPs) give directions for performing certain operations.

5.2.2.4. Protocols give instructions for performing certain discreet operations, and may record the outcome (e.g. qualification and validation protocols).

5.2.2.5. Technical agreements are agreed between contract givers and acceptors for outsourced activities.

5.2.3. Records/reports

5.2.3.1. Records provide evidence of various actions taken to demonstrate compliance with instructions, e.g. activities, events, investigations and, in the case of processed blood and blood components, a history of each unit (including its distribution). Records include the raw data that is used to generate other records. For electronic records, regulated users should define which data are to be used as raw data. All data on which quality decisions are based should be defined as 'raw data'.

5.2.3.2. Certificates of analysis provide a summary of testing results on samples of reagents, products or materials, together with the evaluation for compliance with a stated specification.

5.2.3.3. Reports document the carrying out of particular exercises, projects or investigations, together with results, conclusions and recommendations.

5.3. Generation and control of documentation

5.3.1. All types of documents should be defined and adhered to. Requirements apply equally to all forms of document media types. Complex systems need to be understood, well documented and validated, and adequate controls should be in place. Many documents (instructions and/or records) may exist in hybrid forms (i.e. some elements are electronic and others are paper-based). Relationships and control measures for master documents, official copies, data handling and records need to be stated for both hybrid and homogeneous systems.

5.3.2. A document control system, defined in a written procedure, must be established for the review, revision history and archiving of documents, including SOPs. Appropriate controls for electronic documents, such

as templates, forms and master documents, should be implemented. Appropriate controls should be in place to ensure the integrity of the record throughout the retention period.

5.3.3. Documents should be designed, prepared, reviewed, and distributed with care. Reproduction of working documents from master documents should not allow errors to be introduced through the reproduction process.

5.3.4. Documents containing instructions should be approved, signed and dated by appropriate and authorised persons. This may also be undertaken electronically. Documents should have unambiguous content and be uniquely identifiable. The effective date should be defined.

5.3.5. Documents containing instructions should be laid out in an orderly fashion and be easy to check. The style and language of documents should fit with their intended use. Standard Operating Procedures, Work Instructions and Methods should be written in an imperative mandatory style.

5.3.6. Documents within the Quality Management System should be regularly reviewed and kept up-to-date.

5.3.7. All significant changes to documents must be acted upon promptly, and must be reviewed, dated and signed by a person authorised to undertake this task (Directive/2005/62/EC/Annex 5.3).

5.3.8. Instructional documents should not be hand-written; although, where documents require the entry of data, sufficient space should be provided for such entries.

5.4. *Good documentation practices*

5.4.1. Records must be legible and may be handwritten, transferred to another medium such as microfilm, or documented in a computerised system (Directive/2005/62/EC/Annex 5.2).

5.4.2. Records should be made or completed at the time each action is taken and in such a way that all significant activities concerning the donation, collection, processing, testing and distribution of blood and blood components are traceable.

5.4.3. The record system must ensure continuous documentation of the procedures performed from the blood donor to the recipient. That is, each significant step must be recorded in a manner that permits a component or procedure to be traced, in either direction, from the first step to final use/disposal.

5.4.4. Any alteration made to the entry on a document should be signed and dated; the alteration should permit reading of the original information. Where appropriate, the reason for the alteration should be recorded.

5.5. *Retention of documents*

5.5.1. It should be clearly defined which record is related to each activity and where this record is located. Secure controls must be in place to ensure the integrity of the record throughout the retention period. These controls must be validated if appropriate.

5.5.2. Specific retention requirements for certain documentation apply.

5.5.2.1. Records must be retained for a period according to local, national or EU requirements, as appropriate.

5.5.2.2. Traceability data (that allow tracing from donor to recipient and vice versa) should be retained for a minimum of 30 years (Directive 2002/98 Article 14.3).

5.5.2.3. Documentation regarding investigations into Serious Adverse Events and Serious Adverse Reactions should be retained for a minimum of 15 years.

5.5.2.4. Quality System documentation and associated records should be retained for a minimum of 10 years.

5.5.2.5. For other types of documentation, the retention period must be defined on the basis of the business activity that the documentation supports. These retention periods should be specified.

5.6. Specifications

5.6.1. There should be appropriately authorised and dated specifications for starting and packaging materials, as well as finished blood and blood components.

5.6.2. Specifications for starting and primary or printed packaging materials should include or provide reference to, if applicable:

5.6.2.1. a description of the materials, including:

5.6.2.1.1. the designated name and the internal code reference;

5.6.2.1.2. the approved suppliers and, if reasonable, the original producer of the material;

5.6.2.1.3 a sample of printed materials;

5.6.2.2. directions for sampling and testing;

5.6.2.3. qualitative and quantitative requirements with acceptance limits;

5.6.2.4 storage conditions and precautions;

5.6.2.5. the maximum period of storage before re-examination.

5.6.3. Specifications for in-process and finished components should be available (specifications set out in the Standards section of Chapter 5 - Component monographs contained in the *Guide to the preparation, use and quality assurance of blood components* published by the Council of Europe may be used). Components must be labelled in accordance with Directive 2002/98/EC.

5.7. Preparation instructions

5.7.1. Approved, written instructions for preparation should exist for each type of component that is produced. These should include:

5.7.1.1. a process flow for each stage in the preparation of the component, including where it is undertaken and any critical equipment used;

5.7.1.2. methods (or reference to the methods) to be used for starting up and maintaining critical equipment (e.g. cleaning, assembly, calibration);

5.7.1.3. the requirement to check that the equipment and work station are clear of previous blood components, documents or materials not required for the planned process, and that equipment is clean and suitable for use;

5.7.1.4. detailed stepwise processing instructions (e.g. checks on materials, pre-treatments, sequence for adding materials, and critical process parameters such as time and temperature);

5.7.1.5. the instructions for any in-process controls with their limits;

5.7.1.6. requirements for storage of the components and any critical materials and consumables;

5.7.1.7. any special precautions to be observed.

5.8. Labelling

At all stages of the preparation, labelling should identify the individual components and their nature clearly.

5.8.1. Requirements for in-process labelling. The label on an intermediate component should always allow the stage of processing to be determined and should always include:

5.8.1.1. the name of the component;

5.8.1.2. the unique numeric or alpha-numeric donation identification;

5.8.1.3. the name of the producing blood establishment.

5.8.2 Preparation record: each unit is considered to be a unique batch, but preparation records should provide sufficient information to build the history and traceability of a prepared component. Usually this information is captured in the computerised systems of the blood establishment. In general, the blood establishment should have access to the following processing records for each unit:

5.8.2.1. the name and unique identifier of the component;

5.8.2.2. the dates and times of commencement of significant intermediate stages and of completion of processing:

5.8.2.3. the identification (initials) of the operator(s) who performed each critical step of the process (including the process controls) and, where appropriate, the name of any person who verified such steps;

5.8.2.4. the batch number of any relevant consumables and/or analytical control number of each consumable;

5.8.2.5. a record of the in-process controls and identity of the person(s) carrying them out, as well as the results obtained;

5.8.2.6. the results of testing undertaken on the donation and/or the component (excluding quality monitoring);

5.8.2.7. notes on any deviation, including details of the procedures with signed authorisation;

5.8.2.8. information on the processing of non-standard components with signed authorisation.

5.9. Procedures and records

5.9.1. Receipt

5.9.1.1. There should be written procedures and records for the receipt of each delivery of materials and reagents that can impact on the quality and safety of blood and blood components. Records of the receipts should include:

5.9.1.1.1. the name of the material on the delivery note and the containers;

5.9.1.1.2. the 'in-house' code (if any) of the material;

5.9.1.1.3. date of receipt;

5.9.1.1.4 the names of the supplier and manufacturer;

5.9.1.1.5. the batch or reference number of the manufacturer;

5.9.1.1.6 the total quantity and number of items received;

5.9.1.1.7. the batch number assigned after receipt (as applicable);

5.9.1.1.8. the name/ID of the person who received the shipment;

5.9.1.1.9. any relevant comments.

5.9.1.2. There should be written procedures for the internal labelling, quarantine and storage of starting materials, packaging materials and other materials, as appropriate.

5.10. Sampling

5.10.1. There should be written procedures for sampling, which include the methods and equipment to be used, the amounts to be taken, and any precautions to be observed to avoid contamination of the material or any deterioration in its quality.

5.10.2. Quality monitoring of blood components should be consistent with the current specifications for in-process and finished components.

5.10.3. There should be written procedures for testing materials and blood components at different stages of processing, describing the methods and equipment to be used. The tests performed should be recorded.

5.11. Other

5.11.1. Written release and rejection procedures should be available.

5.11.2. Records should be maintained of the distribution of blood components to facilitate recall of any unit, if necessary.

5.11.3. There should be written policies, procedures, protocols, reports and the associated records of actions taken or conclusions reached (if appropriate) for the following issues:

5.11.3.1. validation and qualification of processes, equipment and systems;

5.11.3.2. equipment assembly and calibration;

5.11.3.3. maintenance, cleaning and sanitation;

5.11.3.4. personnel matters, including signature lists, training in Good Practice and technical matters, clothing and hygiene, and verification of the effectiveness of training;

5.11.3.5. environmental monitoring;

5.11.3.6. pest control;

5.11.3.7. complaints;

5.11.3.8. recalls;

5.11.3.9. returns;

5.11.3.10. change control;

5.11.3.11. investigations of deviations and non-conformances;

5.11.3.12. audits of compliance with internal quality/Good Practice;

5.11.3.13. summaries of records, where appropriate (e.g. review of the quality of blood components);

5.11.3.14. supplier audits.

5.11.4. Records should be kept for major or critical analytical testing, processing equipment, and areas where blood components have been processed. They should be used to record in chronological order (as appropriate) any use of the area, equipment/method, calibrations, maintenance, cleaning or repair operations (including the dates and identity of people who carried out these operations).

6. Blood collection, testing and processing

6.1. Donor eligibility

6.1.1. Procedures for safe identification of donors, suitability interview, and eligibility assessment must be implemented and maintained. They must take place before each donation and comply with the requirements set out in Annex II and Annex III to Directive 2004/33/EC (Directive/2005/62/EC/Annex 6.1.1).

6.1.2. There must be secure and unique identification, as well as recording of the contact details, of donors.

Robust mechanisms must link donors to each of their donations.

6.1.3. Upon arrival at the blood establishment, donors must provide evidence of their identity. All donors must undergo a systematic screening process to assess their suitability.

6.1.4. Only healthy persons with a good medical history can be accepted as donors of blood or blood components.

6.1.5. The selection process must include assessment of each donor carried out by a suitably qualified individual who has been trained to use accepted guidelines and who works under the direction of a physician. This assessment involves an interview, a questionnaire and further direct questions, if necessary.

6.1.6. The questionnaire must be designed to elicit information relevant to the health and lifestyle of the donor. It must be designed to be understandable by the donor and given to all donors each time they attend. On completion, it must be signed by the donor.

6.1.7. Relevant acceptance/deferral criteria must be in place at the blood establishment to control acceptance and deferral of donors.

6.1.8. The donor interview must be conducted in such a way as to ensure confidentiality (Directive/2005/62/EC/Annex 6.1.2).

6.1.9. The confidential interview must be conducted by specifically trained staff to ask further direct questions to supplement the information in the questionnaire. The person who carries out the assessment must certify that the relevant questions have been asked.

6.1.10. Records of suitability and final assessment of donors must be signed by a qualified healthcare professional (Directive/2005/62/EC/Annex 6.1.3).

6.1.11. Records should be kept for each activity associated with the selection of the donor. The record should reflect the decision to accept the donor by taking into consideration the medical history, history of deferral, donor interview, and results of the physical examination. Rejection of a donor and the reason for deferral should be recorded. A system must be in place to ensure that the donor is prevented from making future donations during a permanent or temporary deferral period (including for the duration of a temporary deferral).

6.1.12. Donors must be instructed to inform the blood establishment if signs or symptoms occur after a donation. This scenario indicates that the donation may have been infectious or that any other information not disclosed during the health screening may render prior donation unsuitable for transfusion.

6.1.13. Procedures must be in place to ensure that any abnormal findings arising from the donor selection process are properly reviewed by a qualified health professional and that appropriate action is taken.

6.2. Collection of blood and blood components

6.2.1. The procedure for blood collection must be designed to ensure that the identity of the donor is verified and recorded securely, and that the link between the donor and blood, blood components and blood samples is established clearly (Directive/2005/62/EC/Annex 6.2.1).

6.2.2. Donor identity must be confirmed before each critical step in the process but, at the very least, before donor selection and venepuncture.

6.2.3. A system of unique donation numbers should be used to identify each donor and the related donation and

all of its associated components, samples and records, as well as to link each one to each of the others.

6.2.4. During or following the donation, all records, blood bags and laboratory samples should be checked for the issued donation number. Donation number labels that have not been used should be discarded using a controlled procedure.

6.2.5. Systems of sterile blood bags used for the collection of blood and blood components and their processing must be CE-marked or comply with equivalent standards if the blood and blood components are collected in third countries. The batch number of the bag must be traceable for each blood component (Directive/2005/62/EC/Annex 6.2.2).

6.2.6. All handling of materials and reagents, such as receipt and quarantine, sampling, storage, labelling, processing, packaging and distribution, should be done in accordance with written procedures or instructions and, if necessary, recorded.

6.2.7. Only reagents and materials from approved suppliers that meet documented requirements and specifications should be used.

6.2.8. Blood collection procedures must minimise the risk of microbial contamination (Directive/2005/62/EC/Annex 6.2.3).

6.2.8.1. Sterile collection and processing systems for blood should be used for blood and blood components. Collection systems should be used in accordance with manufacturer instructions.

6.2.8.2. Before venepuncture, a check should be made to ensure that the collection system to be used is not damaged or contaminated, and that it is appropriate

for the intended collection. Abnormal moisture or discolouration could suggest a defect.

6.2.8.3. Appropriate procedures for hand disinfection and personal hygiene should be in place, and should be performed by personnel before each donation.

6.2.8.4. The skin at the venepuncture site must be free from lesions, including eczema.

6.2.8.5. The venepuncture site must be prepared using a defined and validated disinfection procedure. The antiseptic solution must be allowed to dry completely before venepuncture. The prepared area must not be touched with fingers before needle insertion.

6.2.8.6. The effectiveness of the disinfection procedure must be monitored and corrective action taken where it is indicated to be defective.

6.2.8.7. The expiry date of the disinfectant should be checked. The date of manufacture and the date of opening of in-house disinfectants should be stated on their labels.

6.2.8.8. The blood container must be checked after donation for any defect. The integral blood bag collection tubing should be sealed off at the end as close as possible to the blood bag.

6.2.8.9. Standard Operating Procedures should be in place describing the actions to be taken following an unsuccessful donation. These should specify how to handle already-labelled material and the circumstances under which a repeat venepuncture might be possible.

6.2.9. Laboratory samples must be taken at the time of donation and be appropriately stored prior to testing (Directive/2005/62/EC/Annex 6.2.4).

6.2.10. The procedure used for the labelling of records, blood bags, and laboratory samples with donation numbers

must be designed to avoid any risk of identification error and mix-up (Directive/2005/62/EC/Annex 6.2.5).

6.2.11. After blood collection, blood bags must be handled in a way that maintains the quality of the blood and at a storage temperature and transport temperature appropriate to the requirements for further processing (Directive/2005/62/EC/Annex 6.2.6).

6.2.12. Blood and blood components should be placed in controlled and validated conditions as soon as possible after venepuncture. Donations and samples should be transported to the processing site in accordance with procedures that ensure a constant approved temperature and secure confinement. There should be validation data to demonstrate that the method of transport maintains the blood within the specified temperature range throughout the period of transportation. Alternatively, portable temperature loggers may be used to record the temperature during transportation of blood to the processing site.

6.2.13. If a deviation occurs, it should be approved in writing by a competent person.

6.2.14. Where the blood is not transported by the processing establishment itself, the responsibilities of the transport company should be clearly defined and periodic audits should be conducted to ensure compliance.

6.2.15. There must be a system in place to ensure that each donation can be linked to the collection and processing system into which it was collected and/or processed (Directive 2005/62/EC/Annex 6.2.7).

6.3. Laboratory testing

6.3.1. All blood donations should be tested to ensure that they meet specifications and to ensure a high level of safety to the recipient.

6.3.2. All laboratory testing procedures must be validated before use (Directive 2005/62/EC/Annex 6.3.1).

6.3.3. In addition to the validation of the test system by the manufacturer, an on-site validation of the test system in the laboratory is required prior to its use in routine testing. This validation should demonstrate that:

6.3.3.1. the performance specifications of the system established by the kit manufacturer are met by the laboratory;

6.3.3.2. laboratory personnel are thoroughly instructed, trained and competent to operate the test system.

6.3.4. All donation testing activities, handling of donor specimens, sampling, analysis and data processing should be undertaken independently of diagnostic testing of patients.

6.3.5. Each step of the handling and processing of samples should be described, as should the conditions of pre-analytical treatment of specimens (e.g. centrifugation), storage and transportation (duration, temperature, type of container, storage after testing).

6.3.6. Upon receipt of samples at the laboratory, positive identification of the samples received against those expected should be carried out.

6.3.7. There must be data confirming the suitability of any laboratory reagents used in testing of donor samples and blood-component samples (Directive 2005/62/EC/Annex 6.3.4).

6.3.8. Testing of blood components should be carried out in accordance with the recommendations of the manufacturer of reagents and test kits (unless an alternative method has been validated before their use) before release of the blood component.

6.3.9. Pre-acceptance testing must be performed on samples before purchasing batches of commercial reagents. Prospective purchasers must require potential suppliers to provide them with full validation data for all lots of reagents. Each lot of reagent must be qualified by the purchaser to demonstrate suitability for its intended purpose within the system used for testing.

6.3.10. There must be a reliable process in place for transcribing, collating and interpreting results.

6.3.11. The quality of the laboratory testing must be assessed regularly by participation in a formal system of proficiency testing, such as an external quality-assurance programme (Directive/2005/62/EC/Annex 6.3.5).

6.4. Testing for infectious markers

6.4.1. Testing of donations for infectious agents is a key factor in ensuring that the risk of disease transmission is minimised and that blood components are suitable for their intended purpose.

6.4.2. Each donation must be tested in conformity with the requirements laid down in Annex IV to Directive 2002/98/EC (Directive 2005/62/EC/Annex 6.3.2).

6.4.3. Additional testing for other agents or markers may be required, taking into account the epidemiological situation in any given region or country.

6.4.4. Serological testing should be performed on samples transferred directly into the analyser from the original sample tube. Secondary aliquot samples may be used for NAT testing of mini-pools of individual samples.

6.4.5. If NAT testing is performed by assembling various samples in mini-pools, a thoroughly validated system of labelling/identification of samples, a validated strategy and pooling process, and a validated algorithm to

reassign pool results to individual donations should be in place.

6.4.6. There must be clearly defined procedures to resolve discrepant results. Blood and blood components that have a repeatedly reactive result in a serological screening test for infection with the viruses mentioned in Annex IV to Directive 2002/98/EC must be excluded from therapeutic use and must be stored separately in a dedicated environment. Appropriate confirmatory testing must take place. In the case of confirmed positive results, appropriate donor management must take place, including the provision of information to the donor and follow-up procedures (Directive 2005/62/EC/Annex 6.3.3).

6.4.7. Screening algorithms should be defined precisely in writing (i.e. Standard Operating Procedures) to deal with initially reactive specimens, and to resolve discrepancies in results after retesting.

6.5. Blood group serological testing of donors and donations

6.5.1. Blood group serology testing must include procedures for testing specific groups of donors (e.g. first-time donors, donors with a history of transfusion) (Directive/2005/62/EC/Annex 6.3.6).

6.5.2. Each donation should be tested for ABO and RhD blood groups and at least all first-time donors should be tested for clinically significant irregular red-cell antibodies.

6.5.3. ABO and RhD blood groups should be verified on each subsequent donation.

6.5.4. Comparison should be made with the historically determined blood group. If a discrepancy is found, the applicable blood components should not be

released until the discrepancy has unequivocally been resolved.

6.5.5. Donors with a history of transfusions or pregnancy since their last donation should be tested for clinically significant irregular red-cell antibodies. If clinically significant red-cell antibodies are detected and, if applicable, the blood or blood component should be labelled accordingly.

6.5.6. Only test reagents that have been licensed or evaluated and considered to be suitable by a responsible National Health Authority/Competent Authority must be used. In the EU, these reagents are considered as *in vitro* diagnostic devices and must be CE-marked.

6.5.7. EU Directive 98/79/EC classifies ABO, Rh (C, c, D, E, e) anti-Kell reagents in list A of Annex II. The manufacturer of such reagents must have a full Quality System certified by an authorised body, and must submit an application containing all the control results for each lot.

6.5.8. Quality-control procedures must be implemented for the equipment, reagents and techniques used for ABO and RhD blood grouping and phenotyping as well as detection and identification of allo-antibodies. The frequency of the control is dependent on the method used.

6.6. Processing and validation

6.6.1. All equipment and technical devices must be used in accordance with validated procedures (Directive/2005/62/EC/Annex 6.4.1).

6.6.2. The processing of blood components must be carried out using appropriate and validated procedures, including measures to avoid the risk of contamination

and microbial growth in the prepared blood components (Directive/2005/62/EC/Annex 6.4.2).

6.6.3. The use of closed systems is strongly recommended for all steps in component processing. Open systems may exceptionally be necessary due to local constraints and should be undertaken in an environment specifically designed to minimise the risk of bacterial contamination. When open systems are used, careful attention should be given to the use of aseptic procedures.

6.6.4. Validation of freezing processes should consider worst-case scenarios that take into account minimum and maximum loads and positions in the freezer.

6.6.5. Sterile connecting devices must be used in accordance with a validated procedure. When validated, connections made using sterile connecting devices are regarded as closed system processing. The resulting weld must be checked for satisfactory alignment and its integrity must be confirmed.

6.7. Labelling

6.7.1. At all stages, all containers must be labelled with relevant information on their identity. In the absence of a validated computerised system for status control, the labelling must clearly distinguish released from non-released units of blood and blood components (Directive 2005/62/EC/Annex 6.5.1).

6.7.2 Type of label to be used, as well as the labelling methodology, should be defined and established in written Standard Operating Procedures.

6.7.3. Labels applied to containers, equipment or premises should be clear, unambiguous and in the agreed format of the blood establishment.

6.7.4. Labelling system for collected blood, intermediate and finished blood components, and samples must unmistakably identify the type of content, and comply with the labelling and traceability requirements referred to in Article 14 of Directive 2002/98/EC and Directive 2005/61/EC. The label for a final blood component must comply with the requirements of Annex III to Directive 2002/98/EC (Directive 2005/62/EC/Annex 6.5.2).

6.7.5. Blood establishments responsible for the preparation of blood components must provide clinical users of blood components with information on their use, composition, and any special conditions that do not appear on the component label.

6.7.6. For autologous blood and blood components, the label must also comply with Article 7 of Directive 2004/33/EC and the additional requirements for autologous donations specified in Annex IV to that Directive (Directive 2005/62/EC/Annex 6.5.3).

6.8. Release of blood and blood components

6.8.1. There must be a safe and secure system to prevent any single blood sample and blood component from being released before all mandatory requirements set out in Directive 2005/62/EC have been fulfilled. Each blood establishment must be able to demonstrate that each blood or blood component has been formally released by an authorised person. Records must demonstrate that before a blood component has been released, all current declaration forms, relevant medical records, and test results have met all acceptance criteria (Directive 2005/62/EC/Annex 6.6.1).

6.8.2. There should be Standard Operating Procedures that detail the actions and criteria that determine whether

the blood or blood component can be released. The release criteria and specifications of blood components should be defined, validated, documented and approved.

6.8.3. There should be a defined procedure for exceptional release of non-standard blood and blood components under a planned non-conformance system. The decision to allow such release should be documented clearly and traceability should be ensured.

6.8.4. Before release, blood and blood components must be kept administratively and physically segregated from released blood and blood components. In the absence of a validated computerised system for status control, the label of a unit of blood or blood component must identify the release status in accordance with point 6.5.1 stated above (Directive 2005/62/EC/Annex 6.5.1 and 6.6.2).

6.8.5. There should be a system of administrative and physical quarantine for blood and blood components to ensure that components cannot be released until all mandatory requirements have been met.

6.8.6. In the event that the final component fails to be released due to a confirmed positive test result for infection for an agent mentioned in Annex IV of Directive 2002/98/EC, a check must be made to ensure that other components from the same donation and components prepared from previous donations given by the donor have been identified. An immediate update must be made to the donor record (Directive 2005/62/EC Annex 6.3.2, 6.3.3 and 6.6.3).

6.8.7. In the event that a final component fails release due to a potential impact on patient safety, the donor record must be immediately updated to ensure, where

appropriate, that the donor(s) cannot make a further donation.

7. Storage and distribution

7.1. The Quality System of the blood establishment must ensure that, for blood and blood components intended for the manufacture of medicinal products, the requirements for storage and distribution must comply with Directive 2003/94/EC (Directive 2005/62/EC/Annex 7.1).

7.2. Procedures for storage and distribution must be validated to ensure the quality of blood and blood components during the entire storage period, and to exclude mix-ups of blood components. All transportation and storage actions, including receipt and distribution, must be defined by written procedures and specifications (Directive 2005/62/EC/Annex 7.2).

7.3. Storage conditions must be controlled, monitored and checked. Appropriate alarms must be present and checked regularly; all checks must be recorded. Appropriate actions on alarms must be defined.

7.4. There should be a system to ensure stock rotation involving regular and frequent checks that the system is operating correctly. Blood and blood components beyond their expiry date or shelf-life should be separated from usable stock.

7.5. Before distribution, blood components must be visually inspected.

7.6. Autologous blood and blood components, as well as blood components collected and prepared for specific purposes, must be stored separately (Directive 2005/62/EC/Annex 7.3).

7.7. Appropriate records of inventory and distribution must be kept (Directive 2005/62/EC/Annex 7.4).

7.8. Records should be kept of the distribution of blood components between blood establishments, blood establishments and hospital blood banks and between hospital blood banks. These records should show the date of supply, unique component identifier and name of the blood component, the quantity received or supplied, name and address of the supplier or consignee.

7.9. Packaging must maintain the integrity and storage temperature of blood and blood components during distribution and transportation (Directive 2005/62/EC/Annex 7.5).

7.10 Verification of transportation

7.10.1 Blood components should be transported in accordance with the defined conditions.

7.10.2 It is recognised that verification of transportation may be challenging due to the variable factors involved; however, transportation routes should be clearly defined. Seasonal and other variations should also be considered during verification of transport.

7.10.3 A risk assessment should be performed to consider the impact of variables in the transportation process other than those conditions which are continuously controlled or monitored, e.g. delays during transportation, failure of cooling and/or monitoring devices, blood component susceptibility and any other relevant factors.

7.10.4 Due to the variable conditions expected during transportation, continuous monitoring and recording of any critical environmental conditions to which the blood component may be subjected should be performed, unless otherwise justified.

7.11. Return of blood and blood components into inventories for subsequent re-issue must be allowed only if all requirements and procedures relating to quality as laid down by the blood establishment to ensure the integrity of blood components are fulfilled (Directive 2005/62/EC/Annex 7.6).

7.12. Blood components must not be returned to the blood establishment for subsequent distribution unless there is a procedure for the return of blood components that is regulated by a contract, and if there is, documented evidence for each returned blood component that the agreed storage conditions have been met. Before subsequent distribution, records must identify that the blood component has been inspected before reissue.

8. Outsourced activities management

8.1. General principles

8.1.1. Tasks that are performed externally must be defined in a specific written contract (Directive 2005/62/EC/Annex 8).

8.1.2. Outsourced activities that may impact on the quality, safety or efficacy of the blood components should be correctly defined, agreed and controlled in order to avoid misunderstandings which could result in a blood component or work of unsatisfactory quality. There should be a written contract covering these activities, the products or operations to which they are related, and any technical arrangements made in connection with it.

8.1.3. All outsourced arrangements for blood collection, processing and testing, including any proposed changes, should be made in accordance with a written contract, with reference to the specification for the blood or blood component(s) concerned.

8.1.4. The responsibilities of each party should be documented to ensure that Good Practice principles are maintained.

8.1.5. The contract giver is the establishment or institution that subcontracts particular work or services to a different institution and is responsible for setting up a contract defining the duties and responsibilities of each side.

8.1.6. The contract acceptor is the establishment or institution that performs particular work or services under a contract for a different institution.

8.2. The contract giver

8.2.1. The contract giver is responsible for assessing the competence of the contract acceptor to successfully carry out the work being outsourced and for ensuring, by means of the contract, that the principles and guidelines of Good Practice are followed.

8.2.2. The contract giver should provide the contract acceptor with all the information necessary to carry out the contracted operations correctly and in accordance with the specification and any other legal requirements. The contract giver should ensure that the contract acceptor is fully aware of any problems associated with the materials, samples or the contracted operations that might pose a hazard to the premises, equipment, personnel, other materials or other blood components of the contract acceptor.

8.2.3. The contract giver should ensure that all blood and blood components, analytical results and materials delivered by the contract acceptor comply with their specifications and that they have been released under a Quality System approved by the Responsible Person or other authorised person.

8.3. The contract acceptor

8.3.1. The contract acceptor should have adequate premises, equipment, knowledge, experience and competent personnel to satisfactorily carry out the work requested by the contract giver.

8.3.2. The contract acceptor should ensure that all products, materials or test results delivered by the contract giver are suitable for their intended purpose.

8.3.3. The contract acceptor should not pass to a third party any of the work entrusted under the contract without the contract giver's prior evaluation and approval of the arrangements. Arrangements made between the contract acceptor and any third party should ensure that the relevant blood collection, processing and testing information is made available in the same way as between the original contract giver and contract acceptor.

8.3.4. The contract acceptor should refrain from any activity that may adversely affect the quality of the blood and blood components prepared and/or analysed for the contract giver.

8.4. The contract

8.4.1. A contract should be drawn up between the contract giver and the contract acceptor that specifies their respective responsibilities relating to the contracted operations. All arrangements for blood collection, processing and testing should be in compliance with the requirements of Good Practice and regulatory requirements and agreed by both parties.

8.4.2. The contract should specify the procedure, including the necessary requirements to be provided by the contract acceptor, by which the Responsible Person or other authorised person releasing the blood and blood

components for sale or supply can ensure that each component has been prepared and/or distributed in compliance with the requirements of Good Practice and regulatory requirements.

8.4.3. The contract should clearly describe who is responsible for purchasing materials, testing and releasing materials, undertaking blood collection, and for processing and testing (including in-process controls). In the case of subcontracted analyses, the contract should state the arrangements for the collection of samples and the contract acceptor should understand that they may be subject to inspections by the Competent Authorities.

8.4.4. Preparation and distribution records, including reference samples if relevant, should be kept by, or be available to, the contract giver. Any records relevant to assessment of the quality of the blood or a blood component in the event of complaints or a suspected defect should be accessible and specified in the defect/recall procedures of the contract giver.

8.4.5. The contract should permit the contract giver to audit the facilities of the contract acceptor.

9. Non-conformance and recall

9.1. Deviations

9.1.1. Blood components deviating from required standards set out in Annex V to Directive 2004/33/EC shall be released for transfusion only in exceptional circumstances and with the recorded agreement of the prescribing physician and the blood establishment physician (Directive 2005/62/EC/Annex 9.1).

9.1.2. For components not listed in Annex V to Directive 2004/33/EC, quality and safety standards set out

in the Standards section of *Chapter 5 – Component monographs* contained in the *Guide to the preparation, use and quality assurance of blood components* published by the Council of Europe may be used to meet the intent of 9.1.1 above.

9.1.3. There should be a defined procedure for the release of non-standard blood and blood components under a planned non-conformance system. The decision for such release should be clearly documented and authorised by a designated person and traceability should be ensured.

9.1.4. There should be systems in place to ensure that deviations, adverse events, adverse reactions and non-conformances are documented, carefully investigated for causative factors of any defect and, where necessary, followed up by the implementation of corrective actions to prevent recurrence.

9.1.5. The corrective and preventive actions (CAPAs) system should ensure that existing component nonconformity or quality problems are corrected and that recurrence of the problem is prevented.

9.1.6. Deviations from established procedures should be avoided as much as possible and should be documented and explained. Any errors, accidents or significant deviations that may affect the quality or safety of blood and blood components should be fully recorded and investigated in order to identify systematic problems that require corrective action. Appropriate corrective and preventive actions should be defined and implemented.

9.1.7. Investigations relating to serious deficiencies, significant deviations and serious component defects should include an assessment of component impact, including a review and evaluation of relevant operational

documentation and an assessment of deviations from specified procedures.

9.1.8. There should be procedures for notifying responsible management in a timely manner of deficiencies, deviations or non-compliance with regulatory commitments (e.g. in submissions and responses to regulatory inspections), component or product defects, or testing errors and related actions (e.g. quality-related complaints, recalls, regulatory actions, etc.).

9.1.9. Executive management and the Responsible Person should be notified in a timely manner of serious deficiencies, significant deviations and serious component or product defects and adequate resource should be made available for their timely resolution.

9.1.10. A regular review of all significant deviations or non-conformances should be conducted, including their related investigations, to verify the effectiveness of the corrective and preventive actions taken.

9.2. Complaints

9.2.1. All complaints and other information, including serious adverse reactions and serious adverse events that may suggest that defective blood components have been issued, must be documented, carefully investigated for causative factors of the defect and, where necessary, followed up by recall and the implementation of corrective actions to prevent recurrence. Procedures must be in place to ensure that the Competent Authorities are notified, as appropriate, of serious adverse reactions or serious adverse events in accordance with regulatory requirements (Directive 2005/62/EC/Annex 9.2).

9.2.2. A person should be designated as responsible for handling complaints and deciding the measures to be

taken. This person should have sufficient support staff. If this person is not the Responsible Person, the latter should be made aware of any complaint, investigation or recall.

9.2.3. If a blood or blood component defect or testing error is discovered or suspected, consideration should be given to checking related blood and blood components in order to determine whether they are also affected.

9.2.4. All the decisions and measures taken as a result of a complaint should be recorded. Complaint records should be reviewed regularly for any indication of specific or recurring problems requiring attention and the possible recall of distributed blood and blood components.

9.2.5. The Competent Authorities should be informed in cases of complaints resulting from possible faulty processing, component deterioration or any other serious quality problems, including the detection of counterfeiting.

9.3. Recall

9.3.1. There must be personnel authorised within the blood establishment to assess the need for blood and blood component recalls and to initiate and co-ordinate the necessary actions (Directive 2005/62/EC/Annex 9.3.1).

9.3.2. An effective recall procedure must be in place, including a description of the responsibilities and actions to be taken. This must include notification of the Competent Authority (Directive 2005/62/EC/Annex 9.3.2).

9.3.3. Actions must be taken within pre-defined periods of time and must include tracing all relevant blood components and, where applicable, must include trace-back. The purpose of the investigation is to identify

any donor who might have contributed to causing the transfusion reaction and to retrieve available blood components from that donor, as well as to notify consignees and recipients of components collected from the same donor in the event that they might have been put at risk (Directive 2005/62/EC/Annex 9.3.3).

9.3.4. Recall operations should be capable of being initiated promptly and at any time. In certain cases recall operations may need to be initiated to protect public health prior to establishing the root cause(s) and full extent of the quality defect.

9.3.5. The persons authorised to initiate and co-ordinate the recall actions should normally be independent of the commercial management within the organisation. If they do not include the executive management and the Responsible Person (blood establishment), the latter should be made aware of any recall operation.

9.3.6 Recalled blood components or products should be identified and stored separately in a secure area while awaiting a decision on their fate.

9.3.7. The progress of the recall process should be recorded and a final report issued, including reconciliation of the delivered and recovered quantities of the blood and blood components or products.

9.3.8. The effectiveness of the arrangements for recalls should be regularly evaluated.

9.4. Deviation management and corrective and preventive actions

9.4.1. A system to ensure corrective and preventive actions for blood component nonconformity and quality problems must be in place (Directive 2005/62/EC/Annex 9.4.1).

9.4.2. Data must be routinely analysed to identify quality problems that may require corrective action or to identify unfavourable trends that may require preventive action (Directive 2005/62/EC/Annex 9.4.2).

9.4.3. All errors and accidents must be documented and investigated in order to identify problems for correction (Directive 2005/62/EC/Annex 9.4.3).

9.4.4. Deviations with the potential to affect quality should be investigated, and the investigation and its conclusions should be documented including all the original details. The validity and extent of all reported quality defects should be assessed in accordance with Quality Risk Management principles in order to support decisions regarding the degree of investigation and action taken. Where appropriate, corrective actions should be taken prior to distribution of blood and blood components or reporting of a test result. The potential impact of the source of the deviation on other components or results should also be considered and preventive action should be taken to eliminate the root cause of the deviation and thereby avoid recurrences.

9.4.5. Investigations should include a review of previous reports or any other relevant information for any indication of specific or recurring problems requiring attention and possibly further regulatory action. Processes and relevant data should be monitored with a view to taking preventive action to avoid potential deviations occurring in the future. Where appropriate, statistical or other tools should be used to assess and monitor process capabilities. As comprehensive information on the nature and extent of the quality defect may not always be available at the early stages of an investigation, the decision-making processes should still ensure that appropriate risk-reducing

actions are taken at an appropriate time-point during such investigations.

9.4.6. An appropriate level of root cause analysis work should be applied during the investigation of deviations. In cases where the true root cause(s) cannot be determined, consideration should be given to identifying the most likely root cause(s) and to addressing those. Where human error is suspected or identified as the cause of the deviation, this should be formally justified and care should be exercised so as to ensure that process, procedural or system-based errors or problems are not overlooked, if present.

9.4.7. The decisions that are made during and following investigations should reflect the level of risk that is presented by the deviation as well as the seriousness of any non-compliance with respect to the requirements of the blood component specifications or GP. Such decisions should be timely to ensure that patient safety is maintained, in a way that is commensurate with the level of risk that is presented by those issues.

9.4.8. As part of periodic Quality System reviews, an assessment should be made of whether corrective and preventive actions or any revalidation should be undertaken. The reasons for such corrective actions should be documented. Agreed CAPAs should be completed in a timely and effective manner. There should be procedures for the ongoing management and review of these actions and the effectiveness of these procedures should be verified during self-inspection.

10. Self-inspection, audits and improvements

10.1. Self-inspection or audit systems must be in place for all elements of operations to verify compliance

with the standards set out in the Annex to Directive 2005/62/EC. They must be carried out regularly by trained and competent persons, in an independent way, and according to approved procedures (Directive 2005/62/EC/Annex 10.1).

10.2. All results must be documented and appropriate corrective and preventive actions must be taken in a timely and effective manner (Directive 2005/62/EC/Annex 10.2).

11. Quality monitoring and control

11.1. Quality monitoring

11.1.1. Acceptance criteria must be based on a defined specification for each blood donation and blood component (specifications set out in the Standards section of *Chapter 5 – Component monographs* contained in the *Guide to the preparation, use and quality assurance of blood components* published by the Council of Europe may be used).

11.2. Quality control

11.2.1. All quality control procedures must be validated before use.

11.2.2. Results of quality-control testing must be evaluated continuously and steps taken to correct defective procedures or equipment.

11.2.3. Standard procedures for the quality control of blood components must be in place. The suitability of each analytical method to provide the intended information must be validated.

11.2.4. Quality control of blood and blood components must be carried out according to a sampling plan designed to provide the intended information.

11.2.5. Testing must be done in accordance with the instructions recommended by the manufacturer of the reagents and/or test kits.

11.2.6. The performance of the testing procedures must be regularly assessed by participation in a formal system of proficiency testing.

11.2.7. Records of quality-control procedures must include identification of the person(s) undertaking the tests or procedures. Any corrective action taken must also be recorded. If corrections in records are necessary, the original recording must not be obliterated, but must remain legible.

PRINCIPLES

Chapter 1

Introduction

The Principles section of this *Guide* contains background information to support the Standards. It also provides information on developments that have yet to be incorporated into Standards and on technical changes in the field. This includes information on the 'why and how' for work in blood establishments and hospital blood banks.

In addition, several appendices are provided at the end of this *Guide*. These appendices provide detailed information on specific areas of relevance to blood establishments and hospital blood banks which are not addressed in detail elsewhere in the *Guide*.

Chapter 2

Principles of donor selection

1. General remarks

Principles of self-sufficiency from voluntary and non-remunerated donations have been recommended and promoted by the Council of Europe and have been defined in Article 2 of Council of Europe Recommendation No. R (95) 14 as follows:

The definition of voluntary and non-remunerated donation is:

> Donation is considered voluntary and non-remunerated if the person gives blood, plasma or cellular components of his/her own free will and receives no payment for it, either in the form of cash, or in kind which could be considered a substitute for money. This would include time off work other than that reasonably needed for the donation and travel. Small tokens, refreshments and reimbursements of direct travel costs are compatible with voluntary, non-remunerated donation.

They have also been adopted by the EU in Directive 2002/98 EC, which states in the preamble (23): 'The definition of voluntary and unpaid donation of the Council of Europe should be taken into account', and, in Article 20 paragraph 1: 'Member states shall take the necessary measures to encourage voluntary and unpaid blood

donations with a view to ensuring that blood and blood components are in so far as possible provided from such donations.'

Specific immunisation programmes are not considered in this document, but donors enrolled for this purpose should at least fulfil the minimum criteria outlined above (see also Annex 2, Requirements for the collection, processing and quality control of blood, blood components and plasma derivatives, WHO Technical Report Series, No. 840, 1994).

2. Overview

This chapter considers the principles for the selection of donors of whole blood and also donors of components obtained by different apheresis procedures. There are general principles which apply to all donors. Some criteria for the selection of donors vary according to the type of donation involved. There are also further requirements specific to donors of different components collected by different methods.

Selection of donors of haematopoietic progenitor cells is discussed in the *Guide to the quality and safety of tissues and cells for human application* (EDQM/Council of Europe).

The main purpose of selecting individuals for blood and blood component donation is to determine whether the person is in good health so as to safeguard the health of both donor and recipient. All donors undergo a screening process to assess their eligibility (see *Standards*).

The screening process involves:

- provision of pre-donation educational material to all donors of blood or blood components;
- medical assessment of each donor.

Since blood establishments are ultimately responsible for the quality and safety of the blood and blood components collected, they are entitled to decide on the final acceptance or deferral of a donor or a prospective donor (Resolution CM/Res (2008) 5 on donor responsibility and on limitation to donation of blood and blood components,

adopted by the Committee of Ministers on 12 March 2008 at the 1021st meeting of the Ministers' Deputies).

3. Medical assessment of the donor

In practice, a complete medical and physical examination of donors is generally not possible. It is necessary to rely on the donors' appearance, their answers to questions concerning their medical history, general health, and relevant risk factors (e.g. lifestyle, travel history) and on laboratory tests.

Based on this information, a decision on the eligibility of the donor will be made using accepted guidelines. Conditions that are not covered by guidelines should be referred to the physician in charge with responsibility for making the final decision.

To obtain relevant and consistent information about the donor's medical history and general health, a standard questionnaire is to be completed at each donation. Adaptation of the questionnaire to the type of donor (first time, regular, apheresis donor, etc.) is recommended.

The key topics for donor eligibility to be covered by the questionnaire or by direct questions, the intentions of the interview questions, and examples of sample questions are included in Appendix 1.

Age of the donor

The standards set out in this *Guide* define age-limits for donation and provide discretion for the responsible physician to accept donors outside of these limits. This medical discretion can be applied either on an individual basis for a given donor or else through a systematic approach based on an appropriate risk assessment.

Hazardous occupations

Hazardous occupations or hobbies should normally require that there is an interval of not less than 12 hours between donation and returning to the occupation or hobby. Examples of such hazardous occupations or hobbies include piloting, bus or train driving, crane

operation, climbing of ladders or scaffolding, gliding, climbing and diving.

Donor deferral

Considering the requirement that only healthy people are acceptable as blood donors, deferral criteria can be grouped into:

- conditions requiring permanent deferral;
- conditions requiring temporary deferral for a defined duration;
- vaccination;
- conditions requiring individual assessment;
- infectious diseases.

Conditions requiring permanent deferral

See Standards.

Conditions requiring temporary deferral (suspension)

See Standards.

Vaccination

See Standards.

Conditions requiring individual assessment

As donors may present with a variety of prior or current medical problems, only some of the more common examples are considered in Table 2.1 below.

Table 2.1. Conditions requiring individual assessment of the donor

Condition requiring individual assessment	Criteria for deferral
Allergy	Individuals with a documented history of anaphylaxis should not be accepted as donors.
Auto-immune diseases	If more than one organ is affected this leads to permanent deferral.
Beta-thalassaemia trait	Heterozygote carriers of beta-thalassaemia trait may give blood provided they are in good health and have a haemoglobin level within acceptable values.
Bronchitis	Persons with symptoms of severe chronic bronchitis should not be accepted as donors.
Common cold	Accept, if asymptomatic and feels well on the day of donation.
Hypertension	A person who presents with a systolic blood pressure of more than 180 mm Hg or a diastolic blood pressure of more than 100 mm Hg should not be accepted as a blood donor. A mild hypertensive, whose diastolic blood pressure is maintained at less than 100 mm Hg, may be accepted.
Pulse [a]	The pulse should be regular and between 50 beats per minute (bpm) and 100 bpm. Exceptions may be made to accept donors with a lower pulse rate following individual medical review, e.g. athletes.
Jaundice and hepatitis (see Standards)	Hospital staff coming into direct contact with patients with hepatitis are accepted at the discretion of the physician in charge of the blood-collecting unit providing they have not suffered an inoculation injury or mucous membrane exposure, in which case they must be deferred.
Chagas disease (see Standards)	In some countries, donors who were born or have been transfused in areas where the disease is endemic are deferred or tested. The blood of persons who were born or have been transfused in areas where the disease is endemic should be used only for plasma fractionation products unless a validated test for infection with *T. cruzi* is negative.

a Where measured.

Post-donation information

See Standards.

Systems should be in place to define the actions to be taken if a donor informs the blood establishment that he/she previously donated blood but should not have done so in the light of donor selection criteria aimed at protection of the health of recipients (e.g. in retrospect, the donor did not fulfil the criteria mentioned in the donor questionnaire).

Infectious diseases

Transmission of infectious agents by transfusion can be minimised by careful and appropriate use of donor questionnaires and/or laboratory testing. Donors should be questioned on their risk of exposure to infectious agents, which includes taking a travel history.

For infections in which the agent has been fully cleared from the donor's blood on recovery, the donor should be deferred from donation until they are no longer infectious (usually 2 weeks from cessation of symptoms).

In cases of known contact with an infectious agent, the donor should be deferred for approximately twice the length of the incubation period. In case of a geographical risk of exposure to multiple infectious agents, the longest deferral period applies.

Other measures are needed for infections where there is a possibility of asymptomatic infection or existence of a carrier state. Questioning donors about symptoms in these circumstances does not always prevent transmission.

Many infections that can be transmitted by transfusion have defined geographical limits, and the risk of transfusion transmission can be minimised by temporary deferral or testing donors travelling from affected areas (see Table 2.2). Testing becomes especially relevant when deferral policies may potentially affect supply.

Table 2.2. Recommended donor deferral after exposure to infectious agents if testing is not in place

Infectious agents	Incubation period (days)	Strategies to prevent TTI from healthy donors	Strategies to prevent TTI after clinical disease
West Nile virus	2-14	28-day deferral after leaving the risk area	120-day deferral after resolution of symptoms
Chikungunya virus	1-12		
Dengue virus	5-6		
Zika virus	3-12		

Blood services should maintain a watching brief on changes to risks of infectious diseases worldwide. Risk-benefit analyses should be carried out to determine appropriate measures to decrease the risks of infectious diseases in their own country. The risk of importation of an infectious agent through donors visiting an affected area should be balanced by considering the likelihood of this occurring, and the impact of introducing a new donor deferral ruling on blood collection. This risk will vary between countries.

New and emerging infectious agents or those that have moved to infect a new geographical area can also pose a significant challenge. The West Nile virus, dengue virus, Babesiosis, Q fever and the Chikungunya virus are examples of such viruses/diseases. In this situation, donor deferral may not be an option in the newly affected area. Donation testing is then the main tool to reduce the risk of transmission. For plasma and platelets, pathogen-reduction technology may also be considered.

Information about new and emerging infections should be communicated between countries without delay to allow blood establishments to consider their own risks and appropriate actions.

Variant Creutzfeldt-Jakob disease (vCJD) was first described in the UK in 1996. Clinical presentation differs from classical CJD in that it

tends to affect a younger age group, and there is greater involvement of lymphoid tissue.

Estimating the potential size of the vCJD epidemic has been very difficult. Transfusion transmission of vCJD has been documented in animal studies and in humans.

Several measures have been taken in the UK to reduce the risk of further transmission by blood transfusion. Measures include: universal depletion of leucocytes; importation of plasma for fractionation and clinical use in children who have not been exposed to BSE through their diet; lifetime deferral of donors who have been transfused; reducing inappropriate transfusions (particularly for surgical indications). In addition, patients deemed to be 'at risk' because of receiving blood from an implicated donor or receiving a large amount of blood, including fractionated products, are advised not to donate blood, tissues or organs, and have special measures taken during surgery such as use of disposable or dedicated instruments.

Many countries outside the UK defer donors who have lived in the UK for a minimum defined period between 1980 and 1996; the European Medicines Agency (EMA) mandates 1 year of UK residence for donors of plasma for fractionation. In some instances the deferrals have been extended to include donors from other countries with a significant number of cases.

Endogenous risk of vCJD differs between countries. Therefore, different measures to reduce risk will be appropriate depending on each country's own risk assessment: balancing risk with sufficiency of supply.

History of malignancy

Individuals with a malignant disease or a history of malignancy are usually permanently deferred (see Standards). However, there is a lack of evidence to support the theoretical concerns that cancer is transmitted via blood.

Large observational studies have provided convincing evidence that the risk of transmitting cancer via blood transfusions is undetectable or not significant.

Based on this, donors with a history of malignancy may be considered using the following criteria:

- permanent deferral for any history of haematological malignancies (e.g. leukaemia, lymphoma, myeloma);
- permanent deferral for any history of malignancies known to be associated with viraemic conditions (except for carcinoma *in situ* of the cervix, see below);
- for other cancers, the donor should have fully recovered with no expectation of recurrence (i.e. cured) and the following conditions apply:
  - for cancers with negligible metastatic potential (e.g. basal cell carcinoma and carcinoma *in situ* of the cervix), the donor may be accepted immediately following successful removal and cure;
  - for all other cancers, at least 5 years should have elapsed since completion of active treatment;
- no deferral is required for pre-malignant conditions.

4. Specific considerations for donors of different components

Quantity of whole blood donation

In addition to the standard volume of a whole-blood donation (see Standards), up to 35 mL of blood is collected for laboratory tests and for retention of a donation sample.

Because of the risk of adverse reactions, no more than 15 % of the estimated blood volume should be collected as whole blood during one blood donation. The blood volume of the donor can be calculated from their weight, height and gender using a validated formula (it is recommended to calculate the blood volume using the formula developed by

the International Council for Standardisation in Haematology, ICSH). The ICSH formula is derived from a large European study population in which measurements of red cell mass and plasma volume were carried out.[1]

It is generally accepted that all men weighing ≥ 50 kg have a sufficiently large blood volume to donate a total 535 mL of blood (500 mL plus 35 mL for testing and retention of a donation sample), whilst all women weighing ≥ 50 kg have a sufficiently large blood volume to donate a total 485 mL of blood (450 mL plus 35 mL for testing and retention of a donation sample).

In the case of women weighing < 65 kg and donating a total > 485 mL, the estimated blood volume should be calculated. The calculated blood volume should exceed the minimum acceptable blood volume for the volume of blood to be collected (see Table 2.3). If the calculated blood volume is less than acceptable, a smaller volume should be collected or the donor should be deferred.

If the donation volume may exceed 15 % of the blood volume of the donor, blood establishments should use a blood-volume table prepared according to the ICSH formula for checking the blood volume of the donor (see examples in Appendix 2).

Frequency of whole blood donation

It is recommended that an active donor panel of sufficient size be maintained to allow donors to be bled less often than the maximum annual rates stated in *Standards*. Four whole blood donations for males and three donations for females should ordinarily not be exceeded per year, thereby affording the donors extra protection and giving the system flexibility to deal with large-scale emergency situations.

---

1 Pearson TC, Guthrie DL, Simpson J, Chinn C, Barosi G, Ferrant A, Lewis SM, Najean Y. Interpretation of measured red cell mass and plasma volume in adults: Expert Panel on Radionuclides of the International Council for Standardisation in Haematology. *Br J Haem* 1995, 89: 748-56.

Table 2.3. Calculated minimum blood volume of a female donor donating 485 mL, 510 mL or 535 mL

Volume of blood to be collected	Maximum percentage of blood volume collected	Minimum acceptable blood volume
450 mL + 35 mL	15 %	3 233 mL
475 mL + 35 mL	15 %	3 400 mL
500 mL + 35 mL	15 %	3 567 mL

Laboratory examination before donation

Hb level

Haemoglobin should be measured at each donation, preferably before collection. Abnormally high and abnormally low haemoglobin values should be confirmed by a full blood count on a venous sample and, if appropriate, subsequently investigated (as should a fall in haemoglobin concentration of more than 20 g/L between two successive donations).

Iron stores

It is recognised that blood donation may result in iron deficiency in repeat blood donors. This problem may arise without it being evident through pre-donation haemoglobin measurements. This may be especially important in women of child-bearing age and in donors with inadequate dietary iron intake. Blood establishments should include appropriate measures to minimise this problem, and to protect donor health. Such measures may include:

- the provision of materials for donor education, particularly in regard to dietary counselling to increase iron intake and the impact of blood donation on iron stores;
- the individual tailoring of donation frequency and/or type of blood component donation based on iron status;

- the use of tests to assess iron status, such as ferritin, soluble transferrin receptor, zinc protoporphyrin and/or RBC indices;
- iron supplementation may be considered, taking into account the risk of delaying the diagnosis of unapparent underlying diseases, such as gastric/intestinal cancer. The donor needs to be informed of the side-effects of the iron preparation.

At the same time, blood establishments should recognise that many donors currently deferred because of low haemoglobin levels are in a satisfactory state of health. Iron stores recover following an appropriate temporary deferral period. Therefore, use of appropriate measures for the prevention and management of iron deficiency in blood donors not only improves their wellbeing, but may also contribute significantly to achieving sufficiency of blood supply.

Apheresis donors

General remarks

Written informed consent should be obtained before the first apheresis procedure.

The standards require that the maximum extracorporeal volume (ECV) of 20 % not be exceeded. For donors weighing 50-65 kg, the total blood volume should be estimated using the approach described in Appendix 2.

The standards identify the maximum annual donation frequency, the minimum inter-donation intervals and the maximum volumes of components to be collected by apheresis.

The impact of incomplete apheresis procedures, including consideration of non-reinfusion of red cells and the amount of primary component already collected, needs to be taken into account when determining compliance with these requirements.

For apheresis procedures where cells are harvested, the haemoglobin levels defined in the standards apply.

There is increasing concern about long-term effects in donors in intensive apheresis programmes. This includes risks associated with citrate exposure in regular platelet apheresis donors, leading to problems with bone mineral density and diminished IgG levels after long-term intensive plasmapheresis.

Special attention should be given to the following conditions:

- abnormal bleeding episodes;
- a history suggestive of fluid retention (of special interest if steroids and/or plasma expanders are to be used);
- intake of drugs containing acetylsalicylic acid or other platelet-inhibiting components. Platelet apheresis should not be undertaken within 48 h after the last intake of acetylsalicylic acid or piroxicam;
- a history of gastric symptoms (if steroids are to be used);
- adverse reactions to previous donations.

Frequency of apheresis donation and maximal amount of collected plasma

Current recommendations are made in the absence of conclusive studies of outcomes from different regimes of volumes and frequencies of plasmapheresis. Despite some data being available from studies with several years of follow-up, further short- and long-term prospective studies are needed and should be undertaken.

The collection volume (excluding anti-coagulant) for each plasmapheresis procedure must not exceed 16 % of the estimated total blood volume (see *Standards*). The total blood volume should be calculated on the basis of gender, height and weight. Alternatively, a collection volume based on 10.5 mL per kg of body weight broadly equates to 16 % of estimated total blood volume. Short-term effects can be reduced by observing the maximum extracorporeal volume (ECVmax), which must never exceed 20 % (see *Standards*), with a recommended guidance value of 16 %.

Sampling and residual blood remaining in the plasmapheresis devices can result in a non-negligible loss of red cells, with a consequent reduction in serum iron and ferritin. This is especially important for female donors. Haemoglobin levels should be determined at each donation. The minimum value for plasmapheresis should be 120 g/L and 130 g/L for female and male donors, respectively.

Additional recommendations for platelet apheresis

If undertaking high-dose platelet collection (> 5×10^{11} platelets/unit), care should be taken to ensure that the post-donation count does not fall below 100×10^9/L.

Additional recommendations for granulocytapheresis

Clinical efficacy, indications and dosage of granulocyte transfusion have not been established. Prior to collection, the potential donor of granulocytes needs to receive medication, and sedimenting agents may be needed during the apheresis procedure. Both of these have potentially severe side-effects that need to be communicated to the donor as part of the informed consent process.

In addition to the recognised complications of routine donor apheresis, the following side-effects may occur.

- Hydroxyethyl starch (HES): acts as a plasma volume expander. Potential donors should be questioned and/or screened for evidence of renal disease as HES may exacerbate pre-existing disease. Donors who have received HES may experience headaches or peripheral oedema because of expanded circulatory volume. HES may accumulate, which can result in pruritus, and allergic reactions.
- Corticosteroids: may cause, for example, hypertension, diabetes mellitus, cataracts, peptic ulcer, and psychiatric problems.
- Granulocyte-colony stimulating factor (G-CSF): the most common short-term complication following G-CSF administration in peripheral blood stem cell (PBSC) donors is bone pain; although, on very rare occasions, splenic rupture or lung

injury may occur. Concerns relating to the development of acute myeloid leukaemia (AML)/myelodysplasia (MDS) after G-CSF administration are based primarily on reports of increased rates of these disorders among women with breast cancer who received chemotherapy or patients with severe chronic neutropenia (SCN) who received G-CSF support. To date, however, registry data from Europe and the United States have not identified any increased risk of AML/ MDS in over 100 000 healthy individuals who donated PBSCs and received G-CSF as pre-treatment. The median follow-up of these studies is, however, less than 5 years. Therefore, if G-CSF is given to a donor, a protocol for long-term follow-up should be in place (as advised by JACIE/ FACT).[1]

Additional recommendations for donors of red cells for anti-RhD immunisation

Specific protocols for donors of red cells for anti RhD immunisation should be in place and should at least include the following:

- additional testing of markers of infectious disease, such as anti-HTLV-I/II, anti-HBc and NAT tests for pro-viral HIV-DNA and HIV-RNA, HCV-RNA, HBV-DNA, HAV RNA and parvovirus B19DNA or parvovirus B19- antibodies;
- extensive red cell phenotyping should be performed at least twice, and may be supplemented by genotyping;
- the red cells for immunisation should be stored for at least 6 months. After 6 months, all the infectious markers stated above should have been found to be negative (or indicate absence of infection) on a new donor sample before release of the stored red cells for immunisation.

1 JACIE/FACT. International Standards for Cellular Therapy Product Collection, Processing and Administration [available from: www.jacie.org, accessed 4 December 2014].

In order to manage the impact of changes in donor selection criteria and infectious marker testing that may occur over time, protocols should require:

- maintenance of retention samples from each donation suitable for future testing;
- re-qualification of past donations by assessing conformance with additional donor acceptance requirements including, where appropriate, testing of the donor and/or the retention sample.

Exemption of past donations from current standards is not recommended and should only be considered in exceptional circumstances after careful considerations of the risks to the immunised donors and ultimate plasma product recipients.

Designated donations

Although blood donation is voluntary, non-remunerated and anonymous, in some special circumstances it may be necessary to make use of designated donations. This should happen only for clear medical indications. Designated donors should be screened and tested like volunteer allogeneic donors.

Designated donations are those intended for named patients based on medical indications. These donations may involve family members, in which case the physician responsible should weigh up the risks and benefits for the patient. The practice of transfusing parental blood to infants is not without risk. Mothers may have antibodies to antigens that are present on the infant's red blood cells, platelets or white blood cells. Therefore, maternal plasma should not be transfused. Fathers should not serve as cell donors to neonates because maternal antibodies to antigens inherited from the father may have been transmitted through the placenta to the foetus. In addition, due to partial histocompatibility, transfusions of cells from parental or family donors carry an increased risk of transfusion-associated graft versus host disease, even in the immunocompetent recipient, and so such components should be irradiated. In the case of platelets,

pathogen-reduction technologies for components may be used as an alternative to irradiation.

Circumstances where designated donations may be indicated include:

- for patients with rare blood types, where no compatible anonymous donations are available;
- where donor-specific transfusions are indicated for immune modulation or immunotherapy; for instance, in the preparation procedure for kidney transplants or for lymphocyte transfusions aimed at a graft-versus-leukaemia effect;
- in certain cases of allo-immune neonatal thrombocytopaenia; for instance, if HPA-typed platelets are not available and intravenous immunoglobulin therapy is not sufficient.

Directed donations

Directed donations are those intended for named patients, where the request for the donation has been made by patients, relatives or friends. The public often believes that directed donations are safer than anonymous, voluntary, non-remunerated donations. However, this is not the case: even if directed donations are screened and tested in the same manner as voluntary non-remunerated donations, infectious disease marker rates are generally higher among directed donors.

Directed donations are not considered good practice and should be discouraged.

Chapter 3

Principles of blood collection

1. Overview

Records should be kept for each activity associated with the donation. The record should also reflect any unsuccessful donation, the rejection of a donor, adverse reactions or unexpected events. A qualified health professional should sign the donor selection records and the final assessment.

Sterile collection systems should be used in accordance with the instructions of the manufacturer. A check should be made before use to ensure that the collection system used is not damaged or contaminated and that it is appropriate for the intended collection. Defects in blood bags should be reported to the supplier and subjected to trend analysis.

The donor identification, donor selection interview and donor assessment should take place before each donation. The donor should be re-identified immediately prior to venepuncture.

2. Premises for donor sessions

When the venue of the donor session is permanent and under the control of the transfusion establishment, provision should be made for proper cleaning by, for example, the use of a non-slip, washable

floor material that is installed so as not to have inaccessible corners and by avoiding having internal window ledges, etc. If possible, air-conditioning units should be used to provide ventilation to avoid the need for open windows. Air changes, together with temperature and humidity control, should be adequate to cope with the maximum number of people likely to be in the room and with the heat output from any equipment used.

When sessions are performed by mobile teams, a realistic attitude towards environmental standards may be taken. Factors to consider should include adequate heating, lighting and ventilation, general cleanliness, provision of a secure supply of water and electricity, adequate sanitation, compliance with fire regulations, satisfactory access for unloading and loading of equipment by the mobile team, adequate space to allow free access to the bleed and rest beds.

3. Equipment used at blood donation sessions

It is recommended that the manufacturer's identity and container information (catalogue number and the container number of the set), as well as the manufacturer's lot number, should be given in eye- and machine-readable codes.

4. Pre-donation checks and labelling

Defects may be hidden behind the label pasted on the container. Abnormal moisture or discolouration on the surface of the bag or label after unpacking suggests leakage through a defect.

The unique identity number usually consists of a code for the responsible blood collection organisation, the year of donation and a serial number.

5. Venepuncture

Preparation of the venepuncture site

The venepuncture site should be prepared using a defined and validated disinfection procedure. Compliance with the disinfection

procedure should be monitored and corrective action taken where indicated.

Although it is impossible to guarantee sterility of the skin surface for phlebotomy, a strict, standardised procedure for the preparation of the phlebotomy area must exist (see *Standards*). Of particular importance is that the antiseptic solution used be allowed to dry completely before venepuncture. The time taken for this to happen will vary with the product used, Manufacturer's instructions should be followed.

The prepared area must not be touched before the needle has been inserted (see *Standards*).

Successful venepuncture and proper mixing

If an anticoagulant solution is used, the collection bag should be mixed gently immediately after collection has begun and at regular intervals thereafter during the entire collection period. The maximum collection time for acceptance of the donation for component processing should be specified and controlled. Donations that exceed the maximum time period should be recorded and discarded.

Proper mixing of the blood with the anticoagulant is essential at all phases of the collection.

Attention should be paid to the following:

- as the blood begins to flow into the collection bag, it must immediately come into contact with the anticoagulant and be properly mixed;
- the flow of the blood must be sufficient and uninterrupted;
- donation of a whole blood unit should ideally not last more than 10 minutes. If the duration of the collection is longer than 12 minutes, the blood should not be used for the preparation of platelets. If the duration of the collection is longer than 15 minutes, the plasma should not be used for direct transfusion or for the preparation of coagulation factors;

- in apheresis, any unexpected interruption of the flow occurring during the procedure should be assessed to determine if the component is acceptable for use.

Handling of filled containers and samples

At completion of the donation, the donation number issued should be checked on all records, blood bags and laboratory samples. Donation number labels that have not been used should be destroyed via a controlled procedure. Routine procedures to prevent misidentification should be in place.

If integral blood bag collection tubing is to be used to prepare segments for testing, it should be sealed off at the end and then filled with anti-coagulated blood as soon as possible after blood collection.

After blood collection, the blood bags should be handled, transported and placed into storage according to defined procedures.

Immediately after sealing the distal end of the collection bag, the contents of the bag line should be completely discharged into the bag.

Procedures should be designed to avoid the possibility of errors in labelling of blood containers and blood samples. It is recommended that each donor bed should have individual facilities for the handling of samples during donation and labelling.

Donor screening samples should be taken at the time of donation. Procedures should be designed to minimise the risk of bacterial contamination of the collected blood or deterioration of the sample, and to prevent potential misidentification.

The test samples should be taken directly from the bleed line or from a sample pouch (deviation bag) of the collection system.

If samples are taken at the end of donation, this must be done immediately.

The blood bag and corresponding samples must not be removed from the donor's bedside until correct labelling has been checked and verified.

After collection, blood bags should be placed promptly into controlled-temperature storage and transported to the processing site under temperature conditions appropriate for the component that is to be prepared. There should be validation data to demonstrate that the storage and transport conditions used after collection ensure maintenance of the blood within the specified temperature range.

6. Apheresis

Pre-medication and apheresis

With the exception of granulocyte donors, pre-medication of donors for the purpose of increasing component yield is not recommended.

Caution is recommended regarding pre-treatment of donors with corticosteroids and G-CSF.

Automated apheresis

It is recommended to exercise caution to prevent any misconnection of the different components of the apheresis set; in particular, confusion between anticoagulant and saline, which could result in serious adverse reaction in donors.

Manual apheresis

Manual apheresis is no longer recommended.

7. Repository of archive samples

Retention of donor samples for a period of time may provide useful information. Provision of such systems is contingent on the availability of adequate resources.

If archive samples from the donations are kept, then procedures must be in place prescribing their use and final disposal (see *Standards*).

8. Management of adverse reactions in donors

Special attention should be given to all donors in whom an adverse reaction in relation to blood donation has been identified.

In the case of an adverse reaction, the donor should be referred as soon as possible to a designated healthcare worker/physician of the session.

The source of the adverse reaction should be identified and corrective and preventive measures considered.

All adverse reactions, including the treatment and preventive actions taken, should be documented in the donor's records and those of the quality system.

Severe adverse reactions in donors should be reported to the nationally established haemovigilance system (see Chapter 10, Principles of haemovigilance; and Chapter 10, Standards of haemovigilance).

Table 3.1. Examples of adverse reactions related to blood collection

**Local reactions related to needle insertion**	
Vessel injuries	Haematomas<br>Arterial puncture<br>Thrombophlebitis
Nerve injuries	Injury of nerve<br>Injury of nerve by haematoma
Other complications	Tendon injury<br>Allergic reaction (local)<br>Infection (local)

**General reactions**	
Vasovagal reaction	Immediate type<br>Delayed type

Rare, significant complications	
Related to vessel injury	Pseudoaneurysm in the brachial artery<br>Arteriovenous fistulae<br>Compartment syndrome
Accidents	Accidents or injuries related to vasovagal syncope<br>Other kinds of accidents
Cardiovascular reactions	Angina pectoris<br>Myocardial infarction<br>Cerebral ischaemia
Related to apheresis procedures	Citrate toxicity<br>Systemic allergic reaction<br>Anaphylaxis<br>Haemolysis<br>Air embolism

Prevention of adverse reactions in donors

Prospective donors must be informed of the possible adverse reactions of blood donation and how they can be prevented.

Training of the personnel collecting blood should include preventing and recognising the (early) signs of adverse reactions and their rapid treatment.

The physician in charge is responsible for the medical supervision of blood collection, and each session must be staffed with a qualified healthcare professional.

Treatment of adverse reactions in donors

The treatment of adverse reactions related to blood donation must be described in standard operating procedures.

Staff must be properly and regularly trained to be attentive for the early signs of an adverse reaction and be able to respond immediately with the appropriate action.

In each collection facility, a specific space should be reserved for dealing with donors who have an adverse reaction.

The donor should be observed until fully recovered and, in the event of a serious adverse reaction, the blood establishment must remain in contact with the donor until the complication has disappeared or the donor is in a stable condition.

Documentation of adverse reactions in donors

The treatment and outcome of all adverse reactions related to blood donation, at any stage of the procedure, must be fully documented.

The physician in charge must be immediately informed about all serious adverse reactions.

Data should be collected and analysed in order to initiate corrective actions that could prevent or reduce the frequency or minimise the severity of adverse reactions in the future.

Serious adverse reactions must also be reported to the appropriate national authority.

Information for a donor with adverse reactions

When an adverse reaction occurs, the donor must be informed about the reaction, its treatment and the expected outcome. The donor should be given the opportunity to contact the on-call healthcare professional of the blood establishment at any time.

The collection staff should instruct the donor in post-collection care and they should keep the donor under observation until he/she is released.

In particular, a donor who has experienced vasovagal reactions should be informed about the risk of delayed fainting. The donor should not drive a vehicle or resume work or any hazardous occupation or hobby in the ensuing 12 hours if delayed fainting could put the donor or other persons at risk.

9. Donor clinic documentation

Full records should be maintained at blood donation sessions to cover the following parameters:

- the blood component (s) collected, the date, donation number, identity and medical history of the donor;
- the date, donation number, identity and medical history of the donor for each unsuccessful donation, together with reasons for the failure of the donation;
- recording of rejected donors following assessment, together with the reasons for their rejection;
- full details of any adverse reactions in a donor at any stage of the procedure;
- in the case of apheresis, the volumes of blood collected, blood processed, and replacement solution and anticoagulant used.

As far as possible, the records of blood donation sessions should allow blood transfusion staff to identify each important phase associated with the donation. These records should be used for the regular compilation of statistics, which should be studied by the individual with ultimate responsibility for the blood donation session, who can take appropriate actions as deemed necessary.

Chapter 4

Principles of blood component processing

1. Overview

In the past, transfusion therapy was largely dependent on the use of whole blood. While whole blood may still be used in certain limited circumstances, the main thrust of modern transfusion therapy is to use the specific component that is clinically indicated. Components are those therapeutic constituents of blood that can be prepared by centrifugation, filtration and freezing using conventional blood bank methodologies.

Transfusions are used mainly for the following purposes:

- to maintain oxygen/carbon dioxide transport;
- to correct or avoid bleeding and coagulation disorders.

Clearly, whole blood is not necessarily suitable for all these purposes, unless the patient requiring treatment has multiple deficiencies. Even then, frequent storage defects in whole blood make it unsuitable. Patients should only be given the component needed to correct their specific deficiency. This avoids unnecessary and possibly harmful infusions of surplus constituents. The change from collecting blood in glass bottles to multiple plastic bag systems has greatly facilitated the

preparation of high-quality components. Storage considerations are a major reason for promoting the use of individual blood components.

Optimal conditions and, consequently, shelf-lives vary for different components. Red cells maintain optimal functional capability when they are refrigerated. The quality of plasma constituents is best maintained in the frozen state while platelet storage is optimal at room temperature (20-24 °C) with continuous agitation. Thus, only the storage requirements of red cells are fulfilled if whole blood is refrigerated, with a consequent loss of therapeutic effectiveness of most of the other constituents.

Component therapy also offers logistic, ethical and economic advantages. The majority of patients requiring transfusions do not need the plasma in a whole blood unit. Production of plasma-derived products can thus be facilitated by the use of red cells rather than whole blood. Leucocyte depletion may further improve the quality of blood components.

2. Processing procedures

Blood components may be prepared during collection using apheresis technology. Plasma, leucocytes, platelets and red cell concentrates may be obtained in this way. Alternatively, whole blood may be collected in the traditional manner, with individual components extracted using post-donation processing of whole blood.

Time and temperature limits should be defined for the processing of blood components including filtration. Due to the potential deterioration of activity and functionality of labile blood components, the conditions of storage and time before processing are vital to preparation of high-quality blood components. Delays in preparation or unsuitable storage conditions may affect the quality of the final components adversely.

3. Choice of anticoagulant and bag system

Whole blood is collected into a bag containing an anticoagulant solution. The solution contains citrate and cell nutrients such as

glucose and adenine. The first centrifugation steps remove more than half of these nutrients from the residual red cells. Thus, it may be more logical to provide the proper nutrients for the cells using a re-suspension medium instead of incorporating them in the initial anticoagulant solution.

Plasticware used for blood collection, apheresis and component preparation should comply with the requirements of the relevant monographs of the European Pharmacopoeia with regard to haemocompatibility, in addition to its suitability for achieving the respective technological goal. Polyvinylchloride (PVC) has been found to be satisfactory for red blood cell storage. Biocompatibility of any plasticisers used must be ensured. Storage of platelets at + 20-24 °C necessitates use of a plastic with increased oxygen permeability. This feature has been achieved by plastic materials of alternative physical and/or chemical characteristics. Leaching of plasticisers into blood/blood component should not pose undue risk to the recipient. Any possible leaching of adhesives from labels or other device components should be kept within acceptable safety limits. Care should be taken to minimise the levels of residual toxic substances after sterilisation (e.g. ethylene oxide).

Whenever use of new plastics is being considered, an adequate study of component preparation and/or storage should be conducted. The following parameters could be useful:

- red blood cells: glucose, pH, haematocrit, haemolysis, ATP, lactate, extracellular potassium and 2,3-bisphosphoglycerate;
- platelets: pH, pO_2, pCO_2, bicarbonate ion, glucose, lactate accumulation, ATP, P-selectin, LDH release, beta thromboglobulin release, response from hypotonic shock and swirling phenomenon, morphology score and extent of shape change;
- plasma: Factor VIII and signs of coagulation activation (e.g. thrombin–anti-thrombin complexes).

These studies are normally carried out by the manufacturer before introduction of the new plastics and the results should be made available to the transfusion services.

The suitability of new plastics can be determined by the evaluation of post-transfusion *in vivo* recovery and survival of autologous red cells after 24 hours and by the assessment of platelet recovery, survival and corrected count increments (CCI).

In order to maintain a closed system throughout the separation procedure, a multiple bag configuration (either ready-made or sterile-docked) should be used. The design and arrangement of the packaging system should permit sterile preparation of the desired component.

Although use of closed systems is recommended for all steps in component processing, open systems may sometimes be necessary due to local constraints. If open systems are employed, they should be carried out in an environment designed specifically to minimise the risk of bacterial contamination, and careful attention should be given to use of aseptic procedures. Red cells prepared in open systems should be transfused within 24 hours of processing. Platelets prepared in open systems should be transfused within 6 hours of processing.

4. Centrifugation of whole blood derived blood components

The sedimentation behaviour of blood cells is determined primarily by their size as well as the difference of their density from that of the surrounding fluid (see Table 4.1 below). Other factors are the viscosity of the medium and flexibility of the cells (which is temperature-dependent). The optimal temperature for centrifugation with respect to these factors is + 20 °C or higher.

Table 4.1. Volume and density of principal blood constituents

	Mean density (g/mL)	Mean corpuscle volume (fL)
Plasma	1.026	
Platelets	1.058	9
Monocytes	1.062	470
Lymphocytes	1.070	230
Neutrophils	1.082	450
Red cells	1.100	87
Saline / SAGM	1.003	N/A

In the first phase of centrifugation, the surrounding fluid is a mixture of plasma and anticoagulant solution. Leucocytes and red cells centrifuge out more rapidly than platelets as they both have a bigger volume than platelets. Depending on the time and speed of centrifugation, most of the leucocytes and red cells settle in the lower half of the bag and the upper half contains platelet-rich plasma. More prolonged centrifugation results also in platelet sedimentation, driven by a force proportional to the square of the number of rotations per minute and the distance of each cell to the centre of the rotor, whereas the leucocytes (now surrounded by a fluid of higher density, i.e. the red cell mass) move upwards. At the end of centrifugation, cell-free plasma occupies the upper part of the bag and red cells are at the bottom.

Platelets accumulate on top of the red cell layer, while the majority of leucocytes are to be found immediately below the platelets in the top 10 mL of the red cell mass. Haematopoietic progenitor cells have similar characteristics to normal mononuclear blood cells. However, this component may be contaminated with immature or malignant cells from different haematopoietic lineages, which commonly have larger sizes and lower densities than their mature counterparts.

The conditions of centrifugation, such as g-force, acceleration, time, deceleration, etc., determine the composition of the desired component. For example, if platelet-rich plasma is desired, centrifugation should stop prior to the phase where platelet sedimentation commences. A low centrifugation speed allows for some variation in centrifugation time. If cell-free plasma is required, fast centrifugation for an adequate time allows separation into cell-poor plasma and densely packed cells. It is important that the optimal conditions for good separation be carefully standardised for each centrifuge. A number of options exist for the selection of a procedure for centrifugation for the preparation of components from whole blood.

Classical component preparation is performed by centrifuging the bags in a centrifuge with a capacity of 4 or 12 bags, followed by transfer of the bags to a manual or semi-automated expresser. Recently, technologies have become available integrating the expresser function into the centrifuge. These technologies can perform the classical component preparation methods, but also allow a novel method whereby an interim platelet unit is made after a hard spin. This interim platelet unit contains the major part of the platelets and some leucocytes, but no red cells that would need to be removed in a second separation step.

The choice to be made is whether or not the buffy coat is to be separated from the sedimented cells. The advantage of separation of the buffy coat is that red cells are relatively leucocyte-poor, which prevents formation of aggregates during storage. The red cells can be resuspended into a solution designed to offer optimal conditions for red cell storage, e.g. saline-adenine-glucose-mannitol (SAGM). The resuspension can still be done within the closed system. Plasma, after separation, can be frozen and stored as fresh frozen plasma to be used as such or as a starting material for other products, such as medicinal products derived from human plasma.

Filtration

At present, two major types of filtration are available for blood component preparation:

- the separation of plasma from blood by tangential filtration;
- the removal of leucocytes from cell suspensions by depth-filtration or surface filtration.

Washing of cellular components

This technique is occasionally used when there is a requirement for cellular blood components with a very low level of plasma protein.

5. Leucocyte depletion

To enable a comparison of the filters that can be used for leucocyte depletion and to facilitate selection between them, manufacturers should report data on their system performances under defined conditions. Manufacturers should also provide performance data to the blood establishment on variations between different filter types or modifications and between batches.

Mathematical models have been developed to calculate the sample size necessary to validate and control the leucocyte depletion process.

After full validation of the process, tools such as statistical process controls could be used to detect any on-going changes in the process and/or the procedures.

Particular problems may arise with donations from donors with red cell abnormalities (e.g. sickle-cell traits) where adequate leucocyte depletion may not be achieved and more detailed quality control procedures are necessary (e.g. leucocyte counting of every donation). The quality of the red cells collected after slow filtration processes needs further investigation.

6. Freezing and thawing of plasma

Rationale

Freezing is a critical step in the preservation of some plasma proteins, including coagulation factors (in particular Factor VIII). During freezing, pure ice is formed and the plasma solutes are concentrated in the remaining water. Each solute forms crystals when the solubility

of the solutes is exceeded, but this may be influenced by the anticoagulants used.

Ice formation depends on the rate of heat extraction, whereas the diffusion rates of the solutes determine their displacement. At slow freezing rates, the diffusion of solutes is better adapted to the rate of ice formation and so solutes are increasingly concentrated in the middle of a plasma unit.

Since all solutes are displaced simultaneously, the Factor VIII molecules are exposed to a high concentration of salts for a prolonged period of time and are thus inactivated. At a high freezing rate, ice formation overtakes solute displacement. Hence, small clusters of solidified solute are trapped homogeneously in the ice without prolonged contact between the highly concentrated salts and Factor VIII.

To achieve the highest yield of Factor VIII, plasma should be frozen to – 25 °C or lower.

A reduction in Factor VIII content occurs during freezing when the solidification of plasma takes more than one hour. This can be monitored by measuring the total protein content of a core sample of the frozen plasma; the protein concentration should be identical to the total protein content of plasma before freezing. Heat extraction of 38 kcal per hour per unit of plasma is an optimal freezing rate, and can be monitored by use of thermocouples.

To incorporate these methods effectively into a coherent daily routine, the staff of the blood establishment must be familiar with the rationale behind the method, as well as its potential limitations and pitfalls.

Methods of freezing

If freezing plasma, the rate of cooling should be as rapid as possible and, optimally, should bring the core temperature down to – 25 °C or below within 60 minutes.

Experience has shown that without the use of a snap-freezer, it takes several hours to reach this temperature. This time can be reduced, for example, by placing plasma batches in a regular configuration to

maximise exposure to the freezing process (e.g. bags laid flat or, if vertical, in formers) and immersed in an environment at a very low temperature. If a liquid environment is used, it should have been demonstrated that the container cannot be penetrated by the solvent (see Standards, Chapter 5, Component monographs, and the relevant European Pharmacopoeia monographs for the required storage conditions of individual blood components for further fractionation and manufacturing of medicinal products derived from human plasma).

Methods of thawing

Frozen units should be handled with care since the bags may be brittle. The integrity of the pack should be verified before and after thawing to exclude any defects and leakages. Leaky containers must be discarded. The product should be thawed immediately after removal from storage in an appropriately controlled environment at + 37 °C and according to a validated procedure. After thawing of frozen plasma, the content should be inspected to ensure that no insoluble cryoprecipitate is visible.

The product should not be used if insoluble material is present. To preserve labile factors, plasma should be used as soon as possible after thawing. Post-thaw shelf life may be extended for a validated period to facilitate urgent transfusion for some indications. It should not be refrozen.

Thawing of the plasma is an inevitable part of some current viral inactivation processes, after which the products may be refrozen within the production process. In order to preserve component quality, the final component should be used as soon as possible following thawing for clinical use and not further refrozen.

Cryoprecipitation

The isolation of some plasma proteins, most importantly Factor VIII, vWF, fibronectin and fibrinogen, can be achieved by making use of their reduced solubility at low temperatures. In practice, this is done

by freezing units of plasma, thawing and then centrifuging them at low temperature.

Details regarding the freezing, thawing, and centrifugation conditions required for cryoprecipitate production are given in Standards, Chapter 5, Component monographs.

7. Open and closed systems and sterile connection devices

It is recommended that any new developments in component preparation involving an open system should be subjected to intensive testing during the developmental phase to ensure maintenance of sterility.

Blood components prepared by an open system should be used as quickly as possible.

Components prepared in systems using fully validated sterile connecting devices may be stored as if prepared in a closed system. Monitoring should be carried out by pressure testing of all connections and regular traction tests.

8. Irradiation of cellular blood components

Viable lymphocytes in blood components can cause fatal transfusion-associated graft versus host disease, particularly in severely immune-compromised patients, e.g. patients undergoing haematopoietic transplantation, children with inherited cellular immunodeficiency syndromes and some low birth weight neonates. Other clinical settings with an increased risk of this rare complication include intrauterine transfusion, transfusion between family members and transfusion of HLA-matched components.

Lymphocytes can be rendered non-viable by exposure to irradiation. Irradiation at doses specified in the Standards does not cause significant harm to other blood cells. Therefore, an irradiated component can be given safely to all patients. However, the *in vitro* quality of irradiated red cells deteriorates faster during storage than the quality of non-irradiated red cell components. Therefore, irradiation leads to a reduced shelf-life of red cell components (see Standards).

9. Prevention of CMV transmission

Cytomegalovirus (CMV) is a common infectious agent that can be transmitted via the transfusion of blood components. The risk of disease transmission is highest with fresh components containing mono- and poly-morphonuclear leucocytes. CMV infection is often asymptomatic in healthy persons. Antibodies usually appear 4 to 8 weeks after infection and can be demonstrated in standard screening tests. Since the infection is common, the test has to be repeated on each donation from a previously seronegative donor.

Infection caused by this virus is usually not clinically significant in immunocompetent recipients, but can cause severe, even fatal, disease in certain patients not previously exposed to the virus such as:

- transplant recipients;
- patients with severe immunodeficiency;
- foetuses (intrauterine transfusion);
- anti-CMV-negative pregnant women;
- low-weight premature infants and neonates.

These patients should receive components selected or processed to minimise the risk of CMV infectivity. The use of components from anti-CMV-negative donors or leucocyte-depleted components significantly reduces the risk of CMV transmission and CMV disease in immunocompromised patients. However, neither method nor a combination of them can completely prevent transmission due to occasional cases of CMV viraemia in the early stage of acute infection.

There is no consensus on the requirement for CMV screening in blood services that undertake universal leucocyte depletion of blood components. Some services (especially in areas that have a high seroprevalence of CMV) have ceased antibody screening, but others believe that the combination of antibody screening and leucocyte depletion may confer additional safety.

10. Pathogen reduction technologies

The aim of pathogen reduction technologies (PRT) is to remove or inactivate bacteria and/or other pathogens (viruses, parasites) using physical and/or chemical methods. So far, it has not been possible to treat whole blood donations, so blood must be separated into its component parts before use of PRT.

Systems for PRT of plasma components have been available and used routinely in Europe for many years. There are currently no licensed systems for PRT of red cells, but such systems are currently under development.

Several devices are CE-marked for PRT of platelets. These involve the addition of a photo-sensitising chemical to platelets in combination with exposure to UV light. An alternative system is being developed, which is based on exposure of platelets to UV light alone and does not require the addition of any chemicals.

Currently available systems have been demonstrated to inactivate a wide range of viruses, bacteria, parasites and leucocytes. They do not reduce infectivity associated with prion proteins and, hence, vCJD risk.

With regard to the efficacy of PRT of platelets, there is some loss of platelets in the process. However, this can be compensated for by an increase in collection volume for apheresis platelets or an increase in the number of buffy coats used in pooled platelets. Most clinical studies have demonstrated a reduced corrected count increment compared to untreated control platelets, and one study found an increase in bleeding risk associated with this phenomenon. Other studies have not shown a significant effect on clinical bleeding parameters. Other potential risks include toxicity and neo-antigen formation; neither has been observed in haemovigilance studies of short duration, but longer-term surveillance studies will be required to confirm the absence of long-term toxicity. One study has shown that PRT of platelets can be implemented in routine practice without impacting on utilisation of platelets or red blood cells or with a reduction of acute transfusion

reactions. PRT of platelets potentially allows the extension of the shelf-life of platelets to 7 days, with the consequent benefit in reducing platelet wastage. A further advantage of some PRT is inactivation of lymphocytes, which obviates the need for irradiation of platelets.

A symposium was held under the aegis of the EDQM/Council of Europe on implementation of PRT for blood components on 2 and 3 September 2010. A consensus on implementation did not emerge. It was noted that countries have adopted different positions with regard to the implementation of PRT depending on differences in the risk of pathogens. An executive summary including recommendations has been published online.[1] Several blood establishments in Europe have now implemented PRT of platelets, and others are actively considering it. The value and cost-effectiveness of implementation of these technologies should be assessed in conjunction with current and alternative methods for risk reduction (see section 12, Bacterial safety of blood components).

When using PRT, blood establishments should introduce quality-control measures to monitor the effectiveness of the process and, if applicable, removal of active substances and/or their metabolites.

11. Purity of components

Since blood components are used to correct a known deficit, each preparation process must be subjected to strict quality control. The aim is to produce 'pure' components, but a very high degree of purity can be difficult and expensive to obtain and might not even be necessary in all instances. However, it is absolutely necessary to declare the quality and to be able to make different types of preparations in order to give the clinicians a reasonable choice for patients with different transfusion demands.

For example, a red cell concentrate can be produced with varying concentrations of contaminating leucocytes and platelets. A buffy

1 www.edqm.eu/medias/fichiers/Executive_Summary_Pathogen_Reduction.pdf.

coat-depleted preparation (in which most of the leucocytes and platelets have been removed) is useful for most recipients because formation of micro-aggregates during storage is inhibited. If the prospective patient has antibodies against leucocyte antigens or if it can be foreseen that he/she will need a very large number of transfusions, leucocyte depletion will be more effective.

12. Bacterial safety of blood components

Overview

Although blood collection and processing procedures are intended to produce non-infectious blood components, bacterial contamination may still occur. Bacterial cultures of platelet components provide the best indication of the overall rate of contamination of whole blood donation provided that the sample for culture is obtained in a suitable volume and at a suitable time after collection. Surveillance studies have found rates of contamination as high as 0.4 % in single donor platelets, although rates at or below 0.2 % are more often reported. The causes of bacterial contamination include occult bacteraemia in the donor, inadequate or contaminated skin preparation at the phlebotomy site, coring of a skin plug by the phlebotomy needle and breaches of the closed system from equipment defects or mishandling. Platelet components are more likely than other blood components to be associated with sepsis due to their storage at room temperature, which facilitates bacterial growth.

A variety of procedures may be used to obtain a valid platelet sample for bacterial culture. Aseptic techniques are required in order to minimise the risk of false positive cultures due to contamination at the time of sampling or upon inoculation in culture. Additionally, it is prudent to retain a sample that can be used for repeat culture to validate a positive result. Large volume samples (8-16 mL) removed from a multiple-unit pooled platelet component or single donor apheresis platelets can be cultured any time post-collection. However, small volume samples (e.g. 2-5 mL removed from a single whole blood unit) should be taken for culture after a 24 to 48 hour delay post-collection.

Delayed sampling of a small volume permits bacterial growth to a level that subsequent assays can detect reliably, thereby overcoming sampling errors at low contamination levels.

PRT may offer an alternative approach to assuring the bacterial safety of blood components. Currently, systems are available for platelets, but not for red cells.

Quality control for aseptic collection and processing of blood components

- Data on routine bacterial monitoring should be analysed using statistical process control techniques to ensure that the process remains in control.
- Whenever the analysis indicates data that is outside of specified control limits then an investigation into potential causes of contamination should be undertaken and, where appropriate, collection and processing procedures should be revalidated.
- If routine bacterial monitoring of platelet components is not performed, e.g. when pathogen reduction of platelet components is in place, other methods for monitoring aseptic collection and processing should be considered.

Release as 'culture-negative to date' after bacteriological testing of all platelets

Sampling of platelets for the purpose of establishing a release criterion based on a negative result of bacterial cultures requires that the integrity of the closed system should be maintained. This is because platelets may continue to be stored for variable periods after sampling and before use. Suitable methods of sampling in this case include the use of integral satellite containers or the stripping, refilling and then pinching off of duplicate pigtails. Sampling may also be done into collection containers via the use of sterile connecting devices.

13. Storage of blood components

Storage conditions for blood components are designed to preserve optimal viability and functionality during the entire storage period. The risk of bacterial contamination decreases substantially if only closed separation and storage systems are used.

Equipment

- Blood components are stored at different temperatures (for example at + 20-24 °C, at + 2-6 °C or at different temperatures below 0 °C).

Whatever type of storage device is chosen, the following points should be considered before purchase:

- identification of user requirements, specifications and quality criteria;
- refrigerators and freezers must have surplus capacity;
- the space should be easy to inspect;
- the operation must be reliable and temperature distribution must be uniform within the unit;
- the equipment must have temperature recording and alarm devices;
- the equipment should be easy to clean and should withstand strong detergents;
- the equipment should also conform to local safety requirements.

Storage at + 2 to + 6 °C

The space for each of the component types should be clearly indicated. The temperature within the storage device should be continuously recorded. In large refrigerated rooms, two or more sensors should be used. These should be placed in the part of the refrigerator space which has been identified to represent the worst conditions.

The alarm system should preferably have both acoustic and optical signals and should be regularly tested.

Refrigerators for blood components should ideally be connected to a reserve power unit, as well as to the main supply.

There should be a system in place to maintain and control the storage of blood components throughout their shelf-life, including any transportation that may be required. Autologous blood and blood components should be stored separately. Temperature and hygienic conditions should be continuously monitored. Warning systems should be used where applicable.

Storage of frozen plasma components

Freezers with automatic defrosting should be avoided, unless it can be guaranteed that the low temperature is maintained during defrosting.

Freezers should ideally be connected to a reserve power source, as well as to the main supply.

Storage at + 20 to + 24 °C

Platelets are stored at + 20-24 °C. A closed device that permits temperature control is recommended. If such a device is unavailable, the storage location chosen should be capable of maintaining the required constant temperature.

Platelets should be stored in agitators that:

- enable satisfactory mixing in the bag, as well as gas exchange through the wall of the bag;
- avoid folding of the bags;
- have a set speed to avoid foaming.

Aspects of red cell preservation

The anticoagulant solutions used in blood collection have been developed to prevent coagulation and to permit storage of red cells for a certain period of time. Designed originally for storage of whole blood, they have also been used in blood from which components are prepared. All of the solutions contain sodium citrate, citric acid and

glucose, and some of them may also contain adenine, guanosine and phosphate.

Citrate binds calcium and prevents clotting of the blood. Glucose is used by red cells during storage. Each glucose molecule gives two molecules of adenosine triphosphate (ATP), which is formed by phosphorylation of adenosine diphosphate (ADP). ATP is an energy-rich molecule used to support the energy-demanding functions of red cells, such as membrane flexibility and certain transport functions in the cell membrane. During energy-consuming operations, ATP reverts back to ADP. Citric acid is added to anticoagulants to obtain a concentration of hydrogen ions that is suitably high at the beginning of storage at + 4 °C. Without the addition of citric acid, blood is too alkaline at storage temperature.

Acidity increases during storage, which reduces glycolysis. Conversely, the content of adenosine nucleotides (ATP, ADP, AMP) decreases during storage. By adding adenine, which is the main component of adenosine nucleotides, red cells can synthesise new AMP, ADP and ATP and compensate for (or reduce) this decrease. When red cell concentrates are prepared, a considerable part of the glucose and adenine is removed with the plasma. If not compensated for in other ways (e.g. by adding a larger amount than normal of adenine and glucose in the anticoagulant or by separate addition of a suspension/preservative medium), sufficient viability of the red cells can only be maintained if the cells are not over-concentrated. Therefore, normal CPD adenine red cell concentrate should not have an average haematocrit above 0.70. This also keeps the viscosity sufficiently low to permit transfusion of the concentrate without pre-administrative dilution.

Additive solutions

An additive solution should allow maintenance of red cell viability even if more than 90 % of the plasma is removed. The use of glucose and adenine is necessary for the maintenance of red blood cell post-transfusion viability. Phosphate may be used to enhance glycolysis and other substances (e.g. mannitol, citrate) may be used to prevent

in vitro haemolysis. Sodium chloride or disodium phosphate may be used to give the additive solution a suitable osmotic strength.

Micro-aggregates in blood components

Platelets and leucocytes rapidly lose their viability at + 4 °C. They form micro-aggregates that are present in considerable amounts even after 3-4 days of storage of whole blood and, even more so, in concentrates of red cells. Micro-aggregates can pass through the filters of ordinary blood transfusion sets. Micro-aggregates can cause decreased lung function by blocking lung capillaries and this may be of clinical importance in massive transfusions. Removal of platelets during component preparation reduces micro-aggregate formation. Likewise, leucocyte depletion by buffy coat removal also reduces the frequency of febrile transfusion reactions, and helps to achieve high-grade depletion of leucocytes if leucocyte-removal filters are used for this purpose.

Red cell preparations

The maximum duration of storage (expiry date) should be noted on each container. This duration may vary with the type of preparation (concentration of cells, formula of anticoagulant, use of additive solution) and should ensure a mean 24-hour post-transfusion survival of no less than 75 % of transfused red cells.

Red cells may be stored in a fluid state at a controlled temperature of + 2-6 °C. The performance of the storage refrigerator must be controlled very carefully.

Frozen red cells should be prepared and reconstituted according to an approved protocol, be stored at < – 60 °C, and produce satisfactory post-transfusion survival figures.

Platelet preparations

Platelets must be stored under conditions that guarantee that their viability and haemostatic activities are optimally preserved (see Standards).

Plastic bags intended for platelet storage should be sufficiently permeable to gases to guarantee oxygen availability to platelets and diffusion of carbon dioxide. The amount of oxygen required is dependent on the number of platelets and their concentration in the component. Lack of oxygen increases anaerobic glycolysis and lactic acid production. The quality of platelets is preserved if the pH remains consistently above 6.4 throughout the storage period.

Agitation of platelets during storage should be sufficient to guarantee oxygen availability but as gentle as possible to prevent induction of activation and storage lesions. The storage temperature should be + 20-24 °C. Platelets undergo membrane phase transition, and cold activation (below + 20 °C) means that the discoid structure of platelets gradually converts to a sphere.

Granulocyte preparations

Typically, granulocyte suspensions are prepared for a specific patient and administered immediately.

Plasma components could be stored at different temperatures

Recommended storage conditions for fresh frozen plasma and cryoprecipitate and for cryoprecipitate-depleted plasma are given in Table 4.2 below.

Table 4.2. Recommended storage conditions for fresh frozen plasma, cryoprecipitate and cryoprecipitate-depleted plasma

Blood component[a]	Length of storage and temperature[b]
Fresh frozen plasma, cryoprecipitate	36 months at or below – 25 °C
Cryoprecipitate-depleted plasma	3 months at – 18 °C to – 25 °C

a For plasma intended for fractionation, refer to the European Pharmacopoeia monograph *Human plasma for fractionation (0853)*.
b The recommended temperature ranges are based upon practical refrigeration conditions.

14. Transport of blood components

Blood components should be transported by a system that has been validated to maintain the integrity of the component over the proposed maximum time and extremes of ambient temperature of transport. It is recommended that some form of temperature indicator be used to monitor the temperature in transit. Also, the temperature on receipt can be monitored as follows:

- take two bags from the container;
- place a thermometer between the bags and fix them together with rubber bands;
- quickly place them back into the container and close the lid;
- read the temperature after 5 minutes.

Alternatively an electronic sensing device may be used to take immediate measurements from the surface of a pack.

On receipt, if not intended for immediate transfusion, the product should be transferred to storage under the recommended conditions.

Transport of standard red cell components

Red cell components should be kept between + 2 and + 6 °C. The temperature of red-cell bags should not go below + 1 °C nor exceed + 10 °C. A maximum transit time of 24 hours at temperatures not above 10 °C is recommended. Otherwise, transport conditions must be validated to ensure maintenance of the quality of red blood cells.

Transport of platelet components

Platelet components are usually not agitated during transport and, therefore, oxygen delivery to platelets is reduced. Agitation of platelets can be interrupted (simulated shipping conditions) for up to 30 hours for one to three periods without a major impact on the *in vitro* quality of the platelets at the end of a storage time of 5-7 days. The pH of the platelet components is better preserved when agitation is interrupted for several short periods compared to one long period.

Platelet components should be transported in an insulated container with temperature-stabilising elements that ensure transport temperature is maintained as close as possible to the recommended storage temperature. Transport conditions should be chosen to maintain component quality and must be validated for this purpose. It is recommended not to exceed 24 hours if transported without agitation. On receipt, unless intended for immediate therapeutic use, platelet components should be transferred to storage under the recommended conditions (including further agitation).

The impact of transport conditions on the quality of platelet components should be validated by quality control tests, e.g. swirling tests and pH measurements of components at the end of the storage period.

Transport of frozen plasma components

Frozen plasma components should be transported in the frozen state as close as possible to the recommended storage temperature.

15. Component information and principles of labelling

Brief information about the various blood components should be made available to clinicians with regard to composition, indications, and storage and transfusion practices. This includes the proviso that the blood must not be used for transfusion if there is abnormal haemolysis or any other deterioration, and that all blood components must be administered through a 150–230-μm filter (if not stated otherwise). This information should be presented to clinicians in a booklet and/or in an information leaflet on blood components.

The label on the component that is ready for distribution should contain the information (in eye-readable format) necessary for safe transfusion. That is: unique identity number (preferably consisting of a code for the blood-collection organisation, the year of donation, and a serial number); ABO and RhD blood group; name of the blood component; essential information about the properties and handling of the blood component; expiry date (see also labelling requirements in Chapter 5, Component monographs).

Chapter 5

Principles of blood component monographs

Monographs with detailed information on the different categories of blood components are given in the Standards section, Chapter 5, Component monographs.

- Part A – Whole blood components, page 279
- Part B – Red cell components, page 289
- Part C – Platelet components, page 325
- Part D – Plasma components, page 377
- Part E – White cell components, page 399

The blood components described in these monographs are to be regarded as standard blood components across Europe. However, some components are in use only in a few countries. Based on future consensus, the number of variant components may be reduced.

The component monographs have a standardised structure, which encompasses the headings as listed hereafter.

1. Definition and properties

Here, information is given about the component, including its origin, the active constituents and contaminating cells (if appropriate).

2. Preparation

Here, a short description is given about the method(s) of preparation. It differentiates between primary and secondary processing. Primary processing results in different blood components, each of which is described in Chapter 5 of the Standards section. Secondary processing leads to variant preparations, which are very similar to the primary component and do not differ in handling, release and application aspects. More detailed information about preparation processes is described in Chapter 4 of the Principles section.

3. Requirements and quality control

Typical component-specific handling and testing parameters for quality control are given in tables, which are formatted as follows:

Parameter to be checked	Requirements	Frequency of control

If appropriate, the requirements may be met by performing the test on the donation sample that was taken as part of the donor screening process in place of individual component testing.

The monographs provide advice on frequency of control. An alternative approach to identify the number of units to be tested is Statistical Process Control (SPC) (see Appendix 4).

Quality control may be carried out either as a separate quality control procedure for the given component or as a routine part of the issuance and transfusion of these components. Detailed information on the preparation processes are given in Chapter 4, Principles of blood component processing.

4. Storage and transport

Typical mandatory storage and transport conditions for the respective blood components are given. Detailed and descriptive information about the processes of storage and transport are given in Chapter 4, Principles of blood component processing.

5. Labelling

The labelling should comply with the relevant national legislation and international agreements. The given information should be shown on the label or contained in the component information leaflet.

6. Warnings

Typical warnings and side-effects are described that should be communicated to the physician (in written form, such as in a component information leaflet).

Chapter 6

Principles of blood components for intrauterine, neonatal and infant use

1. Overview

Specially designed blood components are required for intrauterine and infant transfusions. The following factors must be considered when transfusing neonates: (1) smaller blood volume, (2) reduced metabolic capacity, (3) higher haematocrit and (4) an immature immunological system. All these aspects are particularly important in foetal transfusions and for small premature infants.

There is a significant risk of transfusion-associated graft versus host disease (TA-GvHD) and Cytomegalovirus (CMV) transmission when a foetus or small infant is transfused. These patients should receive cellular components selected or processed to minimise the risk of CMV transmission.

The rate of transfusion should be controlled to avoid excessive fluctuations in blood volume.

Consideration should be given to producing red cell components for these patients from donors who have screened negative for haemoglobin S.

There are specific national regulations or guidelines for pre-transfusion blood grouping and compatibility testing of neonates.

2. Components for intrauterine transfusions

All components for intrauterine transfusion (IUT) must be irradiated. To minimise the effect of potassium load, red cells for IUT must be used within five days of donation and within 24 hours of irradiation.

Indications for use:

- intrauterine red cell transfusions are performed to treat severe foetal anaemia;
- intrauterine platelets are administered for the correction of severe thrombocytopenia, which may be due to antenatal HPA allo-immunisation.

3. Components for neonatal exchange transfusion

Exchange transfusion is a special type of massive transfusion. The components used must be fresh enough so that metabolic and haemostatic disturbances can be minimised.

A number of components can be utilised for exchange transfusion, including:

- whole blood (LD);
- whole blood (LD and plasma-reduced);
- red cells (LD and resuspended in fresh frozen plasma).

ABO and Rh groups, as well as other red cell antigens to which the mother has become sensitised, have to be taken into account when selecting blood for exchange transfusion.

Whole blood and red cell components for exchange transfusion should be irradiated, unless compelling clinical circumstances indicate that a delay would compromise the clinical outcome. Irradiation is essential if the infant has had a previous IUT.

To minimise the effect of potassium load, components of whole blood and red cells must be used within five days of donation and within 24

hours of irradiation. For reconstituted components, the shelf-life is 24 hours.

Indications for use:

- exchange transfusions of neonates;
- these components are also suitable for large volume (massive) transfusion of neonates and small infants;
- if the platelet count of the infant undergoing/following exchange or other massive transfusion is very low, specific platelet transfusion should be given.

4. Red cells for neonatal and infant small volume transfusion

Preterm infants are amongst the most intensively transfused of all hospital patients and have the greatest potential for long-term survival. Therefore, minimising the number of donor exposures is a central aim in designating proper components and guiding transfusion practice.

It is good practice to divide a component unit into several sub-batches and to dedicate all the satellite units from a donation to a single patient. Fresh blood and red cells are used in IUT and exchange transfusions. Hence, it is often thought that fresh blood is necessary for all neonatal transfusions. There is no scientific or clinical evidence to support this concept in the case of small volume, top-up transfusions, provided that transfusion rates are carefully controlled.

The component may be irradiated where clinically indicated.

Indications for use:

- anaemia of premature infants;
- to replace the blood loss due to investigative sampling;
- suitable for surgical replacement in infants and children.

5. Fresh frozen plasma for neonatal and infant use

In order to reduce donor exposure, a fresh frozen plasma (FFP) unit can be divided into approximately equal volumes in satellite packs, prior to freezing, by using a closed or functionally closed system. Three to four such bags can be dedicated to one patient.

ABO blood group-compatible plasma should be used. National requirements may require the use of plasma only from AB RhD-negative and -positive donors.

Indications for use:

- FFP may be used in coagulation defects, particularly in those clinical situations in which a multiple coagulation deficit exists and only where no suitable viral-inactivated alternative is available;
- congenital deficiency of single clotting factors where no virally-inactivated concentrate exists.

Contraindications:

- FFP should not be used simply to correct a volume deficit in babies in the absence of a coagulation defect nor as a source of immunoglobulins;
- FFP should not be used where a suitable virally-inactivated clotting factor concentrate is available;
- FFP should not be used in a patient with intolerance to plasma proteins.

6. Platelets for neonatal and infant use

When preparing platelets for infants, every effort should be made to minimise donor exposure.

Apheresis-derived platelets offer the greatest potential to reduce donor exposure and can be divided into satellite packs by using a closed system as for FFP.

The clinical situation of a small child may necessitate the use of volume-reduced platelets; volume reduction to around 25 mL causes about a 10 % loss of platelets.

The platelet component must be used within 24 hours of any washing procedure and within 6 hours of any concentration process. The platelet component should be irradiated where clinically indicated.

Indications for use:

- severe neonatal thrombocytopenia (of any cause).

Chapter 7

Principles of autologous transfusion

1. Overview

Several techniques of autologous transfusion, including pre-deposit autologous collection and intraoperative or postoperative red cell salvage, may be useful in surgery. These techniques have been designed to avoid the risks of the allo-immune complications of blood transfusion, and to reduce the risk of transfusion-associated infections. Each technique has its own separate risks that are also presented and discussed. The choice of different techniques (including allogeneic transfusion) should be balanced for the patient's benefit.

Pre-deposit autologous transfusion (PAT) comprises the collection, processing and storage of autologous blood components in the weeks preceding surgery for reinfusion in the perioperative period. In selected conditions, red cell or platelet components can be collected using a cell separator. The equivalent of 2 units of red cells or up to 3 standard adult doses of platelets can be collected in a single procedure. The incidence of severe adverse reactions and severe adverse events associated with the collection of whole blood has been shown to be significantly increased in autologous blood donors compared with allogeneic blood donors.

The principle of pre-deposit autologous collection and transfusion was popular in the 1990s among surgeons and patients because it was seen primarily as a means to reduce the risk of transfusion-transmitted infections. Since then, however, questions have been raised as to its benefits and its use remains controversial.

The frequency of some adverse effects of transfusion, such as bacterial contamination or administrative errors, is not reduced by PAT, and the latter may be increased. However, PAT may be the only option for a patient with an antibody for which compatible blood is difficult to find.

Some other disadvantages are that patients may be rendered anaemic prior to surgery, thus leading to increased transfusion requirements, blood wastage is increased (it is estimated that only 50 % of autologous blood is used) and cost effectiveness is low. In some circumstances, autologous blood may be inappropriately transfused, even if transfusion is not indicated. In addition, where efforts have been made to reduce the use of blood in surgery by improved pre-operative assessment and intraoperative management, the benefit of PAT has been reduced further.

Pre-deposit autologous blood components obtained from PAT must be collected, prepared and stored in the same conditions as for allogeneic donations. For these reasons, PAT must be collected by or under the control of blood establishments or in authorised clinical departments that are subject to the same rules and controls as blood establishments (see *Standards*).

Acute normovolemic haemodilution is the collection of blood immediately before surgery, with blood volume compensation (leading to a haematocrit below 0.32), with subsequent re-infusion during or after surgery.

Red cell salvage during surgery is another means of autologous transfusion. Blood collected from the operation site may be given back to the patient either after a simple filtration or a washing procedure. These techniques do not allow storage of the collected blood. They are

usually performed under the responsibility of anaesthesiologists and/or surgeons.

2. Pre-deposit autologous transfusion

Patient selection

Role of the physician in charge of the patient

In elective surgery, in which blood transfusion is expected, the physician in charge of the patient (usually the anaesthesiologist or surgeon) may prescribe preoperative collections.

The prescription should indicate:

- the diagnosis;
- the type and number of components required;
- the date and location of scheduled surgery.

The patient should be informed of the respective risks and constraints of autologous and allogeneic transfusion, and that allogeneic transfusion may also have to be used if necessary.

Indications

Autologous transfusion should only be considered if there is a clear reason for preferring PAT to allogeneic blood, and there is a strong likelihood that blood will be used.

Special consideration should be given to the use of PAT in exceptional circumstances, such as patients with rare blood groups where allogeneic blood is likely to be difficult to obtain.

Contraindications

Appropriate autologous pre-deposit collection may be carried out safely in elderly patients. However, more careful consideration may need to be given in the case of a patient aged more than 70 years.

Haemoglobin levels should be measured before each collection. Autologous pre-deposit collection should not be done in patients with a haemoglobin concentration below 100 g/L.

In patients with a haemoglobin concentration between 100 and 110 g/L, autologous pre-deposit collection may be discussed according to the number of scheduled collections and aetiology of anaemia.

The presence of cardiac disease is a relative contraindication, and the assessment of a cardiologist may be required. Patients with unstable angina, severe aortic stenosis or uncontrolled hypertension should not be considered.

Pre-deposit autologous transfusion in children

Pre-deposit autologous collection may be considered in children undergoing harvesting of bone marrow and in exceptional cases whereby suitable allogeneic blood is not available for elective surgery. The child should understand the nature of the procedure and be willing to co-operate.

Children under 10 kg should not be included in an autologous pre-deposit collection programme and PAT programme. For children between 10 and 20 kg, the use of volume compensation solutions is usually needed.

The maximum volume that can be drawn at each collection is 10 mL/kg or 12 % of the estimated blood volume.

The volume of anticoagulant in the pack should be adjusted as required to maintain an appropriate ratio of blood to anticoagulant. Paediatric packs of 200 mL or 250 mL (available with small gauge needles) should be used wherever possible.

Adverse reactions related to blood collection, such as haemodynamic disturbances, occur significantly more often in children. Volume replacement with crystalloid solutions reduces the rate of these adverse reactions.

Blood collection

Surgical admission and the day of the surgical procedure should be guaranteed. Sufficient time to enable optimal collection of blood should be allowed before surgery, but should not exceed the storage time of the collected blood component.

Sufficient time should be given from the date and time of the final blood collection prior to surgery for the patient to make a full circulatory and volaemic recovery. This should be at least 72 hours (preferably 7 days).

Iron and/or erythropoietin should be considered to supplement the patient's haemoglobin in conjunction with PAT.

For patients undergoing double-unit red cell apheresis, shorter collection intervals can be accepted at the discretion of the physician responsible for blood collection.

Preparation, storage and distribution of pre-deposit autologous components

See *Standards*.

Labelling

See *Standards*.

Storage

See *Standards*.

Records

Blood establishments and hospitals should both maintain the following records for every patient included in a pre-deposit autologous transfusion programme:

- the date and type of surgery;
- the name of the anaesthesiologist or the surgeon;
- the time of transfusion, specifying whether blood was used during surgery or postoperatively;
- the actual use of the prepared pre-operative autologous blood components;
- the concurrent use of perioperative autologous transfusion techniques;

- the technique used and volume of autologous blood re-infused;
- the use of allogeneic blood components;
- the occurrence of any adverse reactions.

Audit
Blood establishments should audit the use of PAT, where it is provided on a regular basis.

3. Red cell salvage
Cell salvage (CS) is the process of collecting a patient's blood during surgery for transfusion back into the patient. It is also known as 'autotransfusion' and covers a range of techniques that scavenge blood from operative fields or wound sites and re-infuse the blood back into the patient. CS can be performed during intraoperative and/or postoperative periods. The aim of CS is to reduce or eliminate the need for allogeneic blood transfusion. At least one allogeneic packed red cell should be saved. The blood salvage system can, in general, be broken down into a collection and a processing system.

Collection system
The collection system consists of:

- the suction line and suction tip used in the surgical field;
- suction;
- an anticoagulant;
- collection reservoir.

During collection of red blood cells, an appropriate anticoagulant is added to salvaged blood. Then, anticoagulated blood is filtered and collected in a reservoir. When a sufficient amount of blood has been collected (approximately the equivalent of one packed red blood cell should be the result at the end of the entire process), separation of blood by centrifugation and washing of red blood cells follows.

Processing system

Various separation devices use centrifuge bowls for stepwise processing or a disc-shaped separation chamber enabling continuous processing of salvaged red cells. The washing procedure removes (to a large extent) free haemoglobin, plasma, platelets, white blood cells, and anticoagulant. Remaining red blood cells are then resuspended in normal (0.9 %) saline. The resulting haematocrit should be 0.60 and 0.80. Small washing volumes, fast washing rates, and half-full bowls should be avoided. Salvaged red cells should be transfused immediately or at least within 6 hours. Blood filters and standard blood-administration filters are required. Some manufacturers recommend micro-aggregate or leucodepletion filters to remove bacteria, cancer cells or amniotic-fluid contaminants depending on the different clinical settings.

Indications for the use of cell salvage

- patients undergoing cardiothoracic, vascular, transplant and major orthopaedic surgery;
- anticipated blood loss of 1000 mL or of 20 % estimated blood volume;
- patients with low haemoglobin levels or at an increased risk of bleeding;
- patients with multiple antibodies or rare blood types;
- patients with objections to receiving allogeneic blood.

Parameters for quality control

Parameters for quality control of the component should be:

- volume;
- haematocrit;
- haemolysis at the end of the process;
- protein content of the supernatant.

Precautions

Some substances should not be aspirated with blood: antibiotics not licensed for intravenous use; iodine; hydrogen peroxide; alcohol; topical clotting factors; orthopaedic cement; sterile water.

Careful use of a large-bore suction tip under low vacuum pressure can reduce the risk of shear-induced haemolysis.

Colorectal surgery: Salvaged blood can (under special preventive measures) be gained during colorectal surgery or other types of surgery if the blood has come into contact with bacteria. Use of leucodepletion filters and washing of salvaged blood reduces the risk of microbial contamination because these methods also help to minimise the risk of activation of coagulation factors or influx of cytokines and other biologically active substances. As an additional precaution, broad-spectrum antibiotics should be administered to the patient.

Haemorrhage in cancer patients: although the passing of blood through a leucodepletion filter significantly reduces the number of retransfused tumour cells, the salvaged cells should be irradiated.

Obstetric haemorrhage: use of leucodepletion filters in obstetric haemorrhage provides a significant reduction in contamination of cells from amniotic fluid. This is also true for caesarean section. There is also concern regarding reinfusion of foetal red cells from the operative field. If the mother is RhD-negative and the foetus RhD-positive, the extent of maternal exposure should be determined as soon as possible, and a suitable dose of human anti-D immunoglobulin should be administered.

Chapter 8

Principles of immunohaematology

1. Overview

The main purpose of immunohaematology testing is to ensure that a compatible blood component is issued to the right patient. It is therefore essential to obtain accurate results when undertaking blood grouping, and antibody screening on donors and patients and compatibility testing.

Errors at any stage of the performance of such tests can lead to incompatible or incorrect blood components being transfused, with significant adverse health effects in patients. These can be due to inadequate training or procedures and/or human error resulting in misidentification of samples from donors or patients, technical failures in testing or misinterpretation of results, and transcription errors. Haemovigilance data indicate that, in many cases, a combination of errors lead to patient harm with the original error being perpetuated or compounded by the lack of adequate procedural controls within the laboratory or at the bedside.

The implementation of a quality system helps to reduce the number of errors made in laboratories. Elements of the quality system include the use of standard operating procedures, staff training, periodic assessment of the technical competence of staff, documentation and

validation of techniques, reagents and equipment, procedures that monitor day-to-day reproducibility of test results and methods to detect errors in analytical procedures.

2. Immunohaematological testing

Immunohaematological testing includes blood group determination, antibody screening, antibody identification as applicable and compatibility testing. All tests could be manual or automated and performed by serologic or molecular methods as appropriate.

Blood group testing

Serological testing

Serological blood group testing includes ABO/RhD typing and additional phenotyping. This is currently the standard technique used in most transfusion laboratories.

Molecular testing

Molecular testing is becoming increasingly available and used as a supplemental technique to serological testing. In time, molecular testing may replace the need for routine serological testing. Current indications for molecular typing include situations: in which serological testing of blood donors and patients renders unclear results; if there is a suspicion of weak antigens or variants (within *ABO*, *RHD*, *RHCE*, *JK*, *FY*); if serological reagents directed to specific antigens do not exist or are not readily available.

Increased utilisation and access to this technology will support further indications:

- multi-transfused patients that cannot be typed with serological techniques who may benefit from matched erythrocyte transfusions, for example, patients with sickle cell anaemia, thalassaemia and others who depend on treatment by chronic transfusion;

- patients that cannot be typed with indirect agglutination techniques because of positive direct agglutination tests (DAT pos);
- patients that may be expected to present with unusual and unexpected phenotypes/genotypes;
- blood donors with unusual phenotypes in serological typing;
- donors of panel cells if extended typing is needed or if heterozygosity or homozygosity needs to be established;
- foetal *RHD*, *RHCE*, *KEL*, *FY*, *JK* or other clinically significant genotypes (in samples of maternal blood, amniocentesis or chorionic villi) in cases of maternal immunisation against the corresponding antigens.

Testing can be undertaken on samples from blood, amniocentesis, biopsy of chorionic villi, and plasma.

Available test methods include, for example: polymerase chain reaction-restriction fragment length polymorphism (PCR-RFLP), sequence-specific priming (PCR-SSP), allele-specific PCR (AS-PCR), allelic discrimination (AD), real-time PCR and sequence-based typing. Commercial reagents and kits are available for these techniques. Micro-array and mass spectrometry-based techniques are also available.

Molecular investigations may be carried out at regional, national or international laboratories. Before ordering such typing, information on how to handle and ship samples, material or prepared DNA should be requested.

3. Antibody screening and identification

Antibody screening is performed to detect clinically significant non-ABO red cell antibodies. Testing is usually performed using an anti-human globulin technique.

In addition donors may be investigated for high titres of anti-A and/or anti-B to avoid transfusing plasma-containing blood components (FFP, platelets in plasma) with a high titre of these antibodies.

Antibody screening, identification and quantitation may also be performed in pregnant women to evaluate the risk of haemolytic disease of the foetus and newborn.

Most often, red cell antibody quantitation is confined to the quantitation of anti-RhD. It is recommended that quantitation of anti-RhD be carried out by automated techniques rather than by manual titration. If an automated technique is not available, manual titration by the anti-globulin test is recommended.

Pre-transfusion testing

The purpose of pre-transfusion testing is to select compatible blood components that will survive normally in the circulation, and to avoid clinically significant haemolysis of red blood cells during or after transfusion.

ABO/RhD typing of donor and recipient is the most important test.

Antibody screening is performed to detect clinically significant non-ABO red cell antibodies. Positive results of screening tests should be investigated fully to identify antibody specificity. If appropriate, red cell components lacking antigens should be selected for transfusion.

A compatibility test should be undertaken before issuing red cell components for transfusion. This process may involve serological testing or may be achieved with electronic release of the component using a type and screen procedure. The appropriate method for compatibility testing will be determined by the results of screening of blood groups and antibodies on the current sample as well as results of previous testing and clinical urgency of the transfusion.

Serological compatibility testing

The principle of serological compatibility testing is to test donor red cells with the recipient's plasma/serum. In patients with clinically significant red cell antibodies, serologic crossmatch must

be performed using an indirect anti-globulin test. When clinically significant antibodies are absent, an immediate spin crossmatch may be performed immediately before issue of the red cell component. However, immediate spin crossmatch is effective only for excluding ABO incompatibility.

Electronic issue following a type and screen procedure

Electronic issue (EI) should be employed only when a test for clinically significant antibodies is negative and there is no history of clinically significant antibodies in the patient.

The overall process for determining eligibility for EI must be controlled. Supplementary checks (e.g. review of the clinical details on the request form) should be performed. It should not be possible to issue ABO-incompatible red cells. The computer system should have a record of the history of transfusion and antibodies of the patient as well as serological status of the current sample.

The following process criteria should be met:

- samples and reagents are registered and identified within the analyser via barcodes;
- entry of the testing and results of the blood group and antibody screening are fully automated;
- results are transmitted electronically from the analyser to the computer;
- the computer controls the suitability of patients and their samples for EI;
- the computer enables permanent exclusions of patients from EI in the presence of antibodies of likely clinical significance;
- the computer enables temporary exclusions of patients from EI (e.g. limited period of exclusion after transplantation of solid organs);

- stock entry of unique donation number, component code, blood group and expiry date from the unit's label is by barcode reader or other electronic means.

The following criteria for patient and sample should be met:

- interpretation of blood group in the current sample is identical to the historical record;
- no manual amendments have been made to automated results;
- the current antibody screening is negative;
- results of the patient's group and antibody screening are complete and fully authorised in the computer;
- the patient does not have a previously known antibody of likely clinical significance;
- the current sample meets the requirements for timing and storage.

Quality control

The quality control procedures for immunohaematology can be divided into internal and external control programmes. They must be implemented for reagents, techniques, methods and equipment used.

Internal quality control

Quality control of reagents and techniques

Quality control procedures recommended are applied to the reagents used for manual and automated techniques. However, reagents for automated instruments are generally specific for that instrument. Each new lot must be tested for control against specifications.

For antigen testing, quality controls should include positive, preferably heterozygous, and negative controls. For antibody testing a positive, preferably weak, control is included.

The controls should be carried out with each test series or at least once a day provided the same reagents are used throughout.

Quality control of equipment

Equipment used (in particular centrifuges, automatic cell washers, incubators, refrigerators and freezers) should undergo regular quality controls. Equipment for automated blood grouping should also be systematically controlled according to the manufacturer's instructions.

External quality assurance (proficiency testing)

In external quality assurance, blood samples for proficiency tests coded as 'normal' and 'problem' are distributed from a national or regional laboratory to the participants, at least twice a year. The exercise can be limited to compatibility testing, since ABO-typing, RhD-typing and additional phenotyping, as well as alloantibody detection, are automatically included. The proficiency test panel may consist of multiple blood samples and the participants are asked to test each red cell sample against each plasma/serum for compatibility. The test panel should be composed in such a way that compatible as well as incompatible combinations occur. A titration of one or two of the detected antibodies may also be requested as part of the proficiency test.

The results are collated and accuracy scores are determined. These should be communicated to all participating laboratories (in coded or uncoded form, according to local agreements) in order to enable each laboratory to compare its own quality standards with those of a large number of other laboratories.

If no proficiency programme is available in a particular geographical area, the laboratory should arrange mutual proficiency testing with another laboratory. Although such external quality control is not as informative as participation in a comprehensive proficiency-testing programme, it is a valuable addition to the internal quality control procedure.

Chapter 9

Principles of screening for markers of infection

1. Overview

Quality assurance for screening tests for infectious markers is particularly important and implies both general and specific approaches.

Only tests that have been licensed or evaluated and considered suitable by the relevant regulatory authorities can be used. In the EU, these tests are considered as *in vitro* diagnostic devices and must be CE marked. EU Directive 98/79/EC classifies the HIV, HTLV, hepatitis B and C screening tests in list A, Appendix II. The manufacturer must have a full quality system, certified by an authorised body, and must submit batch release certificates containing all the control results for each lot.

In addition, proper validation demonstrates control, generates useful knowledge of the test and establishes future requirements for internal quality control, external quality assurance, calibration and maintenance of equipment and training of personnel, etc.

There must be special emphasis on training of staff, assessment of staff competency, maintenance and calibration of equipment, as well as the

monitoring of the storage conditions of test materials and reagents, together with documentation of all of these actions.

Current tests for the screening of donations are based on the detection of relevant antigens and/or antibodies and viral genomes.

It is further recommended that the tests include an external weak positive control in order to allow for statistical process monitoring.

Ideally, confirmatory tests should be as sensitive as, and more specific than, those used for screening. However, some screening tests are more sensitive than the available confirmatory tests. It is recommended that confirmatory algorithms be developed nationally to enable the appropriate and consistent investigation and resolution of screen reactivity.

2. Algorithm for infectious marker screening and confirmatory testing

Figure 9.1 shows a widely used algorithm for infectious marker screening and confirmatory testing.

Figure 9.1. Algorithm for infectious marker screening and confirmatory testing

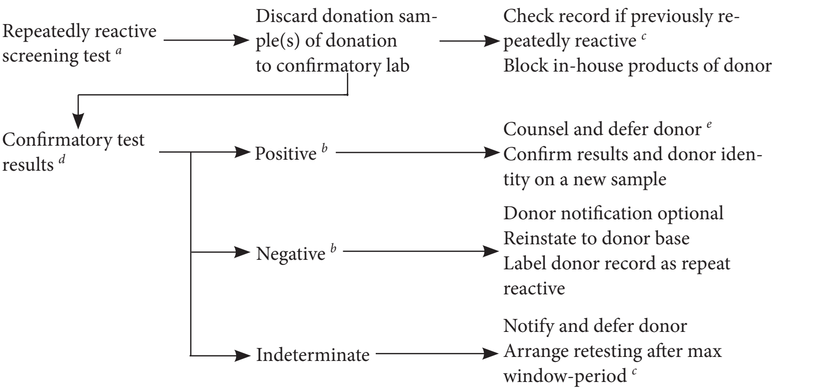

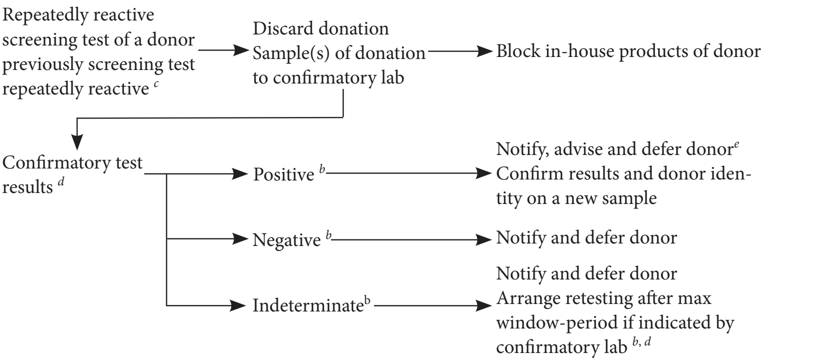

a For example, a repeatedly reactive serological screening test or a positive NAT on a single donation. Confirmatory testing is performed by a certified or accredited medical microbiology reference laboratory, which is responsible for results and may use tests at its discretion. The confirmatory laboratory should be kept informed about the type of screening test used by the blood establishment, and is contracted to use tests at least as sensitive as the screening test and, if feasible, based on other principles.

b The confirmatory laboratory is contracted to provide overall confirmatory test results or interpretations as follows: 'positive', which means infected; 'negative', which means not infected; or 'indeterminate', which means a diagnosis cannot be established (may include a demand for follow-up testing). If a confirmatory test(s) is less sensitive than the screening assay, the conclusion of confirmatory testing should read 'uncertain' (unless positive).

c The blood establishment keeps a donor record allowing longitudinal recording of confirmatory laboratory test results as: screening test positive; confirmatory lab positive; negative; or indeterminate.

d The confirmatory laboratory is contracted to keep longitudinal records of the unique donor ID, linked to laboratory test results.

e Refer donor to a medical doctor (general practitioner or specialist). Inform plasma fractionation centre(s) if plasma from earlier donation(s) has been issued. Inform hospital(s) to allow look back if component(s) from earlier donation(s) have been issued.

The specific approach to the quality of the screening process must rely on the following categories of measures:

- internal day-to-day quality control covering both reagents and techniques. Batch pre-acceptance testing (BPAT) of new manufacturer's lots of kits should be performed as an additional measure of quality assurance;
- external quality checks. In particular, confirmation of screen reactivity should be carried out by an appropriate and certified reference laboratory;
- occasional internal exercises as part of process qualification at implementation and/or after a significant process change using

a panel of sera that have been established by comparison with available standards;

- external proficiency exercises involving the testing of a panel of sera circulated to laboratories by an approved or otherwise competent expert institution;
- collection of representative data may be useful to monitor test performance.

It is recommended that repeatedly reactive rates and confirmed positive results of screening for infectious markers and epidemiological data be collected and monitored at least on a national level. This will allow international comparisons to be made.

It should be noted that following hepatitis B immunisation, a transient positive HBsAg result may be obtained and this can be identified by follow-up HBsAg testing of the donor.

3. Confirmatory testing

Anti-HIV-1/2, anti-HCV and HBsAg

The approaches currently used to confirm HIV or HCV infection consist of the use of a nationally established algorithm, which may include alternative immunoassay (IA), western blot or recombinant immuno-blots. Tests for antigens and the use of nucleic acid amplification techniques (NAT) may be of value in the interpretation of uncertain antibody test results. The positive confirmatory test should be repeated on an additional sample taken as soon as possible and not later than 4 weeks after the first sample. External referral of the donor could be used for this repeat sample.

Confirmation of HBsAg reactivity should include specific neutralisation. The stage of infection of the donor may be determined by anti-HBc (total and IgM-specific) and HBe antigen/antibody (HBeAg/anti-HBe).

Anti-HTLV-I-II

The approach to anti-HTLV-I-II confirmation testing is similar to that of HIV and involves nationally established algorithms as well as specific assays including immuno-blotting and NAT. Sensitive tests for genome detection (including typing) may be helpful in defining the infection status of the donor. NAT detection of chromosomal integrated proviral HTLV (e.g. in whole blood or PBMCs) may be more sensitive compared with plasma test material.

4. Nucleic acid screening

The Committee for Medicinal Products for Human Use (CHMP) has recommended a strategy of pre-testing by manufacturers of mini-pools (of donations or of samples representative of donations) for HCV in order to avoid the loss of a complete manufacturing pool and to facilitate trace-back to the donor in the event of a positive test result.

In order to achieve a sensitivity that detects 5 000 IU/mL of HCV RNA for donations tested in mini-pools of, for example 100 donations, 50 IU/mL should be detected with 95 per cent confidence by the NAT assay. Validated enrichment steps like centrifugation may counteract the dilution effect of mini-pools. Each assay run should include an external run control (usually at 3 times the 95 per cent detection limit). This reagent must be reactive in every run. The external run control may be omitted if the test is licensed (CE marked) and there are other procedures that ensure robustness.

5. Additional screening

Syphilis screening

There is ongoing discussion over the need to test blood donors for syphilis, but the test may be used as an indicator of risk behaviours for sexually transmitted diseases and is still required by most European countries. Most blood establishments use a Treponemal antibody test employing a variant of the Treponema pallidum particle agglutination assay (TPPA) or another IA. Reactive syphilis screening results should

ideally be confirmed by an appropriate Treponemal antibody test including; TPPA, fluorescent treponemal antibody test (FTA) or an immunoblot test. This test is not required for plasma for fractionation.

Anti-HBc screening

Donors or donations may be tested by an approved test that will detect antibodies to hepatitis B core antigen (anti-HBc). The approach to deferral or re-entry of an anti-HBc positive donor should be established in an algorithm.

Re-entry into the donor base of an anti-HBc positive donor and the subsequent release of his/her donations should only be considered when the donor has been shown to have anti-HBs with a titre of at least 100 IU/L and each subsequent donation should test negative for both HBsAg and HBV DNA using approved assays.

CMV screening

Testing for CMV antibodies is most commonly performed using an IA or the latex particle agglutination test. The screening of donations for anti-CMV enables the establishment of a panel of anti-CMV negative components for dedicated use in highly susceptible patients. This test is not required for plasma for fractionation.

Malaria screening

At present, only a few reliable and robust malaria antibody tests are commercially available. Any malarial antibody-testing requirement necessitates integration within local approaches to the taking of donor histories. If malaria antibody testing is used to determine donor acceptance or rejection, the test employed should be shown to detect antibodies to the malaria types that are likely to pose a risk of transfusion transmission. Currently, NAT for malaria cannot be recommended for use in screening of blood donors because it may fail to detect the small number of parasites in a blood donation that can infect a transfusion recipient. Confirmation of reactivity in malaria antibody tests should be performed by a competent and certified reference laboratory that can define the malaria status of the donor. Users

need to be aware that assays may depend on the detection of heterotypic antibodies. Users must ensure that the assay detects antibodies to the Plasmodium species prevalent in their donor panel. This test is not required for plasma for fractionation.

Trypanosoma cruzi screening

Donors who were born or have been transfused in areas where trypanosomiasis is endemic can be selected to be tested for antibodies against *T. cruzi*. This test is not required for plasma for fractionation.

NAT screening

Selective testing of donations might also be used to protect specific vulnerable recipient populations at increased risk of a particular infection e.g. hepatitis E virus or parvovirus B19.

Chapter 10

Principles of haemovigilance

1. Overview

Haemovigilance is defined as the use of organised surveillance procedures related to adverse events or adverse reactions either in donors or in recipients as well as the epidemiological follow-up of infectious disease markers in donors.

The ultimate goal of haemovigilance is to prevent the occurrence and recurrence of adverse events and reactions. For that purpose, results of data analyses should be fed back to their providers periodically and communicated to the Competent Authority (or Regulatory Authority) indicating (if possible) preventive or corrective measures to be adopted.

Haemovigilance should also incorporate an early alert/warning system. Haemovigilance provides useful information on morbidity arising from donations and transfusions of blood, and gives guidance on corrective measures to prevent recurrence of certain incidents. Moreover, haemovigilance is considered to be a part of total health-care vigilance (along with e.g. pharmacovigilance and vigilance on medical devices).

The information provided by haemovigilance contributes to improving the safety of blood collection and transfusion by:

- providing the medical community with a reliable source of information about adverse events and reactions associated with blood collection and transfusion;
- indicating corrective measures required to prevent the recurrence of some incidents or mistakes in the transfusion process;
- warning hospitals and blood establishments about adverse events and reactions that could involve more individuals than a single recipient, including:
  – those related to the transmission of infectious diseases;
  – those related to blood bags, solutions or blood processing.

2. Prerequisites for implementation of a haemovigilance network

Haemovigilance should be a responsibility of the national Competent Authority (or Regulatory Authority) for blood safety. Haemovigilance networks should embody operational linkages between clinical departments, hospital blood banks, blood establishments and national authorities.

Traceability of blood components

Traceability, which is a prerequisite for haemovigilance, may be defined as the ability to trace in all directions each individual unit of blood or the blood components derived from it from the donor to its final destination, whether this is a patient, a manufacturer of medicinal products or its disposal.

The essential element for traceability is a unique identification numeric or alphanumeric code for each donation, with a subsidiary code for each component prepared from that donation (Recommendation No. R (96) 11 of the Council of Europe on documentation and record-keeping to guarantee the traceability of blood and blood

components especially in hospital). This unique identifier must be linked to data that identifies both the donor and the recipient. In this way, all patients transfused with a particular donor's blood (or all donors who donated the blood components that a patient received) may be traced.

Traceability is essential for:

- tracing, retrospectively, a possibly infectious donor in case of transmission of an infectious agent to a recipient;
- tracing, retrospectively, a possibly infected recipient in case of infectivity of a donor;
- tracing recipients in case of systemic problems that put recipients at risk of serious adverse reactions or events.

Traceability can provide information on the total number of:

- patients that have been transfused;
- blood units or components that have been issued or used;
- blood donors that have provided the transfused blood units or components.

Without this information, it is difficult to calculate the incidence of adverse events and reactions and, thus, to estimate risk. The number of adverse events and adverse reactions over a given time period may help to identify critical issues within the various processes of collection, preparation and/or transfusion.

Information systems should be available to facilitate rapid traceability by using patients, blood components and donors as data-access keys. To ensure the reliability of the database, confirmation is needed that the blood component was transfused to the designated patient for whom it was issued. Without this, proving the link between donor and patient requires verification in the patient's notes that the blood component was appropriately transfused. The document confirming the transfusion should also include information on the existence or non-existence of immediate adverse events or reactions.

The following accurate data should be made available:

- personal data uniquely identifying the donor and providing a means to contact him/her;
- the blood establishment in which the blood or blood component collection has been carried out;
- the date of donation;
- the blood components produced and additional component information, if appropriate;
- the blood establishment or hospital blood bank to which the blood component has been distributed, if different from the production facility;
- the hospital and the ward to which the blood component has been issued for transfusion;
- the date and time of issue;
- the final fate of the unit; either the identity of the patient who received it or other use (e.g. quality assurance, reagents, discards, etc.);
- the date and starting time of transfusion.

When blood components have not been issued for transfusion, data should be available to identify the facility where the units have been used or disposed of.

Co-operation between blood establishments, hospital blood banks and clinical departments

The responsibility of reporting adverse events and reactions does not imply a responsibility for individual patient care.

Reporting and analysis of adverse events and reactions associated with transfusion requires close co-operation between the clinical department where transfusion took place, the hospital blood bank that issued the transfused blood component and the blood establishment that collected and distributed the blood unit (if different from the hospital blood bank).

This co-operation is essential to ensure a complete investigation of any adverse event or reaction, including uneventful transfusion errors. In the blood establishment and/or the hospital blood bank, the physician involved may be the person responsible for blood component delivery, or a physician may be specifically in charge of haemovigilance. Similarly, in clinical departments, the responsible person can be the physician in charge of the patient or another physician specifically in charge of haemovigilance.

In case of serious adverse reactions in blood recipients, which may be related to the transfused blood components, notification should be sent as soon as possible to the blood establishment where the components were collected.

Prompt reporting enables the blood establishment to take action to block blood components from related donors, donations or production methods.

Serious adverse reactions include: acute haemolytic transfusion reactions, sepsis due to bacterial contamination, delayed haemolysis, transfusion-related acute lung injury (TRALI), transfusion-associated graft versus host disease (TA-GvHD), transfusion-transmitted infectious diseases, anaphylaxis, transfusion-associated circulatory overload (TACO), hypocalcaemia, hypothermia, post-transfusion purpura (PTP).

3. Types of adverse reactions and adverse events collected in a haemovigilance network

Adverse reactions in patients

Adverse reactions associated with transfusion of blood components are the primary scope of a haemovigilance system, which should collect reports concerning patients of events such as:

- immediate adverse reactions during transfusion, such as: haemolysis; non-haemolytic febrile transfusion reaction; rash; erythema; urticarial; anaphylactic shock; bacterial contamination;

- TRALI, TACO and TA-GvHD;
- delayed adverse reactions after transfusion, such as haemolysis, PTP, iron overload, etc.;
- bacterial, viral, parasitical or TSE transmission;
- occurrence of allo-immunisation against red cell, HLA or platelet antigens.

The rules for reporting may differ according to the type and severity of adverse reaction. In case of minor reactions (such as non-haemolytic febrile transfusion reaction, rash, erythema and urticaria), individual reports should only be sent by the clinical departments to the blood bank. Then, depending on the organisation of the haemovigilance network, the blood bank may send periodic reports to its associated blood establishment or to the Competent Authority (Regulatory Authority) describing the incidence of such events.

This applies to any event that may involve several individuals and to serious hazards. Moreover, in case of viral transmission, the extent of required investigations should be clearly defined.

Adverse reactions in donors

See also Chapter 3, Principles of blood collection.

Adverse reactions in donors are defined as an unintended response associated with the collection of blood or blood components.

Adverse reactions in a donor should be fully documented in donor records and serious adverse reactions should also be documented in the records of the quality system.

Haemovigilance for donors may:

- facilitate the creation of a list of adverse events and reactions in relation to blood collection;
- allow analysis of data and enhance safety of blood collection by implementing corrective actions to prevent recurrences of incidents;

- allow analysis of data and improve transfusion safety depending on donor selection (frequency and causes of blood donation exclusion) and epidemiological follow-up of the donor population (confirmed positive donors in infectious marker screening);
- allow tracing of donors in case of an emerging threat to the safety of blood components (such as a new disease endemic situation).

Adverse events related to blood donation can occur in several fields:

- donor selection: the donor does not fulfil the local medical selection criteria, but has been given clearance for donating blood (with a possible consequence for his/her health or quality of blood components), e.g. insufficient haemoglobin level before donation, insufficient weight, etc.;
- blood collection: inappropriate procedure, e.g. excessive blood loss during blood or blood component donation, inadequate volume of anti-coagulant used for apheresis procedures;
- donor suitability: post-donation information may have consequences for the safety of donated blood components.

Both adverse events and reactions in donors may also have consequences for the quality of the donated blood components.

Data concerning adverse reactions and adverse events in donors should be collected and evaluated within blood establishments and, if appropriate, should be reported at least annually to the national haemovigilance system. Information on adverse events and adverse reactions in donors should be considered to be part of the haemovigilance system.

Adverse events

An adverse event is defined as any untoward occurrence associated with the collecting, testing, processing, storage and distribution of blood and blood components that might lead to an adverse reaction in blood recipients or blood donors.

Serious adverse events are those that might have led to death or life-threatening, disabling or incapacitating conditions for patients or donors (but did not), or which might have resulted in prolonged hospitalisation or morbidity (but did not). Examples of such serious adverse events are: failures to detect an infectious agent; errors in ABO typing; incorrect labelling of blood samples or blood components from donors. For instance, if components were not transfused or an incorrect or inappropriate component was administered due to incorrect identification of the recipient, Directive EC 2002/98 requires that these events must be notified to the Competent Authority.

'Near-miss' events are a subgroup of adverse events. A near-miss event is defined as any error which, if undetected, could result in determination of a wrong blood group or failure to detect a red cell antibody or the issuance, collection or administration of an incorrect, inappropriate or unsuitable component, but where the mistake was recognised before transfusion took place.

Adverse events include incorrect, inappropriate or unsuitable blood component transfusions that did not lead to harm to the recipient (but could have). For example, administration of a mismatched ABO-compatible component or failure to give irradiated components when prescribed.

Notification of adverse events which are transfusion errors that do not cause an adverse reaction may help to identify weaknesses in the clinical transfusion process and thereby reduce risk. The haemovigilance system should inform relevant staff of the importance of reporting of adverse events. The haemovigilance system should provide a system for reporting near misses with anonymisation to protect individuals from blame and to stimulate voluntary reporting.

Information technology systems may facilitate reporting and analysis of haemovigilance data.

Device defects

Reporting of device defects can be viewed as part of haemovigilance (see *Standards*).

4. Tracing and recall of potentially infectious donations for HIV, HCV or HBV (look-back)

Post-transfusion infection in a recipient reported to the blood establishment

Hospitals must inform the blood establishment whenever a recipient of blood components develops laboratory test results and/or disease symptoms indicating that a blood component may have transmitted an infectious agent (see *Standards*).

It is important that the blood establishment be informed without delay by the hospital so as to allow further action on implicated donations and donors and in order to prevent harm to other recipients.

Test results from donations of implicated donors may be re-analysed, or additional or confirmatory tests on archived or freshly obtained samples from the implicated donors may be performed with the aim of excluding infection by HIV, HCV or HBV in the donor(s). If such analyses reasonably exclude infection, such donor(s) may be re-released for future donations and (temporarily) blocked components derived from their donations may be re-released.

Where feasible and appropriate, the blood establishment should (temporarily) defer all implicated donors from further donations and retrieve (temporarily) or quarantine all in-date components for transfusion collected from the implicated donors.

If an implicated donor is found with a confirmed positive test for infection by HIV, HCV or HBV, the blood establishment should act accordingly with regard to deferral of the donor and initiate a look-back procedure on previous potentially infectious donations and inform the hospital(s) concerned.

The incident should be reported to the national haemovigilance system and/or Competent Authorities (or Regulatory Authorities).

Post-donation information

The blood establishment should (temporarily) block all in-house components from the donor and retrieve all in-date components. The relevant plasma fractionation institute must be notified.

The blood establishment should perform a risk analysis to assess whether the incident indicates a potentially infectious blood component destined for recipient(s). Test results from donations of the implicated donors may be re-analysed, or additional or confirmatory tests on archived or freshly obtained samples from the donor may be performed.

If a confirmed HBV, HCV or HIV infection is shown in the donor, the blood establishment should defer the donor and undertake a look-back procedure on previous potentially infectious donations.

Recall of blood components

In the case of a quality deviation (e.g. for HBV, HCV or HIV), the blood establishment must retrieve all in-date blood components distributed to the hospital(s) as a precautionary measure. This procedure may be a temporary measure and implies that certain retrieved blood components may be re-released after appropriate risk analyses and/or additional testing. The measure is taken in order to prevent harm to potential recipients. The relevant plasma fractionation institute must also be notified.

Tracing of recipients of potentially infectious blood donations (look-back/review)

Blood establishments should initiate a look-back procedure that is aimed at tracing recipients of blood components from a potentially infectious blood donation and notifying these recipient(s), via their treating physicians, whenever a blood donation may have taken place within the window period of a (repeat) donor with a confirmed HIV, HBV or HCV infection.

Implicated donations include those within a timeframe equal to the maximum test-specific window period of the infection preceding the last negative screening test result in the donor.

The blood establishment should inform the hospital in writing about the incident and advise the hospital to trace the recipient(s) of the implicated blood component(s) and inform the treating physician about the potentially infectious transfusion. The relevant plasma fractionation institute must also be notified.

It is the responsibility of the treating physician to inform the recipient about a potentially infectious transfusion, unless there are medical arguments not to do so. If the recipient is tested in order to establish or to exclude infection, the blood establishment should be informed by the hospital about such test results. If testing of the recipient is not performed, the blood establishment should also be informed of this by the hospital.

If the recipient is confirmed to be positive for the infection, the incident must be reported to a national haemovigilance system and/or to the Competent Authorities.

Consistent with the recommendations of the national health authority, blood establishments should consider the need to trace and notify blood component recipients and/or their physicians in cases where a blood donor is subsequently diagnosed with vCJD infection.

5. Contracts between blood establishments and hospitals for haemovigilance

In those situations in which blood collection and processing is carried out in facilities located outside of hospitals, the look-back, trace-back and recall procedures may be described in the contract(s) between the blood establishment and the hospital(s).

Minimum information to be captured in the initial incident report at hospital level

Information about transfused patients must be managed according to the confidentiality requirements/legislation of individual countries.

Reported patient identifiers should include at least date of birth, gender and a unique case number. Any clinical signs observed should be documented in a standardised fashion, either specific for a given adverse event or reaction or the same format for every untoward effect. The clinical outcome of all adverse reactions should be stated.

6. Reporting haemovigilance data

Standardisation of reporting

Reports of adverse events and reactions should be made in the same way in all the institutions that participate in the haemovigilance network. This strategy implies not only use of common report forms, but also a common training programme that ensures a similar method of interpretation for a given incident as well as a common and agreed definition of the different types of adverse events and adverse reactions among all participants. In this respect, the persons specifically in charge of haemovigilance may contribute to the standardisation of both reports and definitions.

In practice, standardisation of reporting requires an active training policy initiated inside the network.

Data analysis

All the reports should be carefully analysed before inclusion in the haemovigilance database, which can be exploited at different levels: institutional, regional, national or international. Whatever the magnitude of the network, an individual institution should have permanent and full access to its own data.

Information sent to the haemovigilance database

Information about transfused patients must be managed according to the confidentiality requirements/legislation of individual countries.

Component information

This information should include a detailed description of the component involved:

- unit number and appropriate codes for components;
- description of the component, including:
  - the type of component, i.e. red cell, platelet or plasma;
  - the type of preparation, i.e. from whole blood or from apheresis;
  - other characteristics, i.e. leucocyte-depleted, irradiated, plasma-reduced, etc.;
  - conditions and duration of storage prior to transfusion.

Information about severity

Severity should be graded. A suggested scale is as follows:

**Severity scale**	
0	No clinical signs
1	Immediate signs without vital risk and full resolution
2	Immediate signs with vital risk
3	Long-term morbidity
4	Death

Information about imputability

The possible relationship between the observed adverse reaction and the transfusion of blood components given (imputability) should be identified. A scale, required in the EU, is as follows (Directive 2005/61/EC):

**Imputability scale**		**Explanation**
0	Excluded	When there is conclusive evidence beyond reasonable doubt for attributing the adverse reaction to alternative causes.
0	Unlikely	When the evidence is clearly in favour of attributing the adverse reaction to causes other than the blood or blood components.

Imputability scale		Explanation
N/A	Not assessable	When there is insufficient data for causality assessment.
1	Possible	When the evidence is indeterminate for attributing the adverse reaction either to the blood or blood component or to alternative causes.
2	Likely, probable	When the evidence is clearly in favour of attributing the adverse reaction to the blood or blood component.
3	Certain	When there is conclusive evidence beyond reasonable doubt for attributing the adverse reaction to the blood or blood component.

Information about the type of adverse events and reactions

Report forms should enable differentiation between adverse reactions in patients and donors, as well as from adverse events.

Report forms should include a brief summary that describes the event, as well as the corrective actions taken.

In order to provide an evaluation of the incidence of untoward effects, each participating institution should provide the number of blood components used per year and the number of patients transfused, together with details of all reported events.

Additional information about the current guidelines and procedures in regard to the use of blood components is useful in comparing the results from different institutions or even different countries.

Chapter 11

Principles of clinical use of blood

1. Overview

The clinical transfusion process encompasses the 'transfusion of the right blood component to the right patient at the right time, in the right condition and according to appropriate guidelines'. It is a chain of inter-related events beginning with the appropriate decision that the patient needs transfusion of one or more blood components and ending with the assessment of the clinical outcome of the transfusion.

The safety of transfusion of blood components is underpinned by several key measures:

- the decision to transfuse;
- the completion of the transfusion request form;
- the correct identification of the patient and obtaining a pre-transfusion sample;
- the pre-transfusion testing within the laboratory;
- the selection and issue of appropriate blood components;
- the administration of the component to the patient with appropriate monitoring.

For a safe and appropriate use of blood in therapy, it is necessary to have in place a 'Quality System for Clinical Transfusion' involving different health professionals. Structures and individuals that contribute to the governance of the process include the hospital management, the Hospital Transfusion Committee (HTC), the hospital blood bank and/or the blood establishment providing blood components to the hospital or to the patient, and all hospital staff involved in the transfusion chain and in the haemovigilance system.

Elements of the quality system include:

- adoption and regular updating of clear guidelines for appropriate use of blood and blood components;
- adoption of SOPs for the implementation and surveillance of appropriate blood utilisation;
- thorough dissemination of guidelines and SOPs;
- appropriate selection of suitable blood components for each clinical condition;
- safe storage, issue and handling of blood components;
- ensuring correct patient identification throughout the transfusion process;
- safe administration of the component and monitoring of the patient;
- recognition, management and prevention of adverse effects of transfusion;
- constant monitoring of quality and revision of the processes, results, and blood components of transfusion medicine activities;
- definition of staff responsibilities and needs for training and education.

2. Decision to transfuse

Transfusion of blood components must follow appropriate evidence-based guidelines that are updated regularly. The indication for transfusion should be documented in the patient clinical record.

Where possible, informed consent should be obtained from the patient prior to transfusion. This is mandatory in some countries. It is the responsibility of the prescribing physician and the consent should be documented in the clinical record of the patient. Information could be delivered orally but is preferable in written form and should include appropriate information on the risks and benefits of transfusion therapy and its alternatives. The written information provided should be approved by the Hospital Transfusion Committee.

Before ordering the transfusion, the treating doctor should be aware of the patient's transfusion history including any adverse reactions.

The decision to transfuse should be evidence-based. Therefore, professionals should be familiar with the recommendations of good quality and regularly updated transfusion guidelines that take into account the best available current evidence.

These guidelines should contain detailed instructions on appropriate use of blood components for each clinical condition, guidance on the dosage and need for special requirements (e.g. irradiated, washed).

It is strongly recommended that specific guidelines or recommendations are available, at least for transfusion management in:

- critical/massive haemorrhage;
- obstetric haemorrhage;
- paediatrics;
- intensive care patients;
- cardiovascular surgery;
- patients with haemoglobinopathies and other haematological-transfusion dependent chronic disorders;
- haematopoietic stem cell transplant;

- patients with immune cytopenias, thrombotic thrombocytopenic purpura, coagulation factor deficiencies and disseminated intra-vascular coagulation.

The HTC should plan and review the results of regular transfusion audits and make the audit reports available to prescribing clinicians so that when significant deviations from the guidelines are observed, corrective actions can be put in place.

It is recommended that clinical services include blood components' quality indicators as part of their quality programme management.

The medical staff of the blood establishment and hospital blood bank should provide transfusion medicine clinical support and advice on all aspects of the process.

Patient blood management

Blood transfusion medicine/therapy is a key part of patient blood management (PBM) programmes. These aim to provide the best clinical care, optimising the patient blood counts, reducing unnecessary blood losses and procuring a judicious use of blood components. PBM is based on an interdisciplinary overview of patient´s necessities.

Blood transfusion services and all blood establishments' stakeholders should be directly involved in PBM programmes.

Medical schools, hospitals and blood establishments must support education in transfusion medicine including a specific educational programme in PBM for clinicians in training.

Transfusion of blood components should be ordered when there are no better alternatives. When available, possible alternatives should be discussed with the patient and his/her opinion must be taken into account. Physicians should be aware of alternative treatments which can be less harmful or more specific, and could substitute or avoid blood-component transfusion: coagulation factor concentrates; erythropoietin; agonists of thrombopoietin receptor; antifibrinolytic agents, blood recovering devices and other autotransfusion modalities.

Considering that blood components are a scarce, high-cost resource, they should only be prescribed when some clinical benefit is expected. A transfusion should not be ordered when it could be considered a futile treatment or when potential side effects outweigh their benefit.

3. Completion of the transfusion request form, identification of patient and blood sampling

The transfusion request should be made by a medical doctor or, if permitted, by specially trained nurses.

Detailed instructions for the completion of the request form and the taking of pre-transfusion samples should be available and all staff permitted to make these requests should be trained and competent to undertake this role.

The number of units, type(s) of blood component(s) and associated special requirements (e.g. irradiation or washing), date and location of the transfusion, clinical indication and the urgency of the transfusion should be indicated on the request form. Information on transfusion history and recent pregnancy is also necessary to determine the period of validity of the pre-transfusion sample.

A procedure for auditing transfusion requests should be in place in order to identify compliance with local clinical guidelines and to facilitate interventions to improve this and, where appropriate, to update the guidelines. Validated information technology tools which provide alerts or support physicians in transfusion decision-making are useful.

4. Correct identification of the patient and obtaining a pre-transfusion sample

Collection of blood samples from the intended recipient for pre-transfusion testing is a crucial point in the safety of the transfusion chain.

Where appropriate, the request form must be accompanied by the appropriate blood samples for pre-transfusion testing.

Procedures should be in place to ensure that samples have been drawn from the correct patient. Minimum requirements for patient

identification are family name, given name(s) and date of birth. Where applicable, these data should be supplemented by a unique patient identity number.

Whenever possible, at the time of sampling positive patient identification must be performed. The patient should be asked to state his/her name and date of birth if conscious and/or these or other identifiers should be checked on a wrist band securely attached to the patient. The information, on the tube label must be crosschecked and be identical with that on the request form.

In newborn infants, the gender and the number on the identification wristband should also be recorded on the request form and the sample tube.

If it is not possible to establish a patient's identity, a procedure must be in place to otherwise uniquely identify the intended recipient.

Any patient identification discrepancy at any step of the process must be investigated and corrected.

5. Testing within the laboratory

Information on this is provided in Chapter 8, Principles of immunohaematology.

6. Selection and issue of appropriate blood components

Before issuing a blood component, the hospital blood bank will check that the correct component has been selected, special requirements have been fulfilled and the component(s) remains in-date. A check of the integrity of the unit will also be made.

A compatibility label will then be attached to the component containing the patient identifiers obtained from the sample and/or request form.

Handling and storage of blood components in hospital clinical areas

Transport should be undertaken using systems that maintain the integrity of blood components. Procedures should be in place to document receipt of the issued blood components in the clinical area.

To avoid compromising clinical effectiveness and safety, blood components should be transfused within the time limits required by the current rules or local procedures. It is recommended that the blood component should not remain out of controlled storage for more than 30 minutes if it is not transfused and it is to be returned to storage.

Relevant staff should be properly trained in the principles and practice of handling different types of blood components and written procedures should be available.

7. Administration of blood components

Procedures must be in place to verify the identity of the recipient at the bedside in order to ensure that the blood component is to be transfused to the intended recipient. This involves asking the patient to state his/her name and date of birth and/or by checking the identification details on the patient's wrist-band against the information provided on the compatibility label.

In addition, confirmation of compatibility between patient and blood component should be carried out by:

- checking the written – or electronic – prescription (including special requirements);
- checking the record of the patient's blood group against the blood group on the blood component label;
- checking that the unique identification number on the blood component label matches that on the compatibility label and/or on the hospital blood bank report, where available. Prior to commencing the transfusion a check should be made to verify that the expiry date of the blood component has not been passed and that there is no visible deterioration of the blood components

(with particular emphasis on discolouration or detectable micro-perforations of the bag).

Where undertaken, the bedside confirmation of ABO group should then be performed.

Blood components must be administered using an approved blood administration set that incorporates an integral mesh filter (usually 150–230 microns) to filter out large clots and aggregates and ensure an effective flow rate. This set and any other infusion equipment should be used in accordance with manufacturer recommendations. It is recommended that no transfusion sets be used for more than 6 hours.

Transfusion should be completed within 4 hours of removal from controlled storage.

To ensure traceability, all blood components administered should be recorded in the clinical patient record, including the component identification number and the start and end times of the transfusion.

Special precautions

Warming of blood

Hypothermia induced by rapid/massive transfusion (more than 50 mL/kg/hour in adults and 15 mL/kg/hour in children) increases the risks of organ failure and coagulopathy. If warming of blood is indicated, only validated and regularly controlled warming devices should be used in accordance with manufacturer instructions.

Addition of medicinal products or infusion

Because of the risk of damage to the blood components, addition of medicinal products or infusion solutions to blood units should be avoided unless their safety has been demonstrated.

Transfusion monitoring

Observation of the patient during and after transfusion is essential. Observation during the first 15 minutes of the transfusion is especially important to allow early detection of signs of serious acute reactions.

Requirements should be documented in procedures and personnel should be trained.

Vital signs such as blood pressure, pulse, respiratory rate and temperature should be measured before starting the transfusion, at 15 minutes after the start of the transfusion and at the end of the transfusion of every blood component transfused.

The time when transfusion is started, interrupted and stopped should be clearly reported in patient records, as well as vital signs or any other symptoms that could indicate a transfusion reaction.

Confirmation of transfusion of the blood component should be sent back to the hospital blood bank.

An assessment of the effectiveness of the transfusion should be performed (by post-transfusion increment rates or improvements in patient symptoms and clinical signs) and documented in a clinical record, identifying whether the desired effect was obtained and the likely need for further transfusion.

Management and reporting of transfusion reactions

Complications may occur during or immediately after the transfusion, or after a delay of hours, days or months. Therefore, careful documentation of the transfusion as well as recording and reporting of transfusion complications is essential.

Transfusion complications include both adverse events and adverse reactions associated with transfusions and even failure of expected therapeutic response. Careful recording and reporting of any observed reaction is the responsibility of the attending physician.

In the event of a suspected transfusion reaction, the transfusion should be stopped and the line should be maintained with normal saline. The patient should be assessed for severity of the reaction and treated accordingly. Where the reaction is a mild allergic or febrile reaction and settles with treatment, the transfusion may be restarted at a slower rate with more frequent observations. For severe reactions the key priority is resuscitating the patient and treating any specific

symptoms or suspected causes of the reaction. As ABO-incompatible red cells can cause such a reaction, a clerical check of the documentation associated with the transfusion should be undertaken, including an identification check of the recipient and blood component and a check that the ABO and RhD blood group of the component is compatible with the patient's blood group. New samples should be taken from the patient and the transfusion packs and, together with a transfusion reaction report, these should be sent to the hospital blood bank for further investigation if clinically indicated. In order to assist laboratory investigations of suspected reactions, transfusion packs should not be discarded for at least 24 hours after commencement of the transfusion.

Respiratory complications of blood transfusion are increasingly recognised and have been shown by haemovigilance schemes to be associated with a high mortality in vulnerable patient groups. Any patient experiencing new or worsening breathlessness during or after a transfusion should be fully assessed by a medical doctor to determine if there is an allergic reaction, TACO or TRALI which should then be investigated and managed accordingly. Air embolism is now a rare complication of blood transfusion.

When clinical symptoms and signs suggest the possibility of bacterial infection, blood cultures should be obtained from the patient as well as bacterial culture from the blood component bag. Care should be taken not to contaminate the content of the bag after disconnecting from the patient.

In countries where universal pre-storage leucocyte depletion has not been implemented, the use of leucocyte-depleted blood components for subsequent transfusions is recommended for patients with repeated, febrile non-haemolytic transfusion reactions.

Long-term complications may also occur. These mainly comprise immunological complications e.g. allo-immunisation and transmission of infectious pathogens.

Haemosiderosis is a serious complication of chronic red cell transfusion affecting patients suffering from transfusion-dependent

conditions. Unless patients undergo iron-chelation therapy to control iron overload in the liver and heart, this complication may lead to severe organ impairment and death before the third decade of life.

There should be co-operation between the clinician responsible for the patient and the hospital blood banks/blood establishment to facilitate investigation of possible transfusion-transmitted infections (TTI). Suspected TTI may require investigation when the recipient develops a viral or bacterial infection after transfusion or a donor is found to have developed an infectious disease marker. Medical follow-up of recipients and donors will be required to determine causality.

Follow-up and patient counselling, where appropriate, is also necessary when significant allo-immunisation against transfused cells may have taken place.

Traceability and haemovigilance

Facilities where transfusion occurs should have procedures in place to ensure retention of at least the following data: identification of blood component supplier, the identity and type of blood component transfused, the name of the recipient and the date of transfusion. For any issued blood component that is not transfused, confirmation and date of subsequent disposal should be included.

Each adverse reaction or event related to the transfusion should be investigated, recorded and notified to the haemovigilance system, including errors with no consequence for the patient. Procedures must be in place to notify blood establishments without delay of any serious adverse reactions which may be attributable to the quality or safety of blood and blood components.

These issues are considered in further details in Chapter 10, Principles of haemovigilance.

8. Hospital transfusion committees

Establishment of hospital transfusion committees (HTCs) is to be encouraged. The hospital chief executive and senior hospital management have the responsibility to support and resource the HTC.

A hospital blood transfusion committee should include representatives of the hospital blood bank, the blood establishment and the main clinical units with significant transfusion activity. It is recommended that physicians, nurses and administrative personnel be represented on these committees.

The main goals of HTCs are:

- to define blood transfusion policies adapted to local clinical activities;
- to perform regular evaluation of blood transfusion practices;
- to analyse adverse events and adverse reactions related to blood transfusion;
- to take any corrective and preventive measures if necessary;
- to ensure that all staff involved in transfusion practice receive adequate training.

Audit systems for the clinical use of components further enhance the efficacy and safety of transfusion practices.

STANDARDS

Chapter 1

Introduction

The Standards section of this Guide contains minimum standards for blood establishments and hospital blood banks that are required to comply with European Union (EU) Directive 2005/62/EC. The Standards are aligned closely with European Commission Directives, and include blood component monographs that mirror the structure used in the European Pharmacopoeia. This section states what must be done.

Elements of the Standards that are transcribed from EU Directives are legally binding on blood establishments and hospital blood banks within the EU. These are highlighted within this section of this Guide. The specific numbering used in Directive 2005/62/EC is to assist in cross-referencing of EU requirements. The Standards may also be of benefit to Council of Europe member states outside the EU and to other organisations involved in transfusion activities outside Europe.

Chapter 2

Standards for selection of donors

1. Overview

Measures must be taken to promote the collection of blood and blood components from voluntary non-remunerated donations according to the principles set out in the Convention for the Protection of Human Rights and Dignity of the Human Being with Regard to the Application of Biology and Medicine (Convention on Human Rights and Biomedicine, ETS No. 164) and its Additional Protocol concerning Transplantation of Organs and Tissues of Human Origin (ETS No. 186).

Blood establishments are ultimately responsible for the quality and safety of the blood and blood components collected, and must be entitled to decide on the final acceptance or deferral of a donor or a prospective donor, taking into account Resolution CM/Res (2008) 5 on donor responsibility and on the limitations to donation of blood and blood components.

2. Information to be provided to the donor

The following information must be provided to prospective donors of blood or blood components:

- accurate educational materials that are understandable for members of the general public about the: essential nature of blood; blood-donation procedure; components derived from whole blood and apheresis donations; important benefits to patients;
- for both allogeneic and autologous donations, the reasons for requiring a medical assessment, health and medical history, the testing of donations and the significance of 'informed consent':
  - for allogeneic donations, self-deferral, and temporary and permanent deferral, and the reasons why individuals must not donate blood or blood components if there could be a risk for the recipient or the donor;
  - for autologous donations, the possibility of deferral and the reasons why the donation procedure cannot take place in the presence of a health risk to the individual, whether as a donor or recipient of the autologous blood or blood components;
- information on the protection of personal data: no unauthorised disclosure of the identity of the donor, of information concerning the donor's health or of the results of the tests performed;
- the reasons why individuals must not make donations that may be detrimental to their health;
- specific information on the nature of the procedures involved in the allogeneic or autologous donation process and their respective associated risks. For autologous donations, the possibility that the autologous blood and blood components may not suffice for the intended transfusion requirements;
- all blood donors must be provided with accurate and updated information on HIV/AIDS and hepatitis transmission and be given the opportunity for self-exclusion so that those persons who have unsafe sex practices or other risk behaviours exposing them to potential infectious sources refrain from donating;
- information on the option for donors to change their mind about donating before proceeding further, or the option to withdraw

or self-defer at any time during the donation process without undue embarrassment or discomfort;

- the reasons why it is important that donors inform the blood establishment of any subsequent event that may render any prior donation unsuitable for transfusion;
- information on the responsibility of the blood establishment to inform the donor, through an appropriate mechanism, if test results show any abnormality of significance to the donor's health;
- information why unused autologous blood and blood components are discarded and not transfused to other patients;
- information that test results detecting markers for viruses, such as HIV, HBV, HCV or other relevant blood-transmissible microbiologic agents, will result in donor deferral and destruction of the collected unit and, when required by law, that the results will be reported to health authorities;
- information on the opportunity for donors to ask questions at any time.

This information provides the basis for informed consent from the donor that must be obtained before proceeding to donation.

3. Medical assessment of the donor

Donor eligibility

Procedures for safe donor identification, the suitability interview and eligibility assessment must be implemented and maintained. They must take place before each donation and comply with the requirements set out in Annex II and Annex III to Directive 2004/33/ EC (Directive 2005/62/EC Annex 6.1.1).

All donors must undergo a screening process to assess their suitability. Only healthy individuals with an appropriate medical history can be accepted as donors of blood or blood components.

The donor must be properly identified.

The selection process must include assessment of each donor carried out by a suitably qualified individual who has been trained to use accepted guidelines and who works under the direction of a physician. This assessment involves an interview, a questionnaire and further direct questions, if necessary.

Questionnaire and interview

The questionnaire must be designed to elicit information relevant to the health and lifestyle of the donor. It must be designed to be understandable by the donor and given to all donors each time they attend. On completion, it must be signed by the donor.

A confidential interview must be conducted by specifically trained staff to ask further direct questions to supplement the information in the questionnaire. The person who carries out the assessment must certify that the relevant questions have been asked.

The donor interview must be conducted in such a way as to ensure confidentiality (Directive 2005/62/EC Annex 6.1.2).

The donor suitability records and final assessment must be signed by a qualified health professional (Directive 2005/62/EC Annex 6.1.3).

Donor details

There must be secure, unique donor identification, contact details and robust mechanisms linking the donors to each of their donations.

Age of the donor

The age limits for donation are a minimum of 18 years and maximum of 65 years.

Where allowed by national legislation, blood donation may be considered from donors aged 17.

Donation by first-time donors above the age of 60 years is at the discretion of the responsible physician.

Donation by regular donors over 65 years is at the discretion of the responsible physician. Permission to continue donating after the age

of 65 years can be given annually by the responsible physician either individually to each donor or based on a medical risk assessment for a given donor population.

Donor appearance and inspection

Special note must be taken in case of plethora, poor physique, debilitation, undernutrition, anaemia, jaundice, cyanosis, dyspnoea, mental instability and intoxication from alcohol or drugs.

4. Donor deferral

Deferred donors must be given a clear explanation of the reasons for deferral.

Tables 2.1, 2.2 and 2.3 list conditions for deferral. Specific conditions for infectious diseases are listed.

Table 2.1. Conditions leading to permanent deferral

Cancer/ malignant diseases	Individuals with a malignant disease, or a history of such, are usually permanently deferred. The physician in charge may make exceptions to this rule in selected cases (see Principles).
Creutzfeldt-Jakob disease	All individuals who have in the past been treated with extracts derived from human pituitary glands, have been recipients of dura mater or corneal grafts or who have been told of a family risk of Creutzfeldt-Jakob disease or any other transmissible spongiform encephalopathy. [a]
Diabetes	If insulin therapy is required, rDNA insulin is used.
Drugs	Any history of injectable drug abuse.
Cardiovascular disease	Persons with a history of serious heart disease, especially coronary disease, angina pectoris, severe cardiac arrhythmia, a history of cerebrovascular diseases, arterial thrombosis or recurrent venous thrombosis (see also Principles, Chapter 2).

a A family history of CJD carries a presumption of family risk unless it is determined that: (a) the affected family member had vCJD, not CJD; or (b) the affected family member did not have a genetic relationship to the donor; or (c) the cause of CJD in the affected family member was iatrogenic; or (d) the donor was tested and is known to have a normal genetic polymorphism for PrPc.

b Deferral requirements may be waived by the blood establishment if the donation is used exclusively for plasma for fractionation.

Infectious conditions	Certain infectious states and diseases necessitate permanent deferral:<br>a) Carriers of HIV 1/2, HTLV I/II, HBV and HCV<br>b) Babesiosis [b]<br>c) Leishmaniasis; (Kala-Azar) visceral leishmaniasisb<br>d) Chronic Q fever [b]<br>e) Trypanosomiasis cruzi (Chagas disease) [b]<br>(see also Chapters 2 and 9 Infectious diseases)<br>f) Persons whose sexual behaviour puts them at high risk of acquiring severe infectious diseases that can be transmitted by blood.
Xenotransplantation	All recipients.

a A family history of CJD carries a presumption of family risk unless it is determined that: (a) the affected family member had vCJD, not CJD; or (b) the affected family member did not have a genetic relationship to the donor; or (c) the cause of CJD in the affected family member was iatrogenic; or (d) the donor was tested and is known to have a normal genetic polymorphism for PrPc.

b Deferral requirements may be waived by the blood establishment if the donation is used exclusively for plasma for fractionation.

Table 2.2. Conditions leading to temporary deferral (suspension)

Condition	Deferral period
Endoscopy with biopsy using flexible instruments, inoculation injury, acupuncture, [a] tattooing [a] or body-piercing, mucosal splashes with blood, tissue or cell transplant of human origin	6 months (or 4 months, provided a NAT test for hepatitis C is negative).
Transfusion of blood components	6 months or for 4 months provided a NAT test for hepatitis C is negative. Injection of red cells as part of an approved immunisation programme will need clinical assessment.
Epilepsy	3 years off treatment and without an attack.
Fever above 38 °C, flu-like illness	2 weeks following cessation of symptoms.

a Exceptions can be made according to national risk assessments.

Condition	Deferral period
Kidney disease	Acute glomerulonephritis: 12 months deferral period following full recovery (well, on no treatment and discharged from specialist care).
Medication	The taking of a medication may indicate an underlying disease which may disqualify the donor. It is recommended that a list of commonly used drugs, with rules for acceptability of donors, approved by the medical staff of the transfusion centre, be made available. Donors treated with prescribed drugs, particularly those with proven teratogenic effect, should be deferred for a period consistent with the pharmacokinetic properties of the drug.
Osteomyelitis	2 years after having been declared cured.
Pregnancy	6 months after delivery or termination, except in exceptional circumstances and at the discretion of a physician.
Rheumatic fever	2 years following attack with no evidence of chronic heart disease. The latter complication is a cause for permanent deferral.
Surgery	After surgery, persons should not donate until they have recovered fully and are fit to be donors (typically about 6 months for major surgery).
Tooth extraction	If no complications, 1 week (because of possible risk of transient bacteraemia).
Tropical infections	6 months following return from tropical areas and then only if they have not suffered an unexplained fever or illness (see Chapters 2 and 9, Infectious diseases).

a Exceptions can be made according to national risk assessments.

Table 2.3. Vaccinations

Inoculations, vaccinations	Deferral period
Vaccines with attenuated bacteria and viruses: BCG, yellow fever, rubella, measles, poliomyelitis (oral), mumps, live attenuated typhoid fever, vaccinia, live attenuated cholera vaccine	4 weeks<br>Vaccinia for smallpox at 8 weeks
Vaccines with killed bacteria:<br>cholera, typhoid, capsular polysaccharide typhoid fever vaccine	Accept, if well
Vaccines with inactivated viruses:<br>poliomyelitis (injection), influenza	Accept, if well
Toxoids: diphtheria, tetanus	Accept, if well
Other vaccines:<br>hepatitis A and B vaccine, hepatitis B vaccine, rabies, tick-borne encephalitis	Accept, if well and no exposure is reported<br>1 week in order to prevent vaccine-related positivity in HBs-antigen result<br>Accept, if well<br>One year if post-exposure

Infectious diseases

HIV/AIDS

Persons whose sexual behaviour puts them at a high risk of acquiring severe infectious diseases that can be transmitted by blood must be deferred permanently.

Current sexual partners of people with HIV must be deferred. Previous sexual partners of people with HIV are acceptable 12 months after the last sexual contact.

Brucellosis (confirmed)

Deferral for at least two years following full recovery.

The deferral period does not apply when the donation is used exclusively for plasma fractionation.

Chagas disease

Individuals with Chagas disease or who have had Chagas disease must be permanently deferred.

The blood of persons who were born or have been transfused in areas where the disease is endemic should be used only for the production of plasma that is used exclusively for fractionation into plasma derivatives, unless a validated test for infection with *T. cruzi* is negative.

Jaundice and hepatitis

Individuals with a history of jaundice or hepatitis may, at the discretion of the appropriate Competent Authority, be accepted as blood donors provided a CE-marked test for HBsAg and anti-HCV is negative.

Persons who have been in close household contact with an individual infected by the hepatitis-B virus (acute or chronic) must be deferred for 6 months (4 months if appropriate testing has been performed) from the time of contact unless demonstrated to be immune.

Current sexual partners of people with HBV must be deferred, unless demonstrated to be immune.

Previous sexual partners of people with HBV are acceptable after 6 months since the last sexual contact. This can be reduced to 4 months if HBV NAT and anti-HBc are performed and both test results are negative.

Malaria[1]

Since questioning a donor as to the country(s) in which he/she was born, brought up or has visited is essential for effective detection, every blood establishment must have a current map or list of the endemic zones and time frames in the countries concerned.

---

1 Tests and deferral periods may be waived by the blood establishment if the donation is used exclusively for plasma fractionation.

Persons who have lived in a malaria-endemic area for a continuous period of 6 months or more at any time in their life

These persons may become asymptomatic carriers of the malaria parasite. Therefore, the following rules must apply to these individuals after each return from a malaria area:

- may be accepted as blood donors if the result of a validated immunological test for antibodies to the malaria parasite, performed at least 4 months after the last visit to a malaria area, is negative;
- if the test is repeatedly reactive, the donor must be deferred and may be re-evaluated after a suitable period when the antibody test may have reverted to negative (a period of 3 years is suggested);
- if the test is not performed, the donor must be deferred until the test is performed and negative.

Persons who give a history of malaria

- Must be deferred until asymptomatic and off treatment.
- May be accepted as blood donors if the result of a validated immunological test for antibodies to the malaria parasite performed at least 4 months since cessation of treatment/last symptoms is negative.
- If the test is repeatedly reactive, the donor must be deferred and may be re-evaluated after a suitable period when the antibody test may have reverted to negative (a period of 3 years is suggested).
- If the test is not performed, the donor should be deferred until the test is performed and negative.

Persons who report an undiagnosed febrile illness consistent with malaria during or within 6 months of the end of a visit to a malaria area

- May be accepted as blood donors if the result of a validated immunological test for antibodies to the malaria parasite,

performed at least 4 months since cessation of treatment/last symptoms, is negative.

- If the test is repeatedly reactive, the donor should be deferred and may be re-evaluated after a suitable period when the antibody test may have reverted to negative (a period of 3 years is suggested).
- If the test is not performed, the donor should be deferred until the test is performed and negative.

All other persons who have visited a malaria-endemic area without reporting any clinical symptoms consistent with malaria

- May be accepted as blood donors if the result of a validated immunological test for antibodies to the malaria parasite performed at least 4 months after the last visit to a malaria-endemic area is negative.
- If the test is repeatedly reactive, the donor must be deferred and may be re-evaluated after a suitable period when the antibody test may have reverted to negative (a period of 3 years is suggested).
- If the test is not performed, the donor may be re-accepted once a period of 12 months has elapsed after the most recent return from a malaria area.

Q Fever[1]

Deferral until two years following the date confirmed as cured.

Syphilis[1]

Deferral until one year following the date confirmed as cured.

Toxoplasmosis

Deferral until 6 months following clinical recovery.

---

1 Tests and deferral periods may be waived by the blood establishment if the donation is used exclusively for plasma fractionation.

Tuberculosis

Deferral until two years after having been declared as cured.

Variant Creutzfeldt-Jakob disease

Deferral of donors as a preventative measure for vCJD must be based on appropriate risk assessment.

West Nile virus[1] (WNV)

Deferral until 28 days after leaving an area with ongoing transmission of the disease to humans unless a validated NAT is performed. Persons with a diagnosis of WNV must be deferred until 120 days after recovery.

Zika virus

Deferral until 28 days after leaving an area with ongoing transmission of the disease to humans unless a validated NAT is performed. Persons with a diagnosis of Zika virus infection must be deferred until 120 days after recovery.

5. Specific standards for donors of different types of components

Whole blood donors

A standard donation must not be collected from persons weighing less than 50 kg.

Quantity of donation

Volume of donation

A standard donation of whole blood (excluding anticoagulants) must not exceed 500 mL and usually consists of a donation of 450 mL ± 10 %. This does not include any allowance for samples taken for laboratory tests and for retention of a donor sample. The volume of

1 Tests and deferral periods may be waived by the blood establishment if the donation is used exclusively for plasma fractionation.

a standard donation of whole blood (including samples) must not exceed 15 % of the calculated blood volume of the donor.

Frequency of whole blood donations

A maximum of 6 standard donations per year can be taken from men and up to 4 per year from women, with a minimum interval between standard donations of 2 months.

These maximum donation rates must never be exceeded under any circumstances, and should only be adopted after careful consideration of the dietary habits of the populations concerned and in the knowledge that extra care may be necessary, beyond routine haemoglobin or haematocrit estimation, in the monitoring of donors for iron deficiency.

Laboratory examination

- Haemoglobin concentration must be determined each time the donor attends to donate blood or blood components.
- Minimum values before donation:
  - female donors: 125 g/L or 7. 8 mmol/L (minimum haematocrit = 0.38)
  - male donors: 135 g/L or 8. 4 mmol/L (minimum haematocrit = 0.4)
- Individual donations may be accepted below these levels after consultation with the responsible physicians or as established by a Competent Authority, based on norms for their specific populations.

Apheresis donors

The supervision and medical care of apheresis donors must be under the responsibility of a physician specially trained in these techniques.

Other than in exceptional circumstances (to be decided by the responsible physician), donors for apheresis procedures must meet the criteria for whole blood donations.

People with sickle cell trait must not donate red cell components by apheresis.

Donors taking medicinal drugs that inhibit platelet functions must be temporarily deferred from donation by platelet apheresis.

Frequency of donation and maximal amounts of plasma and red cells to be collected

The total blood volume of each donor must be estimated.

The maximal extra-corporeal volume during apheresis must never be higher than 20 %.

The collection volume (excluding anticoagulant) for each plasmapheresis procedure must not exceed 16 % of the estimated total blood volume, and should never exceed 750 mL unless fluid replacement is undertaken.

Not more than 1.5 L of plasma may be collected from any one donor per week.

A maximum of 33 plasmapheresis procedures may be performed per donor per year. This equates to a maximum annual collection volume of 25 L, based on the maximum volume of 750 mL plasma (excluding anticoagulant) per procedure.

In any apheresis procedure involving collection of plasma, platelets and/or red cells in one apheresis procedure, the total volume of all components collected (plasma, platelets and red cells) must not exceed 16 % of total blood volume, with a maximum of 750 mL (exclusive of anticoagulant), unless fluid replacement is undertaken.

The total amount of red cells collected must not exceed the theoretical amount of red cells that would bring the donor haemoglobin in isovolemic situation below 110 g/L.

The interval between one plasmapheresis or plateletpheresis procedure and a donation of whole blood or apheresis procedure incorporating collection of a single or double unit of red cells (whereby one unit is equivalent to a red cell component obtained from one whole blood donation) must be at least 48 hours. The interval between a whole

blood donation, an apheresis red cell collection or a failed return of red cells during apheresis, and the next apheresis procedure without red cell collection, must be at least one month. The interval between two single-unit red cell collections must be the same as for collections of whole blood.

Additional requirements for donors undergoing plasmapheresis

Protein analysis, such as determination of total serum or plasma protein and/or electrophoresis and/or quantitation of single proteins (especially albumin and IgG) must be performed. Analysis must be carried out at the first donation and at least every year thereafter. Total proteins must not be less than 60 g/L. IgG levels must be within reference values of the normal population and should not fall below 6.0 g/L. Donors with results below these limits must be deferred until IgG and total protein levels are back to normal.

Additional requirements for donors undergoing plateletpheresis

Platelet apheresis must not be carried out on individuals whose platelet count is less than 150×10^9/L. Donors must not be subjected to a platelet apheresis procedure more often than once every 2 weeks. An exception to the donation interval and platelet count may be made in the case of HLA-/HPA-matched donations and for IgA-negative donors at the discretion of the physician responsible for the procedure.

Additional requirements for donors undergoing double unit red cell apheresis

The donor must have an estimated blood volume of > 4 500 mL. The total blood volume must be calculated on the basis of gender, height and weight (see Appendix 2, Tables 1 and 2).

Haemoglobin levels must be determined before each donation and the minimum value must be > 140 g/L. The haemoglobin level of the donor must not fall below 110 g/L after donation.

The interval between a whole blood donation and the donation of a double unit of red cells must be at least 3 months. The interval between a double-unit red cell apheresis and a donation of whole

blood or another double-unit red cell apheresis must be at least 6 months for women and 4 months for men.

The maximum volume of red cells collected must not exceed 400 mL (without re-suspension solution) per collection procedure.

Total red cell volume collected per year must not exceed that acceptable for whole blood donors.

6. Post-donation information

Blood donors must be instructed to inform the blood establishment when signs or symptoms occur after a donation that might indicate that the donation may have been infectious.

Chapter 3

Standards for collection of blood and blood components

1. Premises for donor sessions

Premises (including those of mobile teams) must satisfy general requirements for the health and safety of the staff and donors concerned with due regard to relevant legislation or regulations.

Suitable facilities must be provided to allow a private interview with each donor, assuring privacy and confidentiality.

Before premises are accepted for mobile donor sessions, their suitability must be assessed against the following criteria:

- sufficient size to allow proper operation and ensure donor privacy;
- safety for staff and donors;
- the presence of ventilation, electrical supply, lighting, hand-washing facilities, reliable communication, blood storage and transport.

2. Procedures and equipment used at blood donation sessions

The blood collection procedure must be designed to ensure that the identity of the donor is verified and securely recorded and that the link between the donor and the blood, blood components and blood samples is clearly established (Directive 2005/62/EC Annex 6.2.1).

The sterile blood bag systems used for the collection of blood and blood components and their processing must be CE marked or comply with equivalent standards if the blood and blood components are collected in third countries. The batch number of the blood bag must be traceable for each blood component (Directive 2005/62/EC Annex 6.2.2).

Blood collection procedures must minimise the risk of microbial contamination (Directive 2005/62/EC Annex 6.2.3).

Sterile collection systems must be used for the collection of blood and blood components. These must be used in accordance with the instructions of the manufacturer. A check must be made before use to ensure that the collection system employed has not been damaged or contaminated, and that it is appropriate for the intended collection procedure. Defects in blood bags must be reported to the supplier and be subjected to trend analysis.

3. Pre-donation checks

The blood container must be inspected before use for defects and must be inspected for the prescribed content and appearance of the anti-coagulant solution. If one or more bags in any package is found to be abnormally damp, all the bags in the package must be rejected.

The donor must be re-identified immediately prior to venepuncture.

4. Labelling

Laboratory samples must be taken at the time of donation and be appropriately stored prior to testing (Directive 2005/62/EC Annex 6.2.4).

The procedure used for the labelling of records, blood bags and laboratory samples with donation numbers must be designed to avoid any risk of identification error and mix-up (Directive 2005/62/EC Annex 6.2.5).

Laboratory samples (blood or plasma) must be taken at the time of each donation. Procedures must be designed to minimise the risk of

microbial contamination of the collected blood or deterioration of the sample, and to prevent potential misidentification of donations and samples.

At the time of blood donation, the blood container and those of the samples collected for testing must be labelled to uniquely identify the blood donation. The labelling system must comply with relevant national legislation and international agreements.

The blood donation must be identified by a unique identity number which is both eye- and machine-readable. The labelling system must allow full traceability to all relevant data registered by the blood establishment about the donor and the blood donation.

A careful check must be made of the identity indicator of the donor against the labels issued for that donation.

The manufacturer's label on the collection containers must contain the following eye-readable information:

- the manufacturer's name and address;
- the name of the collection container and/or the name of the plastic material;
- the name, composition and volume of anti-coagulant or additive solution (if any);
- the product catalogue number and the lot number.

5. Venepuncture, bleeding and mixing

Preparation of the venepuncture site

The skin at the venepuncture site must be free from lesions, including eczema.

The venepuncture site must be prepared using a defined and validated disinfection procedure. The antiseptic solution must be allowed to dry completely before venepuncture. The prepared area must not be touched with fingers before the needle has been inserted.

The effectiveness of the disinfection procedure must be monitored and corrective action taken where it is indicated to be defective.

Successful venepuncture and proper mixing

The needle must be inserted into the vein at the first attempt. A second venepuncture with a new needle in the other arm is acceptable with the consent of the donor, provided that bacterial sterility of the system is not compromised.

Where an anticoagulant solution is used in the collection, the collection bag must be mixed gently immediately after starting collection and at regular intervals during the entire collection period. The flow of blood must be sufficient and uninterrupted.

The maximum collection time for acceptance of the donation for component processing must be specified and controlled. Donations that exceed the maximum time period must be recorded and discarded.

If the duration of the bleeding is longer than 12 minutes, the blood must not be used for the preparation of platelets.

If the duration of the bleeding is longer than 15 minutes, the plasma must not be used for direct transfusion or the preparation of coagulation factors.

If manual mixing is used, the blood bag must be inverted every 30-45 seconds. When an automated mixing system is used, an appropriately validated system is required.

At completion of the donation, the donation number must be checked on all records, blood bags and laboratory samples. Donation number labels of a given donation that have not been used must be destroyed via a controlled procedure. Procedures to prevent mislabelling must be in place.

Each activity associated with the donation must be recorded. This also applies to any unsuccessful donations, the rejection of a donor, adverse reactions and adverse events. An authorised interviewer must sign the donor selection records and the final assessment.

6. Handling of filled containers and samples

After blood collection, the blood bags must be handled in a way that maintains the quality of the blood and at a storage and transport temperature appropriate to further processing requirements (Directive 2005/62/EC Annex 6.2.6).

There must be a system in place to ensure that each donation can be linked to the collection and processing system into which it was collected and/or processed (Directive 2005/62/EC Annex 6.2.7).

The blood container must be checked after donation for any defect. During separation from the donor, a completely efficient method of sealing the tube is obligatory.

The blood bag and corresponding samples must not be removed from the donor's bedside until labelling has been checked and is verified as correct.

After collection, blood bags must be placed promptly into controlled temperature storage and transported to the processing site under temperature conditions appropriate for the component that is to be prepared. Validation data must be available to demonstrate that the storage parameters after collection and the method of transport used maintains the blood within the specified temperature range throughout the period of transportation.

7. Special requirements for apheresis

Separation and collection of blood components by cell separators requires premises of suitable size, regular servicing and maintenance of machines (and adequately trained personnel for operating such machines).

The donor must be observed closely during the procedure. A qualified healthcare professional familiar with all aspects of apheresis must be available to provide assistance and emergency medical-care procedures in case of an adverse reaction.

Collection of adequate granulocyte yields by apheresis requires pre-medication of the donor. The potential risk to the donor must be evaluated against the anticipated benefit to the intended recipient.

Return of red blood cells of donors undergoing manual apheresis

Since the biggest inherent danger in manual apheresis is an accidental interchange between two bags of concentrated red blood cells during their centrifugation and return to individual donors, a robust identification system must be in place.

8. Repository of archive samples

If archive samples from donations are kept, then procedures must be in place prescribing their use and final disposal.

Chapter 4

Standards for the processing, storage and distribution of blood components

1. Processing

All equipment and technical devices must be used in accordance with validated procedures (Directive 2005/62/EC Annex 6.4.1).

The processing of blood components must be carried out using appropriate and validated procedures, including measures to avoid the risk of contamination and microbial growth in the prepared blood components (Directive 2005/62/EC Annex 6.4.2).

The premises used for the processing of blood components must be kept in a clean and hygienic condition and the microbial contamination load on critical equipment, surfaces and in the environment of the processing areas must be monitored.

Procedures must detail the specifications for any materials that will influence the quality of the final blood component. In particular, specifications must be in place for blood and blood components (intermediate and final components), starting materials, additive solutions, primary packaging material (bags) and equipment.

Procedures must be developed and validated for all processing activities. These must include time and temperature limits for the processing of blood components.

Sterile connecting devices must be used in accordance with a validated procedure. The resulting weld must be checked for satisfactory alignment and its integrity must be validated. When validated, connections made using sterile connecting devices are regarded as closed system processing.

2. Component labelling and information

At all stages, all containers must be labelled with relevant information of their identity. In the absence of a validated computerised system for status control, the labelling must clearly distinguish released from non-released units of blood and blood components (Directive 2005/62/EC Annex 6.5.1).

The labelling system for the collected blood, intermediate and finished blood components and samples must unmistakably identify the type of content and comply with the labelling and traceability requirements referred to in Article 14 of Directive 2002/98/EC and Directive 2005/61/EC. The label for a final blood component must comply with the requirements of Annex III to Directive 2002/98/EC (Directive 2005/62/EC Annex 6.5.2).

For autologous blood and blood components, the label also must comply with Article 7 of Directive 2004/33/EC and the additional requirements for autologous donations specified in Annex IV to that Directive (Directive 2005/62/EC Annex 6.5.3).

Before use, all blood components must be labelled with relevant identity information. The type of label to be used, as well as the labelling methodology, must be established in written procedures. Critical information must be provided in machine-readable format to eliminate transcription errors.

The blood establishment responsible for the preparation of blood components must provide clinical users of blood components with information on their use, composition and any special conditions that do not appear on the component label.

3. Release of blood components

There must be a safe and secure system to prevent each single blood and blood component from being released until all mandatory requirements set out in Directive 2005/62/EC have been fulfilled. Each blood establishment must be able to demonstrate that each blood or blood component has been formally released by an authorised person. Records must demonstrate that before a blood component is released, all current declaration forms, relevant medical records and test results meet all acceptance criteria (Directive 2005/62/EC Annex 6.6.1).

Before release, blood and blood components must be kept administratively and physically segregated from released blood and blood components. In the absence of a validated computerised system for status control, the label of a unit of blood or blood component must clearly distinguish released from non-released units of blood and blood components (Directive 2005/62/EC Annex 6.5.1 and 6.6.2).

Each blood establishment must be able to demonstrate that a blood or blood component has been approved for release by an authorised person, preferably assisted by validated information technology systems. The specifications for release of blood components must be defined, validated and documented.

Where release is subject to computer-derived information, the following requirements must be met:

- the computer system must be validated to be fully secure against the possibility of blood and blood components being released that do not fulfil all test or donor selection criteria;
- the manual entry of critical data, such as laboratory test results, must require independent verification by a second authorised person;
- the computer system must block the release of all blood or blood components considered not acceptable for release. There must also be a means to block the release of any future donations from the donor.

In the absence of a computerised system for component status control, or in the event of computer system failure, the following requirements must be met:

- the label of a blood component must identify the component status and must clearly distinguish a released from a non-released (quarantined) component;
- records must demonstrate that before a component is released, all current donor declaration forms, relevant medical records and test results have been verified by an authorised person;
- before final component release, if blood or blood component(s) have been prepared from a donor who has donated on previous occasions, a comparison with previous records must be made to ensure that current records accurately reflect the donor history;
- there must be a system of administrative and physical quarantine for blood and blood components to ensure that they cannot be released until all mandatory requirements have been satisfied.

In the event that the final component fails release due to a confirmed positive infection test result for Hepatitis B virus, Hepatitis C virus or HIV 1/2 (Annex IV of Directive 2002/98/EC), a check must be made to ensure that other components from the same donation and components prepared from previous donations given by the donor are identified. There must be an immediate update of the donor record (Directive 2005/62/EC Annex 6.6.3, 6.3.2 and 6.3.3)

In the event that a final component fails release due to a potential impact on patient safety, then all other implicated components must be identified and appropriate action must be taken. A check must be made to ensure that (if relevant) other components from the same donation(s) and components prepared from previous donations given by the donor(s) are identified. The donor record must be updated immediately to ensure (if appropriate) that the donor(s) cannot make a further donation.

4. Storage and distribution

The quality system of the blood establishment must ensure that, for blood and blood components intended for the manufacture of medicinal products, the storage and distribution requirements comply with Directive 2003/94/EC (Directive 2005/62/EC Annex 7.1).

Procedures for storage and distribution must be validated to ensure blood and blood component quality during the entire storage period and to exclude mix-ups of blood components. All transportation and storage actions, including receipt and distribution, must be defined by written procedures and specifications (Directive 2005/62/EC Annex 7.2).

Storage and distribution routines must take place in a safe and controlled way in order to ensure component quality during the entire storage period and to avoid any risk of identification error and mix-up of blood components.

All transportation and storage actions, including receipt and distribution, must be defined by written procedures and specifications.

Storage conditions must be controlled, monitored and checked. Appropriate alarms must be present and regularly checked, and these checks must be recorded. Appropriate actions on alarms must be defined.

Intermediate storage and transport must be carried out under defined conditions to ensure that the specified requirements are met.

Prior to distribution, blood components must be visually inspected. There must be a record identifying the person distributing the components and the institution receiving them.

Autologous blood and blood components, as well as blood components collected and prepared for specific purposes, must be stored separately (Directive 2005/62/EC Annex 7.3).

Storage areas must provide effective segregation of quarantined and released materials or components. There must be a separate area for storage of rejected components and materials.

Appropriate records of inventory and distribution must be kept (Directive 2005/62/EC Annex 7.4).

Packaging must maintain the integrity and storage temperature of blood or blood components during distribution and transportation (Directive 2005/62/EC Annex 7.5).

Return of blood and blood components into inventory for subsequent re-issue can only be accepted when all quality requirements and procedures laid down by the blood establishment to ensure blood component integrity are fulfilled (Directive 2005/62/ EC Annex 7.6).

Blood components must not be returned to the blood establishment for subsequent distribution, unless there is a procedure for return of blood components that is regulated by a contract and that there is documented evidence for each returned blood component that the agreed storage conditions have been met. Before subsequent distribution the records must identify that the blood component has been inspected before reissue.

5. Irradiation of blood components

The irradiation process must ensure that no part of the component receives a dose less than 25 Gray or more than 50 Gray. The exposure time must be set to ensure that all blood and blood components receive the specified recommended minimum dose, with no part receiving more than the maximum recommended dose. Regular dose-mapping of equipment must be undertaken. Exposure time must be standardised for each irradiation source and revalidated at suitable intervals.

Radiation indicators must be used as an aid to differentiating irradiated from non-irradiated blood and blood components. A defined procedure must ensure the segregation of components that have not been irradiated from those that have been irradiated.

Red cell components may be irradiated up to 28 days after collection. Irradiated cells must be transfused as soon as possible, but no later than 14 days after irradiation and, in any case, no later than 28 days after collection. More stringent requirements are included in specific component monographs (see Chapter 5).

6. Leucocyte depletion

Processes used for leucocyte depletion must be validated. The validation must be carried out by the blood establishment using the manufacturer's instructions and against the requirements for leucocyte depletion and other quality aspects of the components (including those for plasma for fractionation).

For quality control, an appropriate validated method must be used for counting leucocytes.

7. Bacterial safety

A systematic programme to assure the bacterial safety of blood collection and processing procedures must be in place.

Chapter 5

Component monographs

Part A. Whole Blood components

1. Whole Blood, 280

2. Whole Blood, Leucocyte-Depleted, 283

Part B. Red cell components

1. Red Cells, 290

2. Red Cells, Buffy Coat Removed, 293

3. Red Cells, in Additive Solution, 297

4. Red Cells, Buffy Coat Removed, in Additive Solution, 300

5. Red Cells, Leucocyte-Depleted, 304

6. Red Cells, Leucocyte-Depleted in Additive Solution, 307

7. Red Cells, Apheresis, 311

8. Red Cells, Washed, 315

9. Red Cells, Cryopreserved, 318

Part C. Platelet components

1. Platelets, Recovered, Single Unit, 326

2. Platelets, Recovered, Pooled, 330

3. Platelets, Recovered, Pooled, Leucocyte-Depleted, 334

4. Platelets, Recovered, Pooled, in Additive Solution, 339

5. Platelets, Recovered, Pooled, Leucocyte-Depleted, in Additive Solution, 343

6. Platelets, Pooled, Pathogen-reduced, 347

7. Platelets, Apheresis, 351

8. Platelets, Apheresis, Leucocyte-Depleted, 355

9. Platelets, Apheresis, in Additive Solution, 359

10. Platelets, Apheresis, Leucocyte-Depleted, in Additive Solution, 363

11. Platelets, Apheresis, Pathogen-reduced, 367

12. Platelets, Cryopreserved, 371

Part D. Plasma components

1. Plasma, Fresh Frozen, 378

2. Plasma, Fresh Frozen, Pathogen Reduced, 382

3. Cryoprecipitate, 387

4. Cryoprecipitate, Pathogen Reduced, 391

5. Plasma, Fresh Frozen, Cryoprecipitate-Depleted, 396

Part E. White cell components

1. Granulocytes, Apheresis, 400

2. Granulocytes, Pooled, 404

Component monographs
Part A. Whole Blood components

1. Whole Blood

Definition and properties

Whole Blood is blood taken from a suitable donor using a sterile and pyrogen-free anticoagulant and container. *Whole Blood* is a source material for component preparation, which is its major use. *Whole Blood* for transfusion is used without further processing.

Whole Blood for transfusion should not contain irregular antibodies of clinical significance.

Preparation

By definition, no (post-donation) preparation is required to produce a unit of *Whole Blood*.

Requirements and quality control

Table 5A-1 lists the requirements for whole blood for direct transfusion. Additional testing might be required to comply with national requirements (see also Chapter 9, Standards for screening for infectious markers).

Table 5A-1

Parameter to be checked	Requirements	Frequency of control
ABO, RhD	Grouping	All units
Anti-HIV 1 & 2	Negative by approved screening test	All units
HBsAg	Negative by approved screening test	All units
Anti-HCV	Negative by approved screening test	All units

Parameter to be checked	Requirements	Frequency of control
Volume [a]	450 mL ± 50 mL volume (excluding anticoagulant)<br>A non-standard donation should be labelled accordingly	as determined by SPC
Haemoglobin [a]	Minimum 45 g per unit	as determined by SPC
Haemolysis at the end of storage [a]	< 0.8 % of red cell mass	as determined by SPC

a A minimum of 90 % of units tested should meet the required value.

Storage and transport

Whole Blood for transfusion must be kept at a controlled temperature, i.e. between + 2 and + 6 °C. The storage time depends on the anti-coagulant/preservative solution used. For example, the storage time is 35 days if stored in CPDA-1. Validated transport systems must ensure that at no time during a maximum transit time of 24 hours does the temperature exceed + 10 °C.

Whole Blood for preparation of blood components may be kept up to 24 hours in conditions validated to maintain a temperature between + 20 °C and + 24 °C, which is a prerequisite for the production of platelet preparations from *Whole Blood*.

Labelling

The labelling must comply with the relevant national legislation and international agreements. The following information must be shown on the label or contained in the component information leaflet, as appropriate:

- the producer's identification;
- the unique identity number;
- the name of the blood component;
- the ABO and RhD groups;
- blood group phenotypes other than ABO and RhD (optional);

- the date of donation;
- the date of expiry;
- the name of the anticoagulant solution;
- additional component information: irradiated, etc. (if appropriate);
- the volume or weight of the blood component;
- the storage temperature;
- that the component must not be used for transfusion if there is abnormal haemolysis or other deterioration;
- that the component must be administered through an approved blood administration set.

Warnings

Compatibility of *Whole Blood* for transfusion with the intended recipient must be verified by suitable pre-transfusion testing.

RhD-negative female recipients of child-bearing age or younger should not be transfused with *Whole Blood* from RhD-positive donors.

Micro-aggregates are formed on storage.

Whole Blood for transfusion is not recommended in cases of:

- anaemia without blood volume loss;
- plasma intolerance;
- intolerance due to allo-immunisation against leucocyte antigens.

Adverse reactions include:

- haemolytic transfusion reaction;
- non-haemolytic transfusion reaction (mainly chills, fever and urticaria);
- anaphylaxis;
- allo-immunisation against red cell and HLA antigens;

- transfusion-related acute lung injury (TRALI);
- post-transfusion purpura;
- graft versus host disease (TA-GvHD);
- sepsis due to inadvertent bacterial contamination;
- viral transmission (hepatitis, HIV, etc.) is possible, despite careful donor selection and screening procedures;
- syphilis can be transmitted if components are stored for less than 96 hours at + 4 °C;
- protozoal transmission (e.g. malaria) may occur in rare instances;
- transmission of other pathogens that are not tested for or recognised;
- citrate toxicity in neonates and in patients with impaired liver function;
- metabolic imbalance in massive transfusion (e.g. hyperkalaemia);
- transfusion-associated circulatory overload;
- iron overload.

2. Whole Blood, Leucocyte-Depleted

Definition and properties

Whole Blood, Leucocyte-Depleted (LD) is a component derived from *Whole Blood* by removing the leucocytes to a maximum residual content.

Whole Blood, LD contains a minimum haemoglobin content of 43 g.

Whole Blood, LD normally contains less than 1.0×10^6 leucocytes.

Preparation

Generally a filtration technique is used to produce *Whole Blood, LD*. Pre-storage leucocyte depletion within 48 hours after donation is the standard.

Requirements and quality control

Table 5A-2 lists the requirements. Additional testing may be required to comply with national requirements (see also Chapter 9, Standards for screening for infectious markers).

Table 5A-2

Parameter to be checked	Requirements	Frequency of control
ABO, RhD	Grouping	All units
Anti-HIV 1 & 2	Negative by approved screening test	All units
HbsAg	Negative by approved screening test	All units
Anti-HCV	Negative by approved screening test	All units
Volume [a]	450 ± 50 mL volume (excluding anticoagulant<br>A non-standard donation should be labelled accordingly	as determined by SPC
Haemoglobin [a]	Minimum 43 g per unit	as determined by SPC
Residual leucocytes content [a]	$< 1 \times 10^6$ per unit by count	as determined by SPC
Haemolysis at the end of storage [a]	< 0.8 % of red cell mass	as determined by SPC

a A minimum of 90 % of units tested should meet the required value.

Storage and transport

Whole Blood, LD must be kept at a controlled temperature between + 2 °C and + 6 °C. The storage time depends on the anticoagulant/preservative solution used. For example, the storage time is 35 days in CPDA-1.

Validated transport systems must ensure that at no time during a maximum transit time of 24 hours did the temperature exceed + 10 °C.

Labelling

The labelling must comply with the relevant national legislation and international agreements. The following information must be shown on the label or contained in the component information leaflet, as appropriate:

- the producer's identification;
- the unique identity number;
- the name of the blood component;
- the ABO and RhD groups;
- blood group phenotypes other than ABO and RhD (optional);
- the date of donation;
- the date of expiry;
- the name of the anticoagulant solution;
- additional component information: irradiated, etc. (if appropriate);
- the volume or weight of the blood component;
- the storage temperature;
- that the component must not be used for transfusion if there is abnormal haemolysis or other deterioration;
- that the component must be administered through an approved blood administration set.

Warnings

Compatibility of *Whole Blood, LD* with the intended recipient must be verified by suitable pre-transfusion testing.

RhD-negative female recipients of child-bearing age or younger should not be transfused with red cells from RhD-positive donors.

Whole Blood, LD is not recommended in cases of:

- anaemia without blood volume loss;
- plasma intolerance.

Adverse reactions include:

- haemolytic transfusion reaction;
- non-haemolytic transfusion reaction (mainly chills, fever and urticaria);
- anaphylaxis;
- allo-immunisation against red cell and HLA antigens;
- transfusion-related acute lung injury (TRALI);
- post-transfusion purpura;
- graft versus host disease (TA-GvHD);
- sepsis due to inadvertent bacterial contamination;
- viral transmission (hepatitis, HIV, etc.) is possible, despite careful donor selection and screening procedures;
- syphilis can be transmitted if components are stored for less than 96 hours at + 4 °C;
- protozoal transmission (e.g. malaria) may occur in rare instances;
- transmission of other pathogens that are not tested for or recognised;
- citrate toxicity in neonates and in patients with impaired liver function;

- metabolic imbalance in massive transfusion (e.g. hyperkalaemia);
- circulatory overload;
- iron overload.

Component monographs
Part B. Red cell components

1. Red Cells

Definition and properties

Red Cells is a component obtained by removal of a major part of the plasma from *Whole Blood*.

Red Cells also contain the greater part of the Whole Blood leucocytes (about 2.5 to 3.0 × 10^9 cells) and a variable content of platelets, depending on the method of centrifugation.

Preparation

For the preparation of *Red Cells*, plasma is removed from *Whole Blood* by centrifugation.

Requirements and quality control

Table 5B-1 lists the requirements. Additional testing may be required to comply with national requirements (see also Chapter 9, Standards for screening for infectious markers).

Table 5B-1

Parameter to be checked	Requirements	Frequency of control
ABO, RhD	Grouping	All units
Anti-HIV 1 & 2	Negative by approved screening test	All units
HBsAg	Negative by approved screening test	All units
Anti-HCV	Negative by approved screening test	All units
Volume [a]	280 mL ± 50 mL	as determined by SPC
Haematocrit [a]	0.65 – 0.75	as determined by SPC

a A minimum of 90 % of units tested should meet the required value.

Parameter to be checked	Requirements	Frequency of control
Haemoglobin[a]	Minimum 45 g per unit	as determined by SPC
Haemolysis at the end of storage [a]	< 0.8 % of red cell mass	as determined by SPC

a A minimum of 90 % of units tested should meet the required value.

Storage and transport

Red Cells must be kept at a controlled temperature between + 2 °C and + 6 °C. The storage time depends on the anticoagulant/preservative solution used. For example, the storage time is 35 days in CPDA-1.

Validated transport systems must ensure that at no time during a maximum transit time of 24 hours did the temperature exceed + 10 °C.

Labelling

The labelling must comply with the relevant national legislation and international agreements. The following information must be shown on the label or contained in the component information leaflet, as appropriate:

- the producer's identification;
- the unique identity number;
- the name of the blood component;
- the ABO and RhD groups;
- blood group phenotypes other than ABO and RhD (optional);
- the date of donation;
- the date of expiry;
- the name of the anticoagulant solution;
- additional component information: irradiated, etc. (if appropriate);
- the volume or weight of the blood component;
- the storage temperature;

- that the component must not be used for transfusion if there is abnormal haemolysis or other deterioration;
- that the component must be administered through an approved blood administration set.

Warnings

Micro-aggregates are formed on storage.

Compatibility of *Red Cells* with the intended recipient must be verified by suitable pre-transfusion testing.

RhD-negative female recipients of child-bearing age or younger should not be transfused with *Red Cells* from RhD-positive donors.

Red Cells are not recommended in cases of:

- plasma intolerance;
- intolerance due to allo-immunisation against leucocyte antigens;
- exchange transfusion in newborns, unless supplementary plasma is added.

Adverse reactions include:

- haemolytic transfusion reaction;
- non-haemolytic transfusion reaction (mainly chills, fever and urticaria);
- anaphylaxis;
- allo-immunisation against red cell and HLA antigens;
- transfusion-related acute lung injury (TRALI);
- post-transfusion purpura;
- graft versus host disease (TA-GvHD);
- sepsis due to inadvertent bacterial contamination;
- viral transmission (hepatitis, HIV, etc.) is possible, despite careful donor selection and screening procedures;

- syphilis can be transmitted if components are stored for less than 96 hours at + 4 °C;
- protozoal transmission (e.g. malaria) may occur in rare instances;
- transmission of other pathogens that are not tested for or recognised;
- citrate toxicity in neonates and in patients with impaired liver function;
- metabolic imbalance in massive transfusion (e.g. hyperkalaemia);
- transfusion-associated circulatory overload;
- iron overload.

2. Red Cells, Buffy Coat Removed

Definition and properties

Red Cells, Buffy Coat Removed (BCR) is a red cell component prepared by the removal of a major part of the plasma and the buffy coat layer from *Whole Blood*.

Red Cells, BCR contains a minimum haemoglobin content of 43 g. The haematocrit is 0.65 to 0.75.

Red Cells, BCR normally contains less than 1.2×10^9 leucocytes and a variable content of platelets, depending on the method of centrifugation.

Preparation

Red Cells, BCR is derived from *Whole Blood* by centrifugation. The plasma and 20 to 60 mL of the buffy coat layer are removed from *Whole Blood* after centrifugation, resulting in the loss of 10 to 30 mL of the red cells from the donated *Whole Blood*. Sufficient plasma is retained to give a haematocrit of 0.65-0.75.

Requirements and quality control

Table 5B-2 lists the requirements. Additional testing may be required to comply with national requirements (see also Chapter 9, Standards for screening for infectious markers).

Table 5B-2

Parameter to be checked	Requirements	Frequency of control
ABO, RhD	Grouping	All units
Anti-HIV 1 & 2	Negative by approved screening test	All units
HBsAg	Negative by approved screening test	All units
Anti-HCV	Negative by approved screening test	All units
Volume [a]	250mL ± 50 mL	as determined by SPC
Haematocrit [a]	0.65 – 0.75	as determined by SPC
Haemoglobin [a]	Minimum 45 g per unit	as determined by SPC
Residual leucocyte content [a]	$< 1.2 \times 10^9$ per unit	as determined by SPC
Haemolysis at the end of storage [a]	< 0.8 % of red cell mass	as determined by SPC

a A minimum of 90 % of units tested should meet the required value.

Storage and transport

Red Cells, BCR must be kept at a controlled temperature between + 2 °C and + 6 °C. The storage time depends on the anticoagulant/preservative solution used. For example, the storage time is 35 days in CPDA-1.

Validated transport systems must ensure that at no time during a maximum transit time of 24 hours did the temperature exceed + 10 °C.

Labelling

The labelling must comply with the relevant national legislation and international agreements. The following information must be shown on the label or contained in the component information leaflet, as appropriate:

- the producer's identification;
- the unique identity number;
- the name of the blood component;
- the ABO and RhD groups;
- blood group phenotypes other than ABO and RhD (optional);
- the date of donation;
- the date of expiry;
- the name of the anticoagulant solution;
- additional component information: irradiated, etc. (if appropriate);
- the volume or weight of the blood component;
- the storage temperature;
- that the component must not be used for transfusion if there is abnormal haemolysis or other deterioration;
- that the component must be administered through an approved blood administration set.

Warnings

Compatibility of *Red Cells, BCR* with the intended recipient must be verified by suitable pre-transfusion testing.

RhD-negative female recipients of child-bearing age or younger should not be transfused with red cells from RhD-positive donors.

Red Cells, BCR is not recommended in cases of:

- plasma intolerance (may not concern units with a low plasma content, unless IgA incompatibility is present);

- exchange transfusions in newborns, unless supplementary plasma is added.

Adverse reactions include:

- haemolytic transfusion reaction;
- non-haemolytic transfusion reaction (mainly chills, fever and urticaria);
- anaphylaxis;
- allo-immunisation against red cell and HLA antigens;
- transfusion-related acute lung injury (TRALI);
- post-transfusion purpura;
- graft versus host disease (TA-GvHD);
- sepsis due to inadvertent bacterial contamination;
- viral transmission (hepatitis, HIV, etc.) is possible, despite careful donor selection and screening procedures;
- syphilis can be transmitted if components are stored for less than 96 hours at + 4 °C;
- protozoal transmission (e.g. malaria) may occur in rare instances;
- transmission of other pathogens that are not tested for or recognised;
- citrate toxicity in neonates and in patients with impaired liver function;
- metabolic imbalance in massive transfusion (e.g. hyperkalaemia);
- transfusion-associated circulatory overload;
- iron overload.

3. Red Cells, in Additive Solution

Definition and properties

Red Cells, in Additive Solution (AS) is a red cell component prepared by the removal of the plasma from *Whole Blood* with subsequent addition of an appropriate additive solution.

Red Cells, AS contains a minimum haemoglobin content of 45 g. The haematocrit is 0.50-0.70.

Red Cells, AS also contains the greater part of the *Whole Blood* leucocytes (about 2.5 to 3.0 × 10^9 cells) and a variable content of platelets, depending on the method of centrifugation.

Preparation

Whole Blood is collected, using CPD as the anticoagulant solution. After centrifugation of *Whole Blood*, plasma is removed and the additive solution is added immediately to red cells and mixed carefully.

Requirements and quality control

Table 5B-3 lists the requirements. Additional testing may be required to comply with national requirements (see also Chapter 9, Standards for screening for infectious markers).

Table 5B-3

Parameter to be checked	Requirements	Frequency of control
ABO, RhD	Grouping	All units
Anti-HIV 1 & 2	Negative by approved screening test	All units
HBsAg	Negative by approved screening test	All units
Anti-HCV	Negative by approved screening test	All units

Parameter to be checked	Requirements	Frequency of control
Volume [a]	To be defined for the system used	as determined by SPC
Haematocrit [a]	0.50-0.70	as determined by SPC
Haemoglobin [a]	Minimum 45 g per unit	as determined by SPC
Haemolysis at the end of storage [a]	< 0.8 % of red cell mass	as determined by SPC

a A minimum of 90 % of units tested should meet the required value.

Storage and transport

Red Cells, AS must be kept at a controlled temperature between + 2 and + 6 °C during storage. Depending on the anticoagulant/additive system, the storage time may be extended up to the approved limit of the additive solution system.

Validated transport systems must ensure that at no time during a maximum transit time of 24 hours did the temperature exceed + 10 °C.

Labelling

The labelling must comply with the relevant national legislation and international agreements. The following information must be shown on the label or contained in the component information leaflet, as appropriate:

- the producer's identification;
- the unique identity number;
- the name of the blood component;
- the ABO and RhD groups;
- blood group phenotypes other than ABO and RhD (optional);
- the date of donation;
- the date of expiry;
- the name of the anticoagulant solution;

- the name and volume of the additive solution;
- additional component information: irradiated, etc. (if appropriate);
- the volume or weight of the blood component;
- the storage temperature;
- that the component must not be used for transfusion if there is abnormal haemolysis or other deterioration;
- that the component must be administered through an approved blood administration set.

Warnings

Micro-aggregates are formed on storage.

Compatibility of *Red Cells, AS* with the intended recipient must be verified by suitable pre-transfusion testing.

RhD-negative female recipients of child-bearing age or younger should not be transfused with *Red Cells, AS* from RhD-positive donors.

Red Cells, AS is not recommended in cases of:

- plasma intolerance;
- intolerance due to allo-immunisation against leucocyte antigens;
- exchange transfusion in newborns, unless used within 5 days of donation and only if the additive solution is replaced by fresh frozen plasma on the day of use.

Adverse reactions include:

- haemolytic transfusion reaction;
- non-haemolytic transfusion reaction (mainly chills, fever and urticaria);
- anaphylaxis;
- allo-immunisation against red cell and HLA antigens;

- transfusion-related acute lung injury (TRALI);
- post-transfusion purpura;
- graft versus host disease (TA-GvHD);
- sepsis due to inadvertent bacterial contamination;
- viral transmission (hepatitis, HIV, etc.) is possible, despite careful donor selection and screening procedures;
- syphilis can be transmitted if components are stored for less than 96 hours at + 4 °C;
- protozoal transmission (e.g. malaria) may occur in rare instances;
- transmission of other pathogens that are not tested for or recognised;
- citrate toxicity in neonates and in patients with impaired liver function;
- metabolic imbalance in massive transfusion (e.g. hyperkalaemia);
- transfusion-associated circulatory overload;
- iron overload.

4. Red Cells, Buffy Coat Removed, in Additive Solution

Definition and properties

Red Cells, Buffy Coat Removed, in Additive Solution (BCR-AS) is a red cell component prepared by the removal of a major part of the plasma and the buffy coat layer from *Whole Blood*, with subsequent addition of an appropriate nutrient solution.

Red Cells, BCR-AS contains a minimum haemoglobin content of 43 g. The haematocrit is 0.50 to 0.70.

Red Cells, BCR-AS contains less than 1.2×10^9 leucocytes and a variable platelet content, depending on the method of centrifugation.

Preparation

Red Cells, BCR-AS is derived from *Whole Blood* by centrifugation. For preparation, the plasma and 20 to 60 mL of the buffy coat layer are removed, resulting in the loss of 10 to 30 mL of the red cells from the donated *Whole Blood*. The additive solution is immediately added to the red cells and carefully mixed.

Requirements and quality control

Table 5B-4 lists the requirements. Additional testing may be required to comply with national requirements (see also Chapter 9, Standards for screening for infectious markers).

Table 5B-4

Parameter to be checked	Requirements	Frequency of control
ABO, RhD	Grouping	All units
Anti-HIV 1 & 2	Negative by approved screening test	All units
HBsAg	Negative by approved screening test	All units
Anti-HCV	Negative by approved screening test	All units
Volume [a]	To be defined for the system used	as determined by SPC
Haematocrit [a]	0.50-0.70	as determined by SPC
Haemoglobin [a]	Minimum 43 g per unit	as determined by SPC
Residual leucocyte content [a]	$< 1.2 \times 10^9$ per unit	as determined by SPC
Haemolysis at the end of storage [a]	< 0.8 % of red cell mass	as determined by SPC

a A minimum of 90 % of units tested should meet the required value.

Storage and transport

Red Cells, BCR-AS must be kept at a controlled temperature between + 2 and + 6 °C during storage. Depending on the anticoagulant/additive system, storage time may be extended up to the approved limit of the additive solution system.

Validated transport systems must ensure that at no time during a maximum transit time of 24 hours did the temperature exceed + 10 °C.

Labelling

The labelling must comply with the relevant national legislation and international agreements. The following information must be shown on the label or contained in the component information leaflet, as appropriate:

- the producer's identification;
- the unique identity number;
- the name of the blood component;
- the ABO and RhD groups;
- blood group phenotypes other than ABO and RhD (optional);
- the date of donation;
- the date of expiry;
- the name of the anticoagulant solution;
- the name and volume of the additive solution;
- additional component information: irradiated, etc. (if appropriate);
- the volume or weight of the blood component;
- the storage temperature;
- that the component must not be used for transfusion if there is abnormal haemolysis or other deterioration;

- that the component must be administered through an approved blood administration set.

Warnings

Compatibility of *Red Cells, BCR-AS* with the intended recipient must be verified by suitable pre-transfusion testing.

RhD-negative female recipients of child-bearing age or younger should not be transfused with red cells from RhD-positive donors.

Red Cells, BCR-AS is not recommended in cases of:

- plasma intolerance;
- intolerance due to allo-immunisation against leucocyte antigens.

Adverse reactions include:

- haemolytic transfusion reaction;
- non-haemolytic transfusion reaction (mainly chills, fever and urticaria);
- anaphylaxis;
- allo-immunisation against red cell and HLA antigens;
- transfusion-related acute lung injury (TRALI);
- post-transfusion purpura;
- graft versus host disease (TA-GvHD);
- sepsis due to inadvertent bacterial contamination;
- viral transmission (hepatitis, HIV, etc.) is possible, despite careful donor selection and screening procedures;
- syphilis can be transmitted if component is stored for less than 96 hours at + 4 °C;
- protozoal transmission (e.g. malaria) may occur in rare instances;
- transmission of other pathogens that are not tested for or recognised;

- citrate toxicity in neonates and in patients with impaired liver function;
- metabolic imbalance in massive transfusion (e.g. hyperkalaemia);
- transfusion-associated circulatory overload;
- iron overload.

5. Red Cells, Leucocyte-Depleted

Definition and properties

Red Cells, Leucocyte-Depleted (LD) is a red cell component derived from *Whole Blood* donation, *Red Cells* or *Red Cells, BCR* by removing the leucocytes.

Red Cells, LD contains a minimum haemoglobin content of 40 g. The haematocrit is 0.65-0.75.

Red Cells, LD contains less than 1.0×10^6 leucocytes.

Preparation

Generally a filtration technique is used to produce *Red Cells, LD*. Leucocyte depletion within 48 hours after donation is the standard.

Red Cells, LD can be produced:

- by leucocyte filtration of *Whole Blood*, with subsequent centrifugation and removal of the plasma;
- by leucocyte filtration of a red cell component.

Requirements and quality control

Table 5B-5 lists the requirements. Additional testing may be required to comply with national requirements (see also Chapter 9, Standards for screening for infectious markers).

Table 5B-5

Parameter to be checked	Requirements	Frequency of control
ABO, RhD	Grouping	All units
Anti-HIV 1 & 2	Negative by approved screening test	All units
HBsAg	Negative by approved screening test	All units
Anti-HCV	Negative by approved screening test	All units
Volume [a]	To be defined for the system used	as determined by SPC
Haematocrit [a]	0.65-0.75	as determined by SPC
Haemoglobin [a]	Minimum 40 g per unit	as determined by SPC
Residual leucocyte content [a]	$< 1 \times 10^6$ per unit	as determined by SPC
Haemolysis at the end of storage [a]	< 0.8 % of red cell mass	as determined by SPC

a A minimum of 90 % of units tested should meet the required value.

Storage and transport

Red Cells, LD must be kept at a controlled temperature between + 2 and + 6 °C during storage. Depending on the anticoagulant/additive system, the storage time may be extended up to the approved limit of the additive solution system.

Validated transport systems must ensure that at no time during a maximum transit time of 24 hours did the temperature exceed + 10 °C.

Labelling

The labelling must comply with the relevant national legislation and international agreements. The following information must be shown on the label or contained in the component information leaflet, as appropriate:

- the producer's identification;
- the unique identity number;
- the name of the blood component;
- the ABO and RhD groups;
- blood group phenotypes other than ABO and RhD (optional);
- the date of donation;
- the date of expiry;
- the name of the anticoagulant solution;
- the name and volume of the additive solution (if appropriate);
- additional component information: irradiated, etc. (if appropriate);
- the volume or weight of the blood component;
- the storage temperature;
- that the component must not be used for transfusion if there is abnormal haemolysis or other deterioration;
- that the component must be administered through an approved blood administration set.

Warnings

Compatibility of *Red Cells, LD* with the intended recipient must be verified by suitable pre-transfusion testing.

RhD-negative female recipients of child-bearing age or younger should not be transfused with red cells from RhD-positive donors.

Red Cells, LD is not recommended in the case of:

- plasma intolerance.

Potential adverse reactions include:

- transfusion-associated circulatory overload;
- haemolytic transfusion reaction;

- anaphylaxis;
- non-haemolytic transfusion reaction (mainly chills, fever and urticaria);
- allo-immunisation against red cell and HLA (very rarely) antigens;
- transfusion-related acute lung injury (TRALI);
- post-transfusion purpura;
- graft versus host disease (TA-GvHD);
- sepsis due to inadvertent bacterial contamination;
- viral transmission (hepatitis, HIV, etc.) is possible, despite careful donor selection and screening procedures;
- syphilis can be transmitted if components are stored for less than 96 hours at + 4 °C;
- protozoal transmission (e.g. malaria) may occur in rare instances;
- transmission of other pathogens that are not tested for or recognised;
- citrate toxicity in neonates and in patients with impaired liver function;
- metabolic imbalance in massive transfusion (e.g. hyperkalaemia);
- iron overload.

6. Red Cells, Leucocyte-Depleted in Additive Solution

Definition and properties

Red Cells, Leucocyte depleted in Additive Solution (LD-AS) is a red cell component derived from *Whole Blood* donation, from *Red Cells, AS* or *Red Cells, BCR-AS* by removing the leucocytes to a maximum residual content.

Red Cells, LD-AS contains a minimum haemoglobin content of 40 g. The haematocrit is 0.50 to 0.70.

Red Cells, LD-AS contains less than 1.0×10^6 leucocytes.

Preparation

Generally, a filtration technique is used to produce *Red Cells, LD-AS*. Leucocyte depletion within 48 hours after donation is the standard.

Red Cells, LD-AS can be produced:

- by leucocyte filtration of *Whole Blood*, with subsequent centrifugation and removal of the plasma and immediate addition of the additive solution, followed by careful mixing;
- by leucocyte filtration of *Red Cells, AS* or *Red Cells, BCR-AS*.

Requirements and quality control

Table 5B-6 lists the requirements. Additional testing may be required to comply with national requirements (see also Chapter 9, Standards for screening for infectious markers).

Table 5B-6

Parameter to be checked	Requirements	Frequency of control
ABO, RhD	Grouping	All units
Anti-HIV 1 & 2	Negative by approved screening test	All units
HBsAg	Negative by approved screening test	All units
Anti-HCV	Negative by approved screening test	All units
Volume [a]	To be defined for the system used	as determined by SPC
Haematocrit [a]	0.50-0.70	as determined by SPC

a A minimum of 90 % of units tested should meet the required value.

Parameter to be checked	Requirements	Frequency of control
Haemoglobin [a]	Minimum 40 g per unit	as determined by SPC
Residual leucocyte content [a]	$< 1 \times 10^6$ per unit	as determined by SPC
Haemolysis at the end of storage [a]	< 0.8 % of red cell mass	as determined by SPC

a A minimum of 90 % of units tested should meet the required value.

Storage and transport

Red Cells, LD-AS must be kept at a controlled temperature between + 2 and + 6 °C during storage. Depending on the anticoagulant/additive system, the storage time may be extended up to the approved limit of the additive solution system.

Validated transport systems must ensure that at no time during a maximum transit time of 24 hours did the temperature exceed + 10 °C.

Labelling

The labelling must comply with the relevant national legislation and international agreements. The following information must be shown on the label or contained in the component information leaflet, as appropriate:

- the producer's identification;
- the unique identity number;
- the name of the blood component;
- the ABO and RhD groups;
- blood group phenotypes other than ABO and RhD (optional);
- the date of donation;
- the date of expiry;
- the name of the anticoagulant solution;
- the name and volume of the additive solution;

- additional component information: irradiated, etc. (if appropriate);
- the volume or weight of the blood component;
- the storage temperature;
- that the component must not be used for transfusion if there is abnormal haemolysis or other deterioration;
- that the component must be administered through an approved blood administration set.

Warnings

Compatibility of *Red Cells, LD-AS* with the intended recipient must be verified by suitable pre-transfusion testing.

RhD-negative female recipients of child-bearing age or younger should not be transfused with red cells from RhD-positive donors.

Red Cells, LD-AS is not recommended in the case of:

- plasma intolerance (may not apply to units with a low plasma content).

Adverse reactions include:

- haemolytic transfusion reaction;
- non-haemolytic transfusion reaction (mainly chills, fever and urticaria);
- anaphylaxis;
- allo-immunisation against red cell and HLA (very rarely) antigens;
- transfusion-related acute lung injury (TRALI);
- post-transfusion purpura;
- graft versus host disease (TA-GvHD);
- sepsis due to inadvertent bacterial contamination;

- viral transmission (hepatitis, HIV, etc.) is possible, despite careful donor selection and screening procedures;
- syphilis can be transmitted if components are stored for less than 96 hours at + 4 °C;
- protozoal transmission (e.g. malaria) may occur in rare instances;
- transmission of other pathogens that are not tested for or recognised;
- citrate toxicity in neonates and in patients with impaired liver function;
- metabolic imbalance in massive transfusion (e.g. hyperkalaemia);
- transfusion-associated circulatory overload;
- iron overload.

7. Red Cells, Apheresis

Definition and properties

Red Cells, Apheresis (Aph) is a red cell component obtained by apheresis of a single donor using automated cell-separation equipment.

Red Cells, Aph contains a minimum haemoglobin content of 40 g. The haematocrit is 0.65 to 0.75 (0.50 to 0.70 if an additive solution is used).

The leucocyte content of *Red Cells, Aph* can vary. When leucocyte- depleted, *Red Cells, Aph* normally contains less than 1.0×10^6 leucocytes.

Preparation

For preparation of *Red Cells, Aph*, *Whole Blood* is removed by an appropriate apheresis machine from the donor and anti-coagulated with a citrate-containing solution. The plasma is returned to the donor. Either one or two units of *Red Cells, Aph* can be collected during a single procedure.

Red Cells, Aph can be used either unmodified or can undergo further processing, e.g. leucocyte depletion or addition of an additive solution.

Requirements and quality control

Table 5B-7 lists the requirements. Additional testing may be required to comply with national requirements (see also Chapter 9, Standards for screening for infectious markers).

Table 5B-7

Parameter to be checked	Requirements	Frequency of control
ABO, RhD	Grouping	All units
Anti-HIV 1 & 2	Negative by approved screening test	All units
HBsAg	Negative by approved screening test	All units
Anti-HCV	Negative by approved screening test	All units
Volume [a]	To be defined for the system used	as determined by SPC
Haematocrit [a]	0.65-0.75	as determined by SPC
Haematocrit [a] (if additive solution)	0.50-0.70	as determined by SPC
Haemoglobin [a]	Minimum 40 g per unit	as determined by SPC
Residual leucocyte content [a]	$< 1 \times 10^6$ per unit	as determined by SPC
Haemolysis at the end of storage [a]	< 0.8 % of red cell mass	as determined by SPC

a A minimum of 90 % of units tested should meet the required value.

Storage and transport

Red Cells, Aph must be kept at a controlled temperature between + 2 and + 6 °C during storage. Depending on the anticoagulant/additive system, the storage time may be extended up to the approved limit of the additive solution system.

Red Cells, Aph to be stored must be collected and prepared in a functionally closed system. If prepared or filtered by methods under an open system, the storage time is limited to 24 hours at + 2 and + 6 °C.

Validated transport systems must ensure that at no time during a maximum transit time of 24 hours did the temperature exceed + 10 °C.

Labelling

The labelling must comply with the relevant national legislation and international agreements. The following information must be shown on the label or contained in the component information leaflet, as appropriate:

- the producer's identification;
- the unique identity number. If 2 or more units are collected from the donor in one session, each component must have a unique component identity number;
- the name of the blood component;
- the ABO and RhD groups;
- blood group phenotypes other than ABO and RhD (optional);
- the date of donation;
- the date of expiry;
- the name of the anticoagulant solution;
- the name and volume of the additive solution (if appropriate);
- additional component information: irradiated, etc. (if appropriate);
- the volume or weight of the blood component;
- the storage temperature;
- that the component must not be used for transfusion if there is abnormal haemolysis or other deterioration;

- that the component must be administered through an approved blood administration set.

Warnings

Compatibility of *Red Cells, Aph* with the intended recipient must be verified by suitable pre-transfusion testing.

RhD-negative female recipients of child-bearing age or younger should not be transfused with red cells from RhD-positive donors.

Red Cells, Aph is not recommended in the case of:

- plasma intolerance (may not apply to units with a low plasma content unless IgA incompatibility is present).

Adverse reactions include:

- transfusion-associated circulatory overload;
- haemolytic transfusion reaction;
- anaphylaxis;
- non-haemolytic transfusion reaction (mainly chills, fever and urticaria);
- allo-immunisation against red cell and HLA (very rarely after leucocyte-depletion) antigens;
- transfusion-related acute lung injury (TRALI);
- post-transfusion purpura;
- graft versus host disease (TA-GvHD);
- sepsis due to inadvertent bacterial contamination;
- viral transmission (hepatitis, HIV, etc.) is possible, despite careful donor selection and screening procedures;
- syphilis can be transmitted if components are stored for less than 96 hours at + 4 °C;
- protozoal transmission (e.g. malaria) may occur in rare instances;

- transmission of other pathogens that are not tested for or recognised;
- citrate toxicity in neonates and in patients with impaired liver function;
- metabolic imbalance in massive transfusion (e.g. hyperkalaemia);
- iron overload.

8. Red Cells, Washed

Definition and properties

Red Cells, Washed (W) is derived from secondary processing of a red cell component or *Whole Blood* involving sequential washing and re-suspension of red cells in an additive solution.

Most of the plasma, leucocytes and platelets are removed. The amount of residual plasma depends upon the washing protocol. The haematocrit can be varied according to clinical need.

Preparation

After centrifugation of the primary component and removal of the plasma or additive solution (and, if applicable, the buffy coat layer), the red cells are washed by sequential addition and removal of an additive solution. Centrifugation must be performed at a controlled temperature.

Requirements and quality control

Table 5B-8 lists the requirements. Additional testing may be required to comply with national requirements (see also Chapter 9, Standards for screening for infectious markers).

Table 5B-8

Parameter to be checked	Requirements	Frequency of control
ABO, RhD	Grouping	All units
Anti-HIV 1 & 2	Negative by approved screening test	All units
HBsAg	Negative by approved screening test	All units
Anti-HCV	Negative by approved screening test	All units
Volume [a]	To be defined for the system used	as determined by SPC
Haematocrit [a]	0.50-0.70	as determined by SPC
Haemoglobin [a]	Minimum 40 g per unit	as determined by SPC
Residual leucocyte content [a]	$< 1 \times 10^6$ per unit	as determined by SPC
Haemolysis at the end of storage [a]	< 0.8 % of red cell mass	as determined by SPC
Protein content of final supernatant [a]	< 0.5 g per unit	as determined by SPC

a A minimum of 90 % of units tested should meet the required value.

Storage and transport

Red Cells, W must be kept at a controlled temperature between + 2 °C and + 6 °C during storage.

When an open system is used for washing, the storage time should be as short as possible after washing and must never exceed 24 hours.

If a closed system and a suitable additive solution are used, storage times may be prolonged, subject to validation.

Validated transport systems must ensure that at no time does the temperature exceed + 10 °C.

Labelling

The labelling must comply with the relevant national legislation and international agreements. The following information must be shown on the label or contained in the component information leaflet, as appropriate:

- the producer's identification;
- the unique identity number;
- the name of the blood component;
- the ABO and RhD groups;
- blood group phenotypes other than ABO and RhD (optional);
- the date of donation;
- the date and time of expiry;
- the name of the anticoagulant solution;
- the name and volume of the washing solution;
- additional component information: irradiated, etc. (if appropriate);
- the volume or weight of the blood component;
- the storage temperature;
- that the component must not be used for transfusion if there is abnormal haemolysis or other deterioration;
- that the component must be administered through an approved blood administration set.

Warnings

Compatibility of *Red Cells, W* with the intended recipient must be verified by suitable pre-transfusion testing.

RhD-negative female recipients of child-bearing age or younger should not be transfused with red cells from RhD-positive donors.

Adverse reactions include:

- haemolytic transfusion reaction;
- non-haemolytic transfusion reaction (mainly chills, fever and urticaria);
- anaphylaxis;
- allo-immunisation against red cell and HLA (very rarely) antigens;
- graft versus host disease (TA-GvHD);
- sepsis due to inadvertent bacterial contamination;
- viral transmission (hepatitis, HIV, etc.) is possible, despite careful donor selection and screening procedures;
- syphilis can be transmitted if components are stored for less than 96 hours at + 4 °C;
- protozoal transmission (e.g. malaria) may occur in rare instances;
- transmission of other pathogens that are not tested for or recognised;
- metabolic imbalance in massive transfusion (e.g. hyperkalaemia);
- transfusion-associated circulatory overload;
- iron overload.

9. Red Cells, Cryopreserved

Definition and properties

Red Cells, Cryopreserved (Cryo) is a red cell component derived by secondary processing of a red cell component or *Whole Blood*. Red cells are frozen (preferably within 7 days of collection) using a cryoprotectant and stored at – 60 °C to – 80 °C or below, depending on the method of cryopreservation.

A reconstituted unit of *Red Cells, Cryo* contains low amounts of protein, leucocytes and platelets. Each unit of *Red Cells, Cryo* contains a minimum haemoglobin content of 36 g. The haematocrit is 0.35-0.70.

Preparation

Two methods are generally used for the preparation of *Red Cells, Cryo*. One is a high glycerol, the other a low glycerol technique. Both methods require a washing/de-glycerolisation procedure.

Requirements and quality control

Table 5B-9 lists the requirements. Additional testing may be required to comply with national requirements (see also Chapter 9, Standards for screening for infectious markers).

Table 5B-9

Parameter to be checked	Requirements	Frequency of control
ABO, RhD	Grouping	All units
Anti-HIV 1 & 2	Negative by approved screening test	All units
HBsAg	Negative by approved screening test	All units
Anti-HCV	Negative by approved screening test	All units
Volume [a]	> 185 mL	as determined by SPC
Haemoglobin (supernatant) [b]	< 0.2 g per unit	as determined by SPC
Haematocrit [a]	0.35-0.70	as determined by SPC
Haemoglobin [a]	Minimum 36 g per unit	as determined by SPC

a If different from 'All units', the frequency of control is an indication of minimal frequency but, where SPC is used to minimise the risk of process deviation, this frequency may be adjusted accordingly.
b Final suspending solution.
c These requirements are deemed to have been met if 90 % of the tested units fall within the values indicated.

Parameter to be checked	Requirements	Frequency of control
Osmolarity [c]	Maximum 20 mOsm/L above osmolarity of resuspending fluid	
Residual leucocyte content [a]	$< 1 \times 10^6$ per unit	as determined by SPC
Sterility [a]	Sterile	as determined by SPC

a If different from 'All units', the frequency of control is an indication of minimal frequency but, where SPC is used to minimise the risk of process deviation, this frequency may be adjusted accordingly.
b Final suspending solution.
c These requirements are deemed to have been met if 90 % of the tested units fall within the values indicated.

Since cryopreservation allows prolonged storage, serum and/or plasma samples obtained at collection must also be stored to enable future testing for newly discovered markers of transmissible diseases when components are thawed for use.

Storage and transport

Red Cells, Cryo in frozen state

Red Cells, Cryo in the frozen state must be constantly maintained between:

- – 60 °C to – 80 °C if stored in an electric freezer and when a high glycerol method is used;
- – 140 °C to – 150 °C if stored in vapour-phase liquid nitrogen and when a low glycerol method is used.

Red Cells, Cryo in the frozen state can be stored for 30 years.

Thawed reconstituted Red Cells, Cryo

Thawed and reconstituted *Red Cells, Cryo* must be stored between + 2 and + 6 °C. The storage time must be validated but should be as short as possible after washing, and must never exceed 24 hours when an open system is used.

If transport in the frozen state is unavoidable, storage conditions must be maintained. Transport of thawed, reconstituted red cells is limited

by the short storage time. Storage conditions must be maintained during transport.

Labelling

The labelling must comply with the relevant national legislation and international agreements.

The following information must be traceable for each frozen unit:

- the producer's identification;
- the unique identity number;
- the date of donation;
- the date of expiry;
- the name and volume of the cryoprotective solution;
- additional component information (if appropriate);
- the volume or weight of the blood component;
- the storage temperature.

Labelling of reconstituted components

After thawing and reconstitution (washing), the date of expiry must be changed to the date (and time) of expiry. Also, the name and volume of the cryoprotective solution must be changed to the name and volume of the additive solution (if any). The following information must be shown on the label of the reconstituted component or must be contained in the component information leaflet, as appropriate:

- the producer's identification;
- the unique identity number;
- the name of the blood component;
- the ABO and RhD groups;
- blood group phenotypes other than ABO and RhD (optional);
- the date of donation;
- the date of expiry;

- the name of the anticoagulant solution;
- the name and volume of the additive solution;
- additional component information (if appropriate);
- the volume or weight of the blood component;
- the storage temperature;
- that the component must not be used for transfusion if there is abnormal haemolysis or other deterioration;
- that the component must be administered through an approved blood administration set.

Warnings

Compatibility of *Red Cells, Cryo* with the intended recipient must be verified by suitable pre-transfusion testing.

RhD-negative female recipients of child-bearing age or younger should not be transfused with red cells from RhD-positive donors.

When *Red Cells, Cryo* are processed in an open system, the risk of bacterial contamination is increased and therefore extra vigilance is required during transfusion.

Adverse reactions include:

- transfusion-associated circulatory overload;
- haemolytic transfusion reaction;
- anaphylaxis;
- allo-immunisation against red cell and HLA (very rarely) antigens;
- sepsis due to inadvertent bacterial contamination;
- viral transmission (hepatitis, HIV, etc.) is possible, despite careful donor selection and screening procedures;
- protozoal transmission (e.g. malaria) may occur in rare instances;

- transmission of other pathogens that are not tested for or recognised;
- iron overload.

Component monographs
Part C. Platelet components

1. Platelets, Recovered, Single Unit

Definition and properties

Platelets, Recovered, Single Unit (Rec, SU) is a platelet component derived from a single *Whole Blood* donation. It contains the majority of the original *Whole Blood* platelet content, suspended in plasma.

Platelets, Rec, SU contains more than 60×10^9 platelets.

Platelets, Rec, SU contains up to 0.2×10^9 leucocytes if prepared by the platelet-rich plasma method, and up to 0.05×10^9 leucocytes if prepared by the buffy coat method.

Platelets, Rec, SU can be used for neonatal and infant transfusion. In order to achieve a ‘standard adult dose’, 4 to 6 units of *Platelets, Rec, SU* have to be transfused.

Preparation

Preparation from platelet-rich plasma (PRP)

A unit of *Whole Blood*, stored for up to 24 hours in conditions validated to maintain the temperature between + 20 °C and + 24 °C, is centrifuged so that an optimal number of platelets remain in the plasma and the number of leucocytes and red cells are reduced to a defined level. Platelets from PRP are sedimented by hard-spin centrifugation; the supernatant platelet-poor plasma is removed, leaving 50-70 mL of it with the platelets. The platelets are allowed to disaggregate and are then resuspended in the remnant plasma.

Preparation from buffy coat

A *Whole Blood* unit, stored for up to 24 hours in conditions validated to maintain the temperature between + 20 and + 24 °C, is centrifuged so that platelets are primarily sedimented to the buffy coat layer together with the leucocytes. The buffy coat is separated and processed further to obtain a platelet concentrate. Single buffy coats diluted with plasma are centrifuged so that the platelets remain in the supernatant, but red cells and leucocytes are sedimented to the bottom of the bag.

Requirements and quality control

Table 5C-1 lists the requirements. Additional testing may be required to comply with national requirements (see also Chapter 9, Chapter 9, Standards for screening for infectious markers).

Demonstration of the swirling phenomenon, which is based on light scattering by platelets in motion and of normal morphology, must be carried out prior to the issue and transfusion of this component. This is best done as soon as possible before transfusion.

Table 5C-1

Parameter to be checked	Requirements	Frequency of control
ABO, RhD	Grouping	All units
Anti-HIV 1 & 2	Negative by approved screening test	All units
HBsAg	Negative by approved screening test	All units
Anti-HCV	Negative by approved screening test	All units
Volume [a]	> 40 mL per 60 × 10^9 of platelets	as determined by SPC
Platelet content per final unit [a]	> 60 × 10^9	as determined by SPC
Residual leucocytes per final unit [a]<br>a. prepared from buffy-coat<br>b. prepared from PRP	a. < 0.05 × 10^9<br>b. < 0.2 × 10^9	as determined by SPC
pH measured (+ 22 °C) at the end of the recommended shelf-life [b]	> 6.4	as determined by SPC

a A minimum of 90 % of units tested should meet the required value.
b All tested units must comply. Measurement of the pH in a closed system is preferable to prevent CO_2 escape. Measurement may be made at another temperature and then corrected.

Storage and transport

Platelets, Rec, SU must be stored under conditions which guarantee that their viability and haemostatic activities are optimally preserved.

The storage temperature must be between + 20 and + 24 °C, under constant agitation.

The maximum storage time for *Platelets, Rec, SU* is 5 days. Storage may be extended to 7 days, in conjunction with appropriate detection or reduction of bacterial contamination.

During transportation, the temperature of *Platelets, Rec, SU* must be kept as close as possible to the recommended storage temperature and, upon receipt, unless intended for immediate therapeutic use, they must be transferred to storage under the recommended conditions.

Labelling

The labelling must comply with the relevant national legislation and international agreements. The following information must be shown on the label or contained in the component information leaflet, as appropriate:

- the producer's identification;
- the unique identity number; if platelets are pooled the original donations must be traceable;
- the name of the blood component;
- the ABO and RhD groups;
- the date of donation;
- the date of expiry;
- the name of the anticoagulant solution;
- additional component information: irradiated, etc. (if appropriate);
- the volume of the blood component;
- the number of platelets (average or actual, as appropriate);

- the storage temperature;
- that the component must be administered through an approved blood administration set.

Warnings

RhD-negative female recipients of child-bearing age or younger should preferably not be transfused with platelets from RhD-positive donors. If this is unavoidable, administration of anti-D immunoglobulin should be considered.

Platelets, Rec, SU are not recommended in cases of:

- plasma intolerance.

Adverse reactions include:

- haemolytic reaction due to transfusion of ABO-incompatible plasma in the component;
- non-haemolytic transfusion reaction (mainly chills, fever and urticaria);
- anaphylaxis;
- allo-immunisation against HLA and red cell antigens;
- allo-immunisation against HPA antigens;
- transfusion-related acute lung injury (TRALI);
- post-transfusion purpura;
- graft versus host disease (TA-GvHD);
- sepsis due to inadvertent bacterial contamination;
- viral transmission (hepatitis, HIV, etc.) is possible, despite careful donor selection and screening procedures;
- syphilis transmission;
- protozoal transmission (e.g. malaria) may occur in rare instances;

- transmission of other pathogens that are not tested for or recognised;
- citrate toxicity in neonates and in patients with impaired liver function;
- transfusion-associated circulatory overload.

2. Platelets, Recovered, Pooled

Definition and properties

Platelets, Recovered, Pooled (Rec, Pool) is a platelet component derived from 4 to 6 fresh *Whole Blood* donations, which contains the majority of the original platelet content in a therapeutically effective dose, suspended in plasma.

Platelets, Rec, Pool contain a minimum content of 2×10^{11} platelets.

Platelets, Rec, Pool contain a maximum of 1×10^{9} leucocytes.

Preparation

Platelets, Rec, Pool can be produced:

- directly from *Whole Blood*-derived buffy coats, which is the method of choice;
- by secondary processing, after pooling of 4-6 units of *Platelets, Rec, SU.*

Preparation from buffy coat

A *Whole Blood* unit, stored in conditions validated to maintain the temperature between + 20 and + 24 °C, for up to 24 hours, is centrifuged so that the platelets are primarily sedimented to the buffy coat layer, together with the leucocytes. The buffy coat is separated and further processed so that, usually, 4 to 6 blood group-compatible buffy coats are pooled in a sterile manner and re-suspended with plasma. After careful mixing, the buffy coat pool is centrifuged (soft- spin) so that the platelets remain in the supernatant, but the red cells and leucocytes are effectively sedimented to the bottom of the bag. The

platelet-containing supernatant is immediately transferred into a suitable platelet storage bag in a sterile manner.

Preparation from Platelets, Recovered, Single Units (PRP method)

Four to six units of *Platelets, Rec, SU* prepared by the PRP method are connected and pooled. If storage for longer than 6 hours is intended, pooling must be undertaken in a sterile manner.

Requirements and quality control

Table 5C-2 lists the requirements. Additional testing may be required to comply with national requirements (see also Chapter 9, Standards for screening for infectious markers).

Table 5C-2

Parameter to be checked	Requirements	Frequency of control
ABO, RhD	Grouping	All units
Anti-HIV 1 & 2	Negative by approved screening test	All units
HBsAg	Negative by approved screening test	All units
Anti-HCV	Negative by approved screening test	All units
Volume [a]	> 40 mL per 60×10^9 of platelets	as determined by SPC
Platelet content per final unit [a]	$> 2 \times 10^{11}$	as determined by SPC
Residual leucocyte content [b]	$< 1 \times 10^9$ per final unit	as determined by SPC
pH measured (+22°C) at the end of the recommended shelf-life [c]	> 6.4	as determined by SPC

a A minimum of 90 % of units tested should meet the required value.
b These requirements are deemed to have been met if 90 % of the tested units fall within the values indicated.
c All tested units must comply. Measurement of the pH in a closed system is preferable to prevent CO_2 escape. Measurement may be made at another temperature and then corrected.

Demonstration of the swirling phenomenon, which is based on light scattering by platelets in motion and of normal morphology, may be carried out either as a separate quality-control procedure or as a routine part of the issue and transfusion of this component. This is best done just before transfusion.

Storage and transport

Platelets, Rec, Pool must be stored under conditions which guarantee that their viability and haemostatic activities are optimally preserved.

The storage temperature must be between + 20 and + 24 °C, under constant agitation.

The maximum storage time for *Platelets, Rec, Pool* is 5 days. Storage may be extended to 7 days, in conjunction with appropriate detection or reduction of bacterial contamination.

When an open system has been used for the preparation of *Platelets, Rec, Pool*, the storage time must not exceed 6 hours.

During transportation, the temperature of *Platelets, Rec, Pool* must be kept as close as possible to the recommended storage temperature and, upon receipt, unless intended for immediate therapeutic use, the component must be transferred to storage under the recommended conditions.

Labelling

The labelling must comply with the relevant national legislation and international agreements. The following information must be shown on the label or contained in the component information leaflet, as appropriate:

- the producer's identification;
- the unique identity number; if platelets are pooled the original donations must be traceable;
- the name of the blood component;
- the ABO and RhD groups;

- the date of donation;
- the date of expiry;
- the name of the anticoagulant solution;
- additional component information: irradiated, number of donations combined to make the pool, etc. (if appropriate);
- the volume of the blood component;
- the number of platelets (average or actual, as appropriate);
- the storage temperature;
- that the component must be administered through an approved blood administration set.

Warnings

RhD-negative female recipients of child-bearing age or younger should preferably not be transfused with platelets from RhD-positive donors. If unavoidable, administration of anti-D immunoglobulin should be considered.

Platelets, Rec, Pool are not recommended in the case of:

- plasma intolerance.

Adverse reactions include:

- transfusion-associated circulatory overload;
- haemolytic reaction due to transfusion of ABO-incompatible plasma in the component;
- anaphylaxis;
- non-haemolytic transfusion reaction (mainly chills, fever and urticaria);
- allo-immunisation against HLA and red cell antigens;
- allo-immunisation against HPA antigens;
- transfusion-related acute lung injury (TRALI);
- post-transfusion purpura;

- graft versus host disease (TA-GvHD);
- sepsis due to inadvertent bacterial contamination;
- viral transmission (hepatitis, HIV, etc.) is possible, despite careful donor selection and screening procedures;
- syphilis transmission;
- protozoal transmission (e.g. malaria) may occur in rare instances;
- transmission of other pathogens that are not tested for or recognised;
- citrate toxicity in neonates and in patients with impaired liver function.

3. Platelets, Recovered, Pooled, Leucocyte-Depleted

Definition and properties

Platelets, Recovered, Pooled, Leucocyte-Depleted (Rec, Pool, LD) is a leucocyte-depleted platelet component derived from 4 to 6 donations of fresh *Whole Blood* and which contains most of the original platelet content in a therapeutically effective dose suspended in plasma.

Platelets, Rec, Pool, LD contains a minimum content of 2×10^{11} platelets.

Platelets, Rec, Pool, LD contains a maximum leucocyte content of 1.0×10^{6} cells.

Preparation

Platelets, Rec, Pool, LD is leucocyte-depleted by filtration. Pre-storage leucocyte filtration is recommended in preference to filtration during or shortly before transfusion.

Platelets, Rec, Pool, LD can be produced:

- directly from *Whole Blood*-derived buffy coats, which is the method of choice;

- by secondary processing, after pooling 4-6 units of *Platelets, Rec, SU*.

Preparation from buffy coat

A *Whole Blood* unit, stored in conditions validated to maintain a temperature between + 20 and + 24 °C for up to 24 hours, is centrifuged so that the platelets are primarily sedimented to the buffy coat layer, together with the leucocytes. The buffy coat is separated and further processed so that, usually, 4 to 6 blood group-compatible buffy coats are pooled in a sterile manner and re-suspended with plasma. After careful mixing, the buffy coat pool is centrifuged (soft-spin) so that the platelets remain in the supernatant, but the red cells and leucocytes are sedimented to the bottom of the bag. The platelet-containing supernatant is immediately filtered and transferred into a suitable platelet storage bag in a sterile manner.

Preparation from Platelets, Recovered, Single Units (PRP method)

Four to six units of *Platelets, Rec, SU*, prepared by the PRP method, are connected, pooled, immediately filtered and transferred into a suitable platelet storage bag. If storage for longer than 6 hours is intended, preparation must be undertaken in a sterile manner.

Requirements and quality control

Table 5C-3 lists the requirements for the final component. Additional testing may be required to comply with national requirements (see also Chapter 9, Standards for screening for infectious markers).

Demonstration of the swirling phenomenon, which is based on light scattering by platelets in motion and of normal morphology, may be carried out either as a separate quality-control procedure or as a routine part of the issue and transfusion of this component. This is best done just before transfusion.

Table 5C-3

Parameter to be checked	Requirements	Frequency of control
ABO, RhD	Grouping	All units
Anti-HIV 1 & 2	Negative by approved screening test	All units
HBsAg	Negative by approved screening test	All units
Anti-HCV	Negative by approved screening test	All units
Volume [a]	> 40 mL per 60 × 10^9 of platelets	as determined by SPC
Platelet content per final unit [a]	> 2 × 10^{11}	as determined by SPC
Residual leucocytes per final unit [a]	< 1 × 10^6	as determined by SPC
pH measured (+ 22 °C) at the end of the recommended shelf-life [b]	> 6.4	as determined by SPC

a A minimum of 90 % of units tested should meet the required value.
b All tested units must comply. Measurement of the pH in a closed system is preferable to prevent CO_2 escape. Measurement may be made at another temperature and then corrected.

Storage and transport

Platelets, Rec, Pool, LD must be stored under conditions which guarantee that its viability and haemostatic activities are optimally preserved.

The storage temperature must be + 20 and + 24 °C, under constant agitation.

The maximum storage time for *Platelets, Rec, Pool, LD* is 5 days. Storage may be extended to 7 days, in conjunction with detection or reduction of bacterial contamination.

When an open system has been used for the preparation of *Platelets, Rec, Pool, LD*, the storage time must not exceed 6 hours.

During transportation, the temperature of *Platelets, Rec, Pool, LD* must be kept as close as possible to the recommended storage temperature and, on receipt, unless intended for immediate therapeutic use, the component must be transferred to storage under the recommended conditions.

Labelling

The labelling must comply with the relevant national legislation and international agreements. The following information must be shown on the label or contained in the component information leaflet, as appropriate:

- the producer's identification;
- the unique identity number. If platelets are pooled, the original donations must be traceable;
- the name of the blood component;
- the ABO and RhD groups;
- the date of donation;
- the date of expiry;
- the name of the anticoagulant solution;
- additional component information: irradiated, number of donations combined to make the pool, etc. (if appropriate);
- the volume of the blood component;
- the number of platelets (average or actual, as appropriate);
- the storage temperature;
- that the component must be administered through an approved blood administration set.

Warnings

RhD-negative female recipients of child-bearing age or younger should preferably not be transfused with platelets from RhD-positive

donors. If unavoidable, administration of anti-D immunoglobulin should be considered.

Platelets, Rec, Pool, LD are not recommended in the case of:

- plasma intolerance.

Adverse reactions include:

- transfusion-associated circulatory overload;
- haemolytic reaction due to transfusion of ABO-incompatible plasma in the component;
- anaphylaxis;
- non-haemolytic transfusion reaction (mainly chills, fever and urticaria);
- allo-immunisation against HLA (very rarely after leucocyte-depletion) and red cell antigens;
- allo-immunisation against HPA antigens;
- transfusion-related acute lung injury (TRALI);
- post-transfusion purpura;
- graft versus host disease (TA-GvHD);
- sepsis due to inadvertent bacterial contamination;
- viral transmission (hepatitis, HIV, etc.) is possible, despite careful donor selection and screening procedures;
- syphilis transmission;
- protozoal transmission (e.g. malaria) may occur in rare instances;
- transmission of other pathogens that are not tested for or recognised;
- citrate toxicity in neonates and in patients with impaired liver function.

4. Platelets, Recovered, Pooled, in Additive Solution

Definition and properties

Platelets, Recovered, Pooled, in Additive Solution (Rec, Pool, AS) is a platelet component derived from 4 to 6 donations of fresh *Whole Blood* which contains most of the original platelet content in a therapeutically effective dose suspended in a mixture of plasma (30-40 %) and an additive solution (60-70 %).

Platelets, Rec, Pool, AS contains a minimum content of 2×10^{11} platelets.

Platelets, Rec, Pool, AS contains a maximum of 0.3×10^{9} leucocytes.

Preparation

Platelets, Rec, Pool, AS is prepared from *Whole Blood*-derived buffy coats.

A *Whole Blood* unit, stored in conditions validated to maintain a temperature between + 20 and + 24 °C for up to 24 hours, is centrifuged so that the platelets are primarily sedimented to the buffy coat layer, together with the leucocytes. The buffy coat is separated and further processed so that, usually, 4 to 6 blood group-compatible buffy coats are pooled in a sterile manner and suspended in an additive solution. After careful mixing, the buffy coat pool is centrifuged (soft-spin) so that the platelets remain in the supernatant, but the red cells and leucocytes are effectively sedimented to the bottom of the bag.

The platelet-containing supernatant is immediately transferred into a suitable platelet storage bag in a sterile manner.

Requirements and quality control

Table 5C-4 lists the requirements. Additional testing may be required to comply with national requirements (see also Chapter 9, Standards for screening for infectious markers).

Table 5C-4

Parameter to be checked	Requirements	Frequency of control
ABO, RhD	Grouping	All units
Anti-HIV 1 & 2	Negative by approved screening test	All units
HBsAg	Negative by approved screening test	All units
Anti-HCV	Negative by approved screening test	All units
Volume [a]	> 40 mL per 60×10^9 of platelets	as determined by SPC
Platelet content per final unit [a]	$> 2 \times 10^{11}$	as determined by SPC
Residual leucocyte content [b]	$< 0.3 \times 10^9$ per final unit	as determined by SPC
pH measured (+ 22 °C) at the end of the recommended shelf-life [c]	> 6.4	as determined by SPC

a A minimum of 90 % of units tested should meet the required value.
b These requirements are deemed to have been met if 90 % of the tested units fall within the values indicated.
c Measurement of the pH in a closed system is preferable to prevent CO_2 escape. Measurement may be made at another temperature and then corrected.

Demonstration of the swirling phenomenon, which is based on light scattering by platelets in motion and of normal morphology, may be carried out either as a separate quality control procedure or as a routine part of the issue and transfusion of this component. This is best done just before transfusion.

Storage and transport

Platelets, Rec, Pool, AS must be stored under conditions which guarantee that its viability and haemostatic activities are optimally preserved.

The storage temperature must be + 20 and + 24 °C, under constant agitation.

The maximum storage time for *Platelets, Rec, Pool, AS* is 5 days. Storage may be extended to 7 days, in conjunction with detection or

reduction of bacterial contamination and depending on the type of additive solution. When an open system has been used for preparation of *Platelets, Rec, Pool, AS*, the storage time must not exceed 6 hours.

During transportation, the temperature of *Platelets, Rec, Pool, AS* must be kept as close as possible to the recommended storage temperature and, on receipt and unless intended for immediate therapeutic use, the component must be transferred to storage under the recommended conditions.

Labelling

The labelling must comply with the relevant national legislation and international agreements. The following information must be shown on the label or contained in the component information leaflet, as appropriate:

- the producer's identification;
- the unique identity number. If platelets are pooled, the original donations must be traceable;
- the name of the blood component;
- the ABO and RhD groups;
- the date of donation;
- the date of expiry;
- the name of the anticoagulant solution;
- the name and volume of the additive solution;
- additional component information: irradiated, number of donations combined to make the pool, etc. (if appropriate);
- the volume of the blood component;
- the number of platelets (average or actual, as appropriate);
- the storage temperature;
- that the component must be administered through an approved blood administration set.

Warnings

RhD-negative female recipients of child-bearing age or younger should preferably not be transfused with platelets from RhD-positive donors. If unavoidable, administration of anti-D immunoglobulin should be considered.

Platelets, Rec, Pool, AS is not recommended in the case of:

- plasma intolerance.

Adverse reactions include:

- transfusion-associated circulatory overload;
- haemolytic reaction due to anti-A, -B in case of incompatible transfusions;
- anaphylaxis;
- non-haemolytic transfusion reaction (mainly chills, fever and urticaria);
- allo-immunisation against HLA and red cell antigens;
- allo-immunisation against HPA antigens;
- transfusion-related acute lung injury (TRALI);
- post-transfusion purpura;
- graft versus host disease (TA-GvHD);
- sepsis due to inadvertent bacterial contamination;
- viral transmission (hepatitis, HIV, etc.) is possible, despite careful donor selection and screening procedures;
- syphilis transmission;
- protozoal transmission (e.g. malaria) may occur in rare instances;
- transmission of other pathogens that are not tested for or recognised;
- citrate toxicity in neonates and in patients with impaired liver function.

5. Platelets, Recovered, Pooled, Leucocyte-Depleted, in Additive Solution

Definition and properties

Platelets, Recovered, Pooled, Leucocyte-Depleted, in Additive Solution (Rec, Pool, LD-AS) is a leucocyte-depleted platelet component derived from 4 to 6 fresh *Whole Blood* donations, which contains the majority of the original platelet content in a therapeutically effective dose, suspended in a mixture of plasma (30-40 %) and an additive solution (60-70 %).

Platelets, Rec, Pool, LD-AS contains a minimum content of 2×10^{11} platelets.

Platelets, Rec, Pool, LD-AS contains a maximum of 1.0×10^{6} leucocytes.

Preparation

Platelets, Rec, Pool, LD-AS is prepared from *Whole Blood*-derived buffy coats and is then leucocyte-depleted by filtration. Pre-storage leucocyte filtration within 6 hours of preparation is recommended.

A *Whole Blood* unit, stored in conditions validated to maintain a temperature between + 20 and + 24 °C for up to 24 hours, is centrifuged so that the platelets are primarily sedimented to the buffy coat layer, together with leucocytes. The buffy coat is separated and further processed so that, usually, 4 to 6 blood group-compatible buffy coats are pooled in a sterile manner and suspended in an additive solution. After careful mixing, the buffy coat pool is centrifuged (soft-spin) so that the platelets remain in the supernatant, but the red cells and leucocytes are effectively sedimented to the bottom of the bag. The platelet-containing supernatant is immediately filtered and transferred into a suitable platelet storage bag in a sterile manner.

Requirements and quality control

Table 5C-5 lists the requirements. Additional testing may be required to comply with national requirements (see also Chapter 9, Standards for screening for infectious markers).

Demonstration of the swirling phenomenon, which is based on light scattering by platelets in motion and of normal morphology, may be carried out either as a separate quality-control procedure or as a routine part of the issue and transfusion of this component. This is best done as soon as possible before transfusion.

Table 5C-5

Parameter to be checked	Requirements	Frequency of control
ABO, RhD	Grouping	All units
Anti-HIV 1 & 2	Negative by approved screening test	All units
HBsAg	Negative by approved screening test	All units
Anti-HCV	Negative by approved screening test	All units
Volume [a]	> 40 mL per 60×10^9 of platelets	as determined by SPC
Platelet content per final unit [a]	$> 2 \times 10^{11}$	as determined by SPC
Residual leucocyte content [b]	$< 1 \times 10^6$ per final unit	as determined by SPC
pH measured (+ 22 °C) at the end of the recommended shelf-life [c]	> 6.4	as determined by SPC

a A minimum of 90 % of units tested should meet the required value.
b These requirements are deemed to have been met if 90 % of the tested units fall within the values indicated.
c Measurement of the pH in a closed system is preferable to prevent CO_2 escape. Measurement may be made at another temperature and then corrected.

Storage and transport

Platelets, Rec, Pool, LD-AS must be stored under conditions which guarantee that their viability and haemostatic activities are optimally preserved.

The storage temperature must be + 20 and + 24 °C under constant agitation.

The maximum storage time for *Platelets, Rec, Pool, LD-AS* is 5 days. Storage may be extended to 7 days, in conjunction with detection or reduction of bacterial contamination and depending on the type of additive solution.

During transportation, the temperature of *Platelets, Rec, Pool, LD-AS* must be kept as close as possible to the recommended storage temperature and, on receipt, unless intended for immediate therapeutic use, the component must be transferred to storage under the recommended conditions.

Labelling

The labelling must comply with the relevant national legislation and international agreements. The following information must be shown on the label or contained in the component information leaflet, as appropriate:

- the producer's identification;
- the unique identity number. If platelets are pooled, the original donations must be traceable;
- the name of the blood component;
- the ABO and RhD groups;
- the date of donation;
- the date of expiry;
- the name of the anticoagulant solution;
- the name and volume of the additive solution;

- additional component information: irradiated, number of donations combined to make the pool, etc. (if appropriate);
- the volume of the blood component;
- the number of platelets (average or actual, as appropriate);
- the storage temperature;
- that the component must be administered through an approved blood administration set.

Warnings

RhD-negative female recipients of child-bearing age or younger should preferably not be transfused with platelets from RhD-positive donors. If unavoidable, administration of anti-D immunoglobulin should be considered.

Platelets, Rec, Pool, LD, AS is not recommended in the case of:

- plasma intolerance.

Adverse reactions include:

- transfusion-associated circulatory overload;
- haemolytic reaction due to anti-A, -B in case of incompatible transfusions;
- anaphylaxis;
- non-haemolytic transfusion reactions (mainly chills, fever and urticaria). The incidence is reduced by the use of pre-storage leucocyte-depleted platelets;
- allo-immunisation against HLA (very rarely after leucocyte-depletion) and red cell antigens;
- allo-immunisation against HPA antigens;
- transfusion-related acute lung injury (TRALI);
- post-transfusion purpura;
- graft versus host disease (TA-GvHD);

- sepsis due to inadvertent bacterial contamination;
- viral transmission (hepatitis, HIV, etc.) is possible, despite careful donor selection and screening procedures;
- syphilis transmission;
- protozoal transmission (e.g. malaria) may occur in rare instances;
- transmission of other pathogens that are not tested for or recognised;
- citrate toxicity in neonates and in patients with impaired liver function.

6. Platelets, Pooled, Pathogen-reduced

Definition and properties

Platelets, Pooled, Pathogen Reduced (Pool, PR) is a leucocyte-depleted platelet component derived from 4 to 6 fresh *Whole Blood* donations, which contains the majority of the original platelet content in a therapeutically effective dose suspended in plasma or a mixture of plasma (30-40 %) and an additive solution (60-70 %). Subsequently, the component is subjected to treatment with an approved and validated pathogen reduction technology (PRT) before storage.

Platelets, Pool, PR contains a minimum content of 2×10^{11} platelets.

Platelets, Pool, PR contains a maximum leucocyte content of 1.0×10^{6} cells.

The PRT typically reduces the risk of infection with enveloped viruses (e.g. HBV, HCV, HIV) and with most bacteria (with the exception of bacterial spores) by at least one-thousand-fold.

Depending on the procedure, some PRT have been shown to inactivate lymphocytes and, if this is the case, irradiation to prevent transfusion-associated TA-GvHD is not required.

Preparation

Platelets, Pool, PR is prepared by pooling several *Whole Blood* donations as described for *Platelets, Recovered, Pooled, Leucocyte- Depleted* and *Platelets, Recovered, Pooled, Leucocyte-Depleted, in Additive Solution*.

The PRT is undertaken according to manufacturers' instructions.

Requirements and quality control

The parameters listed in Table 5C-6 must be checked with the given frequency. A technical procedure must be in place to ensure that the illumination of the PRT has been performed correctly. Additional testing might be necessary to comply with national requirements (see Chapter 9, Standards for screening for infectious markers).

Demonstration of the swirling phenomenon, which is based on light scattering by platelets in motion and of normal morphology, may be carried out either as a separate quality-control procedure or as a routine part of the issue and transfusion of this component. This is best done just before transfusion.

Table 5C-6

Parameter to be checked	Requirements	Frequency of control
ABO, RhD	Grouping	All units
Anti-HIV 1 & 2	Negative by approved screening test	All units
HBsAg	Negative by approved screening test	All units
Anti-HCV	Negative by approved screening test	All units

a A minimum of 90 % of units tested should meet the required value.
b These requirements are deemed to have been met if 90 % of the tested units fall within the values indicated.
c Measurement of the pH in a closed system is preferable to prevent CO_2 escape. Measurement may be made at another temperature and then corrected.

Parameter to be checked	Requirements	Frequency of control
Volume [a]	> 40 mL per 60×10^9 of platelets	as determined by SPC
Platelet content per final unit [a]	> 2×10^{11}	as determined by SPC
Residual leucocyte content [b]	< 1×10^6 per final unit	as determined by SPC
pH measured (+ 22 °C) at the end of the recommended shelf-life [c]	> 6.4	as determined by SPC

a A minimum of 90 % of units tested should meet the required value.
b These requirements are deemed to have been met if 90 % of the tested units fall within the values indicated.
c Measurement of the pH in a closed system is preferable to prevent CO_2 escape. Measurement may be made at another temperature and then corrected.

Storage and transport

Platelets, Pool, PR must be stored under conditions which guarantee that their viability and haemostatic activities are optimally preserved.

Storage temperature must be between + 20 °C to + 24 °C under constant agitation.

The maximum storage time for *Platelets, Pool, PR* may be extended to 7 days depending on the PRT and on the type of additive solution.

During transportation the temperature of *Platelets, Pool, PR* must be kept as close as possible to recommended storage temperature and, on receipt, unless intended for immediate therapeutic use, they should be transferred to storage under recommended conditions.

Labelling

The labelling must comply with the relevant national legislation and international agreements. The following information must be shown on the label or contained in the product information leaflet, as appropriate:

- the producer's identification;
- the unique identity number (the original donations contributing to the pool must be traceable);

- the name of the blood component ;
- the name of the PRT used;
- the ABO and RhD group;
- the date of donation;
- the date of expiry;
- the name of the anticoagulant solution;
- the name and volume of the additive solution;
- additional component information: leucocyte-depleted, number of donations combined to make the pool;
- the volume of the blood component;
- the number of platelets (average or actual, as appropriate);
- the temperature of storage;
- that the component must be administered through an approved blood administration set.

Warnings

RhD negative female recipients of child-bearing age or younger should preferably not be transfused with platelets from RhD positive donors. If unavoidable, administration of anti-D immunoglobulin should be considered.

Platelets, Pool, PR is not recommended:

- in a patient with intolerance to plasma proteins;
- when prepared by amotosalen treatment in neonates undergoing phototherapy with devices that emit a peak energy wavelength less than 425 nm, and/or have a lower bound of the emission bandwidth < 375 nm;
- in a patient with known allergy to the compounds used for, or generated by, the PRT.

Adverse effects include:

- transfusion associated circulatory overload;
- haemolytic reactions due to anti-A, -B in case of incompatible transfusions;
- non-haemolytic transfusion reactions may occur (mainly chills, fever and urticaria). The incidence is reduced by the use of pre-storage leucocyte-depleted platelets;
- allo-immunisation against HLA (very rarely after leucocyte-depletion) and red cell antigens;
- allo-immunisation against HPA antigens;
- transfusion related acute lung injury (TRALI);
- post-transfusion purpura;
- viral transmission and bacterial contamination (other than bacterial spores) is highly unlikely. Transmission of other pathogens that are not sensitive to PRT is possible;
- citrate toxicity in patients with impaired liver function;
- anaphylaxis and allergic reactions (including allergy to the compounds used for, or generated by, PRT).

7. Platelets, Apheresis

Definition and properties

Platelets, Apheresis (Aph) is a component obtained by platelet apheresis of a single donor using automated cell separation equipment, which contains platelets in a therapeutically effective dose suspended in plasma.

Platelets, Aph contains a minimum content of 2×10^{11} platelets.

Platelets, Aph contains a maximum leucocyte content of 0.3×10^{9} cells.

Preparation

For preparation of Platelets, *Aph*, *Whole Blood* is removed from the donor by the apheresis machine, anti-coagulated with a citrate solution and then the platelets are harvested.

For use in neonates and infants, *Platelets, Aph* can be divided into satellite units under sterile conditions.

Requirements and quality control

Table 5C-7 lists the requirements. Additional testing may be required to comply with national requirements (see also Chapter 9, Standards for screening for infectious markers).

Table 5C-7

Parameter to be checked	Requirements	Frequency of control
ABO, RhD	Grouping	All units
Anti-HIV 1 & 2	Negative by approved screening test	All units
HBsAg	Negative by approved screening test	All units
Anti-HCV	Negative by approved screening test	All units
Volume [a]	> 40 mL per 60×10^9 of platelets	as determined by SPC
Platelet content [a]	Standard unit: minimum 2×10^{11} per unit<br>For use in neonates or infants: minimum 0.5×10^{11} per unit	as determined by SPC
Residual leucocyte content [b]	$< 0.3 \times 10^9$ per unit	as determined by SPC
pH measured (+ 22 °C) at the end of the recommended shelf-life [c]	> 6.4	as determined by SPC

a A minimum of 90 % of units tested should meet the required value.
b These requirements are deemed to have been met if 90 % of the tested units fall within the values indicated.
c Measurement of the pH in a closed system is preferable to prevent CO_2 escape. Measurement may be made at another temperature and then corrected.

Demonstration of the swirling phenomenon, which is based on light scattering by platelets in motion and of normal morphology, may be carried out either as a separate quality-control procedure or as a

routine part of the issuance and transfusion of this component. This is best done just before transfusion.

Storage and transport

Platelets, Aph must be stored under conditions which guarantee that their viability and haemostatic activities are optimally preserved.

The storage temperature must be between + 20 to + 24 °C, under constant agitation.

Platelets, Aph to be stored for more than 6 hours must be collected and prepared in a functionally closed system. The maximum storage time for *Platelets, Aph* is 5 days. Storage may be extended to 7 days, in conjunction with detection or reduction of bacterial contamination.

During transportation, the temperature of *Platelets, Aph* must be kept as close as possible to the recommended storage temperature and, on receipt, unless intended for immediate therapeutic use, the component must be transferred to storage under the recommended conditions.

Labelling

The labelling must comply with the relevant national legislation and international agreements. The following information must be shown on the label or contained in the component information leaflet, as appropriate:

- the producer's identification;
- the unique identity number. If two or more units are collected from the donor in one session, each component must have a unique component identity number;
- the name of the blood component;
- the ABO and RhD groups;
- the date of donation;
- the date of expiry;
- the name of the anticoagulant solution;

- additional component information: irradiated, etc. (if appropriate);
- the volume of the blood component;
- the number of platelets (average or actual, as appropriate);
- the storage temperature;
- the relevant HLA and/or HPA type, if determined;
- that the component must be administered through an approved blood administration set.

Warnings

RhD-negative female recipients of child- bearing age or younger should preferably not be transfused with platelets from RhD-positive donors. If unavoidable, administration of anti-D immunoglobulin should be considered.

Platelets, Aph is not recommended in the case of:

- plasma intolerance.

Adverse reactions include:

- transfusion-associated circulatory overload;
- haemolytic reaction due to transfusion of ABO-incompatible plasma in the component;
- anaphylaxis;
- non-haemolytic transfusion reaction (mainly chills, fever and urticaria);
- allo-immunisation against HLA and red cell antigens;
- allo-immunisation against HPA antigens;
- transfusion-related acute lung injury (TRALI);
- post-transfusion purpura;
- graft versus host disease (TA-GvHD);
- sepsis due to inadvertent bacterial contamination;

- viral transmission (hepatitis, HIV, etc.) is possible, despite careful donor selection and screening procedures;
- syphilis transmission;
- protozoal transmission (e.g. malaria) may occur in rare instances;
- transmission of other pathogens that are not tested for or recognised;
- citrate toxicity in neonates and in patients with impaired liver function.

8. Platelets, Apheresis, Leucocyte-Depleted

Definition and properties

Platelets, Apheresis, Leucocyte-Depleted (Aph, LD) is a leucocyte-depleted platelet component obtained by platelet apheresis of a single donor using automated cell separation equipment, which contains platelets in a therapeutically effective dose suspended in plasma.

Platelets, Aph, LD contains a minimum content of 2×10^{11} platelets.

Platelets, Aph, LD normally contains a maximum content of 1.0×10^{6} leucocytes.

Preparation

To prepare *Platelets, Aph, LD*, *Whole Blood* is removed from the donor by the apheresis machine, anti-coagulated with a citrate solution and the platelets are then harvested. Centrifugation, filtration or other in-process steps are included in the process to reduce the number of contaminating leucocytes. Pre-storage leucocyte depletion is recommended (within 6 hours after preparation if performed by filtration).

For use in neonates and infants, *Platelets, Aph, LD* can be divided into satellite units under sterile conditions.

Requirements and quality control

Table 5C-8 lists the requirements. Additional testing may be required to comply with national requirements (see also Chapter 9, Standards for screening for infectious markers).

Demonstration of the swirling phenomenon, which is based on light scattering by platelets in motion and of normal morphology, may be carried out either as a separate quality control procedure or as a routine part of the issue and transfusion of this component. This is best done just before transfusion.

Table 5C-8

Parameter to be checked	Requirements	Frequency of control
ABO, RhD	Grouping	All units
Anti-HIV 1 & 2	Negative by approved screening test	All units
HBsAg	Negative by approved screening test	All units
Anti-HCV	Negative by approved screening test	All units
Volume [a]	> 40 mL per 60×10^9 of platelets	as determined by SPC
Platelet content [a]	Standard unit: minimum 2×10^{11} per unit<br>For use in neonates or infants: minimum 0.5×10^{11} per unit	as determined by SPC
Residual leucocyte content [b]	$< 1 \times 10^6$ per unit	as determined by SPC
pH measured (+ 22 °C) at the end of the recommended shelf-life [c]	> 6.4	as determined by SPC

a A minimum of 90 % of units tested should meet the required value.
b These requirements are deemed to have been met if 90 % of the tested units fall within the values indicated.
c Measurement of the pH in a closed system is preferable to prevent CO_2 escape. Measurement may be made at another temperature and then corrected.

Storage and transport

Platelets, Aph, LD must be stored under conditions which guarantee that their viability and haemostatic activities are optimally preserved.

The storage temperature must be between + 20 to + 24 °C, under constant agitation.

Platelets, Aph, LD to be stored for more than 6 hours must be collected and prepared in a functionally closed system. The maximum storage time for *Platelets, Aph, LD* is 5 days. Storage may be extended to 7 days, in conjunction with detection or reduction of bacterial contamination.

During transportation, the temperature of *Platelets, Aph, LD* must be kept as close as possible to the recommended storage temperature and, upon receipt, unless intended for immediate therapeutic use, the component must be transferred to storage under the recommended conditions.

Labelling

The labelling must comply with the relevant national legislation and international agreements. The following information must be shown on the label or contained in the component information leaflet, as appropriate:

- the producer's identification;
- the unique identity number. If two or more units are collected from the donor in one session, each component must have a unique component identity number;
- the name of the blood component;
- the ABO and RhD groups;
- the date of donation;
- the date of expiry;
- the name of the anticoagulant solution;

- additional component information: irradiated, etc. (if appropriate);
- the volume of the blood component;
- the number of platelets (average or actual, as appropriate);
- the storage temperature;
- the relevant HLA and/or HPA type, if determined;
- that the component must be administered through an approved blood administration set.

Warnings

RhD-negative female recipients of child-bearing age or younger should preferably not be transfused with platelets from RhD-positive donors. If unavoidable, administration of anti-D immunoglobulin should be considered.

Platelets, Apheresis, LD is not recommended in the case of:

- plasma intolerance.

Adverse reactions include:

- transfusion-associated circulatory overload;
- haemolytic reaction due to transfusion of ABO-incompatible plasma in the component;
- anaphylaxis;
- non-haemolytic transfusion reaction (mainly chills, fever and urticaria). The incidence is reduced by the use of pre-storage leucocyte-depleted platelets;
- allo-immunisation against HLA (very rarely) and red cell antigens;
- allo-immunisation against HPA antigens;
- transfusion-related acute lung injury (TRALI);
- post-transfusion purpura;

- graft versus host disease (TA-GvHD);
- sepsis due to inadvertent bacterial contamination;
- viral transmission (hepatitis, HIV, etc.) is possible, despite careful donor selection and screening procedures;
- syphilis transmission;
- protozoal transmission (e.g. malaria) may occur in rare instances;
- transmission of other pathogens that are not tested for or recognised;
- citrate toxicity in neonates and in patients with impaired liver function.

9. Platelets, Apheresis, in Additive Solution

Definition and properties

Platelets, Apheresis, in Additive Solution (Aph, AS) is a component obtained by platelet apheresis of a single donor using automated cell separation equipment, which contains platelets in a therapeutically effective dose suspended in a mixture of plasma (30-40 %) and an additive solution (60-70 %).

Platelets, Aph, AS contains a minimum content of 2×10^{11} platelets.

Platelets, Aph, AS contains a maximum leucocyte content of 0.3×10^{9} cells.

Preparation

To prepare *Platelets, Aph, AS*, *Whole Blood* is removed from the donor by the apheresis machine, anti-coagulated with a citrate solution and then the platelets are harvested. Platelets are stored in a combination of plasma and an appropriate additive solution.

For use in neonates and infants, *Platelets, Aph, AS* can be divided into satellite units under sterile conditions.

Requirements and quality control

Table 5C-9 lists the requirements. Additional testing may be required to comply with national requirements (see also Chapter 9, Standards for screening for infectious markers).

Table 5C-9

Parameter to be checked	Requirements	Frequency of control
ABO, RhD	Grouping	All units
Anti-HIV 1 & 2	Negative by approved screening test	All units
HBsAg	Negative by approved screening test	All units
Anti-HCV	Negative by approved screening test	All units
Volume [a]	> 40 mL per 60×10^9 of platelets	as determined by SPC
Platelet content [a]	Standard unit: minimum 2×10^{11} per unit<br>For use in neonates or infants: minimum 0.5×10^{11} per unit	as determined by SPC
Residual leucocyte content [b]	$< 0.3 \times 10^9$ per final unit	as determined by SPC
pH measured (+ 22 °C) at the end of the recommended shelf-life [c]	> 6.4	as determined by SPC

a A minimum of 90 % of units tested should meet the required value.
b These requirements are deemed to have been met if 90 % of the tested units fall within the values indicated.
c Measurement of the pH in a closed system is preferable to prevent CO_2 escape. Measurement may be made at another temperature and then corrected.

Demonstration of the swirling phenomenon, which is based on light scattering by platelets in motion and of normal morphology, may be carried out either as a separate quality-control procedure or as a routine part of the issue and transfusion of this component. This is best done just before transfusion.

Storage and transport

Platelets, Aph, AS must be stored under conditions which guarantee that their viability and haemostatic activities are optimally preserved.

The storage temperature must be + 20 to + 24 °C, under constant agitation.

Platelets, Aph, AS to be stored for more than 6 hours must be collected and prepared in a functionally closed system. The maximum storage time for *Platelets, Aph, AS* is 5 days. Storage may be extended to 7 days, in conjunction with detection or reduction of bacterial contamination and depending on the type of additive solution.

During transportation, the temperature of *Platelets, Aph, AS* must be kept as close as possible to the recommended storage temperature and, on receipt, unless intended for immediate therapeutic use, the component must be transferred to storage under the recommended conditions.

Labelling

The labelling must comply with the relevant national legislation and international agreements. The following information must be shown on the label or contained in the component information leaflet, as appropriate:

- the producer's identification;
- the unique identity number. If two or more units are collected from the donor in one session, each component must have a unique component identity number;
- the name of the blood component;
- the ABO and RhD groups;
- the date of donation;
- the date of expiry;
- the name of the anticoagulant solution;
- the name and volume of the additive solution;

- additional component information: irradiated, etc. (if appropriate);
- the volume of the blood component;
- the number of platelets (average or actual, as appropriate);
- the storage temperature;
- the relevant HLA and/or HPA type, if determined;
- that the component must be administered through an approved blood administration set.

Warnings

RhD-negative female recipients of child-bearing age or younger should preferably not be transfused with platelets from RhD-positive donors. If unavoidable, administration of anti-D immunoglobulin should be considered.

Platelets, Apheresis, AS is not recommended in the case of:

- plasma intolerance.

Adverse reactions include:

- transfusion-associated circulatory overload;
- haemolytic reaction due to anti-A, -B in case of incompatible transfusions;
- anaphylaxis;
- non-haemolytic transfusion reaction (mainly chills, fever and urticaria);
- allo-immunisation against HLA and red cell antigens;
- allo-immunisation against HPA antigens;
- transfusion-related acute lung injury (TRALI);
- post-transfusion purpura;
- graft versus host disease (TA-GvHD);
- sepsis due to inadvertent bacterial contamination;

- viral transmission (hepatitis, HIV, etc.) is possible, despite careful donor selection and screening procedures;
- syphilis transmission;
- protozoal transmission (e.g. malaria) may occur in rare instances;
- transmission of other pathogens that are not tested for or recognised;
- citrate toxicity in neonates and in patients with impaired liver function.

10. Platelets, Apheresis, Leucocyte-Depleted, in Additive Solution

Definition and properties

Platelets, Apheresis, Leucocyte-Depleted, in Additive Solution (Aph, LD-AS) is a leucocyte-depleted platelet component obtained by platelet apheresis of a single donor using automated cell-separation equipment, which contains platelets in a therapeutically effective dose suspended in a mixture of plasma (30-40 %) and an additive solution (60-70 %).

Platelets, Aph, LD-AS contains a minimum content of 2×10^{11} platelets.

Platelets, Aph, LD-AS contains a maximum of 1.0×10^{6} leucocytes.

Preparation

To prepare *Platelets, Aph, LD-AS*, *Whole Blood* is removed from the donor by the apheresis machine, anti-coagulated with a citrate solution and then the platelets are harvested. Platelets are stored in a combination of plasma and an appropriate nutrient solution. Centrifugation, filtration or other in-process steps are included in the process to reduce the number of contaminating leucocytes. Pre-storage leucocyte depletion is recommended (within 6 hours after preparation if performed by filtration).

For use in neonates and infants, *Platelets, Aph, LD-AS* can be divided into satellite units under sterile conditions.

Requirements and quality control

Table 5C-10 lists the requirements. Additional testing may be required to comply with national requirements (see also Chapter 9, Standards for screening for infectious markers).

Table 5C-10

Parameter to be checked	Requirements	Frequency of control
ABO, RhD	Grouping	All units
Anti-HIV 1 & 2	Negative by approved screening test	All units
HBsAg	Negative by approved screening test	All units
Anti-HCV	Negative by approved screening test	All units
HLA and/or HPA	As required	All units
Volume [a]	> 40 mL per 60×10^9 of platelets	as determined by SPC
Platelet content [a]	Standard unit: minimum 2×10^{11} per unit<br>For use in neonates or infants: minimum 0.5×10^{11} per unit	as determined by SPC
Residual leucocyte content [b]	$< 1 \times 10^6$ per unit	as determined by SPC
pH measured (+ 22 °C) at the end of the recommended shelf-life [c]	> 6.4	as determined by SPC

a A minimum of 90 % of units tested should meet the required value.
b These requirements are deemed to have been met if 90 % of the tested units fall within the values indicated.
c Measurement of the pH in a closed system is preferable to prevent CO_2 escape. Measurement may be made at another temperature and then corrected.

Demonstration of the swirling phenomenon, which is based on light scattering by platelets in motion and of normal morphology, may be carried out either as a separate quality-control procedure or as a

routine part of the issue and transfusion of this component. This is best done just before transfusion.

Storage and transport

Platelets, Aph, LD-AS must be stored under conditions which guarantee that their viability and haemostatic activities are optimally preserved.

The storage temperature must be between + 20 to + 24 °C, under constant agitation.

Platelets, Aph, LD-AS to be stored for more than 6 hours must be collected and prepared in a functionally closed system. The maximum storage time for *Platelets, Aph, LD-AS* is 5 days. Storage may be extended to 7 days, in conjunction with detection or reduction of bacterial contamination and depending on the type of additive solution.

During transportation, the temperature of *Platelets, Aph, LD-AS* must be kept as close as possible to the recommended storage temperature and, on receipt, unless intended for immediate therapeutic use, the component must be transferred to storage under the recommended conditions.

Labelling

The labelling must comply with the relevant national legislation and international agreements. The following information must be shown on the label or contained in the component information leaflet, as appropriate:

- the producer's identification;
- the unique identity number. If two or more units are collected from the donor in one session, each component must have a unique component identity number;
- the name of the blood component;
- the ABO and RhD groups;
- the date of donation;

- the date of expiry;
- the name of the anticoagulant solution;
- the name and volume of the additive solution;
- additional component information: irradiated, etc. (if appropriate);
- the volume of the blood component;
- the number of platelets (average or actual, as appropriate);
- the storage temperature;
- the relevant HLA and/or HPA type, if determined;
- that the component must be administered through an approved blood administration set.

Warnings

RhD-negative female recipients of child-bearing age or younger should preferably not be transfused with platelets from RhD-positive donors. If unavoidable, administration of anti-D immunoglobulin should be considered.

Platelets, Aph, LD-AS is not recommended in the case of:

- plasma intolerance.

Adverse reactions include:

- transfusion-associated circulatory overload;
- haemolytic reaction due to anti-A, -B in case of incompatible transfusions;
- anaphylaxis;
- non-haemolytic transfusion reaction (mainly chills, fever and urticaria);
- allo-immunisation against HLA (very rarely after pre-storage leucocyte-depletion) and red cell antigens;
- allo-immunisation against HPA antigens;

- transfusion-related acute lung injury (TRALI);
- post-transfusion purpura;
- graft versus host disease (TA-GvHD);
- sepsis due to inadvertent bacterial contamination;
- viral transmission (hepatitis, HIV, etc.) is possible despite careful donor selection and screening procedures;
- syphilis transmission;
- protozoal transmission (e.g. malaria) may occur in rare instances;
- transmission of other pathogens that are not tested for or recognised;
- citrate toxicity in neonates and in patients with impaired liver function.

11. Platelets, Apheresis, Pathogen-reduced

Definition and properties

Platelets, Apheresis, Pathogen Reduced (Aph, PR) is a platelet component obtained by platelet apheresis of a single donor using automated cell separation equipment, which contains platelets in a therapeutically effective dose suspended in plasma or a mixture of plasma (30-50 %) and an additive solution (50-70 %). Subsequently, the component is subjected to treatment with an approved and validated PRT before storage. Pools of two to three platelet components obtained from the same platelet apheresis donation can be made prior to PRT if validated.

Platelets, Aph, PR contains a minimum content of 2×10^{11} platelets.

Platelets, Aph, PR contains a maximum leucocyte content of 1.0×10^{6} cells.

The PRT typically reduces the risk of infection by enveloped viruses (e.g. HBV, HCV, HIV) and most bacteria (with the exception of bacterial spores) by at least one-thousand-fold.

Depending on the procedure, some PRT have been shown to inactivate lymphocytes and, if so, irradiation to prevent transfusion-associated TA-GvHD is not required.

Preparation

To prepare *Platelets, Aph, PR*, *Whole Blood* is removed from the donor by the apheresis machine, anti-coagulated with a citrate solution and then the platelets are harvested. Platelets are stored in plasma or a mixture of plasma (30-50 %) and an additive solution (50-70 %). Centrifugation, filtration or other in-process steps are included in the process to reduce the number of contaminating leucocytes.

The PRT is undertaken according to manufacturer instructions.

Requirements and quality control

The parameters listed in Table 5C-11 must be checked according to the reported frequency. A technical procedure must be in place to ensure that the illumination of the PRT has been performed correctly. Additional testing might be necessary to comply with national requirements (see Chapter 9, Standards for screening for infectious markers).

Demonstration of the swirling phenomenon, which is based on light scattering by platelets in motion and of normal morphology, may be carried out either as a separate quality control procedure or as a routine part of the issue and transfusion of this component. This is best done just before transfusion.

Table 5C-11

Parameter to be checked	Requirements	Frequency of control
ABO, RhD	Grouping	All units
Anti-HIV 1 & 2	Negative by approved screening test	All units
HBsAg	Negative by approved screening test	All units
Anti-HCV	Negative by approved screening test	All units
Volume [a]	> 40 mL per 60 $\times$ 10^9 of platelets	as determined by SPC
Platelet content [a]	minimum 2 $\times$ 10^{11} per unit	as determined by SPC
Residual leucocyte content [b]	< 1 $\times$ 10^6 per unit	as determined by SPC
pH measured (+ 22 °C) at the end of the recommended shelf-life [c]	> 6.4	as determined by SPC

a A minimum of 90 % of units tested should meet the required value.
b These requirements are deemed to have been met if 90 % of the tested units fall within the values indicated.
c Measurement of the pH in a closed system is preferable to prevent CO_2 escape. Measurement may be made at another temperature and then corrected.

Storage and transport

Platelets, Aph, PR must be stored under conditions which guarantee that their viability and haemostatic activities are optimally preserved.

The storage temperature must be between + 20 to + 24 °C, under constant agitation.

The maximum storage time for *Platelets, Aph, PR* may be extended to 7 days depending on the type of additive solution and the PRT.

During transportation, the temperature of *Platelets, Aph, PR* must be kept as close as possible to the recommended storage temperature and, on receipt, unless intended for immediate therapeutic use, they should be transferred to storage under the recommended conditions.

Labelling

The labelling must comply with the relevant national legislation and international agreements. The following information must be shown on the label or contained in the product information leaflet, as appropriate:

- the producer's identification;
- the unique identity number;
- the name of the blood component;
- the name of the PRT used;
- the ABO and RhD groups;
- the date of donation;
- the date of expiry;
- the name of the anticoagulant solution;
- the name and volume of the additive solution;
- additional component information: leucocyte-depleted, number of donations combined to make the pool, etc. (as appropriate);
- the volume of the blood component;
- the number of platelets (average or actual, as appropriate);
- the storage temperature;
- that the component must be administered through an approved blood administration set.

Warnings

RhD-negative female recipients of child-bearing age or younger should preferably not be transfused with platelets from RhD-positive donors. If unavoidable, administration of anti-D immunoglobulin should be considered.

Platelets, Aph, PR is not recommended in cases of:

- plasma intolerance;

- when prepared by amotosalen treatment in neonates undergoing phototherapy with devices that emit a peak energy wavelength less than 425 nm, and/or have a lower bound of the emission bandwidth <375 nm;
- in a patient with known allergy to the compounds used for, or generated by, the PRT.

Adverse reactions include:

- transfusion-associated circulatory overload;
- haemolytic reactions due to anti-A, -B in case of incompatible transfusions;
- non-haemolytic transfusion reactions may occur (mainly chills, fever and urticaria). The incidence is reduced by the use of pre-storage leucocyte-depleted platelets;
- allo-immunisation against HLA (very rarely after leucocyte- depletion) and red cell antigens;
- allo-immunisation against HPA antigens;
- transfusion-related acute lung injury (TRALI);
- post-transfusion purpura;
- viral transmission and bacterial contamination (other than bacterial spores) is highly unlikely. Transmission of other pathogens that are not sensitive to PRT is possible;
- citrate toxicity in patients with impaired liver function;
- anaphylaxis and allergic reactions, including allergy to the compounds used for, or generated by, the PRT.

12. Platelets, Cryopreserved

Definition and properties

Platelets, Cryopreserved (Cryo) is a component prepared by the freezing of *platelet components* within 24 hours of collection, using a cryoprotectant.

Reconstituted *Platelets, Cryo* contain more than 40 % of the original component.

The method facilitates extended storage of platelets from selected donors and of autologous platelets.

Preparation

Platelets, Cryo are prepared by secondary processing of *Platelets, Aph, LD*. The component is cryopreserved within 24 hours of collection using a cryoprotectant. Two methods are, in general, used for preparation of *Platelets, Cryo*: DMSO (6% w/v) and a very low glycerol (5% w/v) technique.

Before use, the platelets are thawed, washed and resuspended in (autologous) plasma or in a suitable additive solution.

Requirements and quality control

As indicated for *Platelets, Aph* (see Table 5C-7), with the following additions and changes:

Table 5C-12

Parameter to be checked	Requirements	Frequency of control
Volume	50-200 mL	All units
Platelet content	> 40 % of the pre-freeze platelet content	All units

Platelets, Cryo when thawed will not swirl.

Storage and transport

Platelets in the frozen state must be constantly maintained at:

- – 80 °C, if stored in an electric freezer;
- – 150 °C, if stored in vapour-phase liquid nitrogen.

If storage will be extended for more than one year, storage at – 150 °C is preferred.

If transport in the frozen state is unavoidable, storage conditions must be maintained during transportation.

Thawed platelets must be used as soon as possible after thawing. If short-to-intermediate storage is required, the component must be kept between + 20 °C to + 24 °C.

Transportation of thawed platelets is limited by the short shelf-life of this component. During transportation, the temperature of *Platelets, Cryo* must be kept as close as possible between + 20 to + 24 °C.

Labelling

The labelling must comply with the relevant national legislation and international agreements.

The following information must be shown on the label or contained in the component information leaflet, as appropriate and must be traceable for each frozen unit:

- the producer's identification;
- the unique identity number;
- the date of donation;
- the date of expiry;
- the name and volume of the cryoprotective solution;
- additional component information if appropriate;
- the volume or weight of the blood component;
- the storage temperature.

Labelling of the reconstituted component

After thawing and reconstitution (washing), the date of expiry must be changed to the date (and time) of expiry, and the name and volume of the cryoprotective solution must be changed to the name and volume of the additive solution (if any).

The following information must be shown on the label or contained in the component information leaflet, as appropriate:

- the producer's identification;
- the unique identity number. If 2 or more units are collected from the donor in one session, each component must have a unique component identity number;
- the ABO and RhD groups;
- the date of preparation;
- the name and volume of the cryoprotective solution;
- the name of the blood component;
- additional component information: Leucocyte depleted, irradiated, etc. (if appropriate);
- the date of expiry (and time of expiry when required);
- the volume or weight of the blood component;
- the storage temperature;
- the HLA and/or HPA type (if determined);
- that the component must be administered through an approved blood administration set.

Warnings

Residual cryoprotectant (e.g. DMSO) can be toxic.

RhD-negative female recipients of child-bearing age or younger should preferably not be transfused with platelets from RhD-positive donors. If unavoidable, administration of anti-D immunoglobulin should be considered.

Adverse reactions include:

- transfusion-associated circulatory overload;
- haemolytic reaction due to anti-A, -B in case of incompatible transfusions when thawed platelets are re-suspended in plasma;
- non-haemolytic transfusion reaction (mainly chills, fever and urticaria);

- allo-immunisation against HLA (very rarely) and red cell antigens;
- allo-immunisation against HPA antigens;
- transfusion-related acute lung injury (TRALI);
- post-transfusion purpura;
- graft versus host disease (TA-GVHD);
- sepsis due to inadvertent bacterial contamination;
- viral transmission (hepatitis, HIV, etc.) is possible, despite careful donor selection and screening procedures;
- syphilis transmission;
- protozoal transmission (e.g. malaria) may occur in rare instances;
- transmission of other pathogens that are not tested for or recognised.

Component monographs
Part D. Plasma components

1. Plasma, Fresh Frozen

Definition and properties

Plasma, Fresh Frozen (FFP) is a component for transfusion or for fractionation, prepared either from *Whole Blood* or from plasma collected by apheresis, frozen within a period of time and to a temperature that adequately maintains the labile coagulation factors in a functional state.

FFP used as Human plasma for fractionation must comply with the specifications of the European Pharmacopoeia monograph *Human plasma for fractionation (0853).*

FFP used for clinical transfusion must comply with the specifications as given in this section (Chapter 5, Part D).

It must contain, on average, 70 % or more of the value of Factor VIII of the freshly collected plasma unit and at least similar quantities of the other labile coagulation factors and naturally occurring inhibitors.

It must not contain irregular antibodies of clinical significance. If leucocyte-depleted, the component must contain less than 1×10^6 leucocytes.

Preparation

From Whole Blood

Plasma is separated from *Whole Blood* that has been collected using a blood bag with integral transfer packs employing hard-spin centrifugation with freezing commenced within 6 hours of collection or within a timeframe validated to result in a component meeting specification. An intermediate step involving preparation of platelet-rich plasma is also permissible.

Alternatively, plasma may be separated from *Whole Blood* that, immediately after donation, has been cooled rapidly by a special device validated to maintain the temperature between + 20 °C and + 24 °C and is held at that temperature for up to 24 hours.

Freezing must take place in a system that allows complete freezing within one hour to a temperature below – 25 °C. If FFP is to be prepared from a single-pack *Whole Blood* donation, adequate sterility precautions must be adopted.

By apheresis

FFP may be collected by apheresis. Freezing must commence either within 6 hours of collection or within a timeframe validated to result in a component meeting specification. Freezing must take place in a system that allows complete freezing within one hour to a temperature below – 25 °C.

Quarantine FFP

Quarantine FFP can be released once the donor has been re-tested, at least for HBsAg, anti-HIV and anti-HCV, with negative results after a defined period of time that is designed to exclude the risk associated with the window period. A period of six months is generally applied. This may be reduced if NAT testing is performed.

Requirements and quality control

Table 5D-1 lists the requirements. Additional testing may be required to comply with national requirements (see also Chapter 9, Standards for screening for infectious markers).

Table 5D-1

Parameter to be checked	Requirements	Frequency of control
ABO, RhD [a, b]	Grouping only for clinical FFP	All units
Anti-HIV 1 & 2 [a]	Negative by approved screening test	All units
HbsAg [a]	Negative by approved screening test	All units

a Unless performed on the source whole blood.
b A minimum of 90 % of units tested should meet the required value.

Parameter to be checked	Requirements	Frequency of control
Anti-HCV [a]	Negative by approved screening test	All units
Volume [b]	Stated volume ± 10 %	as determined by SPC
Factor VIII [b]	Average (after freezing and thawing): not less than 70 IU Factor VIII per 100 mL	as determined by SPC on units in the first month of storage [a]
Residual cells [b]	Red cells: $< 6.0 \times 10^9$/L<br>Leucocytes: $< 0.1 \times 10^9$/L<br>Platelets: $< 50 \times 10^9$/L	as determined by SPC
	If leucocyte-depleted: $< 1 \times 10^6$ per unit	as determined by SPC
Leakage	No leakage in any part of container. Requires visual inspection after pressure in a plasma extractor before freezing	All units
Visual changes	No abnormal colour or visible clots	All units

a Unless performed on the source whole blood.
b A minimum of 90 % of units tested should meet the required value.

Storage and transport

The following storage times and temperatures are permitted:

- 36 months at below – 25 °C;
- 3 months at – 18 °C to – 25 °C.

The storage temperature must be maintained during transport. Unless for immediate use, the packs must be transferred at once to storage at the recommended temperature.

Once thawed, the component must not be refrozen and should be transfused as soon as possible. If delay is unavoidable, the component may be stored and should be used within 4 hours if maintained at 22 ± 2 °C or 24 hours if stored at 4 ± 2 °C. For management of major bleeding, thawed FFP that has been stored at 4 ± 2 °C can be used for

up to 5 days, but it should be borne in mind that extended post-thaw storage will result in a decline in the content of labile coagulation factors.

Labelling

The labelling must comply with the relevant national legislation and international agreements. The following information must be shown on the label or contained in the component information leaflet, as appropriate:

- the producer's identification;
- the unique identity number. If two or more units are collected from the donor in one session, each component must have a unique component identity number;
- the name of the blood component;
- the ABO and RhD groups (only for clinical FFP);
- the date of donation;
- the date of expiry;
- the name of the anticoagulant solution;
- additional component information: leucocyte-depleted, irradiated, quarantined, etc. (if appropriate);
- the volume or weight of the blood component;
- the storage temperature;
- that the component must be administered through an approved blood administration set.

After thawing, the date of expiry must be changed to the appropriate date (and time) of expiry. The storage temperature must also be changed accordingly.

Warnings

Transfusion of ABO blood group-incompatible plasma may result in haemolytic transfusion reaction.

FFP must not be used in a patient with an intolerance to plasma proteins.

Before use, the component must be thawed in a properly controlled environment and the integrity of the pack must be verified to exclude any defects or leakages. No insoluble cryoprecipitate must be visible on completion of the thaw procedure.

Adverse reactions include:

- non-haemolytic transfusion reaction (mainly chills, fever and urticaria);
- transfusion-related acute lung injury (TRALI);
- viral transmission (hepatitis, HIV, etc.) is possible, despite careful donor selection and screening procedures;
- sepsis due to inadvertent bacterial contamination;
- transmission of other pathogens that are not tested for or recognised;
- citrate toxicity in neonates and in patients with impaired liver function;
- transfusion-associated circulatory overload;
- anaphylaxis and allergic reactions.

2. Plasma, Fresh Frozen, Pathogen Reduced

Definition and properties

Plasma, Fresh Frozen, Pathogen Reduced (PR) is a component for transfusion prepared from plasma derived from *Whole Blood* or apheresis plasma which is subjected to treatment with an approved and validated PRT and subsequent freezing within a period of time to a temperature that adequately maintains the labile coagulation factors in a functional state.

Plasma, Fresh Frozen, PR may be prepared from small pools of up to 12 individual donations if in accordance with the specifications of the manufacturer of the PR system used.

Plasma, Fresh Frozen, PR used for clinical transfusion must comply with the specifications given in this monograph.

It contains, on average, about 50 to 70 % of the labile coagulation factors and naturally occurring inhibitors present in fresh unfrozen/thawed plasma.

The PRT typically reduces the risk of infection by enveloped viruses (e.g. HBV, HCV, HIV) by at least one-thousand-fold.

Plasma, Fresh Frozen, PR must not contain irregular antibodies of clinical significance.

If leucocyte-depleted, the component must contain less than 1×10^6 leucocytes.

Preparation

Plasma, Fresh Frozen, PR is prepared from plasma obtained from *Whole Blood* or collected by apheresis as described for *Plasma, Fresh Frozen*. The PRT can be applied either before or after freezing and thawing of the plasma.

The PRT is undertaken according to manufacturers' instructions.

Requirements and quality control

Table 5D-2 lists the requirements. Additional testing may be required to comply with national requirements (see also Chapter 9, Standards for screening for infectious markers).

Table 5D-2

Parameter to be checked	Requirements	Frequency of control
ABO, RhD [a]	Grouping<br>Only for clinical FFP	All units
Anti-HIV 1 & 2 [a]	Negative by approved screening test	All units

a Unless performed on the source whole blood.
b A minimum of 90 % of units tested should meet the required value.

Parameter to be checked	Requirements	Frequency of control
HBsAg [a]	Negative by approved screening test	All units
Anti-HCV [a]	Negative by approved screening test	All units
Volume [b]	Stated volume ± 10 %	All units
Factor VIII [b]	Average: not less than 50 IU Factor VIII per 100 mL	as determined by SPC on units in the first month of storage
Fibrinogen [b]	Average (after freezing and thawing) = 60 % of the potency of the freshly collected plasma unit	as determined by SPC on units in the first month of storage
Residual cells [b]	Red cells: $< 6.0 \times 10^9$/L<br>Leucocytes: $< 0.1 \times 10^9$L<br>Platelets: $< 50 \times 10^9$/L	as determined by SPC
	If leucocyte-depleted: $< 1 \times 10^6$ per unit	as determined by SPC
Leakage	No leakage in any part of container. Requires visual inspection after pressure in a plasma extractor, before freezing and after thawing	All units
Visual changes	No abnormal colour or visible clots	All units

a Unless performed on the source whole blood.
b A minimum of 90 % of units tested should meet the required value.

Storage and transport

The following storage times and temperatures are permitted:

- 36 months at or below – 25 °C;
- 3 months at – 18 °C to – 25 °C.

The storage temperature must be maintained during transport. Unless for immediate use, the packs must be transferred at once to storage at the recommended temperature.

In order to preserve labile factors, *Plasma, Fresh Frozen, PR* must be used as soon as possible following thawing. It must not be re-frozen.

Labelling

The labelling must comply with the relevant national legislation and international agreements. The following information must be shown on the label or contained in the component information leaflet, as appropriate:

- the producer's identification;
- the unique identity number. If two or more units are collected from the donor in one session, each component must have a unique component identity number;
- the name of the blood component;
- the name of the PRT used;
- the ABO and RhD groups;
- the date of donation;
- the date of expiry;
- the name of the anticoagulant solution;
- additional component information: leucocyte-depleted, irradiated, etc. (if appropriate);
- the volume or weight of the blood component;
- the storage temperature;
- that the component must be administered through an approved blood administration set.

After thawing, the date of expiry must be changed to the appropriate date (and time) of expiry. The storage temperature must also be changed accordingly.

Warnings

Transfusion of ABO blood group-incompatible plasma may result in haemolytic transfusion reaction.

Plasma Fresh Frozen, PR must not be used in cases of:

- patients with intolerance to plasma proteins;
- when prepared by amotosalen treatment in neonates undergoing phototherapy with devices that emit a peak energy wavelength less than 425 nm, and/or have a lower bound of the emission bandwidth < 375 nm;
- in a patient with known allergy to the compounds used for, or generated by, the PRT;
- patients with G6PD deficiency when the plasma is prepared by the methylene blue procedure;
- patients with a known allergy to the compounds used for, or generated by, the PRT.

Before use, the component must be thawed in a properly controlled environment and the integrity of the pack must be verified to exclude any defects or leakages. No insoluble cryoprecipitate must be visible on completion of the thaw procedure.

Adverse reactions include:

- non-haemolytic transfusion reaction (mainly chills, fever and urticaria);
- transfusion-related acute lung injury (TRALI);
- viral transmission (hepatitis B and C, HIV) is highly unlikely, but transmission of other pathogens that are not tested for or are not sensitive to PRT is possible;
- citrate toxicity in neonates and in patients with impaired liver function;
- transfusion-associated circulatory overload.

3. Cryoprecipitate

Definition and properties

Cryoprecipitate is a component containing the cryoglobulin fraction of plasma obtained by further processing of *Plasma, Fresh Frozen* and then concentrated.

It contains a major portion of the Factor VIII, von Willebrand factor, fibrinogen, Factor XIII and fibronectin present in freshly drawn and separated plasma.

Preparation

Plasma, Fresh Frozen is thawed, either overnight between + 2 to + 6 °C or by the rapid thaw-siphon thaw technique. After thawing, the component is re-centrifuged using a hard spin at the same temperature. The supernatant cryoprecipitate-poor plasma is then partially removed. The sedimented cryoprecipitate is then rapidly frozen.

When *Cryoprecipitate* is prepared from *Whole Blood*-derived plasma, the maximal final volume of the component is 40 mL.

Alternatively, *Plasma, Fresh Frozen* obtained by apheresis may be used as the starting material and the final component can be prepared using the same freezing/thawing/refreezing technique.

Leucocyte depletion of the starting material and/or virus inactivation, and/or quarantine is a requirement in some countries.

Requirements and quality control

Table 5D-3 lists the requirements. Additional testing may be required to comply with national requirements (see also Chapter 9, Standards for screening for infectious markers).

Table 5D-3a

Parameter to be checked	Requirements	Frequency of control
ABO, RhD [a, b]	Grouping<br>Only for clinical FFP	All units
Anti-HIV 1 & 2 [a]	Negative by approved screening test	All units
HBsAg [a]	Negative by approved screening test	All units
Anti-HCV [a]	Negative by approved screening test	All units

a Unless performed on whole blood used as the source.
b Unless intended only for fractionation.

Table 5D-3b

Parameter to be checked	Requirements	Frequency of control
Volume [a]	30-40 mL	All units
Factor VIII [b]	≥ 70 IU per unit	Every 2 months:<br>a. pool of 6 units of mixed blood groups during their first month of storage<br>b. pool of 6 units of mixed blood groups during their last month of storage
Fibrinogen	≥ 140 mg per unit	1 % of all units with a minimum of 4 units per month

a This table is designed for quality control of cryoprecipitate obtained from FFP derived from one unit of whole blood. In the event that apheresis FFP is used as a starting material, the volume may be different.
b Only required if component used for treatment of haemophilia and/or vWD patients respectively.

Parameter to be checked	Requirements	Frequency of control
Von Willebrand Factor [b]	> 100 IU per unit	Every 2 months:<br>a. pool of 6 units of mixed blood groups during their first month of storage<br>b. pool of 6 units of mixed blood groups during their last month of storage

a This table is designed for quality control of cryoprecipitate obtained from FFP derived from one unit of whole blood. In the event that apheresis FFP is used as a starting material, the volume may be different.
b Only required if component used for treatment of haemophilia and/or vWD patients respectively.

Storage and transport

The stability of *Cryoprecipitate* on storage is dependent on the storage temperature. The optimal storage temperature is below – 25 °C.

Recommended storage times are:

- 36 months at or below – 25 °C;
- 3 months at – 18 °C to – 25 °C.

The storage temperature must be maintained during transport. The receiving hospital blood bank must ensure that the *Cryoprecipitate* has remained frozen during transit. Unless for immediate use, the *Cryoprecipitate* must be transferred at once to storage at the temperature stated above.

Before use, *Cryoprecipitate* must be thawed in a properly controlled environment at + 37 °C immediately after removal from storage. Dissolution of the precipitate must be encouraged by careful manipulation during the thawing procedure.

In order to preserve labile factors, *Cryoprecipitate* must be used as soon as possible following thawing. It must not be re-frozen.

Labelling

The labelling must comply with the relevant national legislation and international agreements. The following information must be shown on the label or contained in the component information leaflet, as appropriate:

- the producer's identification;
- the unique identity number. If two or more units are collected from the donor in one session, each component must have a unique component identity number;
- the name of the blood component;
- the ABO group;
- the date of preparation;
- the date of expiry;
- additional component information: leucocyte-depleted, irradiated, quarantined, etc. (if appropriate);
- the volume or weight of the blood component;
- the storage temperature;
- that the component must be administered through an approved blood administration set.

After thawing, the date of expiry must be changed to the appropriate date (and time) of expiry. The storage temperature must also be changed accordingly.

Warnings

Before use the component must be thawed in a properly controlled environment and the integrity of the pack must be verified to exclude any defects or leakages.

Cryoprecipitate is not recommended for patients with an intolerance to plasma proteins.

Adverse reactions include:

- non-haemolytic transfusion reaction (mainly chills, fever and urticaria);
- transfusion-related acute lung injury (TRALI);
- possibility of development of inhibitors to Factor VIII in patients with haemophilia;
- in rare instances, haemolysis of recipient red blood cells due to high titre allo-agglutinins in the donor has been recorded;
- viral transmission (hepatitis, HIV, etc.) is possible, despite careful donor selection and screening procedures;
- sepsis due to inadvertent bacterial contamination;
- transmission of other pathogens that are not tested for or recognised;
- citrate toxicity in neonates and in patients with impaired liver function.

4. Cryoprecipitate, Pathogen Reduced

Definition and properties

Cryoprecipitate, Pathogen Reduced is a component containing the cryoglobulin fraction of plasma obtained by further processing of *Plasma, Fresh Frozen* which is then concentrated.

It is subjected to treatment with an approved and validated Pathogen Reduction Technique (PRT) and subsequent freezing within a period of time to a temperature that adequately maintains the labile coagulation factors in a functional state. It contains a major portion of the Factor VIII, von Willebrand factor, fibrinogen, Factor XIII and fibronectin present in freshly drawn and separated plasma.

The PRT typically reduces the risk of infection by enveloped viruses (e.g. HBV, HCV, HIV) by at least one to ten-thousand-fold.

Cryoprecipitate, PR used for clinical transfusion must comply with the specifications given in this monograph.

Preparation

Plasma, Fresh Frozen is thawed, either overnight between + 2 °C to + 6 °C or by the rapid thaw-siphon thaw technique. After thawing, the component is re-centrifuged using a hard spin at the same temperature. The supernatant cryoprecipitate-poor plasma is then partially removed. The sedimented cryoprecipitate is then either rapidly frozen and kept at < – 25°C until processing by the pathogen reduction method or subject to the PRT process and then frozen.

Cryoprecipitate, PR is prepared from *Whole Blood*-derived plasma or from apheresis-derived plasma.

For the PR step, units may be treated singly or pooled.

The PRT is undertaken according to manufacturers' instructions.

Requirements and quality control

Table 5D-4a and 4b list the requirements. Additional testing may be required to comply with national requirements (see also Chapter 9, Standards for screening for infectious markers).

Table 5D-4a

Parameter to be checked	Requirements	Frequency of control
ABO, RhD [a]	Grouping	All units
Anti-HIV 1 & 2 [a]	Negative by approved screening test	All units
HbsAg [a]	Negative by approved screening test	All units
Anti-HCV [a]	Negative by approved screening test	All units

a Unless performed on donor sample of whole blood donation or apheresis donation used as the source.

Table 5D-4b

Parameter to be checked	Requirements	Frequency of control
Volume	as per system used	All units
Factor VIII	> 50 IU per single unit	Every 2 months<br>a. pool of 6 units of mixed blood groups during their first month of storage<br>b pool of 6 units of mixed blood groups during their last month of storage
Fibrinogen	> 140 mg per single unit	1 % of all units with a minimum of 4 units per month
Von Willebrand Factor	> 100 IU per single unit	Every batch for accurate labelling<br>Every 2 months<br>a. 4 units of small bags during their first month of storage<br>b. 4 units of small bags during their last month of storage

The exact number of units to be tested could be determined by statistical process control.

Storage and transport

The stability of *Cryoprecipitate, PR* on storage is dependent on the storage temperature. The optimal storage temperature is below – 25 °C.

Recommended storage times are:

- 36 months at or below – 25 °C;
- 3 months at – 18 °C to – 25 °C.

The storage temperature must be maintained during transport. The receiving hospital blood bank must ensure that the *Cryoprecipitate,*

PR has remained frozen during transit. Unless for immediate use, the *Cryoprecipitate, PR* must be transferred immediately to storage at the temperature stated above.

Before use, *Cryoprecipitate, PR* must be thawed in a properly controlled environment at + 37 °C immediately after removal from storage. Dissolution of the precipitate must be encouraged by careful manipulation during the thawing procedure.

In order to preserve labile factors, *Cryoprecipitate, PR* must be used as soon as possible following thawing. It must not be re-frozen.

Labelling

The labelling must comply with the relevant national legislation and international agreements. The following information must be shown on the label or contained in the component information leaflet, as appropriate:

- the producer's identification;
- the unique identity number;
- the name of the blood component;
- additional component information: pathogen reduced (indicating the name of the PRT used);
- the ABO group;
- the date of preparation;
- the date of expiry;
- the volume or weight of the blood component;
- the storage temperature;
- that the component must be administered through an approved blood administration set.

After thawing, the date of expiry must be changed to the appropriate date (and time) of expiry. The storage temperature must also be changed accordingly.

Warnings

Before use, the component must be thawed in a properly controlled environment and the integrity of the pack must be verified to exclude any defects or leakages. No insoluble cryoprecipitate must be visible on completion of the thaw procedure.

Cryoprecipitate, PR is not recommended for patients with an intolerance to plasma proteins.

Adverse reactions include:

- non-haemolytic transfusion reaction (mainly chills, fever and urticaria);
- transfusion-related acute lung injury (TRALI);
- development of inhibitors to Factor VIII in patients with haemophilia;
- haemolysis of recipient red blood cells due to high titre allo-agglutinins in the donor viral transmission of lipid enveloped viruses (such as hepatitis B, C, HIV, etc.) is highly unlikely after PR but transmission of non-lipid enveloped viruses (such as hepatitis A, Parvovirus B19) is possible, despite careful donor selection and screening procedures;
- sepsis due to inadvertent bacterial contamination;
- transmission of other pathogens that are not tested for or recognised;
- citrate toxicity in neonates and in patients with impaired liver function;
- transfusion associated circulatory overload (TACO);
- when prepared by amotosalen treatment in neonates undergoing phototherapy with devices that emit a peak energy wavelength less than 425 nm, and/or have a lower bound of the emission bandwidth < 375 nm;
- in a patient with known allergy to the compounds used for, or generated by, the PRT.

5. Plasma, Fresh Frozen, Cryoprecipitate-Depleted

Definition and properties

Plasma, Fresh Frozen, Cryoprecipitate-Depleted is a component prepared from *Plasma, Fresh Frozen* by the removal of the cryoprecipitate.

Its content of albumin, immunoglobulins and coagulation factors is the same as that of *Plasma, Fresh Frozen*, except that the levels of the labile Factors V and VIII are markedly reduced. The fibrinogen concentration is also reduced in comparison to *Plasma, Fresh Frozen*.

Preparation

Plasma, Fresh Frozen, Cryoprecipitate-Depleted is the by-product of the preparation of *Cryoprecipitate* from *Plasma, Fresh Frozen*.

Leucocyte depletion of the starting material and/or virus inactivation, and/or quarantine, is a requirement in some countries.

Requirements and quality control

As indicated for *Plasma, Fresh Frozen* (see Table 5D-1 above), with the additional requirement given in Table 5D-5 below. Additional testing may be required to comply with national requirements (see also Chapter 9, Standards of screening for infectious markers).

Table 5D-5

Parameter to be checked	Requirements	Frequency of control
Volume	Stated volume ± 10 %	All units

Storage and transport

The stability of *Plasma, Fresh Frozen, Cryoprecipitate-Depleted* on storage is dependent on the storage temperature. The optimal storage temperature is below – 25 °C. Approved storage times are:

- 36 months at or below – 25 °C;
- 3 months at – 18 °C to – 25 °C.

The storage temperature must be maintained during transport. The receiving hospital blood bank must ensure that *Plasma, Fresh Frozen, Cryoprecipitate-Depleted* has remained frozen during transit. Unless for immediate use, the units must be transferred at once to storage at the temperature stated above.

In order to preserve labile factors, *Plasma Fresh Frozen, Cryoprecipitate-Depleted* must be used as soon as possible following thawing. It must not be re-frozen.

Labelling

The labelling must comply with the relevant national legislation and international agreements. The following information must be shown on the label or contained in the component information leaflet, as appropriate:

- the producer's identification;
- the unique identity number. If two or more units are collected from the donor in one session, each component must have a unique component identity number;
- the ABO group;
- the date of preparation;
- the name of the anticoagulant solution;
- the name of the blood component;
- additional component information: leucocyte-depleted, irradiated, quarantined, pathogen-reduced, etc. (if appropriate);
- the date of expiry;
- the volume or weight of the blood component;
- the storage temperature;
- that the component must be administered through an approved blood administration set.

After thawing, the date of expiry must be changed to the appropriate date (and time) of expiry. The storage temperature must also be changed accordingly.

Warnings

Transfusion of ABO blood group-incompatible plasma may result in haemolytic transfusion reaction.

Plasma Fresh Frozen, Cryoprecipitate-Depleted is not recommended for patients with an intolerance to plasma proteins.

Adverse reactions include:

- non-haemolytic transfusion reaction (mainly chills, fever and urticaria);
- transfusion-related acute lung injury (TRALI);
- viral transmission (hepatitis, HIV, etc.) is possible, despite careful donor selection and screening procedures;
- sepsis due to inadvertent bacterial contamination;
- transmission of other pathogens that are not tested for or recognised;
- citrate toxicity in neonates and in patients with impaired liver function;
- transfusion-associated circulatory overload.

Component monographs
Part E. White cell components

1. Granulocytes, Apheresis

Definition and properties

Granulocytes, Apheresis is a component that contains granulocytes suspended in plasma and is obtained by apheresis of a single donor using automated cell separation equipment.

An adult therapeutic dose of *Granulocytes, Apheresis* contains between 1.5×10^8 and 3.0×10^8 granulocytes/kg body weight of the designated recipient.

Granulocytes, Apheresis has a significant content of red blood cells, lymphocytes and platelets.

Granulocytes, Apheresis must be irradiated.

IMPORTANT NOTICE

The clinical efficacy, indication and dosage of granulocyte transfusions have not been established. Potential donors of granulocytes need to receive medication before collection, and sedimenting agents are required during the apheresis procedure, both of which have potentially severe side-effects that are described below. Thus, it is essential to secure the informed consent of the donor. In addition to the recognised complications of routine donor apheresis (see Chapters 2 and 3), the following side-effects may occur.

- Hydroxyethyl starch (HES): acts as a volume expander. Donors who have received HES may experience headaches or peripheral oedema because of an expanded circulatory volume. HES may accumulate (which can result in pruritus) and allergic reactions are possible.
- Corticosteroids: may cause, for example, hypertension, diabetes, cataracts and peptic ulcer.
- G-CSF: The most common short-term complication after G-CSF administration in peripheral blood stem cell (PBSC) donors is bone pain though, on very rare occasions, splenic rupture or lung injury may occur. Concerns over acute myeloid leukaemia

(AML)/myelodysplasia (MDS) development following G-CSF administration are based primarily on reports of increased rates of AML/MDS among women with breast cancer who received chemotherapy or patients with severe chronic neutropenia (SCN) who received G-CSF support. To date, registry data from Europe and the United States have not identified any increased risk of AML/MDS (including those based on the data of over 100 000 healthy individuals who donated PBSC and received G-CSF as pre-treatment). However, the median follow-up of these studies is less than 5 years.

Preparation

Donors of *Granulocytes, Apheresis* require pre-treatment with corticosteroids and/or growth factors. *Granulocytes, Apheresis* are collected from a single donor by apheresis. Optimal collection yields require the use of a sedimenting agent, such as HES, low molecular weight dextran or modified fluid gelatine.

Requirements and quality control

Table 5E-1 lists the requirements. Additional testing may be required to comply with national requirements (see also Chapter 9, Standards for screening for infectious markers).

Table 5E-1

Parameter to be checked	Requirements	Frequency of control
ABO, RhD	Grouping	All units
Anti-HIV 1 & 2	Negative by approved screening test	All units
HBsAg	Negative by approved screening test	All units
Anti-HCV	Negative by approved screening test	All units

Parameter to be checked	Requirements	Frequency of control
HLA (when required)	Typing	As required
Volume	< 500 mL	All units
Granulocyte content	Achieve clinical dose: e.g. adult patient of 60 kg = $0.9\text{-}1.8 \times 10^{10}$ granulocytes per unit	All units

Storage and transport

Granulocytes, Apheresis are not suitable for storage and must be transfused as soon as possible after collection. If unavoidable, storage must be limited to the shortest possible period.

The unit must be transported to the user in a suitable container between + 20 and + 24 °C, but without agitation.

Labelling

The labelling must comply with the relevant national legislation and international agreements. The following information must be shown on the label or contained in the component information leaflet, as appropriate:

- the producer's identification;
- the unique identity number;
- the ABO and RhD groups;
- the date of donation;
- the name of the anticoagulant solution, additive solutions and/or other agents;
- the name of the blood component;
- additional component information: irradiated, etc. (as appropriate);
- the date of expiry (and time of expiry, when required);

- the number of granulocytes;
- the storage temperature;
- HLA type, if determined;
- that the component must be administered through an approved blood administration set.

Warnings

Because of the possibility of severe adverse effects associated with the collection (donor side-effects) and transfusion of granulocytes (recipient side-effects), the goals of granulocyte transfusion must be defined clearly before a course of therapy is initiated.

As there is a significant content of red blood cells, compatibility of donor red cells with the designated recipient must be verified by suitable pre-transfusion testing. RhD-negative female recipients of child-bearing potential must not be transfused with Granulocyte Concentrates from RhD-positive donors; if RhD-positive concentrates have to be used, the prevention of RhD immunisation by use of RhD-immunoglobulin must be considered.

Attention to HLA compatibility is also required for allo-immunised recipients.

Granulocytes, Apheresis must be irradiated.

CMV-seronegative components for CMV-seronegative recipients must be considered.

Administration through a micro-aggregate or leucocyte-reduction filter is contraindicated.

The risk of adverse reactions is increased with concomitant administration of Amphotericin B.

Adverse reactions include:

- non-haemolytic transfusion reaction (mainly chills, fever and urticaria);

- allo-immunisation against red cell antigens, HLA, HPA and HNA;
- transfusion-related acute lung injury (TRALI);
- post-transfusion purpura;
- sepsis due to inadvertent bacterial contamination;
- viral transmission (hepatitis, HIV, etc.) is possible, despite careful donor selection and screening procedures;
- syphilis transmission;
- protozoal transmission (e.g. malaria, toxoplasmosis) may occur in rare instances;
- transmission of other pathogens that are not tested for or recognised;
- citrate intoxication in neonates and in patients with impaired liver function;
- accumulation of HES in multi-exposed patients.

2. Granulocytes, Pooled

Definition and properties

Granulocytes, Pooled is a component that contains granulocytes obtained by pooling of up to 12 buffy coats, suspended in either plasma or a mixture of platelet additive solution and plasma. *Granulocytes, Pooled* contains on average 11.0×10^9 granulocytes per unit. The recommended dose for an adult is 1-2 units daily and for a child 0.3×10^9 granulocytes/kg.

Granulocytes, Pooled has a significant content of red blood cells, lymphocytes and platelets.

Granulocytes, Pooled must be irradiated.

Preparation

One method of preparation involves pooling of up to 12 ABO matched buffy coats within 18 hours of donation and platelet additive solution

added prior to centrifugation. The red cell residue, supernatant and granulocyte rich layer (buffy coat) are separated. The buffy coat is then mixed with 70 mL of ABO matched plasma from one of the donations.

An alternative method of preparation involves the use of the remaining cellular residue after preparation of *Platelets, Recovered, Pooled* from buffy coats. Two ABO matched residues are combined and diluted with saline prior to centrifugation. The red cell residue, supernatant and granulocyte rich layer (buffy coat) are separated. The buffy coat is used as such.

The component must be stored in a pack that allows gas exchange (i.e. a platelet pack).

Requirements and quality control

Table 5E-2 lists the requirements. Additional testing may be required to comply with national requirements (see also Chapter 9, Standards for screening for infectious markers).

Table 5E-2

Parameter to be checked	Requirements	Frequency of control
ABO, RhD	Grouping	All units
Anti-HIV 1 & 2	Negative by approved screening test	All units
HBsAg	Negative by approved screening test	All units
Anti-HCV	Negative by approved screening test	All units
Volume	As defined locally	All units
Granulocyte content	$> 5 \times 10^9$/unit	All units

Storage and transport

Granulocytes, Pooled are not suitable for storage and must be transfused as soon as possible after collection. If unavoidable, storage must be limited to the shortest possible period. At the very latest, transfusion should commence by midnight on the day following donation (day 1).

The unit must be transported to the user in a suitable container between + 20 and + 24 °C, but without agitation.

Labelling

The labelling must comply with the relevant national legislation and international agreements. The following information must be shown on the label or contained in the component information leaflet, as appropriate:

- the producer's identification;
- the unique identity number;
- the ABO and RhD groups;
- the date of donation;
- the name of the anticoagulant solution, additive solutions and/or other agents;
- the name of the blood component;
- additional component information: irradiated, etc. (as appropriate);
- the date of expiry (and time of expiry, when required);
- the storage temperature;
- that the component must be administered through an approved blood administration set.

Warnings

Because of the possibility of severe adverse effects associated with the transfusion of granulocytes (recipient side-effects), the goals of

granulocyte transfusion must be defined clearly before a course of therapy is initiated.

As there is a significant content of red blood cells, compatibility of donor red cells with the designated recipient must be verified by suitable pre-transfusion testing. RhD-negative female recipients of child-bearing potential must not be transfused with Granulocyte Concentrates from RhD-positive donors; if RhD-positive concentrates have to be used, the prevention of RhD immunisation by administration of anti-D immunoglobulin should be considered.

Caution is advised for patients with HLA antibodies.

CMV-seronegative components for CMV-seronegative recipients must be considered.

Administration through a micro-aggregate or leucocyte-reduction filter is contraindicated.

The risk of adverse reactions is increased with concomitant administration of Amphotericin B.

Adverse reactions include:

- non-haemolytic transfusion reaction (mainly chills, fever and urticaria);
- allo-immunisation against red cell antigens, HLA, HPA and HNA;
- transfusion-related acute lung injury (TRALI);
- post-transfusion purpura;
- viral transmission (hepatitis, HIV, etc.) is possible, despite careful donor selection and screening procedures;
- syphilis transmission;
- protozoal transmission (e.g. malaria, toxoplasmosis) may occur in rare instances;
- transmission of other pathogens that are not tested for or recognised;

- citrate intoxication in neonates and in patients with impaired liver function.

Chapter 6

Standards for blood components for intrauterine, neonatal and infant use

Specially designed blood components are required for intrauterine, neonatal and infant transfusion.

These recipients are particularly prone to the complications of cytomegalovirus infection and transfusion-associated graft versus host disease and appropriate steps are required to minimise these risks.

Methods of preparation, storage and administration of these components should be validated to ensure that the delivered potassium load is within acceptable limits.

If components are split for use in neonates and infants, each satellite pack must have a unique unit identity number that allows traceability to the source donation and to other subunits prepared from the same component.

Part A. Components for intrauterine transfusions

1. Red Cells, Leucocyte-Depleted for Intrauterine Transfusion, 412
2. Platelets, Leucocyte-Depleted for Intrauterine Transfusion, 414

Part B. Components for neonatal exchange transfusion

1. Whole Blood, Leucocyte-Depleted for Exchange Transfusion, 418

2. Whole Blood, Leucocyte-Depleted, Plasma Reduced for Exchange Transfusion, 419

3. Red Cells, Leucocyte-Depleted, suspended in Fresh Frozen Plasma, for Exchange Transfusion, 421

Part C. Components (small volume) for neonatal and infant transfusion

1. Red Cells for Neonatal and Infant Small Volume Transfusion, 426

Standards for blood components for intrauterine, neonatal and infant use
Part A. Components for intrauterine transfusions

1. Red Cells, Leucocyte-Depleted for Intrauterine Transfusion

Definition and properties

Red Cells, Leucocyte-Depleted for Intrauterine Transfusion (IUT) is a red cell component for intrauterine transfusion.

Red Cells, IUT has a haematocrit (Ht) of 0.70 to 0.85.

Red Cells, IUT contains less than 1×10^6 leucocytes per original source component.

Preparation

Red Cells IUT is prepared by the secondary processing of *Whole Blood LD*, *Red Cells LD* or *Red Cells LD-AS*. In order to achieve the required haematocrit, the storage medium is partly removed and/or exchanged for another appropriate solution.

Red Cells, IUT must be compatible with both mother and foetus. In the event that the foetal blood group is not known, a type O RhD-negative donation must be selected unless the mother has blood group antibodies that necessitate the use of another blood group. The red cells must be antigen-negative for any relevant maternal allo-antibodies.

The component must not contain irregular antibodies of clinical significance.

Red Cells, IUT must be used within five days of donation.

Red Cells, IUT must be irradiated and used within 24 hours of irradiation.

Requirements and quality control

As indicated for the source component with the following additional changes given in Table 6A-1.

Table 6A-1

Parameter to be checked	Requirements	Frequency of control
Haematocrit	0.70-0.85	All units

Storage and transport

The storage and transport conditions are as for the source components. The storage time must not be longer than 24 hours after concentration and irradiation. The component must be used within five days of donation.

Labelling

The additional and/or amended labelling requirements to those of the source component are:

- the relevant blood group phenotype if the maternal antibody is other than anti-RhD;
- the modified date and time of preparation;
- the modified date and time of expiry;
- the name of the anticoagulant or additive solution;
- additional component information: irradiated, etc. (as appropriate);
- the volume or weight of the blood component;
- the haematocrit of the blood component.

Warnings

Compatibility of this component with maternal serum/plasma must be verified by suitable pre-transfusion testing.

The rate of transfusion should be controlled to avoid excessive fluctuations in blood volume.

As the foetus is at increased risk of graft versus host disease, the component must be irradiated.

Adverse reactions:

Note: Although the component is given to the foetus, because of placental transfer adverse reactions may also affect the mother.

The general adverse reactions are outlined in the relevant source component monograph.

In addition, the foetus is especially vulnerable to:

- CMV infection;
- citrate toxicity;
- metabolic imbalance (e.g. hyperkalaemia);
- transfusion-associated circulatory overload.

2. Platelets, Leucocyte-Depleted for Intrauterine Transfusion

Definition and properties

Platelets, Leucocyte-Depleted for Intrauterine Transfusion (IUT) is a platelet component for intrauterine transfusion obtained from a single donor either by apheresis or from whole blood.

Platelets, IUT must be leucocyte-depleted, irradiated and may be hyper-concentrated.

Platelets, IUT contains from 45-85 × 10^9 platelets (on average, 70 × 10^9) in 50-60 mL of suspension medium.

Preparation

Platelets, IUT is prepared either from *Platelets, Apheresis, LD* or by leucocyte-depletion of *Platelets, Recovered* and, where appropriate, the donation is from an HPA-compatible donor.

The component can be concentrated if necessary by removing part of the supernatant solution by centrifugation. This must be followed by a 1-hour rest period.

If platelets obtained from the mother are to be transfused, then these must be depleted of plasma and re-suspended in an additive solution.

Platelets, IUT must be irradiated.

Requirements and quality control

As indicated for the source component, with the following additional standards given in Table 6A-2.

Table 6A-2

Parameter to be checked	Requirements	Frequency of control
HPA [a]	Typing	When required
Volume	50-60 mL	All units
Platelet content	45-85 $\times 10^9$ per unit	All units

a HPA typing of the selected donor, not of the individual component.

Storage and transport

Storage and transport requirements are as defined for the source component, but *Platelets, IUT* must be used within 6 hours after any secondary concentration process.

Labelling

The additional and/or amended labelling requirements to those of the source component *Platelets, IUT* are:

- if components are split for use in neonates and infants, each split must have a unique unit identity number that allows traceability to the source donation and to other subunits prepared from the same component;
- additional component information: irradiated, plasma- or supernatant-reduced, etc. (if appropriate);
- the volume or weight of the blood component;
- the platelet count;
- the date and time of expiry.

Warnings

As the foetus is at increased risk of graft versus host disease, the component must be irradiated.

The rate of transfusion must be controlled to avoid excessive fluctuations in blood volume and possible bleeding after puncture must be monitored.

Adverse reactions:

Note: Although the component is given to the foetus, because of placental transfer adverse reactions may also affect the mother.

The general adverse reactions are outlined in the relevant source component monograph.

In addition, the foetus is especially vulnerable to:

- CMV infection;
- citrate toxicity;
- transfusion-associated circulatory overload.

Standards for blood components for intrauterine, neonatal and infant use
Part B. Components for neonatal exchange transfusion

1. Whole Blood, Leucocyte-Depleted for Exchange Transfusion

Definition and properties

Whole Blood, Leucocyte-Depleted for Exchange Transfusion (ET) corresponds to *Whole Blood, LD* with the properties as defined in the relevant monograph, selected for neonatal exchange transfusion to be transfused within five days of donation.

Preparation

If the maternal antibody is anti-RhD, the component is prepared from type O RhD-negative red cells. If the maternal antibody is other than anti-RhD, red cells are selected that are antigen-negative for any relevant maternal allo-antibodies.

Whole Blood, ET must be irradiated:

- if there is a prior history of intrauterine transfusion;
- for all other patients, unless compelling clinical circumstances indicate that delay would compromise the clinical outcome.

Whole Blood, ET must be used within 24 hours of irradiation.

Requirements and quality control

As indicated for *Whole Blood, LD.*

Storage and transport

The storage and transport of *Whole Blood, ET* is as described in the monograph for *Whole Blood, LD.*

The storage time must not be longer than 24 hours after irradiation and five days from donation.

Labelling

Additional and/or amended labelling requirements to those of *Whole Blood, LD* are:

- blood group phenotype, if the antibody is other than anti-RhD;

- the modified date and time of expiry;
- additional component information: irradiated, etc. (as appropriate).

Warnings

Blood group compatibility with any maternal allo-antibodies is essential. The rate of transfusion must be controlled to avoid excessive fluctuations in blood volume.

Adverse reactions:

In addition to the adverse reactions identified for Whole Blood, LD, particular concerns in the context of newborns undergoing exchange transfusion are:

- metabolic imbalance including: citrate toxicity, hypocalcaemia, hyperkalaemia, hypoglycaemia, hypokalaemia;
- thrombocytopaenia;
- cytomegalovirus infection;
- graft versus host disease, unless irradiated;
- transfusion-associated circulatory overload;
- haemolytic transfusion reaction;
- hypothermia.

2. Whole Blood, Leucocyte-Depleted, Plasma Reduced for Exchange Transfusion

Definition and properties

Whole Blood, Leucocyte-Depleted, Plasma Reduced for Exchange Transfusion (PR, ET) is *Whole Blood, ET* with a proportion of the plasma removed.

Preparation

Whole Blood, LD is selected within five days from donation and a proportion of the plasma is removed to achieve a clinically prescribed haematocrit.

If the maternal antibody is anti-RhD, the component is prepared from a type O RhD-negative donation. If the maternal antibody is other than anti-RhD, red cells are selected that are antigen negative for any relevant maternal allo-antibodies.

Whole Blood, PR, ET must be irradiated:

- if there is a prior history of intrauterine transfusion;
- for all other patients, unless compelling clinical circumstances indicate that delay would compromise the clinical outcome. *Whole Blood, PR, ET* must be used within 24 hours of irradiation.

Requirements and quality control

As indicated for *Whole Blood, LD*, with the following additional standards.

Table 6B-2

Parameter to be checked	Requirements	Frequency of control
Haematocrit	As clinically prescribed or locally defined	All units

Storage and transport

The storage and transport of *Whole Blood, PR, ET* is as in the monograph described for *Whole Blood, LD.*

The storage time must not be longer than 24 hours after irradiation and 5 days from donation.

Labelling

Additional and/or amended labelling requirements to those of *Whole Blood, LD* are:

- blood group phenotype, if the antibody is other than anti-RhD;
- the modified date and time of expiry;
- additional component information: irradiated, haematocrit, etc. (as appropriate).

Warnings

Blood group compatibility with any maternal allo-antibodies is essential. The rate of transfusion must be controlled to avoid excessive fluctuations in blood volume.

Adverse reactions:

In addition to the adverse reactions identified for *Whole Blood, LD*, particular concerns in the context of newborns undergoing exchange transfusion are:

- metabolic imbalance including: citrate toxicity, hypocalcaemia, hyperkalaemia, hypoglycaemia, hypokalaemia;
- thrombocytopaenia;
- cytomegalovirus infection;
- graft versus host disease, unless irradiated;
- transfusion-associated circulatory overload;
- haemolytic transfusion reaction;
- hypothermia.

3. Red Cells, Leucocyte-Depleted, suspended in Fresh Frozen Plasma, for Exchange Transfusion

Definition and properties

Red Cells, Leucocyte-Depleted, suspended in Fresh Frozen Plasma, for Exchange Transfusion (Red Cells, in FFP, ET) is a reconstituted

component derived from *Red Cells, LD* or *Red Cells, LD-AS* to which *Plasma, Fresh Frozen* is added.

Preparation

Red Cells, LD or *Red Cells, LD-AS* are selected within 5 days from collection for secondary processing. The supernatant containing the additive solution and/or plasma is removed after centrifugation, and then thawed fresh frozen plasma is added to reach the clinically required haematocrit.

If the maternal antibody is anti-RhD, the component is prepared from type O RhD-negative red cells. If the maternal antibody is other than anti-RhD, red cells are selected that are antigen-negative for any relevant maternal allo-antibodies. The red cells and FFP must be ABO-compatible with both mother and infant.

Red Cells, in FFP, ET must be irradiated:

- if there is a prior history of prior intrauterine transfusion;
- for all other patients, unless compelling clinical circumstances indicate that delay would compromise the clinical outcome.

Red Cells, in FFP, ET must be used within 24 hours of irradiation.

Requirements and quality control

As indicated for the source components (*Red Cells, LD*; *Red Cells, LD-AS* and *FFP*), with the following additional standards given below (Table 6B-3).

Table 6B-3

Parameter to be checked	Requirement	Frequency of control
Haematocrit	As clinically prescribed or locally defined	All units

Storage and transport

The storage and transport of *Red Cells, in FFP, ET* is as in the monograph described for *Red Cells, LD* or *Red Cells, LD-AS*.

In addition, storage time must not be longer than 24 hours after reconstitution and irradiation and 5 days from the red cell donation.

Labelling

The additional and/or amended labelling requirements to those of the reconstituting components are:

- a new unique identity number by which the source donation identity numbers must be traceable;
- the name of the blood component;
- the ABO and RhD group of the red cells;
- blood group phenotype, if the antibody is other than anti-RhD;
- the date and time of preparation;
- the new date and time of expiry;
- additional component information: irradiated, haematocrit, etc. (as appropriate).

Warnings

Compatibility of *Red Cells, in FFP, ET* with the intended recipient must be verified by suitable pre-transfusion testing. Blood group compatibility with any maternal antibodies is essential.

The rate of transfusion must be controlled to avoid excessive fluctuations in blood volume.

Adverse reactions:

The side-effects correspond to those of the two constituting components.

Particular concerns in the context of newborns undergoing exchange transfusion are:

- metabolic imbalance including: citrate toxicity, hypocalcaemia, hyperkalaemia, hypoglycaemia, hypokalaemia;
- thrombocytopaenia;
- cytomegalovirus infection;
- graft versus host disease, unless irradiated;
- transfusion-associated circulatory overload;
- haemolytic transfusion reaction;
- hypothermia.

Standards of blood components for intrauterine, neonatal and infant use
Part C. Components (small volume) for neonatal and infant transfusion

1. Red Cells for Neonatal and Infant Small Volume Transfusion

Definition and properties

Red Cells for Neonatal and Infant Small Volume Transfusion is a red cell component derived from *Red Cells, BCR*; *Red Cells, BCR-AS*; *Red Cells, LD*; or *Red Cells, LD-AS*, which is divided into satellite units.

The properties are those of the source component.

Preparation

Red Cells for Neonatal and Infant Small Volume Transfusion are prepared by the secondary processing of *Red Cells, BCR*; *Red Cells, BCR-AS*; *Red Cells, LD*; or *Red Cells, LD-AS*. The selected component is divided into 3 to 8 satellite packs by using a closed or functionally closed system.

The component may be irradiated where clinically indicated.

Quality control

Quality control of the primary source component is described in the relevant component monograph standards. Additional quality control of the final component is given in Table 6C-1.

Table 6C-1

Parameter to be checked	Requirement	Frequency of control
Volume	25-100 mL per unit	All units

Storage and transport

Storage and transport requirements are as described for the primary source red cell component.

The storage time must not exceed that of the original component.

The component may be irradiated at any time up to 28 days following collection so long as the component is transfused immediately following irradiation. If the irradiated component is to be stored then irradiation may be undertaken up to 14 days following collection and the component stored for up to 48 hours. This period may be extended to 14 days when effective mechanisms are in place to avoid such units being transfused in large volume and / or rapid transfusion clinical settings.

Labelling

The additional and/or amended labelling requirements to those of the primary red cell component are:

- if components are split for use in neonates and infants, each satellite pack must have a unique unit identity number which allows traceability to the source donation and to other subunits prepared from the same component;
- the name of the blood component;
- additional component information: irradiated, etc. (if appropriate);
- the volume or weight of the component;
- the date and time of expiry.

Warnings

Transfusion rates must be carefully controlled.

Red Cells for Neonatal and Infant Small Volume Transfusion must not be used for rapid transfusion or large volume transfusion, unless used within 5 days from the source red cell donation.

Adverse reactions:

Adverse reactions are those of the primary component selected for secondary processing.

In addition, of particular concern for infants and neonates are:

- metabolic imbalance (e.g. hyperkalaemia in massive transfusion or if rapidly transfused);
- citrate toxicity;
- transfusion-associated circulatory overload;
- cytomegalovirus infection;
- graft versus host disease, unless the component is irradiated.

Chapter 7

Standards for autologous pre-deposit transfusion

1. Overview

Pre-deposit autologous donation (PAT) means blood and blood components collected from an individual and intended solely for subsequent autologous transfusion or other human application in that same individual, i.e. a transfusion in which the donor and the recipient are the same person.

The Standards for allogeneic whole blood and component donations also apply to PAT and derived components, with the following exemptions for donor selection:

- age and body weight;
- haemoglobin level;
- protein level;
- donor platelet level.

Autologous donation must be performed in or under the control of a blood establishment.

2. Selection of patients for PAT and blood collection

Role of the physician in charge of collection

The physician in charge of blood collection has ultimate responsibility for ensuring that the patient's clinical condition allows pre-operative blood donation.

Where autologous donation is contraindicated, the physician in charge of blood collection must inform the patient and the physician in charge of the patient.

Information for donors

Written informed consent must be obtained from the patient by the physician in charge of the blood collection, who must provide the patient with the following information:

- the reasons for requiring a medical history;
- the nature of the procedure and its risks and benefits;
- the possibility of deferral and the reasons why this might occur;
- the tests that are performed and why, and that a positive test for mandatory microbiological markers results in the destruction of the collected unit;
- the significance of 'informed consent';
- the possibility that the pre-deposit donations may not suffice and that allogeneic transfusion may be additionally required;
- that unused blood is discarded and is not transfused to other patients and why this is the case.

In the case of a paediatric patient, the information must be provided both to the child and the parents, and the parents must give written informed consent.

Contraindications or deferral criteria

Active bacterial infection is an absolute contraindication.

Serious cardiac disease, depending on the clinical setting of blood collection, is a relative contraindication (see Principles).

Patients positive for infectious disease markers (as for allogeneic donations) must not be included in a PAT donation programme unless no compatible allogeneic blood is available.

3. Preparation, storage and distribution of pre-deposit autologous blood components

Blood typing and microbiological screening

Blood typing and microbiological screening must be carried out according to the minimum requirements for the equivalent allogeneic components.

Patients positive for infectious disease markers must not be included in pre-deposit autologous programmes, unless no compatible allogeneic blood is available.

Preparation

Autologous blood must be processed as for the equivalent allogeneic components.

Labelling

In addition to the labelling information described for allogeneic components, labels on pre-deposit autologous donations must have:

- the statement: AUTOLOGOUS DONATION;
- the statement: STRICTLY RESERVED FOR;
- family name and first name;
- date of birth;
- identity number of the patient.

Storage and handling

Pre-deposit autologous blood components must be stored, transported and distributed under the same conditions as, but clearly separated from, the equivalent allogeneic components.

Warnings

Release procedures must include a confirmation of identity:

- on the component labels;
- on the prescription document;
- and at the bedside.

Pre-transfusion infectious disease marker tests must be carried out as described for allogeneic components.

Untransfused autologous blood components must not be used for allogeneic transfusion or for plasma for fractionation.

Chapter 8

Standards for immunohaematology

1. Overview

Standards in this chapter apply to the immunohaematology testing of donors, donations and patients, whether by serological or molecular methods.

2. Selection and validation of reagents and methods

All laboratory testing procedures must be validated before use (Directive 2005/62/EC Annex 6.3.1).

There must be data confirming the suitability of any laboratory reagents used in the testing of donor samples and blood component samples (Directive 2005/62/EC Annex 6.3.4).

Only test reagents that have been licensed or evaluated and considered suitable by a responsible National Health Authority must be used. In the EU, such reagents are considered as *in vitro* diagnostic devices and must be CE marked. In-house manufactured reagents may be used for rare occasions (e.g. blood group genotyping of high- or low-frequency antigens where commercial CE-marked reagents are not available).

EU Directive 98/79/EC classifies ABO, Rh (C, c, D, E, e) anti-Kell reagents in list A of Annex II. The manufacturer of such reagents must have a full quality system certified by an authorised body and must submit an application containing all the control results for each lot.

Blood group testing must be undertaken in accordance with the instructions provided by the manufacturer of the reagents and kits.

All techniques and modifications to techniques in use must be validated.

The validation of reagents must detect deviations from the established minimal quality requirements (specifications).

Prospective purchasers must require potential suppliers to provide them with full validation data for all lots of reagents. Each lot of reagent must be qualified by the purchaser to demonstrate suitability for its intended purpose within the system used for testing.

There must be a reliable process in place for transcribing, collating and interpreting results.

3. Quality control

The quality of the laboratory testing must be regularly assessed by the participation in a formal system of proficiency testing, such as an external quality assurance programme (Directive 2005/62/EC Annex 6.3.5).

Quality-control procedures must be implemented for the equipment, reagents and techniques used for ABO, RhD and other blood-group antigen typing and detection and identification of antibodies. The frequency of the control is dependent on the method used.

4. Blood group testing of blood donors and donations

Each donation must be tested in conformity with the requirements laid down in Annex IV to Directive 2002/98/EC (Directive 2005/62/EC Annex 6.3.2).

Blood group serology testing must include procedures for testing specific groups of donors (e.g. first-time donors, donors with a history of transfusion) (Directive 2005/62/EC Annex 6.3.6).

ABO and RhD

The ABO and RhD labelling of blood components of all first-time donors must be based upon the results of two independent ABO and

RhD tests with two independent reagents. At least one of the ABO tests must include reverse grouping.

A positive RhD test must lead to labelling of the unit as 'RhD positive'. Components must be labelled as 'RhD negative' only if the donor has tested negative for RhD using appropriate reagents or tests specifically selected to detect weak D and D variants.

ABO and RhD testing must be performed on all donations excepted for plasma intended only for fractionation.

The ABO and RhD blood group must be verified on each subsequent donation and a comparison must be made with the historically determined blood group.

If a discrepancy is found, the applicable blood components must not be released until the discrepancy is unequivocally resolved.

Additional typing

If additional typing is performed then, before the result of the confirmed phenotype is printed on the label, a test must be done at least twice from two different samples collected from two different donations. In the absence of historic information, two tests carried out independently on the current donation allow the phenotype to be printed on the label of that donation – but not on subsequent donations without further testing. The results should be linked to the donor record.

Irregular antibody testing

All first-time donors as well as repeat and regular donors with a history of pregnancy or transfusions since the last donation must be tested for clinically significant irregular red cell antibodies.

5. Testing of patient samples

The ABO and RhD blood group and, when needed, other blood types must be determined on the patient's blood sample before issuing components for transfusion. In an emergency situation, when a delay may be life-threatening, components may be issued before all results

of grouping and antibody screening are completed. In these situations, testing must be completed as soon as possible.

A sample of the patient's plasma/serum used for compatibility testing and/or antibody screening must be retained for a defined period of time.

Blood grouping and antibody detection

The laboratory must have a reliable and validated procedure for blood grouping and antibody detection that includes an effective mechanism to verify the accuracy of the data at the time of issuing a report on the blood group and other test findings for inclusion in the patient's record.

Sufficiently sensitive techniques for the detection of clinically significant red cell allo-antibodies must be used, including reagent red cells that cover all appropriate antigens, preferably with homozygous expression for the most clinically significant allo-antibodies.

Compatibility testing

Compatibility between red cell components and the recipient's plasma/serum must be assured for transfusions. Sufficiently sensitive techniques for the detection of clinically significant red cell allo-antibodies must be used. Laboratory records of the tests performed and of the destination of all units handled (including patient identification) must be kept.

Compatibility testing must be carried out on a sample taken no more than 3 days before the proposed transfusion for patients who have been transfused or have become pregnant during the last 3 months.

Serological compatibility testing

Serological compatibility testing must be performed if clinically significant red cell allo-antibodies are suspected or have been identified by current or previous testing. Whenever possible, red cell components that lack the corresponding antigens must be selected for transfusion, and a serological compatibility test between donor red

cells and recipient plasma/serum must be undertaken before issuing red cell components for transfusion.

Type and screen

A type and screen procedure may be used as replacement for serological compatibility testing if antibody screening has not detected clinically significant red cell antibodies. The process must include:

- reagent red cells that cover all clinically significant antigens (preferably with homozygous expression) must be used for antibody detection;
- a reliable and validated procedure (preferably by computer) that ensures compatibility between the donor red blood cells and recipient plasma.

Chapter 9

Standards for screening for infectious markers

1. Selection and validation of infectious marker tests

All laboratory testing procedures must be validated before use (Directive 2005/62/EC Annex 6.3.1).

Each donation must be tested, in conformity with the requirements laid down in Annex IV to Directive 2002/98/EC (Directive 2005/62/EC Annex 6.3.2).

There must be data confirming the suitability of any laboratory reagents used in the testing of donor samples and blood component samples (Directive 2005/62/EC Annex 6.3.4).

The quality of the laboratory testing must be regularly assessed by participation in a formal system of proficiency testing, such as an external quality assurance programme (Directive 2005/62/EC Annex 6.3.5).

Only tests that have been licensed or evaluated and considered suitable by the responsible health authorities can be used. In the EU, these reagents are considered as *in vitro* diagnostic devices and must be CE marked. EU Directive 98/79/EC classifies the HIV, HTLV, hepatitis B and hepatitis C screening tests in list A. The manufacturer must have a full quality system certified by an authorised body and must submit an application containing all the control results for each lot.

Screening tests for infectious markers must be performed in accordance with the instructions provided by the manufacturer of the reagents and test kits.

All laboratory assays and test systems for infectious disease marker screening, including any upgrades from the manufacturer, used by blood establishments must be validated before introduction to ensure compliance with the intended use of the test.

Correct determination of negative and positive controls, as provided by and in accordance with the manufacturer's instructions, is a minimum requirement.

2. Mandatory serological screening tests

The minimum mandatory serological blood donor screening tests are:

- antibody to HIV-1 (anti-HIV-1) and HIV-2 (anti-HIV-2), including outlying types (e.g. HIV-1 type O);
- antibody to hepatitis C virus (anti-HCV);
- a hepatitis B surface antigen (HBsAg) assay, which will detect at least 0.13 IU/mL of HBsAg.

Appropriate quality control measures must be in place when screening for infectious markers. Specific requirements are shown in Table 9.1.

Table 9.1. Quality control of mandatory serological screening tests

Parameter to be checked	Requirement	Frequency of control
Anti-HIV 1/2 screening sensitivity	Detection of weak positive serum [a]	Each plate/run
Anti-HCV screening sensitivity	Detection of weak positive serum [a]	Each plate/run
HBsAg screening test	Detection of 0.5 IU/mL standard	Each plate/run

a Where possible, the weak positive control should not be the one provided by the manufacturer.

Laboratories undertaking infectious disease testing of blood donations must participate in a regular external quality assurance programme.

3. Additional serological screening tests

National authorities may also require additional screening tests for donations resulting in blood components for direct clinical use such as: *Treponema pallidum* haemagglutination assay (TPHA); IA for syphilis; antibody to human T-cell lymphotropic virus types I (anti-HTLV-1) and II (anti-HTLV-II); antibody to hepatitis B core antigen (anti-HBc). These tests are not required for plasma for fractionation.

Appropriate quality control measures must be in place when screening for infectious markers. Specific requirements are shown in Table 9.2.

Table 9.2. Quality control of additional serological screening

Parameter to be checked	Requirement	Frequency of control
Syphilis: TPHA or another IA	Detection of weak positive serum [a]	Each plate/run
Anti-HTLV I/II screening test	Detection of weak positive serum [a]	Each plate/run
Anti-HBc screening test	Detection of weak positive serum [a]	Each plate/run

a Where possible, the weak positive control should not be the one provided by the manufacturer.

4. Management of reactive results in serological screening tests

There must be clearly defined procedures to resolve discrepant results and ensure that blood and blood components that have a repeatedly reactive result in a serological screening test for infection with the viruses mentioned in Annex IV to Directive 2002/98/EC must be excluded from therapeutic use and be stored separately in a dedicated environment. Appropriate confirmatory testing must take place. In case of confirmed positive results, appropriate donor management must take place, including the provision of information to the donor and follow-up procedures (Directive 2005/62/EC Annex 6.3.3).

Initially reactive donations must be retested in duplicate. If any of the repeat tests are reactive, then the donation is deemed repeatedly

reactive. The donation must not be used for transfusion or the manufacture of medicinal products. Confirmatory testing must be performed by a certified/accredited laboratory.

Algorithms to enable consistent resolution of repeatedly reactive donations must be in place. In the event that a repeatedly reactive donation is confirmed positive, the donor must be notified and a further sample must be obtained to reconfirm the results and the identity of the donor. The results of confirmatory testing that present evidence of on-going infection must be discussed with the donor and the donor must be deferred from donation and referred for appropriate care.

The above rules do not necessarily apply to all donations found repeatedly reactive for anti-HBc. Additional testing, e.g. for HBs-antibody and/or HBV-DNA might enable some repeatedly reactive donations to be used clinically.

If a confirmed infection by HBV, HCV or HIV is shown in a repeat donor, the blood establishment must undertake a look-back procedure to identify previous potentially infectious donations. If so, the look-back procedure must ensure that:

- the blood establishment informs the hospital in writing about the incident and advises the hospital to trace the recipient(s) of the implicated blood component(s) and to inform the treating physician about the potentially infectious transfusion;
- the relevant organisation that carried out plasma fractionation is notified;
- if the recipient is confirmed to be positive for the given infection, the incident is reported to the national haemovigilance system and/or Competent Authority.

5. Nucleic acid screening

If screening of blood donations by Nucleic Acid Amplification Techniques (NAT) is required by national authorities for the release of blood components, the NAT assays must be validated to detect 5 000 IU/mL for HCV-RNA, 10 000 IU/mL for HIV-RNA and

100 IU/mL for HBV-DNA in single donations. Appropriate quality control measures must be in place. The specific requirements are shown in Table 9.3.

Table 9.3. Quality control of HBV, HCV and HIV nucleic acid amplification techniques

Parameter to be checked	Requirement	Frequency of control
HBV-NAT	Detection of 100 IU/mL HBV-DNA per donation	Internal control for each NAT reaction
HCV-NAT	Detection of 5 000 IU/mL HCV-RNA per donation	Internal control for each NAT reaction
HIV-NAT	Detection of 10 000 IU/mL HIV RNA per donation	Internal control for each NAT reaction

6. Selective screening of donations

Screening of selected donations for antibodies to cytomegalovirus (anti-CMV) may be undertaken. When performed the assay and test system must be fully validated. Confirmation of reactive results and notification of reactive donors is not necessary.

Screening for additional infectious agents, such as malaria antibody, *T. Cruzi* antibody and West Nile Virus NAT or others may be appropriate to reduce the risk of transfusion-transmitted infections in selected donor populations.

When selective screening is in place the assay and test system must be fully validated.

Appropriate quality control measures must be in place when screening for infectious markers. Specific requirements are shown in Table 9.4.

Table 9.4. Quality control of selective screening tests

Parameter to be checked	Requirement	Frequency of control
Anti-CMV screening test	Detection of weak positive serum [a]	Each plate/run
Malaria antibody test	Detection of weak positive serum [a]	Each plate/run
T. Cruzi antibody test	Detection of weak positive serum [a]	Each plate/run
West Nile virus NAT	Detection of 250 copies WNV RNA per mL in each donation	Internal control for each NAT reaction

a Where possible the weak positive control should not be the one provided by the manufacturer.

Chapter 10

Standards for haemovigilance

1. Overview

Haemovigilance procedures must be in place to ensure the organised surveillance of serious adverse or unexpected events or reactions in recipients of blood and blood components and for the epidemiological assessment of infections in donors.

The results of haemovigilance analyses must be fed back periodically to the providers of haemovigilance data and communicated to the field and to the relevant Competent Authorities, together with recommendations for preventive or corrective measures.

2. Prerequisites for implementation of a haemovigilance network

Haemovigilance must be the shared responsibility of the professionals in the field and the Competent Authorities for blood safety.

Traceability of blood components

There must be procedures in place to ensure full traceability, allowing the tracing of each individual unit of blood (or any blood components derived from it), from the donor to its final destination (whether this

is a patient, a manufacturer of medicinal products or disposal) and *vice versa*.

Traceability must also cover cases in which the blood unit or component is not transfused to a patient, but is used for the manufacturing of medicinal products or for research and investigational purposes, or if it is disposed of.

Hospitals must inform the blood establishment whenever a recipient of blood components has a serious adverse reaction, indicating that a blood component may have been the cause.

Confidentiality of haemovigilance data

Any database of haemovigilance reports must operate in compliance with applicable regulations on the confidentiality of individual medical patient and donor data. Individual reports must be anonymised.

3. Device defects

When a causality assessment suggests that a medical device (including *in vitro* diagnostics) had a possible role in causing an adverse reaction/event, the manufacturer or his authorised representative must be notified at the same time as the national health authority, even if at the time of reporting full causality has not necessarily been established. When haemovigilance and medical device vigilance are the responsibility of separate entities, both the health authority responsible for haemovigilance and the health authority responsible for medical device vigilance must be notified.

4. Post-transfusion infection reported to the blood establishment

Hospitals must inform the blood establishment whenever a recipient of blood components develops laboratory tests results and/or disease symptoms, indicating that a blood component may have been infectious for hepatitis (B or C), HIV or other transmissible agents.

The blood establishment must request relevant information from the hospital about the infection and the course of disease in the recipient and possible risk factors in the recipient for the infection.

The blood establishment's physician must establish a plan of investigation, the results of which must be recorded.

The incident must be reported to the Competent Authorities.

APPENDIX 1.
KEY CRITERIA FOR DONOR ELIGIBILITY

The Standards require medical assessment to be undertaken on all prospective donors using a combination of interview, questionnaire and, if necessary, further direct questions. The questionnaire must be designed to elicit information on the health and lifestyle on the donor that may adversely affect the safety of both the recipient and the donor.

Blood establishments should develop a questionnaire that is appropriate for local circumstances. Therefore, it is not possible to provide a generic questionnaire in this *Guide*.

Instead, key eligibility topics for donor inclusion in the questionnaire or direct questions in an interview have been developed and are included in the table below.

Key eligibility topics identified as being critical for the safety of donors and recipients are labelled as 'core'. It is recommended that countries include a question which meets the described intent of the core topics for donor eligibility in their donor questionnaire. Examples of such questions are included, but the wording may be changed provided the question still meets the described intent.

A number of key eligibility topics have also been identified that may be considered to be important for the safety of donors and recipients dependent on local arrangements and circumstances in blood establishments. These are labelled as 'optional'. The blood establishment may choose to include or not include such questions.

Core and optional sample questions have been categorised into those which apply only to first-time and repeat donors, and those which also apply to regular donors.

Blood establishments may also choose to include additional questions.

These recommendations are intended as a guide. Final responsibility for the content of the donor questionnaire lies with the blood establishment and Competent Authorities.

Key evaluative topic for donor eligibility	Intent of question	Core sample question	Optional sample question	First-time & repeat donors	Regular donors
GENERAL – health	To assess general health and provide the donor with an opportunity to volunteer health issues that may not be addressed by specific questions.	Are you in good health?		Y	Y
GENERAL – previous donation history	A donor who has previously volunteered to donate should have a record, which may contain important information regarding their ongoing eligibility. Countries with more than one blood establishment could also have donors who present at different establishments.	Have you ever volunteered to donate blood before?<br>If yes: where/when?		Y	N
GENERAL – previous deferral	To identify people who have been permanently deferred from donating blood previously.	Have you previously been told not to give blood?		Y	N

Key evaluative topic for donor eligibility	Intent of question	Core sample question	Optional sample question	First-time & repeat donors	Regular donors
GENERAL – weight	Total blood volume is proportional to donor weight. Donors must weigh at least 50 kg to safely donate blood.		Is your weight over 50 kg?	Y	Y
GENERAL – donor comprehension	Efficacy of the donor interview process requires the donor to firstly understand the questions being asked of him/her and then to truthfully and accurately complete the questionnaire to the best of his/her knowledge.<br><br>NOTE: If not included as an optional question, then Blood Establishments should include as part of the donor declaration to assist gaining written informed consent.		Have you read and understood the above questions and do you affirm that you have answered the questions truthfully and to the best of your knowledge?	Y	Y

Key evaluative topic for donor eligibility	Intent of question	Core sample question	Optional sample question	First-time & repeat donors	Regular donors
SERIOUS ILLNESS – examples	To capture any history of serious illness, using examples of common and important serious illnesses that have implications for donor and/or recipient safety. Each example listed would require deferral or further assessment of eligibility.	Have you ever suffered from any serious illness? Examples include:<br>• jaundice, malaria, tuberculosis, rheumatic fever?<br>• heart disease, high or low blood pressure?<br>• severe allergy, asthma?<br>• convulsions or diseases of the nervous system?<br>• chronic diseases such as diabetes or malignancies?		Y	N
SERIOUS ILLNESS – physician and hospital visits	Illness that is serious enough to require medical consultation may be relevant to donor selection.	Since your last donation, have you been to see a doctor or to hospital?		N	Y

Key evaluative topic for donor eligibility	Intent of question	Core sample question	Optional sample question	First-time & repeat donors	Regular donors
HAZARDOUS OCCUPATIONS & HOBBIES	To identify donors with occupations or hobbies that may put them or other people at risk in the event of a delayed vasovagal reaction following blood donation.		Do you have a hazardous occupation or hobby such as driving public transport, operating heavy machinery, underwater diving and piloting a plane or other activities?	Y	Y

Key evaluative topic for donor eligibility	Intent of question	Core sample question	Optional sample question	First-time & repeat donors	Regular donors
PREGNANCY	To protect donors from iron depletion +/- risk of vasovagal reaction in late pregnancy. Donors who have recently become pregnant should be deferred temporarily to allow time for iron stores to replenish.	For women: Are you or have you become pregnant in the previous 6 months?		Y	Y
	To identify donors whose blood donations may contain HLA or granulocyte anti-bodies and thereby pose a higher risk of TRALI. These antibodies may develop in response to exposure to foetal antigens during pregnancy.		Have you ever been pregnant?	Y	N

Key evaluative topic for donor eligibility	Intent of question	Core sample question	Optional sample question	First-time & repeat donors	Regular donors
MEDICATIONS – general	Medications may render blood donations partly or completely unsuitable for use. This question also serves as an additional prompt for underlying disease, and therefore the indications for each medication should also be determined.	Have you taken any medications recently?		Y	Y
MEDICATIONS<br>Platelet affecting drugs	Some medications affect platelet function. This question can also serve to capture chronic pain or inflammation.		In the last 48 hours have you taken any aspirin, pain killers or anti-inflammatory medications?	Y	Y

Key evaluative topic for donor eligibility	Intent of question	Core sample question	Optional sample question	First-time & repeat donors	Regular donors
MEDICATIONS – teratogenic	Medications with known teratogenic potential require donor deferral to cover the maximum potential period that the drug will circulate in the donor's peripheral blood, with a subsequent risk if the donation is transfused to a pregnant recipient.	Have you ever had medication with:<br>• isotretinoin (eg Accutane R)<br>• etretinate (eg Tigason R)<br>• acitretin (eg Neotigason R)<br>• finasteride (eg Proscar R, Propecia R)<br>• dutasteride (eg Avodart R)		Y	Y
MEDICATIONS – vaccinations	Recent vaccination may harm immunocompromised blood recipients through the transmission of live/attenuated pathogens, and may also interfere with the interpretation of donor screening tests, such as HBsAg.	Have you had any vaccinations in the last 8 weeks?		Y	Y
BLOOD-BORNE RISKS – intravenous use of drugs	Injecting drug use is an important route of transmission for blood-borne infections including HIV, hepatitis B and C.	Have you ever used needles to take drugs, steroids, or anything not prescribed by your doctor?		Y	Y

Key evaluative topic for donor eligibility	Intent of question	Core sample question	Optional sample question	First-time & repeat donors	Regular donors
SEXUAL ACTIVITY – sex worker	In many countries, sex workers have a significantly higher prevalence of blood-borne and sexually transmitted infections than the general population.	Have you ever received payment (gifts, money or drugs) for sex?		Y	Y
SEXUAL ACTIVITY – male to male sex	Male to male sex is associated with a higher risk of HIV. This group also has a higher risk of syphilis, gonorrhoea, as well as infection by hepatitis B and hepatitis A viruses.	For men: have you had male to male sex in the *[specified time period]*?<br>*(For the purpose of this question, sex is defined as oral or anal inter-course with or without a condom.)*		Y	Y
SEXUAL ACTIVITY – female partner of man who has sex with men	Men who have sex with men have a higher risk of HIV infection and other sexually transmitted diseases. Therefore, women who have sexual contact with men in this group have a higher risk of such diseases than other women.	For women: to the best of your knowledge, has any man with whom you have had sex in the *[specified time period]* ever had sex with another man?<br>*(For the purpose of this question, sex is defined as oral, vaginal or anal intercourse with or without a condom.)*		Y	Y

Key evaluative topic for donor eligibility	Intent of question	Core sample question	Optional sample question	First-time & repeat donors	Regular donors
SEXUAL ACTIVITY – at-risk sexual partner	A donor with a known history of sexual contact with persons in these risk groups has a higher risk of infection by HIV and/or hepatitis.	In the past *[specified time period]* have you had sexual contact with someone who:<br>• is HIV positive or has hepatitis?<br>• has ever used needles to take drugs, steroids, or anything not prescribed by his/her doctor?<br>• receives or has received payment (gifts, money or drugs) for sex?		Y	Y
	Donors who have had sex with a new sexual partner may be at higher risk of infection by HIV and other sexually transmitted diseases.		Have you had sex with a new partner within the past 4 months?	Y	Y
	Some countries have a high prevalence of HIV. Sexual contact with residents or former residents of those countries is a risk factor for HIV exposure.		Since your last donation (or, if a new donor, in the last 12 months) have you had sex with a new partner who currently lives or previously lived in another country?	Y	Y

Key evaluative topic for donor eligibility	Intent of question	Core sample question	Optional sample question	First-time & repeat donors	Regular donors
TRAVEL – entry question	Several infectious diseases relevant to blood safety are restricted to certain geographical regions. These include variant Creutzfeldt-Jakob disease (vCJD), malaria, Chagas disease, and other vector-borne diseases such as West Nile virus, dengue fever and chikungunya.	Were you born or have you lived and/or travelled abroad?		Y	Y
TRAVEL – malaria semi-immunity	A country without endemic malaria can use this question to flag for possible malaria semi-immunity.	Have you ever spent a continuous period of 6 months or more abroad?<br>If so, check whether the donor spent any continuous period of 6 months or more in a malaria-endemic area.		Y	Y

Key evaluative topic for donor eligibility	Intent of question	Core sample question	Optional sample question	First-time & repeat donors	Regular donors
TRAVEL – malaria exposure	A donor who visits a malaria risk area could harbour asymptomatic infection after returning to their country of residence.	Have you been abroad since your last donation (or, for new donors, in the last 12 months)?<br>If so, check whether the donor visited any malaria-endemic areas.		Y	Y
TRAVEL – unexplained fever	A donor who visits a malaria risk area could harbour asymptomatic infection after returning to their country of residence.		Have you ever had an unexplained fever after travelling abroad?<br>If so, check whether it was within 6 months of visiting a malaria-endemic area.	Y	Y
TRAVEL – Chagas' exposure	To identify donors who were born in a Chagas-endemic country, and hence are suitable only for plasma derivative production.	What was your country of birth?		Y	N

**Key evaluative topic for donor eligibility**	**Intent of question**	**Core sample question**	**Optional sample question**	**First-time & repeat donors**	**Regular donors**
TRAVEL – vCJD exposure	The core geographical risk of variant Creutzfeldt-Jakob disease (vCJD) has been defined as extending from 1980 to 1996 in the United Kingdom. In each individual blood establishment, risk assessment should define the appropriate cumulative period and whether additional countries should be added to the risk zone.	From 1980 to 1996 inclusive, did you spend 6 months or more (cumulative) in the UK?		Y	N
OTHER BLOOD-BORNE RISKS – hepatitis	To identify donors with occupational or household exposure to hepatitis, and trigger appropriate clearance/immunity testing.	Have you been exposed to hepatitis or jaundice(via family, household or occupation) in the past 6 months?		Y	Y
OTHER BLOOD-BORNE RISKS – flexible endoscopy	Some countries have reported an association between procedures employing flexible endoscopy and hepatitis C infection.		Have you had an endoscopy or gastroscopy in the last 4 months?<br>If so, was a flexible instrument used and was any biopsy performed?	Y	Y

Key evaluative topic for donor eligibility	Intent of question	Core sample question	Optional sample question	First-time & repeat donors	Regular donors
OTHER BLOOD-BORNE RISKS – dental	Tooth extraction and other dental procedures can be associated with transient bacteraemia, which can theoretically cause bacterial contamination of fresh blood components.	Have you had any dental treatment in the last week?		Y	Y
OTHER BLOOD-BORNE RISKS – invasive procedures	Invasive procedures can be a source of blood-borne infection. The donor may require temporary deferral to exclude window period transmission of infectious disease.	Since your last donation or in the previous 6 months have you had:<br>• an operation or medical investigations?<br>• any body piercing and/or tattoo?<br>• acupuncture treatment by anyone other than a registered practitioner?<br>• an accidental injury involving a needle and/or mucous membrane exposure to human blood?		Y	Y
OTHER BLOOD-BORNE RISKS – familial CJD	Classical CJD (CJD) may potentially be transmitted by blood transfusion.	Have you been told of a family history of Creutzfeldt-Jakob disease (CJD)?		Y	Y

Key evaluative topic for donor eligibility	Intent of question	Core sample question	Optional sample question	First-time & repeat donors	Regular donors
OTHER BLOOD-BORNE RISKS – pituitary extracts	Most reported cases of iatrogenic CJD have been associated with human-derived pituitary hormone treatment.	Have you ever had treatment with human pituitary extracts?		Y	N
OTHER BLOOD-BORNE RISKS – transplantation	Transplantation may result in the transmission of a range of infectious diseases, and corneal transplantation and dura mater grafts have been reported as causes of iatrogenic CJD?	Have you ever had a transplant or graft (organ, bone marrow, cornea, dura mater, bone, etc)?		Y	Y
OTHER BLOOD-BORNE RISKS – cuts and abrasions	Broken or inflamed skin is a potential source of bacterial contamination. A rash may be a sign of underlying disease.		Do you have any cuts, abrasions or sores?	Y	Y
OTHER BLOOD-BORNE RISKS – gastrointestinal symptoms	Gastrointestinal symptoms could be associated with conditions which impact both recipient safety (e.g. Yersinia enterocolitica) and donor safety (e.g. hypokalaemia secondary to vomiting and diarrhoea).	In the past week, have you had any diarrhoea, abdominal pain or vomiting?		Y	Y

Key evaluative topic for donor eligibility	Intent of question	Core sample question	Optional sample question	First-time & repeat donors	Regular donors
OTHER BLOOD-BORNE RISKS – transfusion	Blood transfusion may cause transmission of blood-borne infections, including geographically restricted infections such as vCJD and Chagas' disease.	Have you ever received a blood transfusion or injection of blood products? If so, where and when?		Y	Y
OTHER BLOOD-BORNE RISKS – positive infectious disease testing	HIV, hepatitis B, hepatitis C and HTLV are transfusion-transmissible infectious agents, and all may be transmitted between partners by sexual or blood contact.	Are you or is your partner positive for HIV, hepatitis B, hepatitis C or HTLV?		Y	Y

APPENDIX 2.
TABLES FOR CALCULATION OF BLOOD VOLUMES

Table 1. Blood volume of women in mL as calculated according to the ICSH formula[1]

The weights and heights corresponding to the minimum acceptable blood volumes of 3 233 mL, 3 400 mL and 3 567 mL are indicated with grey backgrounds.

kg	50	51	52	53	54	55	56	57	58	59
**145 cm**	3 141	3 167	3 193	3 219	3 244	3 269	3 294	3 319	3 343	3 367
**146 cm**	3 157	3 183	3 209	3 235	3 260	3 285	3 310	3 335	3 359	3 384
**147 cm**	3 172	3 199	3 225	3 251	3 276	3 301	3 327	3 351	3 376	3 400
**148 cm**	3 187	3 214	3 240	3 266	3 292	3 318	3 343	3 368	3 392	3 417
**149 cm**	3 203	3 230	3 256	3 282	3 308	3 334	3 359	3 384	3 409	3 433
**150 cm**	3 218	3 245	3 272	3 298	3 324	3 350	3 375	3 400	3 425	3 450
**151 cm**	3 234	3 261	3 287	3 314	3 340	3 366	3 391	3 416	3 441	3 466
**152 cm**	3 249	3 276	3 303	3 329	3 356	3 381	3 407	3 433	3 458	3 483
**153 cm**	3 264	3 291	3 318	3 345	3 371	3 397	3 423	3 449	3 474	3 499
**154 cm**	3 279	3 307	3 334	3 361	3 387	3 413	3 439	3 465	3 490	3 515
**155 cm**	3 295	3 322	3 349	3 376	3 403	3 429	3 455	3 481	3 506	3 532
**156 cm**	3 310	3 337	3 365	3 392	3 418	3 445	3 471	3 497	3 523	3 548
**157 cm**	3 325	3 353	3 380	3 407	3 434	3 461	3 487	3 513	3 539	3 564
**158 cm**	3 340	3 368	3 396	3 423	3 450	3 476	3 503	3 529	3 555	3 581
**159 cm**	3 355	3 383	3 411	3 438	3 465	3 492	3 519	3 545	3 571	3 597
**160 cm**	3 370	3 399	3 426	3 454	3 481	3 508	3 535	3 561	3 587	3 613
**161 cm**	3 385	3 414	3 442	3 469	3 497	3 524	3 550	3 577	3 603	3 629
**162 cm**	3 400	3 429	3 457	3 485	3 512	3 539	3 566	3 593	3 619	3 645
**163 cm**	3 416	3 444	3 472	3 500	3 528	3 555	3 582	3 609	3 635	3 661
**164 cm**	3 430	3 459	3 487	3 515	3 543	3 571	3 598	3 625	3 651	3 677
**165 cm**	3 445	3 474	3 503	3 531	3 559	3 586	3 613	3 640	3 667	3 693
**166 cm**	3 460	3 489	3 518	3 546	3 574	3 602	3 629	3 656	3 683	3 709
**167 cm**	3 475	3 504	3 533	3 561	3 589	3 617	3 645	3 672	3 699	3 726
**168 cm**	3 490	3 519	3 548	3 577	3 605	3 633	3 660	3 688	3 715	3 741

1 Pearson TC, Guthrie DL, Simpson J, Chinn C, Barosi G, Ferrant A, Lewis SM, Najean Y; Interpretation of measured red cell mass and plasma volume in adults: Expert Panel on Radionuclides of the International Council for Standardisation in Haematology. Br. J. Haem. 1995, 89:748-56.

kg	50	51	52	53	54	55	56	57	58	59
**169 cm**	3 505	3 534	3 563	3 592	3 620	3 648	3 676	3 703	3 731	3 757
**170 cm**	3 520	3 549	3 578	3 607	3 636	3 664	3 692	3 719	3 746	3 773
**171 cm**	3 535	3 564	3 593	3 622	3 651	3 679	3 707	3 735	3 762	3 789
**172 cm**	3 550	3 579	3 608	3 637	3 666	3 695	3 723	3 750	3 778	3 805
**173 cm**	3 564	3 594	3 624	3 653	3 681	3 710	3 738	3 766	3 794	3 821
**174 cm**	3 579	3 609	3 638	3 668	3 697	3 725	3 754	3 782	3 809	3 837
**175 cm**	3 594	3 624	3 653	3 683	3 712	3 741	3 769	3 797	3 825	3 853
**176 cm**	3 608	3 639	3 668	3 698	3 727	3 756	3 784	3 813	3 841	3 868
**177 cm**	3 623	3 653	3 683	3 713	3 742	3 771	3 800	3 828	3 856	3 884
**178 cm**	3 638	3 668	3 698	3 728	3 757	3 786	3 815	3 844	3 872	3 900
**179 cm**	3 652	3 683	3 713	3 743	3 772	3 802	3 831	3 859	3 887	3 916
**180 cm**	3 667	3 698	3 728	3 758	3 788	3 817	3 846	3 875	3 903	3 931
**181 cm**	3 682	3 712	3 743	3 773	3 803	3 832	3 861	3 890	3 919	3 947
**182 cm**	3 696	3 727	3 758	3 788	3 818	3 847	3 877	3 905	3 934	3 962
**183 cm**	3 711	3 742	3 772	3 803	3 833	3 862	3 892	3 921	3 950	3 978
**184 cm**	3 725	3 756	3 787	3 818	3 848	3 878	3 907	3 936	3 965	3 994
**185 cm**	3 740	3 771	3 802	3 832	3 863	3 893	3 922	3 952	3 981	4 009

kg	60	61	62	63	64	65	66	67	68	69
**145 cm**	3 391	3 414	3 438	3 461	3 484	3 507	3 529	3 552	3 574	3 596
**146 cm**	3 408	3 431	3 455	3 478	3 501	3 524	3 547	3 569	3 591	3 613
**147 cm**	3 424	3 448	3 472	3 495	3 518	3 541	3 564	3 587	3 609	3 631
**148 cm**	3 441	3 465	3 489	3 512	3 535	3 558	3 581	3 604	3 627	3 649
**149 cm**	3 458	3 482	3 505	3 529	3 552	3 576	3 599	3 622	3 644	3 667
**150 cm**	3 474	3 498	3 522	3 546	3 570	3 593	3 616	3 639	3 662	3 684
**151 cm**	3 491	3 515	3 539	3 563	3 587	3 610	3 633	3 656	3 679	3 702
**152 cm**	3 507	3 532	3 556	3 580	3 604	3 627	3 650	3 674	3 697	3 719
**153 cm**	3 524	3 548	3 573	3 597	3 621	3 644	3 668	3 691	3 714	3 737
**154 cm**	3 540	3 565	3 589	3 614	3 638	3 661	3 685	3 708	3 731	3 754
**155 cm**	3 557	3 581	3 606	3 630	3 654	3 678	3 702	3 725	3 749	3 772
**156 cm**	3 573	3 598	3 623	3 647	3 671	3 695	3 719	3 743	3 766	3 789
**157 cm**	3 590	3 615	3 639	3 664	3 688	3 712	3 736	3 760	3 783	3 807
**158 cm**	3 606	3 631	3 656	3 681	3 705	3 729	3 753	3 777	3 801	3 824

kg	60	61	62	63	64	65	66	67	68	69
**159 cm**	3 622	3 647	3 672	3 697	3 722	3 746	3 770	3 794	3 818	3 841
**160 cm**	3 639	3 664	3 689	3 714	3 739	3 763	3 787	3 811	3 835	3 859
**161 cm**	3 655	3 680	3 705	3 730	3 755	3 780	3 804	3 828	3 852	3 876
**162 cm**	3 671	3 697	3 722	3 747	3 772	3 797	3 821	3 845	3 869	3 893
**163 cm**	3 687	3 713	3 738	3 764	3 789	3 813	3 838	3 862	3 886	3 910
**164 cm**	3 703	3 729	3 755	3 780	3 805	3 830	3 855	3 879	3 903	3 928
**165 cm**	3 720	3 746	3 771	3 797	3 822	3 847	3 872	3 896	3 921	3 945
**166 cm**	3 736	3 762	3 788	3 813	3 838	3 864	3 888	3 913	3 938	3 962
**167 cm**	3 752	3 778	3 804	3 830	3 855	3 880	3 905	3 930	3 955	3 979
**168 cm**	3 768	3 794	3 820	3 846	3 872	3 897	3 922	3 947	3 972	3 996
**169 cm**	3 784	3 810	3 837	3 862	3 888	3 914	3 939	3 964	3 988	4 013
**170 cm**	3 800	3 827	3 853	3 879	3 905	3 930	3 955	3 981	4 005	4 030
**171 cm**	3 816	3 843	3 869	3 895	3 921	3 947	3 972	3 997	4 022	4 047
**172 cm**	3 832	3 859	3 885	3 911	3 937	3 963	3 989	4 014	4 039	4 064
**173 cm**	3 848	3 875	3 901	3 928	3 954	3 980	4 005	4 031	4 056	4 081
**174 cm**	3 864	3 891	3 918	3 944	3 970	3 996	4 022	4 047	4 073	4 098
**175 cm**	3 880	3 907	3 934	3 960	3 987	4 013	4 039	4 064	4 090	4 115
**176 cm**	3 896	3 923	3 950	3 977	4 003	4 029	4 055	4 081	4 106	4 132
**177 cm**	3 912	3 939	3 966	3 993	4 019	4 046	4 072	4 097	4 123	4 148
**178 cm**	3 927	3 955	3 982	4 009	4 036	4 062	4 088	4 114	4 140	4 165
**179 cm**	3 943	3 971	3 998	4 025	4 052	4 078	4 105	4 131	4 156	4 182
**180 cm**	3 959	3 987	4 014	4 041	4 068	4 095	4 121	4 147	4 173	4 199
**181 cm**	3 975	4 003	4 030	4 057	4 084	4 111	4 137	4 164	4 190	4 216
**182 cm**	3 991	4 018	4 046	4 073	4 100	4 127	4 154	4 180	4 206	4 232
**183 cm**	4 006	4 034	4 062	4 089	4 117	4 143	4 170	4 197	4 223	4 249
**184 cm**	4 022	4 050	4 078	4 105	4 133	4 160	4 187	4 213	4 239	4 266
**185 cm**	4 038	4 066	4 094	4 121	4 149	4 176	4 203	4 229	4 256	4 282

kg	70	71	72	73	74	75	76	77	78	79
**145 cm**	3 618	3 639	3 661	3 682	3 703	3 724	3 745	3 765	3 786	3 806
**146 cm**	3 635	3 657	3 679	3 700	3 721	3 742	3 763	3 784	3 804	3 825
**147 cm**	3 653	3 675	3 697	3 718	3 739	3 761	3 782	3 802	3 823	3 844
**148 cm**	3 671	3 693	3 715	3 736	3 758	3 779	3 800	3 821	3 842	3 862
**149 cm**	3 689	3 711	3 733	3 754	3 776	3 797	3 818	3 839	3 860	3 881

kg	70	71	72	73	74	75	76	77	78	79
**150 cm**	3706	3729	3751	3772	3794	3816	3837	3858	3879	3900
**151 cm**	3724	3746	3769	3790	3812	3834	3855	3876	3897	3918
**152 cm**	3742	3764	3786	3808	3830	3852	3873	3895	3916	3937
**153 cm**	3759	3782	3804	3826	3848	3870	3892	3913	3934	3956
**154 cm**	3777	3800	3822	3844	3866	3888	3910	3931	3953	3974
**155 cm**	3795	3817	3840	3862	3884	3906	3928	3950	3971	3993
**156 cm**	3812	3835	3858	3880	3902	3924	3946	3968	3990	4011
**157 cm**	3830	3853	3875	3898	3920	3942	3964	3986	4008	4029
**158 cm**	3847	3870	3893	3916	3938	3960	3982	4004	4026	4048
**159 cm**	3865	3888	3911	3933	3956	3978	4001	4023	4044	4066
**160 cm**	3882	3905	3928	3951	3974	3996	4019	4041	4063	4085
**161 cm**	3899	3923	3946	3969	3992	4014	4037	4059	4081	4103
**162 cm**	3917	3940	3963	3986	4009	4032	4055	4077	4099	4121
**163 cm**	3934	3958	3981	4004	4027	4050	4072	4095	4117	4139
**164 cm**	3951	3975	3998	4022	4045	4068	4090	4113	4135	4158
**165 cm**	3969	3992	4016	4039	4062	4085	4108	4131	4153	4176
**166 cm**	3986	4010	4033	4057	4080	4103	4126	4149	4171	4194
**167 cm**	4003	4027	4051	4074	4098	4121	4144	4167	4189	4212
**168 cm**	4020	4044	4068	4092	4115	4139	4162	4185	4207	4230
**169 cm**	4037	4062	4086	4109	4133	4156	4179	4203	4225	4248
**170 cm**	4055	4079	4103	4127	4150	4174	4197	4220	4243	4266
**171 cm**	4072	4096	4120	4144	4168	4192	4215	4238	4261	4284
**172 cm**	4089	4113	4137	4162	4185	4209	4233	4256	4279	4302
**173 cm**	4106	4130	4155	4179	4203	4227	4250	4274	4297	4320
**174 cm**	4123	4147	4172	4196	4220	4244	4268	4291	4315	4338
**175 cm**	4140	4165	4189	4213	4238	4262	4285	4309	4333	4356
**176 cm**	4157	4182	4206	4231	4255	4279	4303	4327	4350	4374
**177 cm**	4174	4199	4223	4248	4272	4297	4321	4344	4368	4392
**178 cm**	4191	4216	4241	4265	4290	4314	4338	4362	4386	4409
**179 cm**	4207	4233	4258	4282	4307	4331	4356	4380	4403	4427
**180 cm**	4224	4250	4275	4300	4324	4349	4373	4397	4421	4445
**181 cm**	4241	4266	4292	4317	4341	4366	4390	4415	4439	4463
**182 cm**	4258	4283	4309	4334	4359	4383	4408	4432	4456	4480
**183 cm**	4275	4300	4326	4351	4376	4401	4425	4450	4474	4498

kg	70	71	72	73	74	75	76	77	78	79
**184 cm**	4 291	4 317	4 343	4 368	4 393	4 418	4 443	4 467	4 491	4 516
**185 cm**	4 308	4 334	4 360	4 385	4 410	4 435	4 460	4 485	4 509	4 533

kg	80	81	82	83	84	85	86	87	88	89	90
**145 cm**	3 826	3 846	3 866	3 886	3 906	3 925	3 944	3 964	3 983	4 002	4 021
**146 cm**	3 845	3 865	3 885	3 905	3 925	3 944	3 964	3 983	4 002	4 021	4 040
**147 cm**	3 864	3 884	3 904	3 924	3 944	3 964	3 983	4 003	4 022	4 041	4 060
**148 cm**	3 883	3 903	3 923	3 943	3 963	3 983	4 003	4 022	4 042	4 061	4 080
**149 cm**	3 902	3 922	3 942	3 963	3 983	4 002	4 022	4 042	4 061	4 081	4 100
**150 cm**	3 920	3 941	3 961	3 982	4 002	4 022	4 041	4 061	4 081	4 100	4 119
**151 cm**	3 939	3 960	3 980	4 001	4 021	4 041	4 061	4 081	4 100	4 120	4 139
**152 cm**	3 958	3 979	3 999	4 020	4 040	4 060	4 080	4 100	4 120	4 139	4 159
**153 cm**	3 977	3 997	4 018	4 039	4 059	4 079	4 099	4 119	4 139	4 159	4 178
**154 cm**	3 995	4 016	4 037	4 057	4 078	4 098	4 119	4 139	4 159	4 178	4 198
**155 cm**	4 014	4 035	4 056	4 076	4 097	4 117	4 138	4 158	4 178	4 198	4 218
**156 cm**	4 032	4 053	4 074	4 095	4 116	4 136	4 157	4 177	4 197	4 217	4 237
**157 cm**	4 051	4 072	4 093	4 114	4 135	4 155	4 176	4 196	4 217	4 237	4 257
**158 cm**	4 069	4 091	4 112	4 133	4 154	4 174	4 195	4 215	4 236	4 256	4 276
**159 cm**	4 088	4 109	4 130	4 152	4 173	4 193	4 214	4 235	4 255	4 275	4 295
**160 cm**	4 106	4 128	4 149	4 170	4 191	4 212	4 233	4 254	4 274	4 295	4 315
**161 cm**	4 125	4 146	4 168	4 189	4 210	4 231	4 252	4 273	4 293	4 314	4 334
**162 cm**	4 143	4 165	4 186	4 208	4 229	4 250	4 271	4 292	4 312	4 333	4 353
**163 cm**	4 161	4 183	4 205	4 226	4 248	4 269	4 290	4 311	4 332	4 352	4 373
**164 cm**	4 180	4 202	4 223	4 245	4 266	4 288	4 309	4 330	4 351	4 371	4 392
**165 cm**	4 198	4 220	4 242	4 263	4 285	4 306	4 328	4 349	4 370	4 390	4 411
**166 cm**	4 216	4 238	4 260	4 282	4 304	4 325	4 346	4 368	4 389	4 410	4 430
**167 cm**	4 234	4 257	4 279	4 300	4 322	4 344	4 365	4 386	4 408	4 429	4 450
**168 cm**	4 253	4 275	4 297	4 319	4 341	4 362	4 384	4 405	4 427	4 448	4 469
**169 cm**	4 271	4 293	4 315	4 337	4 359	4 381	4 403	4 424	4 445	4 467	4 488
**170 cm**	4 289	4 311	4 334	4 356	4 378	4 400	4 421	4 443	4 464	4 486	4 507
**171 cm**	4 307	4 329	4 352	4 374	4 396	4 418	4 440	4 462	4 483	4 505	4 526
**172 cm**	4 325	4 348	4 370	4 392	4 415	4 437	4 459	4 480	4 502	4 523	4 545
**173 cm**	4 343	4 366	4 388	4 411	4 433	4 455	4 477	4 499	4 521	4 542	4 564
**174 cm**	4 361	4 384	4 407	4 429	4 451	4 474	4 496	4 518	4 540	4 561	4 583

kg	80	81	82	83	84	85	86	87	88	89	90
**175 cm**	4379	4402	4425	4447	4470	4492	4514	4536	4558	4580	4602
**176 cm**	4397	4420	4443	4466	4488	4511	4533	4555	4577	4599	4620
**177 cm**	4415	4438	4461	4484	4506	4529	4551	4574	4596	4618	4639
**178 cm**	4433	4456	4479	4502	4525	4547	4570	4592	4614	4636	4658
**179 cm**	4451	4474	4497	4520	4543	4566	4588	4611	4633	4655	4677
**180 cm**	4468	4492	4515	4538	4561	4584	4607	4629	4651	4674	4696
**181 cm**	4486	4510	4533	4556	4579	4602	4625	4648	4670	4692	4714
**182 cm**	4504	4528	4551	4574	4598	4621	4643	4666	4689	4711	4733
**183 cm**	4522	4546	4569	4592	4616	4639	4662	4684	4707	4729	4752
**184 cm**	4540	4563	4587	4610	4634	4657	4680	4703	4725	4748	4770
**185 cm**	4557	4581	4605	4628	4652	4675	4698	4721	4744	4767	4789

Table 2. Blood volume of men in mL as calculated according to the ICSH formula

kg	50	51	52	53	54	55	56	57	58	59
**160 cm**	3774	3813	3852	3890	3927	3965	4001	4038	4074	4110
**161 cm**	3795	3834	3873	3911	3949	3986	4023	4060	4096	4132
**162 cm**	3816	3855	3894	3932	3970	4008	4045	4082	4118	4154
**163 cm**	3837	3876	3915	3954	3992	4030	4067	4104	4140	4177
**164 cm**	3858	3897	3936	3975	4013	4051	4089	4126	4162	4199
**165 cm**	3878	3918	3957	3996	4035	4073	4110	4148	4184	4221
**166 cm**	3899	3939	3978	4017	4056	4094	4132	4169	4206	4243
**167 cm**	3919	3960	3999	4038	4077	4116	4154	4191	4228	4265
**168 cm**	3940	3980	4020	4060	4098	4137	4175	4213	4250	4287
**169 cm**	3961	4001	4041	4081	4120	4158	4197	4235	4272	4309
**170 cm**	3981	4022	4062	4102	4141	4180	4218	4256	4294	4331
**171 cm**	4002	4042	4083	4123	4162	4201	4240	4278	4316	4353
**172 cm**	4022	4063	4103	4144	4183	4222	4261	4300	4338	4375
**173 cm**	4042	4084	4124	4164	4204	4244	4283	4321	4359	4397
**174 cm**	4063	4104	4145	4185	4225	4265	4304	4343	4381	4419
**175 cm**	4083	4125	4166	4206	4246	4286	4325	4364	4403	4441
**176 cm**	4103	4145	4186	4227	4267	4307	4347	4386	4424	4463

kg	50	51	52	53	54	55	56	57	58	59
**177 cm**	4124	4166	4207	4248	4288	4328	4368	4407	4446	4484
**178 cm**	4144	4186	4228	4269	4309	4349	4389	4429	4468	4506
**179 cm**	4164	4206	4248	4289	4330	4371	4410	4450	4489	4528
**180 cm**	4184	4227	4269	4310	4351	4392	4432	4471	4511	4550
**181 cm**	4205	4247	4289	4331	4372	4413	4453	4493	4532	4571
**182 cm**	4225	4267	4310	4351	4393	4433	4474	4514	4554	4593
**183 cm**	4245	4288	4330	4372	4413	4454	4495	4535	4575	4614
**184 cm**	4265	4308	4350	4393	4434	4475	4516	4556	4596	4636
**185 cm**	4285	4328	4371	4413	4455	4496	4537	4578	4618	4657
**186 cm**	4305	4348	4391	4434	4476	4517	4558	4599	4639	4679
**187 cm**	4325	4368	4412	4454	4496	4538	4579	4620	4660	4700
**188 cm**	4345	4389	4432	4475	4517	4559	4600	4641	4682	4722
**189 cm**	4365	4409	4452	4495	4537	4579	4621	4662	4703	4743
**190 cm**	4385	4429	4472	4515	4558	4600	4642	4683	4724	4764
**191 cm**	4405	4449	4492	4536	4578	4621	4663	4704	4745	4786
**192 cm**	4424	4469	4513	4556	4599	4641	4683	4725	4766	4807
**193 cm**	4444	4489	4533	4576	4619	4662	4704	4746	4787	4828
**194 cm**	4464	4509	4553	4597	4640	4683	4725	4767	4808	4849
**195 cm**	4484	4529	4573	4617	4660	4703	4746	4788	4829	4871
**196 cm**	4503	4549	4593	4637	4681	4724	4766	4809	4850	4892
**197 cm**	4523	4568	4613	4657	4701	4744	4787	4829	4871	4913
**198 cm**	4543	4588	4633	4677	4721	4765	4808	4850	4892	4934
**199 cm**	4562	4608	4653	4698	4742	4785	4828	4871	4913	4955
**200 cm**	4582	4628	4673	4718	4762	4806	4849	4892	4934	4976

kg	60	61	62	63	64	65	66	67	68	69
**160 cm**	4145	4180	4215	4249	4283	4317	4350	4384	4417	4449
**161 cm**	4168	4203	4238	4272	4306	4340	4374	4407	4440	4473
**162 cm**	4190	4225	4260	4295	4329	4363	4397	4431	4464	4497
**163 cm**	4212	4248	4283	4318	4352	4387	4421	4454	4488	4521
**164 cm**	4235	4270	4306	4341	4375	4410	4444	4478	4511	4544
**165 cm**	4257	4293	4328	4364	4398	4433	4467	4501	4535	4568

kg	60	61	62	63	64	65	66	67	68	69
**166 cm**	4279	4315	4351	4386	4421	4456	4490	4525	4558	4592
**167 cm**	4302	4338	4374	4409	4444	4479	4514	4548	4582	4615
**168 cm**	4324	4360	4396	4432	4467	4502	4537	4571	4605	4639
**169 cm**	4346	4383	4419	4454	4490	4525	4560	4594	4629	4663
**170 cm**	4368	4405	4441	4477	4513	4548	4583	4618	4652	4686
**171 cm**	4390	4427	4464	4500	4535	4571	4606	4641	4675	4710
**172 cm**	4413	4449	4486	4522	4558	4594	4629	4664	4699	4733
**173 cm**	4435	4472	4508	4545	4581	4617	4652	4687	4722	4756
**174 cm**	4457	4494	4531	4567	4603	4639	4675	4710	4745	4780
**175 cm**	4479	4516	4553	4590	4626	4662	4698	4733	4768	4803
**176 cm**	4501	4538	4575	4612	4649	4685	4721	4756	4792	4826
**177 cm**	4522	4560	4598	4635	4671	4708	4744	4779	4815	4850
**178 cm**	4544	4582	4620	4657	4694	4730	4766	4802	4838	4873
**179 cm**	4566	4604	4642	4679	4716	4753	4789	4825	4861	4896
**180 cm**	4588	4626	4664	4701	4739	4775	4812	4848	4884	4919
**181 cm**	4610	4648	4686	4724	4761	4798	4835	4871	4907	4942
**182 cm**	4632	4670	4708	4746	4783	4820	4857	4894	4930	4966
**183 cm**	4653	4692	4730	4768	4806	4843	4880	4916	4953	4989
**184 cm**	4675	4714	4752	4790	4828	4865	4902	4939	4975	5012
**185 cm**	4697	4736	4774	4812	4850	4888	4925	4962	4998	5035
**186 cm**	4718	4757	4796	4834	4872	4910	4947	4984	5021	5058
**187 cm**	4740	4779	4818	4856	4895	4932	4970	5007	5044	5080
**188 cm**	4761	4801	4840	4878	4917	4955	4992	5030	5067	5103
**189 cm**	4783	4822	4862	4900	4939	4977	5015	5052	5089	5126
**190 cm**	4804	4844	4883	4922	4961	4999	5037	5075	5112	5149
**191 cm**	4826	4866	4905	4944	4983	5021	5060	5097	5135	5172
**192 cm**	4847	4887	4927	4966	5005	5044	5082	5120	5157	5194
**193 cm**	4869	4909	4949	4988	5027	5066	5104	5142	5180	5217
**194 cm**	4890	4930	4970	5010	5049	5088	5126	5165	5202	5240
**195 cm**	4911	4952	4992	5032	5071	5110	5149	5187	5225	5263
**196 cm**	4933	4973	5014	5053	5093	5132	5171	5209	5247	5285
**197 cm**	4954	4995	5035	5075	5115	5154	5193	5232	5270	5308
**198 cm**	4975	5016	5057	5097	5137	5176	5215	5254	5292	5330
**199 cm**	4997	5038	5078	5119	5158	5198	5237	5276	5315	5353

kg	60	61	62	63	64	65	66	67	68	69
**200 cm**	5018	5059	5100	5140	5180	5220	5259	5298	5337	5375

kg	70	71	72	73	74	75	76	77	78	79
**160 cm**	4482	4514	4545	4577	4608	4639	4670	4701	4731	4761
**161 cm**	4506	4538	4570	4601	4633	4664	4695	4726	4756	4787
**162 cm**	4530	4562	4594	4626	4657	4689	4720	4751	4782	4812
**163 cm**	4553	4586	4618	4650	4682	4713	4745	4776	4807	4837
**164 cm**	4577	4610	4642	4675	4706	4738	4770	4801	4832	4862
**165 cm**	4601	4634	4667	4699	4731	4763	4794	4826	4857	4887
**166 cm**	4625	4658	4691	4723	4755	4787	4819	4850	4882	4913
**167 cm**	4649	4682	4715	4747	4780	4812	4844	4875	4906	4938
**168 cm**	4673	4706	4739	4772	4804	4836	4868	4900	4931	4963
**169 cm**	4696	4730	4763	4796	4828	4861	4893	4925	4956	4988
**170 cm**	4720	4753	4787	4820	4852	4885	4917	4949	4981	5012
**171 cm**	4744	4777	4811	4844	4877	4909	4942	4974	5006	5037
**172 cm**	4767	4801	4835	4868	4901	4934	4966	4998	5030	5062
**173 cm**	4791	4825	4858	4892	4925	4958	4990	5023	5055	5087
**174 cm**	4814	4848	4882	4916	4949	4982	5015	5047	5080	5112
**175 cm**	4838	4872	4906	4940	4973	5006	5039	5072	5104	5136
**176 cm**	4861	4896	4930	4963	4997	5030	5063	5096	5129	5161
**177 cm**	4885	4919	4953	4987	5021	5054	5088	5121	5153	5186
**178 cm**	4908	4943	4977	5011	5045	5079	5112	5145	5178	5210
**179 cm**	4931	4966	5001	5035	5069	5103	5136	5169	5202	5235
**180 cm**	4955	4990	5024	5059	5093	5127	5160	5193	5227	5259
**181 cm**	4978	5013	5048	5082	5116	5150	5184	5218	5251	5284
**182 cm**	5001	5036	5071	5106	5140	5174	5208	5242	5275	5308
**183 cm**	5024	5060	5095	5129	5164	5198	5232	5266	5300	5333
**184 cm**	5047	5083	5118	5153	5188	5222	5256	5290	5324	5357
**185 cm**	5071	5106	5142	5177	5211	5246	5280	5314	5348	5381
**186 cm**	5094	5129	5165	5200	5235	5270	5304	5338	5372	5406
**187 cm**	5117	5153	5188	5224	5259	5293	5328	5362	5396	5430
**188 cm**	5140	5176	5212	5247	5282	5317	5352	5386	5420	5454
**189 cm**	5163	5199	5235	5270	5306	5341	5376	5410	5444	5478
**190 cm**	5186	5222	5258	5294	5329	5364	5399	5434	5468	5503

kg	70	71	72	73	74	75	76	77	78	79
**191 cm**	5 209	5 245	5 281	5 317	5 353	5 388	5 423	5 458	5 492	5 527
**192 cm**	5 231	5 268	5 304	5 340	5 376	5 412	5 447	5 482	5 516	5 551
**193 cm**	5 254	5 291	5 327	5 364	5 400	5 435	5 470	5 506	5 540	5 575
**194 cm**	5 277	5 314	5 351	5 387	5 423	5 459	5 494	5 529	5 564	5 599
**195 cm**	5 300	5 337	5 374	5 410	5 446	5 482	5 518	5 553	5 588	5 623
**196 cm**	5 323	5 360	5 397	5 433	5 470	5 506	5 541	5 577	5 612	5 647
**197 cm**	5 345	5 383	5 420	5 456	5 493	5 529	5 565	5 600	5 636	5 671
**198 cm**	5 368	5 405	5 443	5 479	5 516	5 552	5 588	5 624	5 660	5 695
**199 cm**	5 391	5 428	5 466	5 503	5 539	5 576	5 612	5 648	5 683	5 719
**200 cm**	5 413	5 451	5 488	5 526	5 562	5 599	5 635	5 671	5 707	5 742

kg	80	81	82	83	84	85	86	87	88	89
**160 cm**	4 791	4 821	4 851	4 880	4 909	4 938	4 967	4 995	5 024	5 052
**161 cm**	4 817	4 847	4 876	4 906	4 935	4 964	4 993	5 022	5 050	5 078
**162 cm**	4 842	4 872	4 902	4 932	4 961	4 990	5 019	5 048	5 076	5 105
**163 cm**	4 868	4 898	4 928	4 957	4 987	5 016	5 045	5 074	5 103	5 131
**164 cm**	4 893	4 923	4 953	4 983	5 013	5 042	5 071	5 100	5 129	5 158
**165 cm**	4 918	4 948	4 979	5 009	5 038	5 068	5 097	5 127	5 155	5 184
**166 cm**	4 943	4 974	5 004	5 034	5 064	5 094	5 123	5 153	5 182	5 211
**167 cm**	4 968	4 999	5 030	5 060	5 090	5 120	5 149	5 179	5 208	5 237
**168 cm**	4 994	5 024	5 055	5 085	5 116	5 145	5 175	5 205	5 234	5 263
**169 cm**	5 019	5 050	5 080	5 111	5 141	5 171	5 201	5 231	5 260	5 290
**170 cm**	5 044	5 075	5 106	5 136	5 167	5 197	5 227	5 257	5 286	5 316
**171 cm**	5 069	5 100	5 131	5 162	5 192	5 223	5 253	5 283	5 312	5 342
**172 cm**	5 094	5 125	5 156	5 187	5 218	5 248	5 278	5 309	5 338	5 368
**173 cm**	5 119	5 150	5 181	5 212	5 243	5 274	5 304	5 334	5 364	5 394
**174 cm**	5 144	5 175	5 206	5 238	5 269	5 299	5 330	5 360	5 390	5 420
**175 cm**	5 168	5 200	5 232	5 263	5 294	5 325	5 355	5 386	5 416	5 446
**176 cm**	5 193	5 225	5 257	5 288	5 319	5 350	5 381	5 412	5 442	5 472
**177 cm**	5 218	5 250	5 282	5 313	5 345	5 376	5 407	5 437	5 468	5 498
**178 cm**	5 243	5 275	5 307	5 338	5 370	5 401	5 432	5 463	5 494	5 524
**179 cm**	5 267	5 300	5 332	5 363	5 395	5 426	5 458	5 488	5 519	5 550
**180 cm**	5 292	5 324	5 357	5 388	5 420	5 452	5 483	5 514	5 545	5 576
**181 cm**	5 317	5 349	5 381	5 414	5 445	5 477	5 508	5 540	5 571	5 601

kg	80	81	82	83	84	85	86	87	88	89
**182 cm**	5 341	5 374	5 406	5 438	5 470	5 502	5 534	5 565	5 596	5 627
**183 cm**	5 366	5 399	5 431	5 463	5 495	5 527	5 559	5 590	5 622	5 653
**184 cm**	5 390	5 423	5 456	5 488	5 521	5 553	5 584	5 616	5 647	5 678
**185 cm**	5 415	5 448	5 481	5 513	5 545	5 578	5 610	5 641	5 673	5 704
**186 cm**	5 439	5 472	5 505	5 538	5 570	5 603	5 635	5 667	5 698	5 730
**187 cm**	5 464	5 497	5 530	5 563	5 595	5 628	5 660	5 692	5 724	5 755
**188 cm**	5 488	5 521	5 555	5 588	5 620	5 653	5 685	5 717	5 749	5 781
**189 cm**	5 512	5 546	5 579	5 612	5 645	5 678	5 710	5 742	5 774	5 806
**190 cm**	5 537	5 570	5 604	5 637	5 670	5 703	5 735	5 767	5 800	5 831
**191 cm**	5 561	5 595	5 628	5 662	5 695	5 728	5 760	5 793	5 825	5 857
**192 cm**	5 585	5 619	5 653	5 686	5 719	5 752	5 785	5 818	5 850	5 882
**193 cm**	5 609	5 643	5 677	5 711	5 744	5 777	5 810	5 843	5 875	5 907
**194 cm**	5 633	5 668	5 702	5 735	5 769	5 802	5 835	5 868	5 900	5 933
**195 cm**	5 658	5 692	5 726	5 760	5 793	5 827	5 860	5 893	5 925	5 958
**196 cm**	5 682	5 716	5 750	5 784	5 818	5 851	5 885	5 918	5 951	5 983
**197 cm**	5 706	5 740	5 775	5 809	5 842	5 876	5 909	5 943	5 976	6 008
**198 cm**	5 730	5 764	5 799	5 833	5 867	5 901	5 934	5 968	6 001	6 033
**199 cm**	5 754	5 788	5 823	5 857	5 891	5 925	5 959	5 992	6 026	6 059
**200 cm**	5 778	5 813	5 847	5 882	5 916	5 950	5 984	6 017	6 051	6 084

kg	90	91	92	93	94	95	96	97	98	99
**160 cm**	5 080	5 107	5 135	5 163	5 190	5 217	5 244	5 271	5 297	5 324
**161 cm**	5 106	5 134	5 162	5 190	5 217	5 244	5 271	5 298	5 325	5 352
**162 cm**	5 133	5 161	5 189	5 217	5 244	5 272	5 299	5 326	5 353	5 379
**163 cm**	5 160	5 188	5 216	5 244	5 271	5 299	5 326	5 353	5 380	5 407
**164 cm**	5 186	5 215	5 243	5 271	5 298	5 326	5 353	5 381	5 408	5 435
**165 cm**	5 213	5 241	5 270	5 298	5 325	5 353	5 381	5 408	5 435	5 462
**166 cm**	5 239	5 268	5 296	5 324	5 353	5 380	5 408	5 436	5 463	5 490
**167 cm**	5 266	5 295	5 323	5 351	5 379	5 407	5 435	5 463	5 490	5 518
**168 cm**	5 292	5 321	5 350	5 378	5 406	5 434	5 462	5 490	5 518	5 545
**169 cm**	5 319	5 348	5 376	5 405	5 433	5 461	5 489	5 517	5 545	5 573
**170 cm**	5 345	5 374	5 403	5 432	5 460	5 488	5 517	5 545	5 572	5 600
**171 cm**	5 371	5 400	5 429	5 458	5 487	5 515	5 544	5 572	5 600	5 627
**172 cm**	5 398	5 427	5 456	5 485	5 514	5 542	5 571	5 599	5 627	5 655

kg	90	91	92	93	94	95	96	97	98	99
**173 cm**	5 424	5 453	5 482	5 511	5 540	5 569	5 597	5 626	5 654	5 682
**174 cm**	5 450	5 479	5 509	5 538	5 567	5 596	5 624	5 653	5 681	5 709
**175 cm**	5 476	5 506	5 535	5 564	5 594	5 622	5 651	5 680	5 708	5 736
**176 cm**	5 502	5 532	5 561	5 591	5 620	5 649	5 678	5 707	5 735	5 764
**177 cm**	5 528	5 558	5 588	5 617	5 647	5 676	5 705	5 734	5 762	5 791
**178 cm**	5 554	5 584	5 614	5 644	5 673	5 702	5 732	5 760	5 789	5 818
**179 cm**	5 580	5 610	5 640	5 670	5 700	5 729	5 758	5 787	5 816	5 845
**180 cm**	5 606	5 636	5 666	5 696	5 726	5 756	5 785	5 814	5 843	5 872
**181 cm**	5 632	5 662	5 693	5 723	5 752	5 782	5 811	5 841	5 870	5 899
**182 cm**	5 658	5 688	5 719	5 749	5 779	5 808	5 838	5 867	5 897	5 926
**183 cm**	5 684	5 714	5 745	5 775	5 805	5 835	5 865	5 894	5 923	5 953
**184 cm**	5 709	5 740	5 771	5 801	5 831	5 861	5 891	5 921	5 950	5 979
**185 cm**	5 735	5 766	5 797	5 827	5 857	5 888	5 917	5 947	5 977	6 006
**186 cm**	5 761	5 792	5 823	5 853	5 884	5 914	5 944	5 974	6 003	6 033
**187 cm**	5 786	5 818	5 848	5 879	5 910	5 940	5 970	6 000	6 030	6 060
**188 cm**	5 812	5 843	5 874	5 905	5 936	5 966	5 997	6 027	6 057	6 086
**189 cm**	5 838	5 869	5 900	5 931	5 962	5 992	6 023	6 053	6 083	6 113
**190 cm**	5 863	5 895	5 926	5 957	5 988	6 019	6 049	6 079	6 110	6 140
**191 cm**	5 889	5 920	5 952	5 983	6 014	6 045	6 075	6 106	6 136	6 166
**192 cm**	5 914	5 946	5 977	6 009	6 040	6 071	6 101	6 132	6 162	6 193
**193 cm**	5 940	5 971	6 003	6 034	6 066	6 097	6 128	6 158	6 189	6 219
**194 cm**	5 965	5 997	6 029	6 060	6 092	6 123	6 154	6 185	6 215	6 246
**195 cm**	5 990	6 022	6 054	6 086	6 117	6 149	6 180	6 211	6 241	6 272
**196 cm**	6 016	6 048	6 080	6 112	6 143	6 175	6 206	6 237	6 268	6 298
**197 cm**	6 041	6 073	6 105	6 137	6 169	6 200	6 232	6 263	6 294	6 325
**198 cm**	6 066	6 099	6 131	6 163	6 195	6 226	6 258	6 289	6 320	6 351
**199 cm**	6 091	6 124	6 156	6 188	6 220	6 252	6 284	6 315	6 346	6 377
**200 cm**	6 117	6 149	6 182	6 214	6 246	6 278	6 310	6 341	6 372	6 403

kg	100	101	102	103	104	105	106	107	108	109
**160 cm**	5 350	5 376	5 402	5 428	5 454	5 479	5 505	5 530	5 555	5 580
**161 cm**	5 378	5 404	5 430	5 456	5 482	5 508	5 534	5 559	5 584	5 609
**162 cm**	5 406	5 432	5 459	5 485	5 511	5 536	5 562	5 588	5 613	5 638
**163 cm**	5 434	5 460	5 487	5 513	5 539	5 565	5 591	5 616	5 642	5 667

kg	100	101	102	103	104	105	106	107	108	109
**164 cm**	5462	5488	5515	5541	5567	5593	5619	5645	5671	5696
**165 cm**	5489	5516	5543	5569	5596	5622	5648	5674	5699	5725
**166 cm**	5517	5544	5571	5597	5624	5650	5676	5702	5728	5754
**167 cm**	5545	5572	5599	5625	5652	5678	5704	5731	5757	5782
**168 cm**	5572	5600	5626	5653	5680	5706	5733	5759	5785	5811
**169 cm**	5600	5627	5654	5681	5708	5735	5761	5787	5814	5840
**170 cm**	5628	5655	5682	5709	5736	5763	5789	5816	5842	5868
**171 cm**	5655	5682	5710	5737	5764	5791	5818	5844	5870	5897
**172 cm**	5682	5710	5737	5765	5792	5819	5846	5872	5899	5925
**173 cm**	5710	5738	5765	5793	5820	5847	5874	5901	5927	5954
**174 cm**	5737	5765	5793	5820	5848	5875	5902	5929	5955	5982
**175 cm**	5765	5793	5820	5848	5875	5903	5930	5957	5984	6010
**176 cm**	5792	5820	5848	5876	5903	5930	5958	5985	6012	6039
**177 cm**	5819	5847	5875	5903	5931	5958	5986	6013	6040	6067
**178 cm**	5846	5875	5903	5931	5958	5986	6014	6041	6068	6095
**179 cm**	5873	5902	5930	5958	5986	6014	6041	6069	6096	6123
**180 cm**	5901	5929	5957	5986	6014	6041	6069	6097	6124	6151
**181 cm**	5928	5956	5985	6013	6041	6069	6097	6125	6152	6180
**182 cm**	5955	5983	6012	6040	6069	6097	6125	6152	6180	6208
**183 cm**	5982	6010	6039	6068	6096	6124	6152	6180	6208	6236
**184 cm**	6009	6038	6066	6095	6123	6152	6180	6208	6236	6263
**185 cm**	6035	6065	6093	6122	6151	6179	6207	6236	6264	6291
**186 cm**	6062	6092	6121	6149	6178	6207	6235	6263	6291	6319
**187 cm**	6089	6118	6148	6177	6205	6234	6263	6291	6319	6347
**188 cm**	6116	6145	6175	6204	6233	6261	6290	6318	6347	6375
**189 cm**	6143	6172	6202	6231	6260	6289	6317	6346	6374	6403
**190 cm**	6169	6199	6229	6258	6287	6316	6345	6373	6402	6430
**191 cm**	6196	6226	6255	6285	6314	6343	6372	6401	6430	6458
**192 cm**	6223	6253	6282	6312	6341	6370	6399	6428	6457	6486
**193 cm**	6249	6279	6309	6339	6368	6398	6427	6456	6485	6513
**194 cm**	6276	6306	6336	6366	6395	6425	6454	6483	6512	6541
**195 cm**	6302	6333	6363	6392	6422	6452	6481	6510	6539	6568
**196 cm**	6329	6359	6389	6419	6449	6479	6508	6538	6567	6596
**197 cm**	6355	6386	6416	6446	6476	6506	6535	6565	6594	6623

kg	100	101	102	103	104	105	106	107	108	109
**198 cm**	6382	6412	6443	6473	6503	6533	6562	6592	6621	6651
**199 cm**	6408	6439	6469	6500	6530	6560	6589	6619	6649	6678
**200 cm**	6434	6465	6496	6526	6556	6587	6616	6646	6676	6705

kg	110	111	112	113	114	115	116	117	118	119	120
**160 cm**	5605	5630	5655	5679	5704	5728	5752	5776	5800	5824	5848
**161 cm**	5634	5659	5684	5709	5733	5758	5782	5806	5830	5854	5878
**162 cm**	5664	5689	5713	5738	5763	5787	5812	5836	5860	5884	5908
**163 cm**	5693	5718	5743	5767	5792	5817	5841	5866	5890	5914	5938
**164 cm**	5721	5747	5772	5797	5822	5846	5871	5895	5920	5944	5968
**165 cm**	5750	5776	5801	5826	5851	5876	5901	5925	5950	5974	5998
**166 cm**	5779	5805	5830	5855	5880	5905	5930	5955	5979	6004	6028
**167 cm**	5808	5834	5859	5884	5910	5935	5960	5984	6009	6034	6058
**168 cm**	5837	5863	5888	5913	5939	5964	5989	6014	6039	6063	6088
**169 cm**	5866	5891	5917	5943	5968	5993	6018	6043	6068	6093	6118
**170 cm**	5894	5920	5946	5972	5997	6022	6048	6073	6098	6123	6147
**171 cm**	5923	5949	5975	6001	6026	6052	6077	6102	6127	6152	6177
**172 cm**	5951	5978	6004	6029	6055	6081	6106	6132	6157	6182	6207
**173 cm**	5980	6006	6032	6058	6084	6110	6135	6161	6186	6211	6236
**174 cm**	6009	6035	6061	6087	6113	6139	6165	6190	6215	6241	6266
**175 cm**	6037	6063	6090	6116	6142	6168	6194	6219	6245	6270	6295
**176 cm**	6065	6092	6118	6145	6171	6197	6223	6248	6274	6300	6325
**177 cm**	6094	6120	6147	6173	6200	6226	6252	6278	6303	6329	6354
**178 cm**	6122	6149	6175	6202	6228	6255	6281	6307	6332	6358	6384
**179 cm**	6150	6177	6204	6231	6257	6283	6310	6336	6362	6387	6413
**180 cm**	6179	6206	6232	6259	6286	6312	6338	6365	6391	6417	6442
**181 cm**	6207	6234	6261	6288	6314	6341	6367	6394	6420	6446	6472
**182 cm**	6235	6262	6289	6316	6343	6370	6396	6422	6449	6475	6501
**183 cm**	6263	6290	6317	6345	6371	6398	6425	6451	6478	6504	6530
**184 cm**	6291	6318	6346	6373	6400	6427	6454	6480	6507	6533	6559
**185 cm**	6319	6347	6374	6401	6428	6455	6482	6509	6535	6562	6588
**186 cm**	6347	6375	6402	6430	6457	6484	6511	6538	6564	6591	6617
**187 cm**	6375	6403	6430	6458	6485	6512	6539	6566	6593	6620	6646
**188 cm**	6403	6431	6458	6486	6513	6541	6568	6595	6622	6649	6675

kg	110	111	112	113	114	115	116	117	118	119	120
**189 cm**	6431	6459	6487	6514	6542	6569	6596	6624	6650	6677	6704
**190 cm**	6459	6487	6515	6542	6570	6597	6625	6652	6679	6706	6733
**191 cm**	6486	6515	6543	6570	6598	6626	6653	6681	6708	6735	6762
**192 cm**	6514	6542	6570	6598	6626	6654	6682	6709	6736	6763	6791
**193 cm**	6542	6570	6598	6626	6654	6682	6710	6737	6765	6792	6819
**194 cm**	6569	6598	6626	6654	6683	6710	6738	6766	6793	6821	6848
**195 cm**	6597	6626	6654	6682	6711	6739	6766	6794	6822	6849	6877
**196 cm**	6625	6653	6682	6710	6739	6767	6795	6823	6850	6878	6905
**197 cm**	6652	6681	6710	6738	6767	6795	6823	6851	6879	6906	6934
**198 cm**	6680	6709	6737	6766	6794	6823	6851	6879	6907	6935	6962
**199 cm**	6707	6736	6765	6794	6822	6851	6879	6907	6935	6963	6991
**200 cm**	6735	6764	6793	6821	6850	6879	6907	6935	6963	6991	7019

APPENDIX 3.
DATA PROCESSING SYSTEMS

1. Planning of a system

There are a variety of computer systems and software programs available and each has different functions. Prior to purchase, the user should:

- establish a list of requirements that will meet the needs of the user, including the duration of record keeping (in general 15 years in EU member states) and the duration of data keeping for traceability (30 years are required under EU Directive 2002/98/EC);
- evaluate the different computer systems and choose the one that meets the established requirements;
- audit the developer/manufacturer to ensure that they are able to provide a product that meets regulatory requirements;
- establish responsibility between the user and the developer/supplier/manufacturer to define roles and responsibilities with regard to testing, user instructions, maintenance, system improvements and access to source codes.

These steps ensure that the user has all the necessary information about the purchased system and has an established relationship with the developer. This course of action also minimises the need for 'workarounds' by the user, which can be a source of error.

2. Defining the system

The computer system of a blood establishment or hospital blood bank includes: hardware, software, peripheral devices and documentation (e.g. manuals and SOPs). To define the system, in co-operation with the vendor or developer, the user should generate a written description of the system, the functions that it is designed to perform and all human interactions. The documentation should be current, effectively updated, accurate and as detailed as necessary to ensure proper operation of the system. The documentation should include:

- a detailed specification (inventory) of the hardware, software and peripheral devices, including their environmental requirements and limitations;
- diagrams or flow charts of the system's operations that describe all component interfaces, a network diagram (if applicable) and all database structures, e.g. file sizes, input and output formats, etc.;
- SOPs that describe how the system is used. The user should develop the SOPs based on the instructions for use provided by the software developer and the internal procedures of the blood establishment or hospital blood bank. In particular, SOPs should address all manual and automated interactions with the system including:
  - routine back-up, maintenance and diagnostic procedures, including assignment of responsibilities;
  - 'workarounds' for system limitations;
  - procedures for handling errors, including assignment of responsibilities;
  - procedures for handling disasters and contingency planning, including assignment of responsibilities;
  - procedures for supervised changes to incorrect data;
  - procedures for validation of a change;
  - a training system including training manuals, documentation and procedures for training.

3. Implementation and validation

Provisions of the Good Practice Guidelines for blood establishments and hospital blood banks must be taken into account.

Validation documents and the results of tests performed and approved by the supplier/vendor/developer of the system should be presented to the user. The user can then perform tests according to a pre-defined and documented test plan. Types of risk to consider include:

inadequate design of the system, errors that may occur in use (user error or system defects), and loss/compromise of data. Testing should involve the entire system, and in the manner it is expected to perform in the blood facility. Testing may be performed by a third party but, in that case, must also include personnel from the blood facility. The following types of basic testing should be conducted:

Functional testing of components

The system components are presented with all types of expected interaction, including normal value, boundary, invalid and special case inputs. The system must produce the correct outputs, including error messages by control programs. It is useful to perform this testing in parallel with a reference or standard system.

Each test case should include the input, expected output, acceptance criteria and whether the test passed or failed. For traceability purposes and to facilitate quality assurance review and follow-up, it is recommended that any supporting documentation, such as print- screens, be included to verify the specific test case.

Data migration

The process for data migration should be defined, documented and appropriately tested. This should ensure full maintenance of traceability, including archiving of data where necessary.

Environmental testing

All qualification steps and results should be documented and approved before routine use of the system.

In the actual operating environment, functional tests are performed to demonstrate that:

- the software systems work properly with the hardware;
- all applications of the software perform properly with the operating system software;

- proper information passes correctly through system interfaces, including appropriate data transfer to or from other laboratory and automated (e.g. apheresis machine) systems, if applicable;
- accessories, such as barcode scanners, perform as expected with the blood establishment's barcode symbols;
- printed reports are appropriately and correctly formatted;
- personnel are trained and use the system correctly;
- the system performs properly at peak production times and with the maximum number of concurrent users;
- back-ups restore data in a correct way;
- if the system includes wireless radio frequency (RF) technology, it should be evaluated for electromagnetic compatibility (EMC) and electromagnetic interference (EMI) in the setting in which it is used.

Change control

In case of changes in the software, the validation status must be re-established. If a revalidation analysis is needed, it should be based on risk assessment and conducted not only for validation of the individual change, but also to determine the extent and impact of that change on the entire computerised system.

Maintenance of the system

The database should be checked periodically and systematically to identify and remove unwanted data such as duplicate records, and to ensure that data entries are accurate and stored correctly. Manual entry of critical data requires independent verification by a second authorised person.

Security of the database should be maintained by:

- an adequate change history of the system, including both software and hardware (when necessary);

- periodically altering electronic passwords (without reuse) and by removing unnecessary or outdated access;
- creating records of all data changes, i.e. an audit trail, including a retained record of the previous data and the reason for the change;
- the appropriate use of programs to detect and remove computer viruses;
- the control of administrative security access to ensure that only authorised personnel can make changes to the software, to the system configuration and to the data;
- regular testing to verify the proper integrity and accuracy of backed up data.

Data should be archived periodically using a long-term stable medium and placed 'off-site' at a location other than that of the hardware to ensure safety. Such archives should be challenged at least annually to verify data retrieval.

Procedures should be defined for:

- investigation and correction of discrepancies in the database;
- corrective actions to be taken when validation testing yields unexpected results;
- handling, reporting, documenting and, if needed, correcting real-time problems, errors and alarms;
- manual operations (contingency planning) in the event of any system outage (even partial).

Quality assurance

The quality assurance programme should exercise oversight of the electronic data processing systems that affect product quality. At a minimum, such oversight should include:

- ensuring the ongoing accuracy and completeness of all documentation on equipment, software maintenance and operator training;
- performing periodic audits to verify proper accomplishment of all performance tests, routine maintenance, change procedures, data integrity checks, error investigations and operator competency evaluations.

4. Electronic signature

Strict rules must be defined on using electronic signatures to document or track required activities when collecting, processing, testing, transporting, holding, distributing or issuing blood components.

Many member states have specific requirements for the use of electronic signatures in specific documentation or points of the transfusion chain.

When in use, electronic signatures must be compliant with the community framework for the use of electronic signatures and must guarantee full compliance with the standards on traceability and documentation established by the EU Directives 2005/61 & 62. Member states must ensure that electronic signatures can be used as evidence in legal proceedings in all member states.

The 'advanced electronic signature' defines a process that does not describe a particular technology, but rather a process that creates an enforceable electronic signature if the signature:

- is uniquely linked to the signatory;
- is capable of identifying the signatory;
- is created using means that the signatory can maintain under their sole control;
- is linked to the data to which it relates in such a manner that any subsequent change in the data is detectable.

An advanced signature is generally taken to be a specific type of electronic signature that meets an additional set of criteria for signatory

identification. The main purpose of an advanced electronic signature is authentication, i.e. to give added assurance that the individual signing the message really is the person that he or she claims to be.

General requirements

Each electronic signature should be unique to one individual and should not be reused by, or reassigned to, anyone else.

Before an organisation establishes, assigns, certifies or otherwise sanctions an individual's electronic signature, or any element of such electronic signature, the organisation should verify the identity of the individual.

Signature manifestations

Signed electronic records should contain information associated with the signing that clearly indicates all of the following:

- the printed name of the signatory;
- the date and time when the signature was executed;
- the meaning (such as review, approval, responsibility, or authorship) associated with the signature.

Signature/record linking

Electronic signatures should be linked to their respective electronic records to ensure that the signatures cannot be excised, copied or otherwise transferred so as to falsify an electronic record by ordinary means.

Controls for identification codes/passwords/biometrics

Persons who use electronic signatures based upon use of identification codes in combination with passwords should employ controls to ensure their security and integrity. Such controls shall include:

- maintaining the uniqueness of each combined identification code and password, such that no two individuals have the same combination of identification code and password;

- ensuring that identification code and password issuances are periodically checked, recalled, or revised (e.g., to cover such events as password ageing);
- following loss management procedures to electronically unauthorise lost, stolen, missing, or otherwise potentially compromised tokens, cards, and other devices that bear or generate identification code or password information, and to issue temporary or permanent replacements using suitable, rigorous controls;
- use of transaction safeguards to prevent unauthorised use of passwords and/or identification codes, and to detect and report in an immediate and urgent manner any attempts at their unauthorised use to the system security unit, and, as appropriate, to organisational management;
- initial and periodic testing of devices, such as tokens or cards, that bear or generate identification code or password information, to ensure that they function properly and have not been altered in an unauthorised manner.

APPENDIX 4.
STATISTICAL PROCESS CONTROL

1. Introduction

Statistical Process Control (SPC) is a tool that enables an organisation to detect changes in the processes and procedures it carries out by monitoring data collected over a period of time in a standardised fashion. SPC became mandatory in 2005 for blood establishments in the EU (Directive 2004/33/EC). Methods and standards for the application of SPC to quality assurance of blood components need to be continuously studied and further developed. The technique can be applied to all activities in a blood facility, including administrative/clerical, scientific and technical processes. It is important that the processes to which SPC are to be applied are prioritised due to the amount of work involved. Currently, SPC is proving most beneficial in monitoring the performance of infectious markers and leucocyte-depletion testing. SPC is one of the few methods that can show how an improvement to a process has achieved the desired result, and enables decision-making to be placed on a much more rational and scientific basis.

2. Implementation of SPC

As for all other aspects of quality, implementation of SPC demands understanding and commitment on the part of the management of the blood facility. It must be included in the quality system of the facility, and a training programme should be introduced for senior management as well as operational staff. Plans must be made for data collection, including of control charts, and all matters dealing with changes detected in processes, especially sudden situations. Regular reviews of processes against SPC data should take place, with the specific objective of continuous improvement.

3. Strategy for statistical sampling

As much as possible, the number and frequency of components sampled for quality control and the number of test failures per sample that trigger an appropriate response (e.g. investigation or re-validation of materials and procedures) should be based on the considerations detailed below.

Tolerance of failure

A ‘target failure rate’ should be established as the failure rate that should not be exceeded. This ensures that monitoring of aspects of quality is continuous and that a failure rate exceeding target values triggers appropriate corrective action.

Confidence level

A confidence level should be set for the detection of an actual failure rate that lies above the ‘target failure rate’.

A valid method of statistical analysis should be used to determine either actual failure rate lies above the ‘target failure rate’.

4. Frequency of control sampling

A number of challenges arise in framing statistically based quality control testing programs for labile blood components. Due to the complexity of the transfusion system, blood facilities should consult statistical experts when designing process control systems. Issues include the: very large variation in volumes of blood components at different blood establishments; need to minimise losses in blood components through testing at small centres; very low expected rate of non-conformance for some processes, and the number of discrete conditions that arise in the manufacture of otherwise similar components. These may include:

- number of sites, operators and work shifts;
- different collection and processing systems and equipment;
- use of multiple reagent lots;
- alternative preparation times and temperatures;
- donor-related variables may affect the final quality of the blood component, even in a fully controlled process (e.g. for HbS donor blood with poor leucofiltration properties);

- the fact that blood components may be used for more than one clinical indication and require different levels of control (e.g. leucocyte-depleted RBCs for neonates vs for general transfusion).

Additionally, in many cases, the medical basis for currently accepted quality standards has not been rigorously established, making it difficult to determine the level of deviation from the expected level of conformance that can be tolerated. Nevertheless, to implement SPC, blood establishments need to establish the 'target failure rate' that should not be exceeded for each control test.

It is also desirable that the criterion for non-conformance should have at least a power of 80 per cent to detect the target failure rate, while giving a false-positive result in fewer than 5 % of determinations.

Consideration must also be given to the strategy for representative sampling of units for control testing. Because similar components are prepared under a variety of conditions, it is important that the sample set should include representative units prepared in all possible ways. Sampling may need to be stratified accordingly (i.e. to include a minimum number of samples from each condition).

The sample numbers specified for statistically valid process controls are minimum samples. In circumstances in which there are multiple processing conditions, and in blood establishments with large volumes of blood components, quality-control testing should be increased above the statistically determined minimum. This should be done in a controlled manner through the application of more rigorous statistical parameters, such as an increase in the expected proportion of samples that conform to a defined standard.

Additional considerations that may apply to the design of a quality control strategy include:

- the public-health importance of the standard being controlled (i.e. the period of time during which a process deviation could be tolerated before detection and correction);
- the overall blood component volume;

- the capacity for sampling and quality-control testing of the facility, including whether the quality-control testing is ablative (i.e. destructive of the processed blood component);
- the target failure rate of a process that is in control;
- a pre-defined strategy for managing non-process failures, e.g. a failed leucocyte-depletion procedure where further evaluation determined that the donor was HbS positive.

Three methods of statistical process control are provided below as examples.[1]

Example 1. Use of control charts

By plotting historical and prospective data on specially constructed charts, signs of process change can often be detected at an early stage, enabling remedial action to be taken. Steps for the construction of SPC charts are the same for all applications:

- collection of historical data;
- calculation of 'location and variation statistics' (see below);
- calculation of statistical control limits for the location and variation statistics;
- construction of the chart;
- plotting of prospective data.

Two types of data are conventionally collected:

- variable data, appropriate to anything that is measured directly such as cell count, pH, time taken for a process, etc.;
- attribute data, appropriate to anything that is counted on a 'yes or no' basis.

The type of SPC chart used depends on the type of data collected.

1 Beckman N, Nightingale MJ, Pamphilon D. Practical guidelines for applying statistical process control to blood component blood component. *Transfus Med* 2009; 19: 329-39.

Control charts for variable data

Major applications in a blood establishment are likely to be Individual/Moving Range charts and Average/Range charts.

Individual/Moving Range charts are used where a process is monitored by a single measurement on the sample, of the parameter in question e.g. residual leucocyte count on a platelet preparation. The steps for constructing an SPC chart are as follows:

- historical data are collected by measuring a random sample each day, and the moving range established by taking the difference between each sample and its predecessor;
- the location statistic is the average of the individual counts, whereas the variation statistic is the average moving range;
- the natural variation in a process is defined as the process average, plus or minus 3 standard deviations. Hence, the Upper Control Limit (UCL) and the Lower Control Limit (LCL) for the location and variation statistics are determined as the appropriate average, plus and minus 3 standard deviations;
- SPC charts conventionally have two distinct parts: one for the location statistic, which appears above the other for the variation statistic. For each part, the average is drawn as a solid line between two dotted lines that signify the UCL and LCL.

Prospective data are plotted on SPC charts in a similar way.

Average/Range charts are used in a situation where an early statistical response to a small process change is required, and where multiple control samples (up to 10) are subjected to the process. A typical example might be repeated use of a control sample during the daily use of a cytometer. In this situation, the average daily count on the control sample is calculated, the location statistic being the average of the averages. Each day shows a range in the control counts; the variation statistic is the average of these ranges. The Average/Range chart is then constructed in a similar manner to the Individual/Moving Range chart, except that the LCL for the Range part of the chart is, by definition, zero.

Control charts for attribute data

Attribute data, in general, fall into one of two categories: those counting the number of units sampled which are defective, and those counting the incidence of non-conformance to a requirement (each non-conformance in this case being classified as a defect). For example, a completed form is classified as 'defective' even if it contains only one non-conformance (though it may, in fact, contain multiple defects).

Attribute charts for the proportion of defective units (sometimes known as p-charts) are based on the calculation of the proportion of units found to be defective, i.e. having one or more defects per unit sampled, in sets of units sampled at intervals. The location statistic for the attribute is calculated by dividing the total number of defective units by the total number of units sampled, unless the sets of samples are always the same size, in which case the average of the proportion of defective units in each set may be taken. Since the data stem from yes/no criteria, attribute charts do not have a variation statistic.

UCL and LCL are determined as described above. In this system, it is possible to arrive at a negative value for the LCL, in which case it defaults to zero.

It should be noted that the calculation of standard deviation in a yes/no system such as this depends on the sample size. Hence, an increase or decrease in the set of units sampled necessitates re-establishing the UCL and LCL. An increase in sampling size generally results in convergence of UCL and LCL, making the system more sensitive to changes in the process.

Construction of the chart is carried out as described above.

Attribute charts for defects (sometimes known as u-charts) are generally useful when the object under investigation often has more than one non-conformance with requirements. They are well-suited to the control of clerical procedures. Collection of historical data involves counting the number of defects in each unit of a set of samples, repeated at intervals.

The location statistic is the average number of defects per unit, calculated by dividing the total number of defects in the total number of historical samples. As before, there is no variation statistic for attribute data.

Once again, UCL and LCL are calculated on the basis of the location statistic, plus and minus 3 standard deviations. Standard deviation in this system again depends on sample size, and any prospective increase requires re-establishment of the UCL and LCL.

The likely result is a convergence on the average, facilitating the detection of smaller changes in the process.

Construction of the u-chart follows the convention set for all SPC charts.

Interpretation of control charts

In general, if prospective data are plotted on the control chart and they follow the pattern established using historical data, the process may be assumed to be 'in control'. Changes in the pattern area reliable and sensitive means of detecting that a change has taken place in the process, warranting investigations into the cause. Rules have been established to give guidance to users as to when a change has occurred:

- rule 1: any point outside one of the control limits;
- rule 2: seven consecutive points all above or all below the average line;
- rule 3: seven consecutive points all increasing or all decreasing (a particular indicator of drift in the process average or range).

In addition, any unusual pattern or trend within the control lines may be an indicator of change.

Should information from the charts indicate that unplanned change is taking place within the process, action should be taken to identify any specific or common cause of the change. Application of SPC is the most reliable way of confirming that measures taken to improve the efficiency of a process are giving the desired results, by showing

reduction in variation around the mean (for measured data) or a trend toward zero defects (for counted data).

Example 2. Method of scan statistics

The method of scan statistics provides a suitable model for determining the frequency of control testing.[2] In this method, the number of non-conforming test results in a fixed sample size is determined. However, the sample set is regarded as a 'window' of observations that 'moves' progressively as test results are accumulated. For example, if the 'window size' is set at 60 observations, the first test set includes observations 1 through to 60; the second test set includes observations 2 through to 61; the third test set includes observations 3 through to 62. Progression of the 'window' can also be done a few samples at a time, such as by addition of daily test results as a group. To apply this method, the blood facility must identify a reasonably large 'universe' of ultimate test samples, typically representing a year or more of testing, or a period after which routine re-validation might be expected to occur because of process modifications (e.g. equipment replacement, software upgrades). The size of the moving window can then be determined based on the expected rate of failed tests for a conforming process (as defined in the Quality Control tables of each component described in Chapter 5), the size of the test universe, and the target failure rate to be detected as indicating a non-conforming process. The table below shows the minimum failure rate that can be detected at 80 per cent or greater power in any single window of control tests for test criteria with false-positive rates below 5 %.

Requiring that the number of control tests in the 'window' should take place in the desired time interval yields the frequency of control testing.

The following example illustrates how the method of scan statistics can be used.

---

2 Glaz J, Naus J, Wallenstein S, Scan Statistics. 2001; Springer, New York.

A blood facility seeks to monitor the failure rate of Leucocyte-Depleted. The expected failure rate (rate of non-conforming tests for a conforming process) is taken to be 0.1 %. The facility sets an action trigger at 5 % as a means to detect a defective lot of filters. The quality-control standard is established to ensure, with at least 80 % confidence, that a true failure rate of 5 % would be detected, but at a false-positive rate below 5 % for a declaration of non-conformance.

For a blood facility with 400 quality-control tests per year (approximately 34 per month), a non-conforming process can be declared if, in any 'moving window' of 60 consecutive such tests, two or more non-conforming test results are found (i.e. the trigger is greater than one non-conforming test in any window of 60 tests). This model has a power of 80.8 % to detect a true rate of non-conformance of 5 % in any window of 60 tests, and near certainty to detect this rate over 1 year. Based on scan statistics, the false-positive rate of such declarations is only 2.0 %.

If the number of quality control tests is 1 200 per year (100 per month), a non-conforming process can be declared if in any 'moving window' of 120 sequential quality control tests, three or more non-conforming test results are found. The false-positive rate of such declarations is only 0.7 %. The power is 80.7 % to detect a non-conformance rate of 4.6 % (the power is 85.6 % to detect a 5 % failure rate) for any window of 120 tests, and near certainty over 1 year.

Table 1. Sample size ('window') and maximum number of failed tests allowed for a conforming process based on scan statistics

Allowed failure rate for a con-forming process	Number of tests in 'universe' (e.g. the number of tests per year)	Sample size (i.e. the fixed number of tests in a moving 'win-dow')	Maxi-mum allowed number of failed tests in window	False positive rate of test criterion	Minimum failure rate of a non-conforming process detectable at > 80 % power in any single 'window'	
					Minimum 'target fail-ure rate' for a non-con-forming process	Power to detect non-conforming process in any window of quality control tests
25 %	400	30	16	2.5 %	63 %	81.9 %
		60	26	2.9 %	50 %	81.7 %
	1 200	30	17	2.0 %	66 %	81.3 %
		60	27	3.8 %	52 %	83.0 %
10 %	400	30	9	3.5 %	40 %	82.4 %
		60	14	2.7 %	30 %	83.8 %
	1 200	30	10	2.8 %	43 %	81.1 %
5 %	400	30	6	3.7 %	29 %	81.0 %
		60	9	2.3 %	21 %	83.7 %
	1 200	30	7	2.2 %	33 %	82.3 %
1 %	400	30	3	1.0 %	18 %	81.4 %
		60	4	0.9 %	11 %	80.3 %
	1 200	60	4	2.7 %	11 %	80.3 %

Allowed failure rate for a con-forming process	Number of tests in 'universe' (e.g. the number of tests per year)	Sample size (i.e. the fixed number of tests in a moving 'win-dow')	Maxi-mum allowed number of failed tests in window	False positive rate of test criterion	Minimum failure rate of a non-conforming process detectable at > 80 % power in any single 'window'	
					Minimum 'target fail-ure rate' for a non-con-forming process	Power to detect non-conforming process in any window of quality control tests
0.1 %	400	30	1	1.1 %	10 %	81.6 %
		60	1	2.0 %	5 %	80.8 %
	1 200	30	1	3.2 %	10 %	81.6 %
		120	2	0.7 %	4.6 %	80.7 %

Example 3. Statistical process control for dichotomous outcomes: an approach based upon hypergeometric/binomial distributions

A hypergeometric distribution is based upon random sampling (without replacement) of a factor that has a dichotomous outcome. This distribution is applicable for the assessment of quality control measures related to blood components for which the outcome is pass/fail. A binomial distribution is very similar to a hypergeometric distribution, but it is based upon sampling with replacement. At sampling levels of $n \geq 59$ to meet the 95 % criterion, these two distributions are essentially identical.

For statistical quality control using the hypergeometric/binomial approach, a cycle is defined as the blood-component volume that is being subject to quality assessment within a defined time period. The appropriate size for a quality-control cycle is determined based upon

the desired frequency of control sampling as described above and the selected proportion of conforming samples.[3]

Statistical quality control based upon a hypergeometric distribution is applicable for cycle sizes between n = 30 and n = 4 500.[4] Successful control requires that predetermined random sample sizes be assessed with an outcome of 0, 1 or 2 failures, depending on the cycle size.

For cycle sizes above n = 4 500, the hypergeometric distribution approaches the binomial distribution and the traditional binomial approach can be applied, i.e. assessing n = 60 random samples per cycle with an outcome of zero failures; n = 93 with one failure or n = 124 with 2 failures.

---

3 For example, 95 % conformance (and the resulting high level of quality-control testing) would be appropriate for a safety-related blood component standard such as residual leucocytes in a Leucocyte-Depleted component. However, 75 % conformance may be acceptable for a standard such as components content, where standardisation is desirable, but is not directly related to recipient safety.

4 For a cycle size of 30, greater than 95 % conformance is reflected by, at most, one non-conforming unit because 29/30 = 96.7 % and 28/30 = 93.3 %. To define this conformance statistically, it is necessary to be able to conclude with 95 % confidence that greater than 95 % of the units are conforming (i.e. ≤ n = 1 non-conforming unit for a cycle size of n = 30). Using a null hypothesis that there are at least two non-conforming units among the 30 units, the alternative hypothesis is that there are fewer than two non-conforming units among the 30 units. Under this null hypothesis, the probability that the first 22 units are all good is 6.4 %, which is calculated as:

$$\frac{28}{30}\times\frac{27}{29}\times\frac{26}{28}\cdots\frac{9}{11}\times\frac{8}{10}\times\frac{7}{9}=\frac{8\times7}{30\times29}=0.064$$

So the null hypothesis cannot be rejected at the 5 % significance level, which corresponds to 'with 95 % confidence'.

Under the null hypothesis stated above, the probability that the first 23 units are all good is 4.8 %:

$$\frac{28}{30}\times\frac{27}{29}\times\frac{26}{28}\cdots\frac{8}{10}\times\frac{7}{9}\times\frac{6}{8}=\frac{7\times6}{30\times29}=0.048$$

So the null hypothesis can be rejected at the 5 % significance level which corresponds to 'with 95 % confidence'. Thus, 23 samples without a non-conformance are needed to conclude with 95 % confidence that greater than 95 % of the units are conforming.

The table below provides random sample sizes across a range of cycle sizes. With a larger cycle size, 1 or 2 occurrences of non-conformance are allowed in conjunction with a larger pre-specified sample size.

For example, if the cycle size is 65 (95 %/95 %), there are three options that need to be pre-determined: a sample size of 34 without any failure, a sample size of 49 with 1 failure, or a sample size of 59 with 2 failures. If (i) a sample size of 34 and observation of one failure, or (ii) a sample size of 49 and observation of two failures is chosen, 100 per cent quality control can still be done to make the final determination, whether or not greater than 95 per cent of the components meet the standard.

After the cycle size reaches 7 000 for 95 %/95 % and 13 000 for 95 %/75 %, the results based on the hypergeometric distribution are same as those based on a binomial distribution.

Table 2. Sizes of random samples needed at various quality control cycle sizes to assess 95 %, 90 % or 75 % conformance to a standard with 95 % confidence

	**95 %/95 %** *95 % confidence that > 95 % of the components meet the standard*				**95 %/90 %** *95 % confidence that > 90 % of the components meet the standard*				**95 %/75 %** *95 % confidence that > 75 % of the components meet the standard*			
**Lot size**	**Failures allowed in lot**	**Sample size**			**Failures allowed in lot**	**Sample size**			**Failures allowed in lot**	**Sample size**		
		No failure	*1 failure allowed*	*2 failures allowed*		*No failure*	*1 failure allowed*	*2 failures allowed*		*No failure*	*1 failure allowed*	*2 failures allowed*
30	1	23	30	N/A	2	19	26	30	7	9	13	17
31	1	24	31	N/A	3	16	23	28	7	9	14	18
32	1	25	32	N/A	3	17	24	29	7	9	14	18
33	1	26	33	N/A	3	17	25	30	8	9	13	17
34	1	26	34	N/A	3	18	25	31	8	9	14	18
35	1	27	35	N/A	3	18	26	32	8	9	14	18
36	1	28	36	N/A	3	19	27	33	8	9	15	19
37	1	29	37	N/A	3	19	28	33	9	9	14	18

	**95 %/95 %** *95 % confidence that > 95 % of the components meet the standard*				**95 %/90 %** *95 % confidence that > 90 % of the components meet the standard*				**95 %/75 %** *95 % confidence that > 75 % of the components meet the standard*			
**Lot size**	**Failures allowed in lot**	**Sample size**			**Failures allowed in lot**	**Sample size**			**Failures allowed in lot**	**Sample size**		
		No failure	*1 failure allowed*	*2 failures allowed*		*No failure*	*1 failure allowed*	*2 failures allowed*		*No failure*	*1 failure allowed*	*2 failures allowed*
38	1	30	38	N/A	3	20	28	34	9	9	14	18
39	1	30	39	N/A	3	20	29	35	9	9	15	19
40	1	31	39	N/A	3	21	30	36	9	10	15	19
45	2	28	39	45	4	20	29	36	11	9	14	19
50	2	31	43	50	4	22	33	40	12	9	15	19
55	2	35	48	55	5	21	32	40	13	10	15	20
60	2	38	52	60	5	23	34	43	14	10	16	21
65	3	34	49	59	6	22	33	42	16	10	15	20
70	3	37	52	63	6	24	36	46	17	10	16	20
75	3	39	56	68	7	23	35	44	18	10	16	21

	**95 %/95 %** *95 % confidence that > 95 % of the components meet the standard*				**95 %/90 %** *95 % confidence that > 90 % of the components meet the standard*				**95 %/75 %** *95 % confidence that > 75 % of the components meet the standard*			
**Lot size**	**Failures allowed in lot**	**Sample size**			**Failures allowed in lot**	**Sample size**			**Failures allowed in lot**	**Sample size**		
		No failure	*1 failure allowed*	*2 failures allowed*		*No failure*	*1 failure allowed*	*2 failures allowed*		*No failure*	*1 failure allowed*	*2 failures allowed*
80	3	42	60	72	7	24	37	47	19	10	16	21
85	4	38	56	69	8	23	36	46	21	10	16	21
90	4	40	59	73	8	25	38	49	22	10	16	21
95	4	42	62	77	9	24	37	47	23	10	16	21
100	4	45	65	81	9	25	39	50	24	10	16	22
120	5	47	69	87	11	26	40	52	29	10	17	22
140	6	48	72	92	13	26	41	53	34	11	17	22
160	7	49	75	95	15	27	41	54	39	11	17	22
180	8	50	77	98	17	27	42	55	44	11	17	22
200	9	51	78	101	19	27	42	55	49	11	17	23

	95 %/95 % *95 % confidence that > 95 % of the components meet the standard*				95 %/90 % *95 % confidence that > 90 % of the components meet the standard*				95 %/75 % *95 % confidence that > 75 % of the components meet the standard*			
**Lot size**	**Failures allowed in lot**	**Sample size** *No failure*	*1 failure allowed*	*2 failures allowed*	**Failures allowed in lot**	**Sample size** *No failure*	*1 failure allowed*	*2 failures allowed*	**Failures allowed in lot**	**Sample size** *No failure*	*1 failure allowed*	*2 failures allowed*
220	10	52	79	103	21	27	42	56	54	11	17	23
240	11	52	80	104	23	27	43	56	59	11	17	23
260	12	53	81	106	25	27	43	57	64	11	17	23
280	13	53	82	107	27	28	43	57	69	11	17	23
300	14	54	83	108	29	28	43	57	74	11	17	23
320	15	54	83	109	31	28	44	57	79	11	17	23
340	16	54	84	110	33	28	44	58	84	11	17	23
360	17	54	85	111	35	28	44	58	89	11	17	23
380	18	55	85	111	37	28	44	58	94	11	17	23
400	19	55	85	112	39	28	44	58	99	11	17	23

	**95 %/95 %** *95 % confidence that > 95 % of the components meet the standard*				**95 %/90 %** *95 % confidence that > 90 % of the components meet the standard*				**95 %/75 %** *95 % confidence that > 75 % of the components meet the standard*			
**Lot size**	**Failures allowed in lot**	**Sample size**			**Failures allowed in lot**	**Sample size**			**Failures allowed in lot**	**Sample size**		
		No failure	*1 failure allowed*	*2 failures allowed*		*No failure*	*1 failure allowed*	*2 failures allowed*		*No failure*	*1 failure allowed*	*2 failures allowed*
450	22	54	84	111	44	28	44	59	112	11	17	23
500	24	56	87	114	49	28	44	59	124	11	17	23
550	27	55	86	113	54	28	45	59	137	11	17	23
600	29	56	88	116	59	28	45	59	149	11	17	23
650	32	56	87	115	64	28	45	59	162	11	17	23
700	34	57	89	117	69	28	45	60	174	11	17	23
750	37	56	88	116	74	28	45	60	187	11	17	23
800	39	57	89	118	79	28	45	60	199	11	17	23
850	42	56	89	117	84	28	45	60	212	11	17	23
900	44	57	90	119	89	28	45	60	224	11	17	23

	**95 %/95 %** *95 % confidence that > 95 % of the components meet the standard*				**95 %/90 %** *95 % confidence that > 90 % of the components meet the standard*				**95 %/75 %** *95 % confidence that > 75 % of the components meet the standard*			
**Lot size**	**Failures allowed in lot**	**Sample size**			**Failures allowed in lot**	**Sample size**			**Failures allowed in lot**	**Sample size**		
		No failure	*1 failure allowed*	*2 failures allowed*		*No failure*	*1 failure allowed*	*2 failures allowed*		*No failure*	*1 failure allowed*	*2 failures allowed*
950	47	57	89	118	94	29	45	60	237	11	17	23
1000	49	57	90	119	99	29	45	60	249	11	17	23
1500	74	58	91	121	149	29	45	60	374	11	17	23
2000	99	58	92	122	199	29	46	61	499	11	17	23
2500	124	58	92	122	249	29	46	61	624	11	17	23
3000	149	58	92	123	299	29	46	61	749	11	17	23
3500	174	58	93	123	349	29	46	61	874	11	17	23
4000	199	58	93	123	399	29	46	61	999	11	17	23
4500	224	59	93	123	449	29	46	61	1124	11	17	23
5000	249	59	93	123	499	29	46	61	1249	11	17	23

	**95 %/95 %** *95 % confidence that > 95 % of the components meet the standard*				**95 %/90 %** *95 % confidence that > 90 % of the components meet the standard*				**95 %/75 %** *95 % confidence that > 75 % of the components meet the standard*			
**Lot size**	**Failures allowed in lot**	**Sample size**			**Failures allowed in lot**	**Sample size**			**Failures allowed in lot**	**Sample size**		
		No failure	*1 failure allowed*	*2 failures allowed*		*No failure*	*1 failure allowed*	*2 failures allowed*		*No failure*	*1 failure allowed*	*2 failures allowed*
6000	299	59	93	123	599	29	46	61	1499	11	17	23
7000	349	59	93	124	699	29	46	61	1749	11	17	23
8000	399	59	93	124	799	29	46	61	1999	11	17	23
9000	449	59	93	124	899	29	46	61	2249	11	17	23
10000	499	59	93	124	999	29	46	61	2499	11	17	23
11000	549	59	93	124	1099	29	46	61	2749	11	17	23
12000	599	59	93	124	1199	29	46	61	2999	11	17	23
13000	649	59	93	124	1299	29	46	61	3249	11	18	23
14000	699	59	93	124	1399	29	46	61	3499	11	18	23
15000	749	59	93	124	1499	29	46	61	3749	11	18	23

APPENDIX 5.
HEALTH ECONOMICS IN BLOOD TRANSFUSION

1. Overview

Providing blood is expensive and the heavy burden that it places on national health budgets may continue to grow as it becomes necessary to implement further safety measures, including extra screening tests, new pathogen reduction technologies and additional quality requirements. Under these circumstances, costs throughout the blood transfusion chain from donor to recipient are bound to come under intense scrutiny as funders seek to economise and increasingly demand value for money.

The objective for blood establishments responsible for preparing, controlling and issuing blood components should be to use appropriate means in order to economise and reduce capital and recurrent costs in the blood transfusion service, but without compromising the quality, effectiveness and safety of their therapeutic blood components for the benefit of patients in need of transfusion.

Therefore, healthcare managers and professionals in blood transfusion and quality management should be aware of cost structures in the blood transfusion chain, in conjunction with efforts to optimise the use of blood components and minimise relative costs.

2. Investing in quality

Evidence-based data and research on the economics of blood are limited. Standard methods for costing and financial planning should be established to enable the calculation of total economic costs associated with blood services, bench-marking, budget planning, financial accountability, purchasing and logistics.

Competent Authorities for blood transfusion should define priorities and decide on the data and indicators that must be collected. The blood supply chain from donor to patient should be analysed to identify opportunities for cost reductions. Best practices should be implemented using effective bench-marking procedures. The contribution of management tools towards controlling costs and improving the efficiency of blood transfusion should be evaluated.

3. Costing analysis

The criteria used for cost analysis and realistic cost-effectiveness projections at national, regional and local level should comply with WHO guidelines for costing blood transfusion services.

An important step towards a cost-effectiveness analysis is to define the regulatory framework in order to allow the estimation of costs and outputs of specific activities. An activity-based cost procedure should identify major groups of activities in the blood service, with cost-output measurable indicators defined for each area (e.g. blood collection, blood processing, blood storage and distribution, haemovigilance). The total costs for each activity include both capital (building, equipment, training, furniture, vehicles, etc.) and recurrent costs (personnel, supplies, transportation, utilities, administration, insurance, etc.).

Managers of blood transfusion services (BTS) should define the objectives of cost analyses for the purposes of budget planning, financial accountability and evaluation, and cost effectiveness analysis. In this way, cost information can be used to monitor the efficiency of the components of BTS, and for resource mobilisation and other tasks.

4. Modelling cost-effectiveness analysis in transfusion

BTS managers need to collect data to support analyses of cost-effectiveness based on the following rules:

- the central element is the activity, defined as a set of inter-linked tasks resulting in the production of goods and services;
- activities are not isolated, but are part of a process;
- each activity has a supplier and a client (internal and external) and contributes to the creation of value.

The BTS manager should perform for each activity:

- calculation of blood component costs;
- calculation of selling prices;

- calculation of margins between selling prices and costs;
- cost accounting with a view to benchmarking;
- decision-making regarding the possible introduction of an innovation and the choice between alternative methods.

5. Economic aspects of the clinical use of blood

The economic aspects of the clinical use of blood should also be evaluated in relation to outcomes and effectiveness, taking into account parameters such as the amount of blood component administered, duration of treatment, length of hospital stay and quality adjusted life years (QALYs). Inappropriate use of blood (i.e. in terms of having unexpected adverse reactions and a direct bearing on healthcare budgets) should be investigated in order to substantiate the cost-benefit and the cost-effectiveness of transfusion.

Carrying out an economic evaluation of expenditures related to the use of blood and blood components involves the identification of the therapeutic use of blood components and the costs from the initiation of treatment to its completion.

Assessing the economic implications and effectiveness of therapeutic interventions would be facilitated by measuring outcomes and effectiveness. Therefore, it is necessary to record data both before and after the use of blood components, in order to substantiate the benefits that accrue.

Alternative treatment strategies using blood components need to be examined with respect to therapeutic outcomes and in relation to cost-benefit, cost-effectiveness and cost utility.

Methods for evaluating a more expensive therapy (e.g. leucocyte-depleted cells) against a cheaper one should be considered, given that the former may result in a shorter hospital stay and as a consequence reduced hospital charges.

Inappropriate use of blood has a direct bearing on healthcare budgets. Over and under-use of blood components may harm the patient. Misuse of blood may also result in an unexpected adverse outcome.

DEFINITIONS

**Additive solution**	Solution specifically formulated to maintain beneficial properties of cellular components during storage.
**Advanced electronic signature**	An electronic signature which meets the following requirements: (a) it is uniquely linked to the signatory; (b) it is capable of identifying the signatory; (c) it is created using means that the signatory can maintain under their sole control; and (d) it is linked to the data to which it relates in such a manner that any subsequent change of the data is detectable.
**Adverse event**	Any untoward occurrence associated with the collecting, testing, processing, storage and distribution of blood and blood components that might lead to an adverse reaction in blood recipients or blood donors.
**Adverse reaction**	Any unintended response in a donor or patient associated with the collection or transfusion of blood or blood components.
**Affected area**	An area with ongoing transmission of an infection to humans. This means that there has been at least one autochthonous case as a result of local transmission in the area according to the agreed, standardised and disease-specific case definition. Under exceptional circumstances, a probable case can be used to determine transmission but only in specific and agreed situations when case confirmation cannot be performed within a reasonable time.
**Allogeneic donation**	Blood and blood components collected from an individual and intended for transfusion to another individual, for use in medical devices or as starting material/raw material for manufacturing into medicinal products.
**Antibody quantitation**	Technique routinely used to measure the level of antibody, i.e. anti-RhD (or anti-c) antibody in maternal sera.
**Antiglobulin testing technique**	The direct antiglobulin test (direct Coombs' test) and the indirect antiglobulin test. It detects antibody or complement bound to red cells in vivo.
**Anti-IgA antibodies**	IgG or occasionally IgM anti-IgA produced by an IgA- deficient patient. Severe anaphylactoid transfusion reactions can occur in such patients.
**Apheresis**	Method of obtaining one or more blood components by machine processing of whole blood, in which the residual components of the blood are returned to the donor during or at the end of the process.

**Audit programme**	A systematic and independent examination to determine whether quality activities and related results comply with planned arrangements and whether these arrangements are implemented effectively and are suitable to achieve objectives.
**Autologous collection**	Autologous collection means blood and blood components collected from an individual and intended solely for subsequent autologous transfusion or other human application to that same individual.
**Autologous donors**	Individuals who give blood for their own use if the need for blood can be anticipated and a collection plan developed.
**Autologous transfusion**	Transfusion in which the donor and the recipient are the same person and in which pre-deposited blood and blood components are used.
**Automated system**	A broad range of systems including, but not limited to, automated processing equipment, automated laboratory equipment, process control, processing execution, laboratory information management, processing resource planning and document management systems. The automated system consists of the hardware, software and network components, together with the controlled functions and associated documentation.
**Biometrics**	A method of verifying an individual's identity based on measurement of the individual's physical feature(s) or repeatable action(s) where those features and/or actions are both unique to that individual and measurable.
**Blood component**	Therapeutic components of blood (red cells, white cells, platelets, plasma) that can be prepared by centrifugation, filtration and freezing using conventional methodologies in blood establishment.
**Blood component release**	Procedure which enables a blood component to be released from a quarantine status by the use of systems and procedures to ensure that the finished product meets its release specifications.
**Blood establishment**	Any structure or body that is responsible for any aspect of the collection and testing of human blood or blood components, whatever their intended purpose, and their processing, storage and distribution if intended for transfusion. This does not include hospital blood banks.
**Blood product**	Any therapeutic product derived from human blood or plasma.
**Buffy coat**	Blood component prepared by centrifugation of a unit of whole blood, which contains a considerable proportion of the leucocytes and platelets.

**Buoyant density centrifugation**	Technique for separation based on density differences between cells.
**Calibration**	Set of operations that establish, under specified conditions, the relationship between values indicated by a measuring instrument/system or values represented by a material measure and the corresponding known values of a reference standard.
**Case**	A particular disease, health disorder, or condition under investigation found in an individual or within a population or study group.
**Cell free plasma**	Plasma obtained by cross-flow filtration, when blood flows along a membrane with a pore size allowing free passage of plasma proteins, but not of blood cells.
**Cell separator**	An instrument for apheresis.
**Change control**	A formal system by which qualified representatives of appropriate disciplines review proposed or actual changes that might affect the validated status of facilities, systems, equipment or processes. The intent is to determine the need for action that would ensure and document that the system is maintained in a validated state.
**Complaint**	An act of expressing customer dissatisfaction with the quality of products or services provided by the responsible organisation.
**Computerised system**	A system comprising the input of data, electronic processing and the output of information to be used either for reporting, automatic control or documentation.
**Consent**	To give assent or approval, such as consent to be transfused
**Counter-current centrifugation (elutriation)**	Technique where cells subjected simultaneously to a liquid flow and a centrifugal force in opposite directions tend to be separated according to their size.
**CPD-Adenine (CPDA)**	Citrate-Phosphate-Dextrose with Adenine is a preservative-anticoagulant solution used for whole blood collection.
**Cryopreservation**	Prolongation of the storage life of blood components by freezing.
**Cytapheresis**	An apheresis procedure intended for the collection of a cellular component of blood, such as red cells, leucocytes or platelets.

**Depth and surface filtration**	Technique of filtration using a filter bed of fibres: owing to the specific properties of platelets and granulocytes, as well as the low flexibility of lymphocytes, these cells are more easily trapped in such filters than are red cells.
**Distribution**	Act of delivery of blood and blood components to other blood establishments, hospital blood banks, and manufacturers of blood- and plasma-derived products. It does not include issuing blood or blood components for transfusion.
**Donor**	A person in normal health with a good medical history who voluntarily gives blood or blood components for therapeutic use.
**Donor deferral**	Suspension of the eligibility of an individual to donate blood or blood components; such suspension being either permanent or temporary.
**Emergency**	A serious, unexpected, and potentially dangerous situation requiring immediate action.
**Endemic area**	A risk area where an infectious disease lingers at around the same incidence for a long time.
**Epidemiological surveillance**	The continuous gathering, analysing, and interpreting data about diseases, and disseminating conclusions of the analyses to relevant organisations and audiences.
**Facilities**	Hospitals, clinics, manufacturers and biomedical research institutions to which blood or blood components may be delivered.
**Febrile transfusion reactions**	A febrile response associated with the administration of blood or blood components.
**First-time donor**	Someone who has never donated either blood or a blood component.
**Full blood count**	Analysis of haematological parameters including Hb and RBC indices as well as counts of RBCs, white cells and platelets.
**Glycerol**	Propanetriol, used as a cell-cryoprotective agent for the storage of red cells in the frozen state.
**Good practice**	All elements in established practice that collectively lead to final blood or blood components that consistently meet pre-defined specifications and compliance with defined regulations.

**Haematocrit**	Result obtained by the measurement of the volume of red cells in blood, after centrifugation, expressed as a percentage or as a ratio in the SI system.
**Haematopoietic progenitor cells**	HPC are primitive pluripotent cells capable of self-renewal as well as differentiation and maturation into all haematopoietic lineages. They are found in bone marrow (bone marrow cells (BMC)), in the mononuclear cells of circulating blood (peripheral blood stem cells (PBSC)) and in umbilical cord blood (umbilical stem cells (USC)).
**Haemovigilance**	Organised surveillance procedures related to serious adverse or unexpected events or reactions in donors or recipients, and the epidemiological follow-up of donors.
**Hospital blood bank**	Hospital unit which stores and distributes and may perform compatibility tests on blood and blood components exclusively for use within the hospital facilities, including hospital-based transfusion activities.
**Imputability**	The likelihood that a serious adverse reaction in a recipient can be attributed to the blood or blood component transfused or that a serious adverse reaction in a donor can be attributed to the donation process.
**Inspection**	Formal and objective control according to adopted standards to assess compliance with a given directive and other relevant legislation and to identify problems.
**Issue**	The provision of blood or blood components by a blood establishment or a hospital blood bank for transfusion to a recipient. (Dir.2005/61/EC)
**Leucocyte depletion**	The removal of leucocytes from blood.
**Medicinal product**	Any substance or combination of substances presented as having properties for treating or preventing disease in human beings (Directive 2001/83/EC, 2003/94/EC)
**Mobile site**	A temporary or movable place used for the collection of blood and blood components which is in a location outside of, but under the control of the blood establishment.
**Open system**	A system in which a breach has occurred but every effort is made to prevent microbial contamination by operating in a clean environment using sterilised materials and aseptic handling techniques.
**Pathogen reduced (PR)**	A term applied to a blood component that has been prepared following the use of PRT.

**Pathogen reduction technologies (PRT)**	Procedures that irreversibly impede proliferation of pathogens, either by removal or inactivation with physical and/or chemical methods.
**Peripheral blood stem cells (PBSC)**	Primitive pluripotent cells capable of self-renewal as well as differentiation and maturation into all haematopoietic line ages, and found in the mononuclear cells of circulating blood (see haematopoietic progenitor cells).
**Plasma**	The liquid portion of the blood in which the cells are suspended. Plasma may be separated from the cellular portion of whole blood for therapeutic use as fresh frozen plasma or further processed to cryoprecipitate and cryoprecipitate-depleted plasma for transfusion. It may be used for the manufacture of medicinal products derived from human blood and human plasma or used in the preparation of pooled platelets, or pooled leucocyte-depleted platelets. It may also be used for resuspension of red cell preparations for exchange transfusion or perinatal transfusion.
**Platelet standard adult dose**	A dose of platelets derived from 4-6 whole blood donations or obtained by apheresis, with a minimum platelet content of 200×10^9 platelets.
**Prescription form**	A form on which the clinician prescribes a medicine or a blood component to be transfused to the patient.
**Preservation**	The use of chemical agents, alterations in environmental conditions or other means during processing to prevent or retard biological or physical deterioration of blood or blood components
**Pre-transfusion sampling**	Procedure for taking blood samples from the patient requiring a transfusion, for compatibility investigation.
**Procedure**	A procedure controls a distinct process or activity, including the associated inputs and outputs. A series of tasks usually performed by one person according to instructions.
**Process**	A set of related tasks and activities that accomplish a work goal.
**Processing**	Any step in the preparation of a blood component that is carried out between the collection of blood and the issuing of a blood component.
**Procurement**	A process by which blood or blood components are made available (Directive 2004/33/EC).
**Proficiency testing**	The evaluation of participant performance against pre-established criteria by means of external quality assessment scheme, inter-laboratory comparisons by use of externally sourced samples or panels.

**Qualification**	Part of validation: the action of verifying that any personnel, premises, equipment or material works correctly and delivers the expected result.
**Quality**	Totality of characteristics of an entity that bear on its ability to satisfy stated and implied needs. Consistent and reliable performance of services or products in conformity with specified standards.
**Quality assurance**	All the activities from blood collection to distribution carried out with the objective of ensuring that blood and blood components are of the quality required for their intended use.
**Quality control**	Part of a quality system focussed on fulfilling quality requirements.
**Quality management**	The co-ordinated activities to direct and control an organisation with regard to quality at all levels within the blood establishment.
**Quality monitoring**	That part of a quality assurance programme concerned with maintenance and improvement of quality which deals with the identification and use of indicators to detect variations from standards or specifications.
**Quality system**	The organisational structure, responsibilities, procedures, processes, and resources for implementing quality management.
**Quarantine**	The physical isolation of blood components or incoming materials/reagents over a variable period of time while awaiting acceptance, issuance or rejection of the blood components or incoming materials/reagents.
**Recipient**	Someone who has been transfused with blood or blood components.
**Reconciliation**	Comparison and assessment of any discrepancy between the amount of material entering and leaving a given operation or series of operations.
**Record**	Written or electronically captured evidence that an event has occurred or an outcome has been achieved. A document that contains objective evidence which shows how well activities are being performed or what kind or results are being achieved.
**Regular donor**	Someone who routinely donates their blood or plasma (i.e. within the last 2 years), in accordance with minimum time intervals, in the same donation centre.
**Repeat donor**	Someone who has donated before, but not within the last two years in the same donation centre.
**Replacement donor**	Donor recruited by a patient to enable them to undergo elective surgery.

**Reporting establishment**	The blood establishment, the hospital blood bank or facilities where the transfusion takes place that reports serious adverse reactions and/or serious adverse events to the competent authority.
**Resources**	Include people, money, information, knowledge, skills, energy, facilities, machines, tools, equipment, technologies and techniques.
**RhD Immunoglobulin**	Immunoglobulin specific for RhD antigen is given routinely to RhD-negative mothers bearing RhD- positive infants to protect them from red cell exposure during pregnancy and delivery, and so prevent allo-immunisation.
**Risk area**	An area where individuals are exposed to the risk (which can be small or large) of being infected with a locally-acquired infection. This is a generalised use of the term 'risk area,' to prevent the imprecision linked to this term due to its use to signify a specific level of risk in an area.
**Risk assessment**	Method to assess and characterise the critical parameters in the functionality of equipment, systems or processes.
**Self-inspection**	An audit carried out by people from within the organisation to ensure compliance with GPG and regulatory requirements.
**Serious adverse event**	Any untoward occurrence associated with the collecting, testing, processing, storage and distribution of blood and blood components that might lead to death or life-threatening, disabling or incapacitating conditions for donors or recipients or which results in, or prolongs, hospitalisation or morbidity.
**Serious adverse reaction**	Unintended response in donor or in recipient associated with the collection or transfusion of blood or blood components that is fatal, life-threatening, disabling, incapacitating, or which results in, or prolongs hospitalisation or morbidity.
**Signatory**	A person who holds a signature creation device and acts either on their own behalf or on behalf of the natural or legal person or entity they represent.
**Specification**	Description of the criteria that must be fulfilled in order to achieve the required quality standard.
**Standard**	The requirements that serve as the basis for comparison.
**Standard operating procedures (SOPs)**	Detailed written procedures that give direction for performing certain operations.

**Statistical process control**	Method of quality control of a product or a process that relies on a system of analysis of an adequate sample size, without the need to measure every product of the process.
**Sterile connecting device**	A device that connects two tubes without breaching the sterility of their interior.
**Storage**	Maintaining the product under appropriate controlled conditions until distribution or issue.
**Surveillance**	Systematic and continuous collection, analysis, and interpretation of data, closely integrated with the timely and coherent dissemination of the results and assessment to those who have the right to know so that action can be taken.
**Traceability**	The ability to trace each individual unit of blood or blood component derived thereof from the donor to its final destination, whether this is a recipient, a manufacturer of medicinal products or disposal, and vice versa.
**Trace-back**	The process of investigating a report of a suspected transfusion-associated adverse reaction in a recipient to identify a potentially implicated donor.
**Validation**	Refers to establishment of documented and objective evidence that the pre-defined requirements for a specific procedure or process can be fulfilled consistently.
**Validation plan**	Description of validation activities, responsibilities and procedures. It describes specifically how a certain validation is to be done.
**Washed**	A process of removing plasma or storage medium from cellular components by centrifugation, decanting of the supernatant liquid from the cells and addition of an isotonic suspension fluid, which in turn is generally removed and replaced following further centrifugation of the suspension. The centrifugation, decanting, replacing process may be repeated several times.
**Washed red cells**	A component derived from whole blood by centrifugation and removal of plasma, with subsequent washing of the red cells in an isotonic solution.
**Whole blood**	Blood collected from a single donor and processed either for transfusion or further manufacturing.

**Written procedures**	Controlled documents that describe how specified operations are to be carried out.
**Xenotransplantation**	Any procedure that involves transplantation or infusion into a human recipient of live animal cells, tissues or organs, or human body fluids, cells, tissues or organs that have ex vivo contact with live animal cells, tissues or organs.

ABBREVIATIONS

**Ag**	Antigen
**AIDS**	Acquired Immune Deficiency Syndrome
**ALT**	Alanine Amino Transferase
**AML**	Acute Myeloid Leukaemia
**AS**	Additive Solution
**AS-BCR**	Additive Solution-Buffy Coat Removed
**BCR**	Buffy Coat Removed
**BPAT**	Batch Pre-Acceptance Testing
**BSE**	Bovine Spongiform Encephalopathy
**BTS**	Blood Transfusion Services
**CAPA**	Corrective and Preventative Action
**CD-P-TS**	European Committee on Blood Transfusion
**CETS**	Council of Europe Treaty Series (formerly ETS: European Treaty Series)
**CJD**	Creutzfeldt–Jakob disease
**CMV**	Cytomegalovirus
**DMSO**	Dimethylsulfoxide
**DQ**	Design Qualification
**EC**	European Commission
**EDQM**	European Directorate for the Quality of Medicines & HealthCare
**ELISA**	Enzyme-linked immuno-sorbent assay
**EMA**	European Medicines Agency
**EU**	European Union
**FFP**	Fresh Frozen Plasma
**FTA**	Fluorescent Treponemal Antibody

**GCSF**	Granulocyte Colony Stimulating Factor
**GMP**	Good Manufacturing Practice
**GPG**	Good Practice Guidelines
**GTS**	Ad hoc working group on the guide to the preparation, use and quality assurance of blood components
**ECV**	Extracorporeal volume
**Hb**	Haemoglobin
**HBc**	Hepatitis B core antigen
**HBsAg**	Hepatitis B surface antigen
**HCV**	Hepatitis C virus
**Hct**	Haematocrit
**HES**	Hydroxyethyl starch
**HIV**	Human Immunodeficiency virus
**HLA**	Human Leucocyte Antigen
**HPA**	Human Platelet Antigen
**HTLV**	Human T-cell lymphotropic virus
**INR**	International Normalised Ratio
**IQ**	Installation Qualification
**ISBT**	International Society of Blood Transfusion
**IU**	International Unit
**JACIE**	Joint Accreditation Committee-ISCT (Europe) & EBMT
**LD**	Leucocyte-Depleted
**LISS**	Low Ionic Strength (Salt) Solution
**MDS**	Myelodysplasia

**NAT**	Nucleic Acid Amplification Techniques
**OQ**	Operational Qualification
**PAT**	Pre-deposit Autologous Donation
**Ph. Eur.**	European Pharmacopoeia
**PQ**	Performance Qualification
**PR**	Pathogen Reduced
**PRP**	Platelet-rich Plasma
**PRT**	Pathogen Reduction Technology
**QA**	Quality Assurance
**QC**	Quality Control
**RBC**	Red Blood Cells
**SAGM**	Saline Adenine Glucose Mannitol solution
**SOPs**	Standard Operating Procedures
**SPC**	Statistical Process Control
**T. cruzi**	Trypanosoma cruzi
**TA**	Transfusion-Associated
**TA-GVHD**	Transfusion-Associated Graft-Versus-Host disease
**TACO**	Transfusion-Associated Circulatory Overload
**TPHA**	Treponema pallidum Haemagglutination Assay
**TRALI**	Transfusion-Related Acute Lung Injury
**TTI**	Transfusion-Transmitted Infection
**TTP**	Thrombotic Thrombocytopenic Purpura
**vCJD**	Variant Creutzfeld–Jakob disease
**VMP**	Validation Master Plan

REFERENCES

Recommendations and resolutions of the Council of Europe in the field of blood transfusion

Resolution (78) 29	on harmonisation of legislations of member states relating to removal, grafting and transplantation of human substances
Recommendation No. R (79) 5	concerning international exchange and transportation of human substances
Recommendation No. R (80) 5	on blood products for the treatment of haemophiliacs
Recommendation No. R (81) 5	concerning antenatal administration of anti-D immunoglobulin
Recommendation No. R (81) 14	on preventing the transmission of infectious diseases in the international transfer of blood, its components and derivatives
Recommendation No. R (83) 8	on preventing the possible transmission of acquired immune deficiency syndrome (AIDS) from affected blood donors to patients receiving blood or blood products
Resolution 812 (1983)	of the Parliamentary Assembly on acquired immune deficiency syndrome (AIDS)
Recommendation No. R (84) 6	on the prevention of the transmission of malaria by blood transfusion
Recommendation No. R (85) 5	on a model curriculum for the training of specialists in blood transfusion
Recommendation No. R (85) 12	on the screening of blood donors for the presence of AIDS markers
Recommendation No. R (86) 6	on guidelines for the preparation, quality control and use of fresh frozen plasma (FFP)
Recommendation No. R (87) 25	concerning a common European public health policy to fight the acquired immunodeficiency syndrome (AIDS)
Recommendation No. R (88) 4	on the responsibilities of health authorities in the field of blood transfusion
Recommendation No. R (90) 3	concerning medical research on human beings
Recommendation No. R (90) 9	on plasma products and European self-sufficiency
Recommendation No. R (93) 4	concerning clinical trials involving the use of components and fractionated products derived from human blood or plasma

Recommendation No. R (95) 14	on the protection of health of donors and recipients in the area of blood transfusion
Recommendation No. R (95) 15	On the preparation, use and quality assurance of blood components
Recommendation No. R (96) 11	on documentation and record-keeping to guarantee the traceability of blood and blood products , especially in hospital
Recommendation No. R (98) 2	on provision of haematopoietic progenitor cells
Recommendation No. R (98) 10	on the use of human red blood cells for the preparation of oxygen-carrying substances
Recommendation Rec (2001) 4	on the prevention of the possible transmission of variant Creutzfeldt–Jakob disease (vCJD) by blood transfusion
Recommendation Rec (2002) 11	on the hospital's and clinician's role in the optimal use of blood and blood products
Recommendation Rec (2003) 11	on the introduction of pathogen inactivation procedures for blood components
Recommendation Rec (2004) 8	on autologous cord blood banks
Recommendation Rec 2004) 18	on teaching transfusion medicine to nurses
Resolution Res (2008) 5	on donor responsibility and limitation of donation of blood and blood components
Resolution CM/Res (2013) 3	on sexual behaviours of blood donors that have an impact on transfusion safety
Resolution CM/Res (2015) 2	on principles concerning human immunoglobulin therapies for immunodeficiency and other diseases
Resolution CM/Res (2015) 3	on principles concerning haemophilia therapies

N.B. The figure in parentheses indicates the year of adoption.

Council of Europe publications in the field of blood transfusion

1976	Production and use of cellular blood components for transfusion. Study Director: B. Bucher with M. Benbunan, H. Heisto, U. Reesink
1978	Indications for the use of albumin, plasma protein solutions and plasma substitutes. Study Director: J. O'Riordan with M. Aebischer, J. Darnborough and I. Thoren
1980	Preparation and use of coagulation factors VIII and IX for transfusion. Study Director: R. Masure with G. Myllyla, I. Temperley and K. Stampli
1981	Assessment of the risks of transmitting infectious diseases by international transfer of blood, its components and derivatives. Study Director: W. Weise with T. Nielsen, P. Skinhot, J. P. Saleun
1982	European Co-operation in the field of blood: miscellany reports on the occasion of the 20th anniversary of the Committee of Experts on Blood Transfusion and Immuno-haematology 1962–1982. P. Cazal, A. André, P. Lundsgaard-Hansen, W. Weise, R. Butler, C. P. Engelfriet, and A. Hässig
1983	Essential aspects of tissue typing. B. Bradley and S. Gore
1985	Study on the current position of training programmes for future specialists in blood transfusion in Council of Europe member states and in Finland. Study Director: E. Freiesleben with A. André, A. Franco, B. Baysal, J. Cash
1986	Quality control in blood transfusion services. Study Director: E. Freiesleben, R. Butler, C. Hogman, W. Wagstaff
1987	Renal transplantation: sense and sensitisation. B. Bradley and S. Gore, Martinus Nijhoff Publishers
1988	First European Symposium on quality in blood transfusion Résumé of lectures (publication of the Health Division of the Council of Europe)
1989	European Course on Blood transfusion (Athens, March 1988) Compendium of lecturers (publication of the Health Division of the Council of Europe)
1990	Blood transfusion: 2nd European Course (Madrid 1990) Compendium of lecturers (publication of the Health Division of the Council of Europe)
1992	Impact of the Aids epidemic on health care services and planning in Europe (publication of the Health Division of the Council of Europe)
	Plasma products and European self-sufficiency: collection, preparation and use. Study Director: J. Leikola with W. van Aken, C. Hogman, D. Lee, M. Muglia, H. Schmitt

1993	Blood transfusion in Europe: a 'white paper'. Safe and sufficient blood in Europe by Piet J. Hagen
	Survey of blood transfusion services of central and eastern European countries and their co-operation with western transfusion services. Report by H. T. Heiniger
	The collection and use of human blood and plasma in Europe. Prof. Dr W.G. van Aken
1995	Guide on the preparation, use and quality assurance in blood components (appendix to Recommendation No. R (95) 15)
1997	Collection and use of blood and plasma in Europe (member States of the Council of Europe not members of the European Union). Study 1995, report by Dr Rejman
	Activities of blood banks in relation to bone marrow transplantations. Study Director: I.M. Francklin; Group members S. Koskimies, R. Kroczek, M. Reti, L. de Waal, R. Arrieta, F. Carbonell-Uberos
1998	Blood transfusion: half a century of contribution by the Council of Europe. Report by Prof. Dr B. Genetet
2000	Collection and use of human blood and plasma in the non-European Union Council of Europe member states in 1997. Report by Dr Rejman
	Autologous blood donation and transfusion in Europe – 1997 data. Report by Prof. Politis
2001	Pathogen inactivation of labile blood products. Study Director: Prof. A. Morell
2002	Autologous blood donation and transfusion in Europe – 2000 data. Report by Prof. Politis
2004	Collection, testing and use of blood and blood products in Europe – 2001 data. Report by Drs C.L. van der Poel, M.P. Janssen and M.E. Behr-Gross
2005	Collection, testing and use of blood and blood products in Europe – 2002 data. Report by Drs C.L. van der Poel, M.P. Janssen and M.E. Behr-Gross
2007	Collection, testing and use of blood and blood products in Europe – 2003 data. Report by Drs C.L. van der Poel, M.P. Janssen and M.E. Behr-Gross
2008	Collection, testing and use of blood and blood products in Europe – 2004 data. Report by Drs C.L. van der Poel, M.P. Janssen and M.E. Behr-Gross

2011	Trends and observations on the collection, testing and use of blood and blood components in Europe – 2001-2005 data. Report by Drs C.L. van der Poel, M.P. Janssen and M.E. Behr-Gross
	Collection, testing and use of blood and blood products in Europe – 2006 data. Report by Drs C.L. van der Poel, M.P. Janssen and M.E. Behr-Gross
	Collection, testing and use of blood and blood products in Europe – 2007 data. Report by Drs C.L. van der Poel, M.P. Janssen and M.E. Behr-Gross
	Collection, testing and use of blood and blood products in Europe – 2008 data. Report by Drs C.L. van der Poel, M.P. Janssen and M.E. Behr-Gross
2013	Trends and observations on the collection, testing and use of blood and blood components in Europe – 2001-2008 data. Report by Drs C.L. van der Poel, M.P. Janssen and M.E. Behr-Gross
	Collection, testing and use of blood and blood products in Europe – 2009 data. Report by Drs C.L. van der Poel, M.P. Janssen and M.E. Behr-Gross
	Collection, testing and use of blood and blood products in Europe – 2010 data. Report by Drs C.L. van der Poel, M.P. Janssen and M.E. Behr-Gross
2014	Collection, testing and use of blood and blood products in Europe – 2011 data. Report by Drs L.R. van Hoeven, M.P. Janssen and G. Rautmann
2015	Trends and observations on the collection, testing and use of blood and blood components in Europe – 2001–2011 data. Report by Drs L.R. van Hoeven, M.P. Janssen and G. Rautmann
	Collection, testing and use of blood and blood products in Europe – 2012 data. Report by Drs L.R. van Hoeven, M.P. Janssen and G. Rautmann
2016	Collection, testing and use of blood and blood products in Europe – 2013 data. Report by Drs L.R. van Hoeven, M.P. Janssen and G. Rautmann
2017	Collection, testing and use of blood and blood products in Europe – 2014 data. Report by Drs M.P. Janssen and G. Rautmann

The use of blood components represents the only therapy available for many seriously ill patients who suffer from acute or chronic diseases.

To provide all those working in the field of transfusion medicine – from blood services to hospital departments to regulators – with a compendium of measures designed to ensure the safety, quality and efficacy of blood components, the Council of Europe has developed a guide as a technical annex to its Recommendation No. R (95) 15 on the preparation, use and quality assurance of blood components. The Guide contains recommendations for blood establishments on blood collection, blood components, technical procedures, transfusion practices and quality systems. It represents the basis for a large number of national regulations, as well as for the blood directives of the European Commission.

This is the 19th Edition of the Guide, compiled by leading European experts under the aegis of the European Committee (Partial Agreement) on Blood Transfusion (CD-P-TS).

For matters dealing with the use of organs and tissues and cells, see the Council of Europe *Guide to the quality and safety of organs for transplantation* and *Guide to the quality and safety of tissues and cells for human application*, respectively.

ENG

www.edqm.eu

The Council of Europe is the continent's leading human rights organisation. It comprises 47 member states, 28 of which are members of the European Union. The European Directorate for the Quality of Medicines & HealthCare (EDQM) is a directorate of the Council of Europe. Its mission is to contribute to the basic human right of access to good quality medicines and healthcare and to promote and protect public health.

9 789287 184153

www.edqm.eu/store
ISBN 978-92-871-8415-3
€ 30

European Directorate for the Quality of Medicines & HealthCare | Direction européenne de la qualité du médicament & soins de santé

COUNCIL OF EUROPE
CONSEIL DE L'EUROPE

19th Edition

Guide to the preparation, use and quality assurance of blood components

EDQM
