# Supplementary material for: Ultrastructure of human platelet concentrates after treatment with pathogen reduction technologies for prolonged storage
Source: Front Med (Lausanne). 2025 Oct 20;12:1682909. doi: 10.3389/fmed.2025.1682909 (PMC12580302; doi:10.3389/fmed.2025.1682909)
Supplement: Supplementary file 2 [file Data_Sheet_2.pdf]

Platelet Concentrate

Platelets (thrombocytes) are the cellular elements of haemostasis. After injury to blood vessels, platelets attach to the damaged vessel walls to form a plug. This plug is further reinforced by the incorporation of protein strands.

Platelet concentrates (PCs) can either be obtained from whole blood donations (4 to 6 donations are combined for this purpose) or using a mechanical procedure. An anticoagulant solution is always added during the collection of PCs. They are stored in donor plasma, which can be replaced with a plasma substitute solution. PCs are stored at temperatures between +20°C and +24°C under constant movement.

Search results 1 to 25 from a total of 180

Name	Marketing Authori-sation Holder	License Number	License Date	Further Information
Apherese-Thrombozytenkonzentrat (KA)	Städtisches Klinikum Karlsruhe gGmbH - Abteilung für Transfusionsmedizin / Blutspendezentrale	PEI.H.00733.01.1	31.05.1999	PharmNet.Bund [https://portal.dimdi.de/amguifree/am/searchresult.xhtml?accessid=amis_off_am_ppv&directdisplay=true&gripsQuery=STATUS=CURRENT+AND+ENR=2601332]
Bestrahltes Apherese-Thrombozytenkonzentrat (KA)	Städtisches Klinikum Karlsruhe gGmbH - Abteilung für Transfusionsmedizin / Blutspendezentrale	PEI.H.00751.01.1	28.06.1999	PharmNet.Bund [https://portal.dimdi.de/amguifree/am/searchresult.xhtml?accessid=amis_off_am_ppv&directdisplay=true&gripsQuery=STATUS=CURRENT+AND+ENR=2601368]
Bestrahltes Pool-Thrombozytenkonzentrat - W	DRK-Blutspendedienst West gGmbH der Landesverbände Nordrhein, Westfalen-Lippe, Rheinland-Pfalz und Saarland, Institut für Transfusionsmedizin in Hagen	PEI.H.01242.01.1	20.01.2000	PharmNet.Bund [https://portal.dimdi.de/amguifree/am/searchresult.xhtml?accessid=amis_off_am_ppv&directdisplay=true&gripsQuery=STATUS=CURRENT+AND+ENR=2601620]
Bestrahltes Pool-Thrombozytenkonzentrat (E)	Universitätsklinikum Essen	PEI.H.03447.01.1	25.06.2008	PharmNet.Bund [https://portal.dimdi.de/amguifree/am/searchresult.xhtml?accessid=amis_off_am_ppv&directdisplay=true&gripsQuery=STATUS=CURRENT+AND+ENR=2603728#__DEFANCHOR__]
Bestrahltes Pool-Thrombozytenkonzentrat (KA)	Städtisches Klinikum Karlsruhe gGmbH	PEI.H.02722.01.1	06.06.2006	PharmNet.Bund [https://portal.dimdi.de/amguifree/am/searchresult.xhtml?accessid=amis_off_am_ppv&directdisplay=true&gripsQuery=STATUS=CURRENT+AND+ENR=2603092]

Name	Marketing Authorisation Holder	License Number	License Date	Further Information
Bestrahltes Pool-Thrombozytenkonzentrat B-PTK/TUE	Zentrum für Klinische Transfusionsmedizin Tübingen gGmbH (ZKT)	PEI.H.01702.01.1	01.02.2001	PharmNet.Bund [https://portal.dimdi.de/amguifree/am/searchresult.xhtml?accessid=amis_off_am_ppv&directdisplay=true&gripsQuery=STATUS=CURRENT+AND+ENR=2601941]
Bestrahltes Thrombozytapheresekonzentrat	Blutspendedienst der Landesverbaende des DRK Niedersachsen, Sachsen-Anhalt, Thueringen, Oldenburg und Bremen gGmbH	PEI.H.01259.01.1	04.03.2009	PharmNet.Bund [https://portal.dimdi.de/amguifree/am/searchresult.xhtml?accessid=amis_off_am_ppv&directdisplay=true&gripsQuery=STATUS=CURRENT+AND+ENR=2601632]
Bestrahltes Thrombozytapheresekonzentrat - BS	Städtisches Klinikum Braunschweig gGmbH	PEI.H.03629.01.1	04.03.2009	PharmNet.Bund [https://portal.dimdi.de/amguifree/am/searchresult.xhtml?accessid=amis_off_am_ppv&directdisplay=true&gripsQuery=STATUS=CURRENT+AND+ENR=2604143]
Bestrahltes Thrombozytapheresekonzentrat (E)	Universitätsklinikum Essen	PEI.H.03449.01.1	23.04.2008	PharmNet.Bund [https://portal.dimdi.de/amguifree/am/searchresult.xhtml?accessid=amis_off_am_ppv&directdisplay=true&gripsQuery=STATUS=CURRENT+AND+ENR=2603730#__DEFANCHOR__]
Bestrahltes Thrombozytapheresekonzentrat in Additivlösung - W	DRK-Blutspendedienst West gGmbH der Landesverbände Nordrhein, Westfalen-Lippe, Rheinland-Pfalz und Saarland - Institut für Transfusionsmedizin in Hagen	PEI.H.00030.01.1	06.04.1999	PharmNet.Bund [https://portal.dimdi.de/amguifree/am/searchresult.xhtml?accessid=amis_off_am_ppv&directdisplay=true&gripsQuery=STATUS=CURRENT+AND+ENR=2600975]
Bestrahltes Thrombozytapheresekonzentrat, plasmared. Th-S	Institut für Transfusionsmedizin Suhl gGmbH	PEI.H.00471.01.1	14.07.1999	PharmNet.Bund [https://portal.dimdi.de/amguifree/am/searchresult.xhtml?accessid=amis_off_am_ppv&directdisplay=true&gripsQuery=STATUS=CURRENT+AND+ENR=2601199]
Bestrahltes Thrombozytenhochkonzentrat - Id (AC)	Universitätsklinikum Aachen	PEI.H.04492.01.1	19.12.2008	PharmNet.Bund [https://portal.dimdi.de/amguifree/am/searchresult.xhtml?accessid=amis_off_am_ppv&directdisplay=true&gripsQuery=STATUS=CURRENT+AND+ENR=2600334]

Name	Marketing Authorisation Holder	License Number	License Date	Further Information
Bestrahltes Thrombozytenkonzentrat (Apherese) B-TKZ/TUE	Zentrum für Klinische Transfusionsmedizin Tübingen gGmbH (ZKT)	PEI.H.01694.01.1	07.05.2001	PharmNet.Bund [https://portal.dimdi.de/amguifree/am/searchresult.xhtml?accessid=amis_off_am_ppv&directdisplay=true&gripsQuery=STATUS=CURRENT+AND+ENR=2601933]
Bestrahltes Thrombozytenkonzentrat N-W	DRK-Blutspendedienst West gGmbH der Landesverbände Nordrhein, Westfalen-Lippe, Rheinland-Pfalz und Saarland	PEI.H.04369.01.1	29.10.2010	PharmNet.Bund [https://portal.dimdi.de/amguifree/am/searchresult.xhtml?accessid=amis_off_am_ppv&directdisplay=true&gripsQuery=STATUS=CURRENT+AND+ENR=2604183]
Bestrahltes Thrombozytenkonzentrat-W	DRK-Blutspendedienst West gGmbH der Landesverbände Nordrhein, Westfalen-Lippe Rheinland-Pfalz und Saarland - Institut für Transfusionsmedizin in Hagen	PEI.H.00995.01.1	22.11.2001	PharmNet.Bund [https://portal.dimdi.de/amguifree/am/searchresult.xhtml?accessid=amis_off_am_ppv&directdisplay=true&gripsQuery=STATUS=CURRENT+AND+ENR=2601504]
Bestrahltes, gepooltes Thrombozytenkonzentrat Th-S	Blutspendezentrum Augsburg GmbH	PEI.H.02398.01.1	07.11.2001	PharmNet.Bund [https://portal.dimdi.de/amguifree/am/searchresult.xhtml?accessid=amis_off_am_ppv&directdisplay=true&gripsQuery=STATUS=CURRENT+AND+ENR=1355308]
Filt. Thrombohochkonz. SB-FTHK maschinell	Blutspendezentrale Saar-Pfalz gGmbH	PEI.H.02153.01.1	08.06.2006	
Filt. Thrombohochkonz. SB-FTHK maschinell/bestrahlt	Blutspendezentrale Saar-Pfalz gGmbH	PEI.H.03286.01.1	22.01.2009	PharmNet.Bund [https://portal.dimdi.de/amguifree/am/searchresult.xhtml?accessid=amis_off_am_ppv&directdisplay=true&gripsQuery=STATUS=CURRENT+AND+ENR=2603336]
Gefiltertes Thrombozytapheresekonzentrat ACD-A (UKGM)	Universitätsklinikum Gießen und Marburg GmbH	PEI.H.01308.01.1	01.08.2000	PharmNet.Bund [https://portal.dimdi.de/amguifree/am/searchresult.xhtml?accessid=amis_off_am_ppv&directdisplay=true&gripsQuery=STATUS=CURRENT+AND+ENR=2601663]
Gefiltertes Thrombozytapheresekonzentrat ACD-A (UKGM), bestrahlt	Universitätsklinikum Giessen und Marburg GmbH	PEI.H.00888.01.1	29.09.1999	PharmNet.Bund [https://portal.dimdi.de/amguifree/am/searchresult.xhtml?accessid=amis_off_am_ppv&directdisplay=true&gripsQuery=STATUS=CURRENT+AND+ENR=2601444]

Name	Marketing Authori-sation Holder	License Number	License Date	Further Information
Gefiltertes Thrombozytenkonzentrat CPD (UKGM)	Universitätsklinikum Giessen und Marburg GmbH	PEI.H.02342.01.1	27.09.2001	PharmNet.Bund [https://portal.dimdi.de/amguifree/am/searchresult.xhtml?accessid=amis_off_am_ppv&directdisplay=true&gripsQuery=STATUS=CURRENT+AND+ENR=2602853]
Gefiltertes Thrombozytenkonzentrat CPD (UKGM), bestrahlt	Universitätsklinikum Giessen und Marburg GmbH	PEI.H.02793.01.1	12.08.2005	PharmNet.Bund [https://portal.dimdi.de/amguifree/am/searchresult.xhtml?accessid=amis_off_am_ppv&directdisplay=true&gripsQuery=STATUS=CURRENT+AND+ENR=2603156]
Gepooltes, gefiltertes Thrombozytenkonzentrat Th-S	Institut für Transfusionsmedizin Suhl gGmbH	PEI.H.01885.01.1	23.08.2005	PharmNet.Bund [https://portal.dimdi.de/amguifree/am/searchresult.xhtml?accessid=amis_off_am_ppv&directdisplay=true&gripsQuery=STATUS=CURRENT+AND+ENR=2602093]
Human-Thrombozytenkonzentrat AL-AD S BSD/BRK - bestrahlt	Blutspendedienst des Bayerischen Roten Kreuzes gGmbH	PEI.H.04244.01.1	19.02.2008	PharmNet.Bund [https://portal.dimdi.de/amguifree/am/searchresult.xhtml?accessid=amis_off_am_ppv&directdisplay=true&gripsQuery=STATUS=CURRENT+AND+ENR=2600912#__DEFANCHOR__]
Human-Thrombozytapheresekonzentrat (UKB)	Universitätsklinikum Bonn	PEI.H.03162.01.1	14.09.2004	PharmNet.Bund [https://portal.dimdi.de/amguifree/am/searchresult.xhtml?accessid=amis_off_am_ppv&directdisplay=true&gripsQuery=STATUS=CURRENT+AND+ENR=0171138]

Disclaimer

The list of medicines contains the products that have a valid marketing authorization. It contains no information as to whether the preparations are available on the market.

The information contained in the Federal Gazette, which is the official publication organ of the Paul-Ehrlich-Institut, is legally binding.

As of: PEI announcement No. 531 in BAnz AT 17.07.2025 B3.

Summary of Product Characteristics (SmPCs) and the Package Leaflet (PL)

Where the European Medicines Agency (EMA) offers further information, you will find a link to the EPAR (European public assessment report) in the table.

If user and technical information or public assessment reports are available in PharmNet.Bund, the Federal and State Pharmaceutical Information Portal, these are directly linked to PharmNet.Bund in the table.

The Paul-Ehrlich-Institut is an Agency of the German Federal Ministry of Health.
Its research and control activities promote the quality, efficacy and safety of biological medicinal products.

© Paul-Ehrlich-Institut 2025
